# Supplementary material for: Hybrids of Sterically Hindered Phenols and Diaryl Ureas: Synthesis, Switch from Antioxidant Activity to ROS Generation and Induction of Apoptosis
Source: Int J Mol Sci. 2023 Aug 10;24(16):12637. doi: 10.3390/ijms241612637 (PMC10454409; doi:10.3390/ijms241612637)
Supplement: Supplementary file 1 [file ijms-24-12637-s001.zip › ijms-2533435-supplementary.pdf]

# Supporting Information

# Hybrids of sterically hindered phenols and diaryl ureas: syn-thesis, switch from antioxidant activity to ROS-generation and induction of apoptosis

Elmira Gibadullina[1,*], Margarita Neganova[1,2,], Yulia Aleksandrova[1,2], Nguyen Hoang Bao Tran[3], Alexandra Voloshina[1], Mikhail Khrizanforov[1,], Nguyen Thi Thu[3], Ekaterina Vinyukova[2], Konstantin Volcho[4], Dmitry Tsypyshev[4], Anna Lyubina[1], Syumbelya Gumerova[1], Anna Strelnik[1], Julia Voronina[5], Daut Islamov[6], Rakhmetulla Zhapparbergenov[7,*], Nurbol Appazov[7], Beauty Chabuka[8], Kimberley Christopher[8], Alexander Burilov[1], Oleg Sinyashin[1] and Igor Alabugin[1,8]

1

1 Arbuzov Institute of Organic and Physical Chemistry, FRC Kazan Scientific Center, Russian Academy of Sciences, Akad. Arbuzov st. 8, Kazan, 420088, Russia;
2 Institute of Physiologically Active Compounds at Federal Research Center of Problems of Chemical Physics and Medicinal Chemistry, Russian Academy of Sciences, Severnij pr. 1, Chernogolovka, 142432, Russia;
3 The Kazan National Research Technological University, Karl Marx st., 68, Kazan, 420015, Russia;
4 Department of Medicinal Chemistry, Novosibirsk Institute of Organic Chemistry, Lavrentiev av. 9, Novosibirsk, 630090, Russia;
5 N. S. Kurnakov Institute of General and Inorganic Chemistry RAS, Leninskii prospekt, 31, Moscow, 119071, Russia;
6 Laboratory for structural analysis of biomacromolecules, Kazan Scientific Center of Russian Academy of Science, 31, Kremlevskaya, Kazan, 420008, Russia
7 Korkyt Ata Kyzylorda University, 120014, 29A, Aiteke Bi street, Kyzylorda, Republic of Kazakhstan;
8 Florida State University, Department of Chemistry and Biochemistry, 95 Chieftan Way, Tallahassee, Fl, 32306-3290, USA.
* Correspondence: elmirak@iopc.ru, elmirak_1978@mail.ru (EG); Tel.: +7-843-272-7324; neganova83@mail.ru (MN); Tel.: +7-843-272-7324.

Contents

Compound characterization data .......... 1

The X-ray diffraction data .......... 22

Electrochemical measurements .......... 27

Cartesian Coordinates .......... 30

NMR spectra of compounds .......... 33

References .......... 82

Compound characterization data

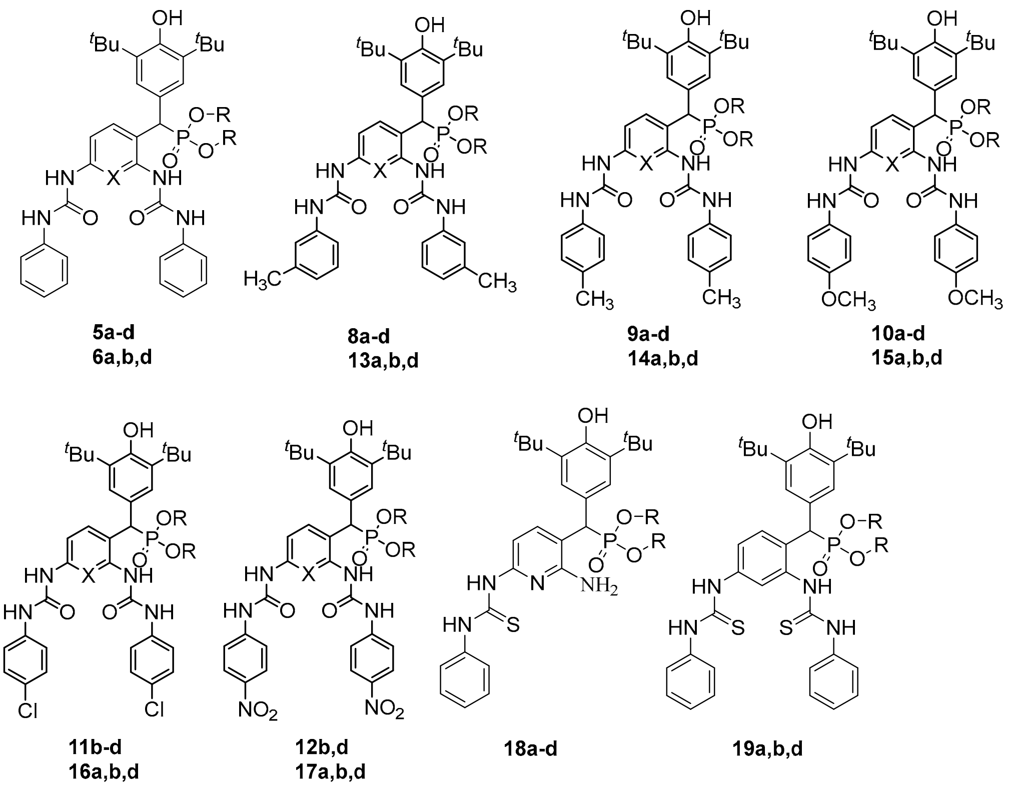


**Scheme 1.** Diarylmethylphosphonates containing sterically hindered phenols and (thio)urea moieties

The $^{1}H$- and $^{13}C$-NMR spectra were recorded on a Bruker AVANCE 400 spectrometer (Bruker BioSpin, Rheinstetten, Germany) operating at 400 MHz (for $^{1}H$ NMR), 101 MHz (for $^{13}C$ NMR) and 162 MHz (for $^{31}P$ NMR); Brucker spectrometers AVANCE*III*-500 (Bruker Corporation, Rheinstetten, Germany) operating at 500 MHz (for $^{1}H$ NMR) and 126 MHz (for $^{13}C$ NMR); Brucker spectrometers AVANCE*III*-600 (Bruker Corporation, Rheinstetten, Germany) operating at 600.13 MHz (for $^{1}H$ NMR), 150.19 MHz (for $^{13}C$ NMR) and 242.94 MHz (for $^{31}P$ NMR). Chemical shifts were measured in δ (ppm) with reference to the solvent δ = 2.56 ppm and 39.52 ppm for DMSO-$d_6$ for $^{1}H$ and $^{13}C$ NMR, respectively. IR spectra were recorded on IR Fourier spectrometer Tensor 37 (Bruker Optik GmbH, Germany) in the 400–3600 $cm^{-1}$ range in KBr. MALDI TOF/TOF-MS spectra were recorded on a Bruker Daltonics GmbH, Bremen, Germany instrument. Elemental analysis was performed on a CHNS-O Elemental Analyser EuroEA3028-HT-OM (EuroVector S.p.A., Milan, Italy). The melting points were determined on JK-MAM-4 Melting-point Apparatus with Microscope (SGW-X4 JINGKE SCIENTIFIC INSTRUMENT CO, Shanghai, China). The progress of reactions and the purity of products were monitored by TLC on Sorbfil UV-254 plates (Sorbpolimer, Krasnodar, Russia); the chromatograms were developed under UV light.

Dialkyl/diphenyl[(3,5-di-tert-butyl-4-hydroxyphenyl)(2,6-diaminopyridin-3-yl)methyl]phosphonate 3a-d (1.0 mmol) or dialkyl/diphenyl[(3,5-di-tert-butyl-4-

hydroxyphenyl)(2,4-diaminophenyl)methyl]phosphonate 4a,b,d was synthesized according to the literature [1,2].

**General procedure for the synthesis of compounds 5a-d; 6a,b,d; 8a-d - 10a-d; 11c,d - 12c,d; 13a,b,d - 17a,b,d**. To a 5 mL chloroform solution of dialkyl/diphenyl[(3,5-di-*tert*-butyl-4-hydroxyphenyl)(2,4-diaminophenyl)methyl]phosphonate 2a,b,d (1.0 mmol) or dialkyl/diphenyl[(3,5-di-*ter*t-butyl-4-hydroxyphenyl)(2,6-diaminopyridin-3-yl)methyl]phosphonate 3a-d (1.0 mmol), the phenyl isocyanate (8.0 mmol), respective aryl isocyanate (8.0 - 12.0 mmol) or phenyl isothiocyanate (8.0 mmol) was added. The resulting solutions were on stirred until a precipitation formed at ambient temperature for **5a-d**; **6a,b,d** – 4 h, for **8a-d** - 12 h and for **9a-d**; **10a-d**; **11c,d**; **12c,d**; **13a,b,d** - **17a,b,d** 3 h, for **18a-d** and **19a,b,d** heated under reflux 15 h. The precipitate was filtered off, washed once with ethyl acetate and hexane and dried under vacuum (0.06 mm Hg) at 40 °C to constant weight. The resulting compounds were urea compounds **5a-d; 6a,b,d; 8a-d - 10a-d; 11c,d; 12c,d; 13a,b,d - 17a,b,d** (46–91% yield) and thiourea compounds **18a-d and 19a,b,d** (70–80% yield) as into gray or white amorphous solids.

**Dimethyl[(2,6-bis(3-phenylureido)pyridin-3-yl)(3,5-di-*tert*-butyl-4-hydroxyphenyl)methyl]phosphonate 5a**. Gray-white solid, yield 0.57 g (85%), M.p.: 207–208°C. IR (KBr), *ν*, $cm^{-1}$: 757 (P–C), 1040, 1058 (P–O–$C_{alk}$), 1228 (P=O), 1599 (C=$C_{arom}$), 1677 (C=O), 3306 (NH), 3636 (OH). $^1$H NMR (400.05 MHz, DMCO-*d*$_6$), *δ*, ppm: 1.34 [s, 18H, C($CH_3$)$_3$], 3.54 (d, 3H, $^3J_{PH}$ = 10.7 Hz, $OCH_3$), 3.58 (d, 3H, $^3J_{PH}$ = 10.7 Hz, $OCH_3$), 4.93 (d, 1H, $^2J_{PH}$ = 26.9 Hz, CHP), 6.93 (s, 1H, OH), 6.96 (d, 1H, $^3J_{HH}$ = 8.0 Hz, $CH_{py}$), 6.99 (t, 1H, $^3J_{HH}$ = 7.4 Hz, NHC$_6$H$_5$), 7.04 (t, 1H, $^3J_{HH}$ = 7.4 Hz, NHC$_6$H$_5$), 7.23 (t, 2H, $^3J_{HH}$ = 7.9 Hz, NHC$_6$H$_5$), 7.30-7.34 [m, 4H, NHC$_6$H$_5$ and CHCC($CH_3$)$_3$], 7.59 (d, 2H, $^3J_{HH}$ = 7.6 Hz, NHC$_6$H$_5$), 7.65 (d, 2H, $^3J_{HH}$ = 7.7 Hz, NHC$_6$H$_5$), 8.02 (dd, 1H, $^3J_{HH}$ = 8.5 Hz, $^4J_{PH}$ = 1.5 Hz $CH_{py}$), 9.00 (1H, NH), 9.50 (1H, NH), 9.86 (1H, NH), 10.51 (1H, NH). $^{13}$C{$^1$H} NMR (100.60 MHz, DMCO-*d*$_6$), *δ*, ppm: 30.81 [C($CH_3$)$_3$], 35.07 [C($CH_3$)$_3$], 41.66 (d, $^1J_{PC}$ = 138.8 Hz, CHP), 53.04 (d, $^2J_{PC}$ = 7.1 Hz, $OCH_3$), 53.72 (d, $^2J_{PC}$ = 6.9 Hz, $OCH_3$), 106.24 ($CH_{py}$), 114.72 (d, $^2J_{PC}$ = 3.6 Hz, $C_{py}$), 119.72 (NHC$_6$H$_5$), 120.02 (NHC$_6$H$_5$), 122.94 (NHC$_6$H$_5$), 123.03 (NHC$_6$H$_5$), 125.98 [d, $^3J_{PC}$ = 7.9 Hz, CHCC($CH_3$)$_3$], 126.84 (d, $^2J_{PC}$ = 4.6 Hz, CCHP), 128.91 (NHC$_6$H$_5$), 129.16 (NHC$_6$H$_5$), 139.63 (NHC$_6$H$_5$), 139.68 [CC($CH_3$)$_3$], 141.31 (d, $^3J_{PC}$ = 5.0 Hz, $CH_{py}$), 147.86 (d, $^3J_{PC}$ = 10.7 Hz, $C_{py}$), 150.09 ($C_{py}$), 152.32 (C=O), 152.44 (C=O), 153.46 (COH). $^{31}$P NMR (161.94 MHz, DMCO-*d*$_6$), *δ*, ppm: 28.54. Anal. Calcd for $C_{36}H_{44}N_5O_6P$ (%): C, 64.18; H, 6.58; N, 10.39; P, 4.60. Found (%): C, 64.21; H, 6.60; N, 10.41; P, 4.63. HRMS (MALDI-TOF) *m*/*z* for $C_{36}H_{44}N_5O_6P$: calc. 673.30 $[M]^+$, found 674.63 $[M+H]^+$, 696.66 $[M+Na]^+$, 712.59 $[M+K]^+$.

**Diethyl[(2,6-bis(3-phenylureido)pyridin-3-yl)(3,5-di-*tert*-butyl-4-hydroxyphenyl)methyl]phosphonate 5b**. Gray-white solid, yield 0.56 g (80%), M.p.: 237–238°C. IR (KBr), *ν*, $cm^{-1}$: 758, 1022, 1049, 1228, 1600, 1667, 3314, 3632. $^1$H NMR (600.13 MHz, DMCO-*d*$_6$), *δ*, ppm: 1.01 (t, 6H, $^3J_{HH}$ = 7.0 Hz, $OCH_2CH_3$), 1.10 (t, 6H, $^3J_{HH}$ = 6.9 Hz, $OCH_2CH_3$), 1.35 [s, 18H, C($CH_3$)$_3$], 3.84 (m, 1H, $OCH_2CH_3$), 3.95 (m, 3H, $OCH_2CH_3$), 4.86 (d, 1H, $^2J_{PH}$ = 27.5 Hz, CHP), 6.93 (s, 1H, OH), 6.94 (d, 1H, $^3J_{HH}$ = 8.4 Hz, $CH_{py}$), 6.99 (t, 1H, $^3J_{HH}$ = 7.3 Hz, NHC$_6$H$_5$), 7.04 (t, 1H, $^3J_{HH}$ = 7.3 Hz, NHC$_6$H$_5$), 7.24 (t, 2H, $^3J_{HH}$ = 7.4 Hz, NHC$_6$H$_5$), 7.30-7.34 [m, 4H, NHC$_6$H$_5$ and CHCC($CH_3$)$_3$], 7.59 (d, 2H, $^3J_{HH}$ = 7.8 Hz, NHC$_6$H$_5$), 7.67 (d, 2H, $^3J_{HH}$ = 8.1 Hz, NHC$_6$H$_5$), 8.02 (d, 1H, $^3J_{HH}$ = 7.9 Hz, $CH_{py}$), 9.00 (1H, NH), 9.50 (1H, NH), 9.85 (1H, NH), 10.57 (1H, NH). $^{13}$C{$^1$H} NMR (150.19 MHz, DMCO-*d*$_6$), *δ*, ppm: 16.46 (d, $^3J_{PC}$ = 5.4 Hz, $OCH_2CH_3$), 16.61 (d, $^3J_{PC}$ = 5.4 Hz, $OCH_2CH_3$), 30.84 [C($CH_3$)$_3$], 35.11 [C($CH_3$)$_3$], 42.41 (d, $^1J_{PC}$ = 138.4 Hz, CHP), 62.45 (d, $^2J_{PC}$ = 6.3 Hz, $OCH_2CH_3$), 62.84

(d, $^{2}J_{PC}$ = 6.3 Hz, $O\underline{C}H_2CH_3$), 106.12 ($CH_{py}$), 114.90 ($C_{py}$), 119.72 ($NHC_6H_5$), 120.12 ($NHC_6H_5$), 122.97 ($NHC_6H_5$), 123.04 ($NHC_6H_5$), 126.18 [d, $^{3}J_{PC}$ = 7.1 Hz, $\underline{C}HCC(CH_3)_3$], 127.04 ($\underline{C}CHP$), 128.94 ($NHC_6H_5$), 129.21 ($NHC_6H_5$), 139.64 ($NHC_6H_5$), 139.69 ($NHC_6H_5$), 139.76 [$\underline{C}C(CH_3)_3$], 141.30 ($CH_{py}$), 147.94 (d, $^{3}J_{PC}$ = 10.1 Hz, $C_{py}$), 150.05 ($C_{py}$), 152.30 (C=O), 152.52 (C=O), 153.45 (COH). $^{31}$P NMR (161.94 MHz, DMCO-*d*$_6$), *δ*, ppm: 25.41. Anal. Calcd for $C_{38}H_{48}N_5O_6P$ (%): C, 65.03; H, 6.89; N, 9.98; P, 4.41. Found (%): C, 64.98; H, 6.91; N, 9.94; P, 4.46. HRMS (MALDI-TOF) *m*/*z* for $C_{38}H_{48}N_5O_6P$: calc. 701.33 $[M]^+$, found 702.61 $[M+H]^+$, 724.65 $[M+Na]^+$, 740.65 $[M+K]^+$.

**Diisopropyl[(2,6-bis(3-phenylureido)pyridin-3-yl)(3,5-di-*tert*-butyl-4-hydroxyphenyl)methyl]phosphonate 5c**. Gray-white solid, yield 0.60 g (82%), M.p.: 138–139°C. IR (KBr), *ν*, cm$^{-1}$: 754, 993, 1235, 1598, 1713, 3367, 3629. $^{1}$H NMR (500.13 MHz, DMCO-*d*$_6$), *δ*, ppm: 0.82 [t, 3H, $^{3}J_{HH}$ = 6.2 Hz, $OCH(C\underline{H}_3)_2$], 1.01 [t, 3H, $^{3}J_{HH}$ = 6.2 Hz, $OCH(C\underline{H}_3)_2$], 1.06 [t, 3H, $^{3}J_{HH}$ = 6.2 Hz, $OCH(C\underline{H}_3)_2$], 1.19 [t, 3H, $^{3}J_{HH}$ = 6.2 Hz, $OCH(C\underline{H}_3)_2$], 1.35 [s, 18H, $C(CH_3)_3$], 4.41 [m, 1H, $OCH(C\underline{H}_3)_2$], 4.52 [m, 1H, $OCH(C\underline{H}_3)_2$], 4.74 (d, 1H, $^{2}J_{PH}$ = 26.9 Hz, CHP), 6.90 (s, 1H, OH), 6.91 (d, 1H, $^{3}J_{HH}$ = 7.5 Hz, $CH_{py}$), 7.00 (t, 1H, $^{3}J_{HH}$ = 7.4 Hz, $NHC_6\underline{H}_5$), 7.05 (t, 1H, $^{3}J_{HH}$ = 7.4 Hz, $NHC_6\underline{H}_5$), 7.24 (t, 2H, $^{3}J_{HH}$ = 7.4 Hz, $NHC_6\underline{H}_5$), 7.34 (t, 2H, $^{3}J_{HH}$ = 7.4 Hz, $NHC_6\underline{H}_5$), 7.37 [s, 2H, $C\underline{H}CC(CH_3)_3$], 7.59 (d, 2H, $^{3}J_{HH}$ = 8.5 Hz, $NHC_6\underline{H}_5$), 7.69 (d, 2H, $^{3}J_{HH}$ = 7.6 Hz, $NHC_6\underline{H}_5$), 8.07 (dd, 1H, $^{3}J_{HH}$ = 8.5 Hz, $^{4}J_{PH}$ = 1.5 Hz, $CH_{py}$), 9.03 (s, 1H, NH), 9.50 (s, 1H, NH), 9.57(s, 1H, NH), 10.65 (s, 1H, NH). $^{13}$C{$^{1}$H} NMR (125.76 MHz, DMCO-*d*$_6$), *δ*, ppm: 22.51 [d, $^{3}J_{PC}$ = 5.4 Hz, $OCH(\underline{C}H_3)_2$], 23.23 [d, $^{3}J_{PC}$ = 5.3 Hz, $OCH(\underline{C}H_3)_2$], 24.20 [d, $^{3}J_{PC}$ = 3.3 Hz, $OCH(\underline{C}H_3)_2$], 24.48 [d, $^{3}J_{PC}$ = 2.7 Hz, $OCH(\underline{C}H_3)_2$], 30.78 [$C(\underline{C}H_3)_3$], 35.07 [$\underline{C}(CH_3)_3$], 43.24 (all d, $^{1}J_{PC}$ = 139.4 Hz, CHP), 70.71 [d, $^{2}J_{PC}$ = 7.3 Hz, $O\underline{C}H(\underline{C}H_3)_2$], 71.39 [d, $^{2}J_{PC}$ = 7.1 Hz, $O\underline{C}H(\underline{C}H_3)_2$], 105.94 ($CH_{py}$), 116.09 ($C_{py}$), 119.57 ($NHC_6H_5$), 120.17 ($NHC_6H_5$), 122.27 ($NHC_6H_5$), 122.96 ($NHC_6H_5$), 126.30 [d, $^{3}J_{PC}$ = 8.0 Hz, $\underline{C}HCC(CH_3)_3$], 128.87 ($\underline{C}CHP$), 129.20 ($NHC_6H_5$), 129.23 ($NHC_6H_5$), 139.59 [$\underline{C}C(CH_3)_3$], 139.68 ($NHC_6H_5$), 139.74 ($NHC_6H_5$), 141.06 ($CH_{py}$), 147.85 (d, $^{3}J_{PC}$ = 10.5 Hz, $C_{py}$), 149.95 ($C_{py}$), 152.17 (C=O), 152.19 (C=O), 153.38 (OH). $^{31}$P NMR (202.46 MHz, DMCO-*d*$_6$), *δ*, ppm: 25.92. Anal. Calcd for $C_{40}H_{52}N_5O_6P$ (%): C, 65.83; H, 7.18; N, 9.60; P, 4.24. Found (%): C, 65.81; H, 7.20; N, 9.63; P, 4.22. HRMS (MALDI-TOF) *m*/*z* for $C_{40}H_{52}N_5O_6P$: calc. 729.37 $[M]^+$, found 730.72 $[M+H]^+$.

**Diphenyl[(2,6-bis(3-phenylureido)pyridin-3-yl)(3,5-di-*tert*-butyl-4-hydroxyphenyl)methyl]phosphonate 5d**. Gray-white solid, yield 0.65 g (81%), M.p.: 128–129°C. IR (KBr), *ν*, cm$^{-1}$: 754, 938, 1236, 1597, 1721, 3242, 3625. $^{1}$H NMR (500.13 MHz, DMCO-*d*$_6$), *δ*, ppm: 1.23, 1.31, 1.34, 1.35 [all s, 18H, $C(CH_3)_3$], 4.93, 5.01, 5.47, 5.84 (all d, 1H, $^{2}J_{PH}$ = 26.8 Hz, CHP), 6.63 (d, 2H, $^{3}J_{HH}$ = 8.3 Hz, $OC_6H_5$), 6.70 (d, 2H, $^{3}J_{HH}$ = 8.4 Hz, $OC_6H_5$), 6.92, 6.98 (all s, 1H, OH), 7.00–7.36 [m, 7H, $CH_{py}$, $NHC_6\underline{H}_5$, $C\underline{H}CC(CH_3)_3$ and $OC_6H_5$], 7.47 (t, 2H, $^{3}J_{HH}$ = 7.2 Hz, $OC_6H_5$), 7.51 (d, 2H, $^{3}J_{HH}$ = 7.1 Hz, $OC_6H_5$), 7.57 (d, 2H, $^{3}J_{HH}$ = 7.5 Hz, $OC_6H_5$), 7.62 (d, 2H, $^{3}J_{HH}$ = 7.6 Hz, $NHC_6\underline{H}_5$), 7.69 (d, 2H, $^{3}J_{HH}$ = 7.6 Hz, $NHC_6\underline{H}_5$), 8.20, 8.30, 8.53 (all d, 1H, $^{3}J_{HH}$ = 8.5 Hz, $CH_{py}$), 8.64, 9.13 (all s, 1H, NH), 9.57(s, 1H, NH), 9.18, 9.86 (all s, 1H, NH), 10.53, 11.02 (s, 1H, NH). $^{13}$C{$^{1}$H} NMR (125.76 MHz, DMCO-*d*$_6$), *δ*, ppm: 30.38, 30.61, 30.68 [$C(\underline{C}H_3)_3$], 34.82, 34.99, 35.07 [$\underline{C}(CH_3)_3$], 42.80, 43.24, 44.84 (d, $^{1}J_{PC}$ = 139.6 Hz, CHP), 106.40, 107.23, 108.63, 113.59 ($CH_{py}$), 113.60, 113.71 ($C_{py}$), 118.67 ($OC_6H_5$), 119.05 ($OC_6H_5$), 119.98 ($NHC_6H_5$), 119.78 ($NHC_6H_5$), 119.98 ($OC_6H_5$), 120.06 ($OC_6H_5$), 120.58, 120.75 ($OC_6H_5$), 120.82, 121.00 ($OC_6H_5$), 122.12 ($OC_6H_5$), 122.85 ($OC_6H_5$), 122.99 ($NHC_6H_5$), 123.10 ($NHC_6H_5$), 124.64, 125.44, 125.74 [all d, $^{3}J_{PC}$ = 7.2 Hz, $\underline{C}HCC(CH_3)_3$], 126.53, 126.62, 126.76 (all d, $^{2}J_{PC}$ = 7.2 Hz, $\underline{C}CHP$), 128.84, 128.94 ($OC_6H_5$), 129.23, 129.41 ($OC_6H_5$), 129.92, 129.99 ($NHC_6H_5$), 130.20, 130.29 ($NHC_6H_5$), 139.26, 139.58 ($NHC_6H_5$), 139.63 ($NHC_6H_5$), 139.69 [$\underline{C}C(CH_3)_3$], 140.14, 141.13 ($CH_{py}$), 146.36, 147.12, 148.08 (all d, $^{3}J_{PC}$ = 6.7 Hz, $C_{py}$), 150.26, 150.34, 150.71 ($C_{py}$), 152.33, 152.38, 152.43 (d, $^{2}J_{PC}$ = 6.2 Hz, $OC_6H_5$), 153.01 (C=O), 153.10 (C=O), 153.94,

154.13, 154.99 (COH). $^{31}P$ NMR (202.46 MHz, DMCO-$d_6$), $\delta$, ppm: 18.41, 18.51, 19.29, 19.37. Anal. Calcd for $C_{46}H_{48}N_5O_6P$ (%): C, 69.25; H, 6.06; N, 8.78; P, 3.88. Found (%): C, 69.27; H, 6.09; N, 8.80; P, 3.91. HRMS (MALDI-TOF) *m/z* for $C_{46}H_{48}N_5O_6P$: calc. 797.33 $[M]^+$, found 798.72 $[M+H]^+$.

**Dimethyl[(2,4-bis(3-phenylureido)phenyl)(3,5-di-*tert*-butyl-4-hydroxyphenyl)methyl]phosphonate 6a**. White solid, yield 0.60 g (90%), M.p.: 240–241°C. IR (KBr), $\nu$, cm$^{-1}$: 754, 1038, 1058, 1222, 1600, 1641; 3326, 3628. $^1H$ NMR (500.13 MHz, DMCO-$d_6$), $\delta$, ppm: 1.33 [s, 18H, $C(CH_3)_3$], 3.50 (d, 3H, $^3J_{PH}$ =10.6 Hz, $OCH_3$), 3.52 (d, 3H, $^3J_{PH}$ =10.6 Hz, $OCH_3$), 4.75 (d, 1H, $^2J_{PH}$ = 26.4 Hz, CHP), 6.87 (s, 1H, OH), 6.97 (t, 2H, $^3J_{HH}$ = 7.3 Hz, $NHC_6\underline{H}_5$), 7.26–7.29 [m, 5H, $CH_{arom}$, $NHC_6\underline{H}_5$), 7.31 [s, 2H, $C\underline{H}CC(CH_3)_3$], 7.45 (t, 2H, $^3J_{HH}$ = 8.0 Hz, $NHC_6\underline{H}_5$), 7.66 (dd, 1H, $^3J_{HH}$ = 8.6 Hz, $^4J_{PH}$ = 1.5 Hz, $CH_{arom}$), 7.77 (s, 1H, $CH_{arom}$), 8.18 (s, 1H, NH), 8.57 (s, 1H, NH), 8.70 (s, 1H, NH), 8.85 (s, 1H, NH). $^{13}C\{^1H\}$ NMR (125.76 MHz, DMCO-$d_6$), $\delta$, ppm: 30.81 [$C(\underline{C}H_3)_3$], 35.01 [$\underline{C}(CH_3)_3$], 42.54 (d, $^1J_{PC}$ = 139.8 Hz, CHP), 53.34 (d, $^2J_{PC}$ = 7.1 Hz, $OCH_3$), 53.42 (d, $^2J_{PC}$ = 7.1 Hz, $OCH_3$), 114.45 ($CH_{arom}$), 114.86 ($CH_{arom}$), 118.49 ($NHC_6H_5$), 118.64 ($NHC_6H_5$), 122.20 ($NHC_6H_5$), 122.29 ($NHC_6H_5$), 123.63 ($\underline{C}CHP$), 126.00 [d, $^3J_{PC}$ = 8.0 Hz, $\underline{C}HCC(CH_3)_3$], 127.80 (d, $^2J_{PC}$ = 4.3 Hz, $C_{arom}$), 129.23 ($NHC_6H_5$), 129.83 ($CH_{arom}$), 137.10 (d, $^3J_{PC}$ = 10.5 Hz, $C_{arom}$), 139.09 ($C_{arom}$), 139.48 [$\underline{C}C(CH_3)_3$], 140.12 ($NHC_6H_5$), 140.34 ($NHC_6H_5$), 152.87 (C=O), 153.23 (COH), 153.36 (C=O). $^{31}P$ NMR (202.46 MHz, DMCO-$d_6$), $\delta$, ppm: 28.75. Anal. Calcd for $C_{37}H_{45}N_4O_6P$ (%): C, 66.06; H, 6.74; N, 8.33; P, 4.60. Found (%): C, 66.01; H, 6.73; N, 8.35; P, 4.58. HRMS (MALDI-TOF) *m/z* for $C_{37}H_{45}N_4O_6P$: calc. 672.57 $[M]^+$, found 695.58 $[M+Na]^+$, 711.62 $[M+K]^+$.

**Diethyl[(2,4-bis(3-phenylureido)phenyl)(3,5-di-*tert*-butyl-4-hydroxyphenyl)methyl]phosphonate 6b**. White solid, yield 0.59 g (85%), M.p.: 245–246°C. IR (KBr), $\nu$, cm$^{-1}$: 758, 1022, 1049, 1228, 1600, 1667, 3313, 3633. $^1H$ NMR (600.13 MHz, DMCO-$d_6$), $\delta$, ppm: 1.00 (t, 3H, $^3J_{HH}$ = 7.1 Hz, $OCH_2C\underline{H}_3$), 1.07 (t, 3H, $^3J_{HH}$ = 7.1 Hz, $OCH_2C\underline{H}_3$), 1.32 [s, 18H, $C(CH_3)_3$], 3.77 (m, 1H, $OC\underline{H}_2CH_3$), 3.83 (m, 1H, $OC\underline{H}_2CH_3$), 3.90 (m, 2H, $OC\underline{H}_2CH_3$), 4.68 (d, 1H, $^2J_{PH}$ = 26.2 Hz, CHP), 6.83 (s, 1H, OH), 6.96 (t, 2H, $^3J_{HH}$ = 6.5 Hz, $NHC_6\underline{H}_5$), 6.24-7.29 (m, 5H, $CH_{arom}$ and $NHC_6\underline{H}_5$), 7.30 [s, 2H, $C\underline{H}CC(CH_3)_3$], 7.44 (t, 4H, $^3J_{HH}$ = 8.3 Hz, $NHC_6\underline{H}_5$), 7.67 (dd, 1H, $^3J_{HH}$ = 8.3 Hz, $^4J_{PH}$ = 0.9 Hz, $CH_{arom}$), 7.77 (д, 1H, $^4J_{HP}$ = 1.5 Hz, $CH_{arom}$), 8.16 (s, 1H, NH), 8.56 (s, 1H, NH), 8.68 (s, 1H, NH), 8.87 (s, 1H, NH). $^{13C\{1H\}\ NMR}$ (150.19 MHz, DMCO-$d_6$), $\delta$, ppm: 16.43 (d, $^3J_{PC}$ = 5.5 Hz, $OCH_2\underline{C}H_3$), 16.59 (d, $^3J_{PC}$ = 5.2 Hz, $OCH_2\underline{C}H_3$), 30.79 [s, $C(\underline{C}H_3)_3$], 35.00 [$\underline{C}(CH_3)_3$], 43.15 (d, $^1J_{PC}$ = 139.0 Hz, CHP), 62.27 (d, $^2J_{PC}$ = 6.7 Hz, $O\underline{C}H_2CH_3$), 62.53 (d, $^2J_{PC}$ = 6.8 Hz, $O\underline{C}H_2CH_3$), 114.15 ($CH_{arom}$), 114.47 ($CH_{arom}$), 118.47 ($NHC_6H_5$), 118.64 ($NHC_6H_5$), 122.17 ($NHC_6H_5$), 122.27 ($NHC_6H_5$), 123.50 ($C_{arom}$), 126.13 [d, $^3J_{PC}$ = 7.9 Hz, $\underline{C}HCC(CH_3)_3$], 127.94 ($\underline{C}CHP$), 129.22 ($NHC_6H_5$), 129.80 (д, $^3J_{PC}$ = 4.8 Hz, $CH_{arom}$), 137.22 (d, $^3J_{PC}$ = 11.4 Hz, $C_{arom}$), 139.04 ($C_{arom}$), 139.39 [$\underline{C}C(CH_3)_3$], 140.12 ($NHC_6H_5$), 140.35 ($NHC_6H_5$), 152.87 (C=O), 153.15 (s, COH), 153.29 (C=O). $^{31}P$ NMR (242.94 MHz, DMCO-$d_6$), $\delta$, ppm: 26.72. Anal. Calcd for Calc. for $C_{39}H_{49}N_4O_6P$ (%): C, 66.84; H, 7.05; N, 7.99; P, 4.42. Found (%): C, 66.87; H, 7.02; N, 8.01; P, 4.45. HRMS (MALDI-TOF) *m/z* for $C_{39}H_{49}N_4O_6P$: calc. 700.34 $[M]^+$, found 723.06 $[M+Na]^+$, 738.95 $[M+K]^+$.

**Diphenyl[(2,4-bis(3-phenylureido)phenyl)(3,5-di-*tert*-butyl-4-hydroxyphenyl)methyl]phosphonate 6d**. White solid, yield 0.71 g (90%), M.p.: 251–252 °C. IR (KBr), $\nu$, cm$^{-1}$: 752, 936, 1209, 1599, 1644, 3329, 3627. $^1H$ NMR (600.13 MHz, DMCO-$d_6$), $\delta$, ppm: 1.29 [s, 18H, $C(CH_3)_3$], 5.20 (d, 1H, $^2J_{PH}$ = 27.5 Hz, CHP), 6.64 (d, 2H, $^3J_{HH}$ = 8.0 Hz, $OC_6H_5$), 6.93 (s, 1H, OH), 6.95-6.99 (m, 4H, $OC_6H_5$ and $NHC_6\underline{H}_5$), 7.09 (t, 1H, $^3J_{HH}$ = 7.4 Hz, $OC_6H_5$), 7.16-7.21 (m, 3H, $OC_6H_5$), 7.26-7.32 (m, 7H, $CH_{arom}$, $OC_6H_5$ and $NHC_6\underline{H}_5$), 7.39 [s, 2H, $C\underline{H}CC(CH_3)_3$], 7.45 (д, 2H, $^3J_{HH}$ = 8.0 Hz, $NHC_6\underline{H}_5$), 7.47 (д, 2H, $^3J_{HH}$ = 8.0 Hz, $NHC_6\underline{H}_5$), 7.82 (d, 1H, $^3J_{HH}$ = 7.7 Hz, $CH_{arom}$), 7.83 (s, 1H, $CH_{arom}$), 8.27 (1H, NH), 8.59 (1H, NH), 8.75 (1H, NH), 8.85 (1H, NH). $^{13}C\{^1H\}$ NMR (150.19

MHz, DMCO-*d6*), *δ*, ppm: 30.70 [C(C̲H3)3], 35.02 [C̲(CH3)3], 43.76 (d, $^1J_{PC}$ = 140.5 Hz, CHP), 114.67 ($CH_{arom}$), 115.14 ($CH_{arom}$), 118.57 ($NHC_6H_5$), 118.71 ($NHC_6H_5$), 120.63 ($OC_6H_5$), 120.94 ($OC_6H_5$), 122.29 ($NHC_6H_5$), 122.36 ($NHC_6H_5$), 122.90 ($C_{arom}$), 125.40 ($OC_6H_5$), 125.68 ($OC_6H_5$), 126.27 (C̲CHP), 126.55 [d, $^3J_{PC}$ = 7.8 Hz, C̲HCC($CH_3$)3], 129.27 ($NHC_6H_5$), 129.71 ($CH_{arom}$), 129.95 ($OC_6H_5$), 130.26 ($OC_6H_5$), 137.45 (d, $^3J_{PC}$ = 11.6 Hz, $C_{arom}$), 139.56 ($C_{arom}$), 139.75 [C̲C($CH_3$)3], 140.12 ($NHC_6H_5$), 140.32 ($NHC_6H_5$), 150.47 (d, $^2J_{PC}$ = 10.0 Hz, $OC_6H_5$), 150.80 (d, $^2J_{PC}$ = 8.9 Hz, $OC_6H_5$), 152.89 (C=O), 153.43 (C=O), 153.73 (s, COH). $^{31}$P NMR (242.94 MHz, DMCO-*d6*), *δ*, ppm: 20.22. Anal. Calcd for $C_{47}H_{49}N_4O_6P$ (%): C, 70.84; H, 6.20; N, 7.03; P, 3.89. Found (%): C, 70.87; H, 6.26; N, 7.06; P, 3.94. HRMS (MALDI-TOF) *m/z* for $C_{47}H_{49}N_4O_6P$: calc. 796.34 $[M]^+$, found 819.33 $[M+Na]^+$, 835.35 $[M+K]^+$.

**Dimethyl[(2-amino-6-(3-phenylureido)pyridin-3-yl)(3,5-di-*tert*-butyl-4-hydroxyphenyl)methyl]phosphonate 7a**. To a solution of **3a** (1 mmol) in 5 mL of chloroform were added phenyl isocyanate (2 mmol). The reaction mixture was stirred at ambient temperature for 2 h. The precipitate was filtered off, washed with hexane. From the mixture with **5a** were isolated **7a** white crystals in trace amounts. $^1$H NMR (600.13 MHz, DMCO-*d6*,), *δ*, ppm: 1.35 [s, 18H, C($CH_3$)3], 3.48 (d, 3H, $^3J_{HH}$ = 10.3 Hz, $OCH_3$), 3.55 (d, 3H, $^3J_{HH}$ = 10.3 Hz, $OCH_3$), 5.01 (d, 1H, $^2J_{PH}$ = 27.2 Hz, CHP), 6.15 (d, 1H, $^3J_{HH}$ = 8.6 Hz, $CH_{py}$), 6.31 (br.s, 2H, $NH_2$), 6.90 (s, 1H, OH), 6.99 (t, 1H, $^3J_{HH}$ = 7.3 Hz, $NHC_6\underline{H}_5$), 7.23 (t, 1H, $^3J_{HH}$ = 7.4 Hz, $NHC_6\underline{H}_5$), 7.22-7.34 [m, 3H, $NHC_6\underline{H}_5$ and CH̲CC($CH_3$)3], 7.66 (d, 2H, $^3J_{HH}$ = 8.3 Hz, $NHC_6\underline{H}_5$), 7.80 (d, 1H, $^3J_{HH}$ = 8.6 Hz, $CH_{py}$), 8.70 (s, 1H, NH), 11.85 (s, 1H, NH). $^{31}$P NMR (242.94 MHz, DMCO-*d6*), *δ*, ppm: 29.04. HRMS (MALDI-TOF) *m/z* for $C_{29}H_{39}N_4O_5P$: calc. 554.27 $[M]^+$, found 555.30 $[M+H]^+$.

**Diethyl[(2-amino-6-(3-phenylureido)pyridin-3-yl)(3,5-di-*tert*-butyl-4-hydroxyphenyl)methyl]phosphonate 7b**. Similar to the previous one from **3b**. White crystals **7b** in trace amounts were isolated from the mixture. $^1$H NMR (600.13 MHz, DMCO-*d6*), *δ*, ppm: 1.02 (t, 3H, $^3J_{HH}$ = 7.2 Hz, $OCH_2C\underline{H}_3$), 1.09 (t, 3H, $^3J_{HH}$ = 7.1 Hz, $OCH_2C\underline{H}_3$), 1.34 [s, 18H, C($CH_3$)3], 3.84 (m, 1H, $OC\underline{H}_2CH_3$), 3.94 (m, 3H, $OC\underline{H}_2CH_3$), 4.93 (d, 1H, $^2J_{PH}$ = 27.4 Hz, CHP), 6.14 (d, 1H, $^3J_{HH}$ = 8.4 Hz, $CH_{py}$), 6.29 (br.s, 2H, $NH_2$), 6.83 (s, 1H, OH), 6.99 (t, 1H, $^3J_{HH}$ = 7.2 Hz, $NHC_6\underline{H}_5$), 7.23 (t, 1H, $^3J_{HH}$ = 7.4 Hz, $NHC_6\underline{H}_5$), 7.22-7.34 [m, 3H, $NHC_6\underline{H}_5$ and CH̲CC($CH_3$)3], 7.66 (d, 2H, $^3J_{HH}$ = 8.5 Hz, $NHC_6\underline{H}_5$), 7.83 (d, 1H, $^3J_{HH}$ = 8.2 Hz, $CH_{py}$), 8.70 (s, 1H, NH), 11.86 (s, 1H, NH). $^{31}$P NMR (DMCO-*d6*, 242.94 MHz), δ, ppm: 26.64. HRMS (MALDI-TOF) *m/z* for $C_{31}H_{43}N_4O_5P$: calc. 582.30 $[M]^+$, found 583.47 $[M+H]^+$.

**Dimethyl((2,6-bis(3-(*m*-tolyl)ureido)pyridin-3-yl)(3,5-di-*tert*-butyl-4-hydroxyphenyl)methyl)phosphonate 8a.** White solid, yield 75%, M.p.: 201-202°C. IR (KBr), *ν*, $cm^{-1}$: 774; 1027; 1240; 1564, 1655; 1711; 2954; 3367; 3544. $^1$H NMR (DMSO-*d6*, 399.93 MHz), *δ*, ppm: 1.35 [s, 18H, C($CH_3$)3], 2.20 (s, 3H, $CH_3$), 2.28 (s, 3H, $CH_3$), 3.54 (d, 3H, $OCH_3$, $^3J_{PH}$ = 6.9), 3.58 (d, 3H, $OCH_3$, $^3J_{PH}$ = 7.1), 4.92 (d, 1H, CHP, $^2J_{PH}$ 26.7), 6.81 (d, 1H, $NHC_6\underline{H}_4CH_3$, $^3J_{HH}$ = 7.5), 6.85 (d, 1H, $NHC_6\underline{H}_4CH_3$, $^3J_{HH}$ = 7.5), 6.90 (d, 1H, $CH_{py}$, $^3J_{HH}$ = 8.4), 6.93 (s, 1H, OH), 7.11 (t, 1H, $NHC_6\underline{H}_4CH_3$, $^3J_{HH}$ = 7.8), 7.19 (t, 1H, $NHC_6\underline{H}_4CH_3$,$^3J_{HH}$ = 8.0), 7.32 (s, 2H, CH̲CC($CH_3$)3), 7.40 (s, 1H, $NHC_6\underline{H}_4CH_3$), 7.40 (d, 1H, $NHC_6\underline{H}_4CH_3$,$^3J_{HH}$ = 8.0), 7.45 (s, 1H, $NHC_6\underline{H}_4CH_3$), 7.50 (d, 2H, $NHC_6\underline{H}_4CH_3$, $^3J_{HH}$ = 8.1), 8.01 (d, 1H, $CH_{py}$, $^3J_{HH}$ = 8.5), 8.95 (s, 1H, NH), 9.50 (s, 1H, NH), 9.74 (s, 1H, NH), 10.51 (s, 1H, NH). $^{13}C\{^1H\}$ NMR (DMSO-*d6*, 125.76 MHz), *δ*, ppm: 21.55 ($CH_3$), 21.71 ($CH_3$), 30.85 [C(C̲H3)3], 35.11 [C̲(CH3)3], 41.73 (d, CHP, $^1J_{PC}$ = 138.9), 53.44 (d, $POCH_3$, $^2J_{PC}$ = 6.0), 53.74 (d, $POCH_3$, $^2J_{PC}$ = 6.0), 106.20 ($CH_{py}$); 114.63 ($C_{py}$), 116.87 ($NH\underline{C}_6H_4CH_3$), 117.28 ($NH\underline{C}_6H_4CH_3$), 120.18 ($NH\underline{C}_6H_4CH_3$), 120.58 ($NH\underline{C}_6H_4CH_3$), 123.67 ($NH\underline{C}_6H_4CH_3$), 123.73 ($NH\underline{C}_6H_4CH_3$), 126.00 [C̲HCC($CH_3$)2], 126.05 [C̲HCC($CH_3$)2], 126.89 (d, C̲CHP, $^2J_{PC}$ = 3.0), 128.75 ($NH\underline{C}_6H_4CH_3$), 129.02 ($NH\underline{C}_6H_4CH_3$), 138.16

($NH\underline{C}_6H_4CH_3$), 138.37 ($NH\underline{C}_6H_4CH_3$), 139.65 ($NH\underline{C}_6H_4CH_3$), 139.70 ($NH\underline{C}_6H_4CH_3$), 139.72 [$\underline{C}C(CH_3)_3$], 141.29 (d, $CH_{py}$, $^3J_{PC}$ = 3.0), 147.88 (d, $C_{py}$, $^3J_{PC}$ = 10.6), 150.19 ($C_{py}$), 152.24 (C=O), 152.46 (C=O), 153.50 (COH). $^{31}$P NMR (DMSO-$d_6$, 202.46 MHz), $\delta$, ppm: 28.15. Anal. Calcd for $C_{38}H_{48}N_5O_6P$ (%): C, 65.03; H, 6.89; N, 9.98; P, 4.41. Found (%): C, 65.02; H, 6.91; N, 9.99; P, 4.42. HRMS (MALDI-TOF) m/z for $C_{38}H_{48}N_5O_6P$: calc. 701.3 $[M]^+$, found 702.6 $[M+H]^+$; 724.6 $[M+Na]^+$, 740.6 $[M+K]^+$.

**Diethyl((2,6-bis(3-(*m*-tolyl)ureido)pyridin-3-yl)(3,5-di-*tert*-butyl-4-hydroxyphenyl)methyl) phosphonate 8b.** White solid, yield 63%, M.p.: 201-202°C. IR (KBr), $\nu$, cm$^{-1}$: 776; 1025; 1200; 1559, 1597; 1664; 2959; 3309; 3621. $^1$H NMR (DMSO-$d_6$, 399.93 MHz), $\delta$, ppm: 1.04 (t, 3H, $OCH_2C\underline{H}_3$, $^3J_{HH}$ = 7.0), 1.11 (t, 3H, $OCH_2C\underline{H}_3$, $^3J_{HH}$ = 7.0), 1.35 [s, 18H, $C(CH_3)_3$], 2.21 (s, 3H, $NHC_6H_4C\underline{H}_3$), 2.29 (s, 3H, $NHC_6H_4C\underline{H}_3$), 3.80 - 4.00 (m, 4H, $OC\underline{H}_2CH_3$), 4.84 (d, 1H, CHP, $^2J_{PH}$ = 26.9), 6.82 (d, 1H, $NHC_6\underline{H}_4CH_3$,$^3J_{HH}$ = 7.3), 6.86 (d, 2H, $NHC_6\underline{H}_4CH_3$, $^3J_{HH}$ = 7.3), 6.89 (d, 1H, $C_{py}$, $^3J_{HH}$ = 8.5), 6.93 (s, 1H, OH), 7.12 (t, 1H, $NHC_6\underline{H}_4CH_3$, $^3J_{HH}$ = 7.8), 7.19 (t, 1H, $NHC_6\underline{H}_4CH_3$,$^3J_{HH}$ = 7.6), 7.33 (s, 2H, $C\underline{H}CC(CH_3)_3$), 7.40 (s, 1H, $NHC_6\underline{H}_4CH_3$), 7.40 (d, 1H, $NHC_6\underline{H}_4CH_3$,$^3J_{HH}$ = 5.8), 7.46 (s, 1H, $NHC_6\underline{H}_4CH_3$), 7.52 (d, 1H, $NHC_6\underline{H}_4CH_3$, $^3J_{HH}$ = 8.3), 8.02 (dd, 1H, $CH_{py}$, $^3J_{HH}$ 8.3, $^4J_{PH}$ = 1.7), 8.95 (s, 1H, NH), 9.50 (s, 1H, NH), 9.72 (s, 1H, NH), 10.57 (s, 1H, NH). $^{13}$C{$^1$H} NMR (DMSO-$d_6$, 150.92 MHz), $\delta$, ppm: 16.46 (d, $OCH_2\underline{C}H_3$, $^3J_{PC}$ = 4.5), 16.61 (d, $OCH_2\underline{C}H_3$, $^3J_{PC}$ = 4.5), 21.56 ($CH_3$), 21.71 ($CH_3$), 30.84 [$C(\underline{C}H_3)_3$], 35.11 [$\underline{C}(CH_3)_3$], 42.42 (d, CHP, $^1J_{PC}$ = 138.9), 62.45 (d, $O\underline{C}H_2CH_3$, $^2J_{PC}$ = 6.1), 62.84 (d, $O\underline{C}H_2CH_3$, $^2J_{PC}$ = 6.1), 106.03 ($CH_{py}$); 114.74 ($C_{py}$), 116.82 ($NH\underline{C}_6H_4CH_3$), 117.33 ($NH\underline{C}_6H_4CH_3$), 120.13 ($NH\underline{C}_6H_4CH_3$), 120.64 ($NH\underline{C}_6H_4CH_3$), 123.66 ($NH\underline{C}_6H_4CH_3$), 123.70 ($NH\underline{C}_6H_4CH_3$), 126.16 [$\underline{C}HCC(CH_3)_2$], 126.20 [$\underline{C}HCC(CH_3)_2$], 127.04 (d, $\underline{C}CHP$, $^2J_{PC}$ = 3.0), 128.73 ($NH\underline{C}_6H_4CH_3$), 129.02 ($NH\underline{C}_6H_4CH_3$), 138.13 ($NH\underline{C}_6H_4CH_3$), 138.37 ($NH\underline{C}_6H_4CH_3$), 139.64 [$\underline{C}C(CH_3)_3$], 139.68 ($NH\underline{C}_6H_4CH_3$), 139.73 ($NH\underline{C}_6H_4CH_3$), 141.25 ($CH_{py}$), 147.92 (d, $C_{py}$, $^3J_{PC}$ = 10.1), 150.10 ($C_{py}$), 152.17 (C=O), 152.49 (C=O), 153.44 (COH). $^{31}$P NMR (DMSO-$d_6$, 161.90 MHz), $\delta$, ppm: 26.91. Anal. Calcd for $C_{40}H_{52}N_5O_6P$ (%): C, 65.83; H, 7.18; N, 9.60; P, 4.24. Found (%): C, 65.85; H, 7.17; N, 9.61; P, 4.26. HRMS (MALDI-TOF) m/z for $C_{40}H_{52}N_5O_6P$: calc. 729.4 $[M]^+$, found 730.5 $[M+H]^+$, 768.5 $[M+K]^+$.

**Diisopropyl ((2,6-bis(3-(*m*-tolyl)ureido)pyridin-3-yl)(3,5-di-*tert*-butyl-4-hydroxyphenyl)methyl)phosphonate 8c.** White solid, yield 52%, M.p.: 205-206°C. IR (KBr), $\nu$, cm$^{-1}$: 779; 997; 1201; 1555, 1609; 1671; 2960; 3301; 3631. $^1$H NMR (DMSO-$d_6$, 399.93 MHz), $\delta$, ppm: 0.81 (d, 3H, $CH(C\underline{H}_3)_2$, $^3J_{HH}$ = 6.2), 1.00 (d, 3H, $CH(C\underline{H}_3)_2$, $^3J_{HH}$ = 6.2), 1.19 (d, 6H, $CH(C\underline{H}_3)_2$, $^3J_{HH}$ = 6.2), 1.35 [s, 18H, $C(CH_3)_3$], 2.21 (s, 3H, $NHC_6H_4C\underline{H}_3$), 2.29 (s, 3H, $NHC_6H_4C\underline{H}_3$), 4.41 (m, 1H, $C\underline{H}(CH_3)_2$), 4.52 (m, 1H, $C\underline{H}(CH_3)_2$), 4.73 (d, 1H, CHP, $^2J_{PH}$ = 26.9), 6.81 (d, 1H, $CH_{py}$,$^3J_{HH}$ = 7.5), 6.85 (d, 2H, $NHC_6\underline{H}_5CH_3$, $^3J_{HH}$ = 8.0), 6.90 (s, 1H, OH), 6.93 (s, 1H, OH), 7.12 (t, 1H, $NHC_6\underline{H}_4CH_3$, $^3J_{HH}$ = 7.8), 7.19 (t, 1H, $NHC_6\underline{H}_4CH_3$,$^3J_{HH}$ = 8.1), 7.37 (s, 2H, $C\underline{H}CC(CH_3)_3$), 7.39 (s, 1H, $NHC_6\underline{H}_4CH_3$), 7.40 (d, 1H, $NHC_6\underline{H}_4CH_3$,$^3J_{HH}$ = 6.4), 7.47 (s, 1H, $NHC_6\underline{H}_4CH_3$), 7.54 (d, 1H, $NHC_6\underline{H}_4CH_3$, $^3J_{HH}$ = 8.2), 8.07 (d, 1H, $CH_{py}$, $^3J_{HH}$ = 8.2), 8.98 (s, 1H, NH), 9.51 (s, 1H, NH), 9.67 (s, 1H, NH), 10.67 (s, 1H, NH). $^{13}$C{$^1$H} NMR (DMSO-$d_6$, 125.76 MHz), $\delta$, ppm: 21.69 ($CH_3$), 23.23 [d, $OCH(\underline{C}H_3)_2$, $^3J_{PC}$ = 5.0], 23.74 [d, $OCH(\underline{C}H_3)_2$, $^3J_{PC}$ = 5.0], 24.21 [$OCH(\underline{C}H_3)_2$], 24.49 [$OCH(\underline{C}H_3)_2$], 30.79 [$C(\underline{C}H_3)_3$]; 35.08 [$\underline{C}(CH_3)_3$], 42.44 (d, CHP, $^1J_{PC}$ = 142.38), 70.71 (d, $O\underline{C}H_2CH_3$, $^2J_{PC}$ = 7.6), 71.39 (d, $O\underline{C}H_2CH_3$, $^2J_{PC}$ = 7.6), 105.6 ($CH_{py}$); 114.97 ($C_{py}$), 116.68 ($NH\underline{C}_6H_4CH_3$), 117.39 ($NH\underline{C}_6H_4CH_3$), 119.99 ($NH\underline{C}_6H_4CH_3$), 120.71 ($NH\underline{C}_6H_4CH_3$), 123.63 ($NH\underline{C}_6H_4CH_3$), 123.64 ($NH\underline{C}_6H_4CH_3$), 126.26 [$\underline{C}HCC(CH_3)_2$], 126.33 [$\underline{C}HCC(CH_3)_2$], 127.17 (d, $\underline{C}CHP$, $^2J_{PC}$ = 2.5), 128.67 ($NH\underline{C}_6H_4CH_3$), 129.02 ($NH\underline{C}_6H_4CH_3$), 138.07 ($NH\underline{C}_6H_4CH_3$), 138.37 ($NH\underline{C}_6H_4CH_3$), 139.59 [$\underline{C}C(CH_3)_3$], 139.68 ($NH\underline{C}_6H_4CH_3$), 139.71 ($NH\underline{C}_6H_4CH_3$), 140.97 (d, $CH_{py}$, $^3J_{PC}$ = 7.6), 147.78 ($C_{py}$), 149.97 ($C_{py}$), 152.05 (C=O), 152.51 (C=O),

153.39 (COH). $^{31}$P NMR (DMSO-$d_6$, 161.90 MHz), $\delta$, ppm: 24.39. Anal. Calcd for $C_{42}H_{56}N_5O_6P$ (%): C, 66.56; H, 7.45; N, 9.24; P, 4.09. Found (%): C, 66.58; H, 7.47; N, 9.26; P, 4.10. HRMS (MALDI-TOF) m/z for $C_{42}H_{56}N_5O_6P$: calc. 757.4 $[M]^+$, found 758.7 $[M+H]^+$; 780.8 $[M+Na]^+$, 796.7 $[M+K]^+$.

**Diphenyl((2,6-bis(3-(*m*-tolyl)ureido)pyridin-3-yl)(3,5-di-*tert*-butyl-4-hydroxyphenyl)methyl)phosphonate 8d.** White solid, yield 60%, M.p.: 208-209°C. IR (KBr), $\nu$, cm$^{-1}$: 771; 1161; 1207; 1558, 1595; 1664; 2924; 3351; 3624. $^1$H NMR (DMSO-$d_6$, 399.93 MHz), $\delta$, ppm: 1.32 [s, 18H, $C(CH_3)_3$], 2.22 (s, 3H, $CH_3$), 2.29 (s, 3H, $CH_3$), 5.44 (d, 1H, CHP, $^2J_{PH}$ = 28.0), 6.79 (d, 1H, $CH_{py}$, $^3J_{HH}$ = 8.4), 6.82 (d, 1H, $NHC_6\underline{H}_4CH_3$,$^3J_{HH}$ = 7.5), 6.84 (d, 1H, $NHC_6\underline{H}_4CH_3$,$^3J_{HH}$ = 7.5), 6.68 (d, 2H, $OC_6H_5$, $^3J_{HH}$ = 8.0), 7.01 (s, 1H, OH), 7.01 (t, 2H, $OC_6H_5$, $^3J_{HH}$ = 7.6), 7.10-7.30 (m, 6H, $OC_6H_5$, $NHC_6\underline{H}_4CH_3$), 7.32 (t, 2H, $OC_6H_5$, $^3J_{HH}$ = 7.8), 7.34 (s, 1H, $NHC_6\underline{H}_4CH_3$), 7.41 (s, 1H, $NHC_6\underline{H}_4CH_3$), 7.43 (s, 2H, $C\underline{H}CC(CH_3)_3$), 7.46 (d, 1H, $NHC_6\underline{H}_4CH_3$,$^3J_{HH}$ = 8.0), 7.52 (d, 1H, $NHC_6\underline{H}_4CH_3$,$^3J_{HH}$ = 8.0), 8.17 (d, 1H, $CH_{py}$, $^3J_{HH}$ = 8.5), 9.09 (s, 1H, NH), 9.56 (s, 1H, NH), 9.74 (s, 1H, NH), 10.52 (s, 1H, NH). $^{13}$C{$^1$H} NMR (DMSO-$d_6$, 100.57 MHz), $\delta$, ppm: 22.07 ($CH_3$), 22.22 ($CH_3$), 31.22 [$C(\underline{C}H_3)_3$], 35.61 [$\underline{C}(CH_3)_3$], 43.35 (d, CHP, $^1J_{PC}$ = 139.4), 106.85 ($CH_{py}$); 114.10 ($C_{py}$), 117.43 ($NH\underline{C}_6H_4CH_3$), 117.82 ($NH\underline{C}_6H_4CH_3$), 120.74 ($NH\underline{C}_6H_4CH_3$), 121.14 ($NH\underline{C}_6H_4CH_3$), 121.14 (d, $OC_6H_5$, $^3J_{PC}$ = 3.0), 121.31 (d, $OC_6H_5$, $^3J_{PC}$ = 3.0), 124.24 ($NH\underline{C}_6H_4CH_3$), 124.31 ($NH\underline{C}_6H_5CH_3$), 126.00 (d, $\underline{C}$CHP, $^2J_{PC}$ = 5.1), 126.02 ($OC_6H_5$), 126.26 ($OC_6H_5$), 127.04 [$\underline{C}HCC(CH_3)_3$], 127.11 [$\underline{C}HCC(CH_3)_3$], 129.54 ($NH\underline{C}_6H_4CH_3$), 129.59 ($NH\underline{C}_6H_4CH_3$), 129.10 ($NH\underline{C}_6H_4CH_3$), 130.51 ($OC_6H_5$), 130.82 ($OC_6H_5$), 138.69 ($NH\underline{C}_6H_4CH_3$), 138.91 ($NH\underline{C}_6H_4CH_3$), 140.10 ($NH\underline{C}_6H_4CH_3$), 140.18 ($NH\underline{C}_6H_4CH_3$), 140.47 [$\underline{C}C(CH_3)_3$], 141.60 (d, $CH_{py}$, $^3J_{PC}$ = 3.0), 147.62 (d, $C_{py}$, $^3J_{PC}$ = 12.12), 150.96 (d, $OC_6H_5$, $^2J_{PC}$ = 11.1), 151.01 ($C_{py}$), 151.22 (d, $OC_6H_5$, $^2J_{PC}$ = 10.1), 152.81 (C=O), 152.96 (C=O), 154.49 (COH). $^{31}$P NMR (DMSO-$d_6$, 161.90 MHz), $\delta$, ppm: 19.29. Anal. Calcd for $C_{48}H_{52}N_5O_6P$ (%): C, 69.80; H, 6.35; N, 8.48; P, 3.75. Found (%): C, 69.82; H, 6.37; N, 8.49; P, 3.76. HRMS (MALDI-TOF) m/z for $C_{48}H_{52}N_5O_6P$: calc. 825.4 $[M]^+$, found 826.3 $[M+H]^+$; 848.2 $[M+Na]^+$, 864.2 $[M+K]^+$.

**Dimethyl((2,6-bis(3-(*p*-tolyl)ureido)pyridin-3-yl)(3,5-di-*tert*-butyl-4-hydroxyphenyl)methyl)phosphonate 9a.** White solid, yield 60%, M.p.: 217-218°C. IR (KBr), $\nu$, cm$^{-1}$: 795; 1028; 1055;1239; 1595, 1656; 1706; 3363; 3539. $^1$H NMR (DMSO-$d_6$, 399.93 MHz), $\delta$, ppm: 1.34 [s, 18H, $C(CH_3)_3$], 2.25 (s, 3H, $CH_3$), 2.28 (s, 3H, $CH_3$), 3.54 (d, 3H, $OCH_3$, $^3J_{PH}$ = 10.8), 3.57 (d, 3H, $OCH_3$, $^3J_{PH}$ = 7.2), 3.72 (s, 3H, $OCH_3$), 4.92 (d, 1H, CHP, $^2J_{PH}$ = 26.9), 6.92 (d, 1H, $CH_{py}$, $^3J_{HH}$ = 8.6), 6.94 (s, 1H, OH), 7.05 (d, 2H, $NHC_6\underline{H}_4CH_3$, $^3J_{HH}$ = 8.1), 7.13 (d, 2H, $NHC_6\underline{H}_4CH_3$, $^3J_{HH}$ = 8.0), 7.31 (s, 2H, $C\underline{H}CC(CH_3)_3$), 7.47 (d, 2H, $NHC_6\underline{H}_4CH_3$, $^3J_{HH}$ = 7.9), 7.55 (d, 2H, $NHC_6\underline{H}_4CH_3$, $^3J_{HH}$ = 8.0), 8.01 (d, 1H, $CH_{py}$, $^3J_{HH}$ = 8.6), 8.93 (s, 1H, NH), 9.44 (s, 1H, NH), 9.78 (s, 1H, NH), 10.39 (s, 1H, NH). $^{13}$C{$^1$H} NMR (DMSO-$d_6$, 150.92 MHz), $\delta$, ppm: 20.87 ($CH_3$), 30.86 [$C(\underline{C}H_3)_3$], 35.12 [$\underline{C}(CH_3)_3$], 41.73 (d, CHP, $^1J_{PC}$ = 139.9), 53.42 (d, $POCH_3$, $^2J_{PC}$ = 6.0), 53.74 (d, $POCH_3$, $^2J_{PC}$ = 6.0), 106.06 ($CH_{py}$); 114.47 ($C_{py}$), 119.83 ($NH\underline{C}_6H_4CH_3$), 120.08 ($NH\underline{C}_6H_4CH_3$), 126.03 [$\underline{C}HCC(CH_3)_2$], 126.06 [$\underline{C}HCC(CH_3)_2$], 126.89 ($\underline{C}$CHP), 129.37 ($NH\underline{C}_6H_4CH_3$), 129.60 ($NH\underline{C}_6H_4CH_3$), 131.79 ($NH\underline{C}_6H_4CH_3$), 131.96 ($NH\underline{C}_6H_4CH_3$), 137.12 ($NH\underline{C}_6H_4CH_3$), 137.23 ($NH\underline{C}_6H_4CH_3$), 139.71 [$\underline{C}C(CH_3)_3$], 141.30 ($CH_{py}$), 148.02 (d, $C_{py}$, $^3J_{PC}$ = 10.6), 150.18 ($C_{py}$), 152.32 (C=O), 152.49 (C=O), 153.51 (COH). $^{31}$P NMR (DMSO-$d_6$, 161.90 MHz), $\delta$, ppm: 28.59. Anal. Calcd for $C_{38}H_{48}N_5O_6P$ (%): C, 65.03; H, 6.89; N, 9.98; P, 4.41. Found (%): C, 65.04; H, 6.90; N, 10.00; P, 4.43. HRMS (MALDI-TOF) m/z for $C_{38}H_{48}N_5O_6P$: calc. 701.3 $[M]^+$, found: 702.6 $[M+H]^+$; 724.6 $[M+Na]^+$, 740.7 $[M+K]^+$.

**Diethyl((2,6-bis(3-(*p*-tolyl)ureido)pyridin-3-yl)(3,5-di-*tert*-butyl-4-hydroxyphenyl)methyl)phosphonate 9b.** White solid, yield 71%, M.p.: 229-230°C. IR (KBr), $\nu$, cm$^{-1}$: 815; 1100; 1224;1548; 1603, 1642; 2960; 3325; 3620. $^1$H NMR (DMSO-$d_6$, 600.13 MHz), $\delta$, ppm: 1.03 (t, 3H, $OCH_2C\underline{H}_3$, $^3J_{HH}$ = 7.0), 1.10 (t, 3H, $OCH_2C\underline{H}_3$, $^3J_{HH}$ = 7.0), 1.35 [s, 18H, $CH(C\underline{H}_3)_2$], 2.25

(s, 3H, $CH_3$), 2.28 (s, 3H, $CH_3$), 3.83 (m, 1H, $OC\underline{H}_2CH_3$), 3.94 (m, 3H, $OC\underline{H}_2CH_3$), 4.84 (d, 1H, CHP, $^2J_{PH}$ = 26.7), 6.90 (s, 1H, OH), 6.91 (d, 1H, $CH_{py}$, $^3J_{HH}$ = 6.0), 7.05 (d, 2H, $NHC_6\underline{H}_4CH_3$, $^3J_{HH}$ = 8.0), 7.13 (d, 2H, $NHC_6\underline{H}_4CH_3$, $^3J_{HH}$ = 8.0), 7.32 (s, 2H, $C\underline{H}CC(CH_3)_3$, 7.47 (d, 2H, $NHC_6\underline{H}_4CH_3$, $^3J_{HH}$ = 8.0), 7.55 (d, 2H, $NHC_6\underline{H}_4CH_3$, $^3J_{HH}$ = 8.1), 8.00 (d, 1H, $C\underline{H}_{py}$, $^3J_{HH}$ = 8.5), 8.92 (s, 1H, $N\underline{H}$), 9.43 (s, 1H, $N\underline{H}$), 9.75 (s, 1H, $N\underline{H}$), 10.43 (s, 1H, $N\underline{H}$). ). $^{13}C\{^1H\}$ NMR (DMSO-$d_6$, 150.92 MHz), $\delta$, ppm: 16.46 (d, $OCH_2\underline{C}H_3$, $^3J_{PC}$ = 4.6), 16.61 (d, $OCH_2\underline{C}H_3$, $^3J_{PC}$ = 4.6), 20.87 ($CH_3$), 30.84 [$C(\underline{C}H_3)_3$], 35.10 [$\underline{C}(CH_3)_3$], 42.38 (d, CHP, $^1J_{PC}$ = 138.9), 62.43 (d, $O\underline{C}H_2CH_3$, $^2J_{PC}$ = 6.1), 62.82 (d, $O\underline{C}H_2CH_3$, $^2J_{PC}$ = 6.1), 105.90 ($CH_{py}$); 114.61 ($C_{py}$), 119.75 ($NH\underline{C}_6H_4CH_3$), 120.10 ($NH\underline{C}_6H_4CH_3$), 126.15 [$\underline{C}HCC(CH_3)_2$], 126.20 [$\underline{C}HCC(CH_3)_2$], 127.04 (d, $\underline{C}CHP$, $^2J_{PC}$ = 3.0), 129.35 ($NH\underline{C}_6H_4CH_3$), 129.61 ($NH\underline{C}_6H_4CH_3$), 131.78 ($NH\underline{C}_6H_4CH_3$), 131.93 ($NH\underline{C}_6H_4CH_3$), 137.11 ($NH\underline{C}_6H_4CH_3$), 137.23 ($NH\underline{C}_6H_4CH_3$), 139.63 [$\underline{C}C(CH_3)_3$], 141.24 ($CH_{py}$), 148.01 (d, $C_{py}$, $^3J_{PC}$ = 10.1), 150.07 ($C_{py}$), 152.23 (C=O), 152.49 (C=O), 153.42 (COH). $^{31}P$ NMR (DMSO-$d_6$, 242.94 MHz), $\delta$, ppm: 25.93. Anal. Calcd for $C_{40}H_{52}N_5O_6P$ (%): C, 65.83; H, 7.18; N, 9.60; P, 4.24. Found (%): C, 65.84; H, 7.19; N, 9.62; P, 4.25. HRMS (MALDI-TOF) m/z for $C_{40}H_{52}N_5O_6P$: calc. 729.4 $[M]^+$, found: 730.8 $[M+H]^+$; 752.8 $[M+Na]^+$, 768.8 $[M+K]^+$.

**Diisopropyl((2,6-bis(3-(*p*-tolyl)ureido)pyridin-3-yl)(3,5-di-*tert*-butyl-4-hydroxyphenyl)methyl)phosphonate 9c.** White solid, yield 55%, M.p.: 201-202°C. IR (KBr), $\nu$, $cm^{-1}$: 815; 1100; 1236; 1599; 1671, 1726; 2974; 3307; 3633. $^1H$ NMR (DMSO-$d_6$, 600.13 MHz), $\delta$, ppm: 0.82 [d, 3H, $OCH(\underline{CH_3})_2$,$^3J_{HH}$ = 12], 1.01 [d, 3H, $CH(\underline{CH_3})_2$, $^3J_{HH}$ = 6], 1.19 [d, 6H, $OCH(\underline{CH_3})_2$, $^3J_{HH}$ = 12], 1.35 [s, 18H, $C(CH_3)_3$], 2.26 (d, 3H, $C_6H_4\underline{CH_3}$), 2.29 (d, 3H, $C_6H_4\underline{CH_3}$), 4.42 [m, 1H, $O\underline{CH}(CH_3)_2$], 4.52 [m, 1H, $O\underline{CH}(CH_3)_2$], 4.74 (d, 1H, CHP, $^2J_{PH}$ = 27.0), 6.88 (s, 1H, OH), 6.89 (d, 2H, $CH_{py}$, $^3J_{HH}$ = 9.0), 7.06 (d, 2H, $NHC_6\underline{H}_4CH_3$, $^3J_{HH}$ = 8.1), 7.15 (d, 2H, $NHC_6\underline{H}_4CH_3$, $^3J_{HH}$ = 8.1), 7.37 (s, 2H, $C\underline{H}CC(CH_3)_3$), 7.48 (d, 2H, $NHC_6\underline{H}_4CH_3$, $^3J_{HH}$ = 12), 7.58 (d, 2H, $NHC_6\underline{H}_4CH_3$, $^3J_{HH}$ = 8.1), 8.06 (d, 1H, $CH_{py}$, $^3J_{HH}$ = 12), 8.96 (s, 1H, NH), 9.43 (s, 1H, NH), 9.72 (s, 1H, NH), 10.53 (s, 1H, NH). $^{13}C\{^1H\}$ NMR (DMSO-$d_6$, 150.92 MHz), $\delta$, ppm: 20.87 ($CH_3$), 23.27 [d, $OCH(\underline{C}H_3)_2$, $^3J_{PC}$ = 4.5], 23.76 [d, $OCH(\underline{C}H_3)_2$, $^3J_{PC}$ = 4.5], 24.24 [$OCH(\underline{C}H_3)_2$], 24.50 [$OCH(\underline{C}H_3)_2$], 30.82 [$C(\underline{C}H_3)_3$]; 35.10 [$\underline{C}(CH_3)_3$], 42.03 (d, CHP, $^1J_{PC}$ = 140.4), 70.74 (d, $O\underline{C}H(CH_3)_2$, $^2J_{PC}$ = 7.6), 71.40 (d, $O\underline{C}H(CH_3)_2$, $^2J_{PC}$ = 7.6), 105.75 ($CH_{py}$); 114.88 ($C_{py}$), 119.66 ($NH\underline{C}_6H_4CH_3$), 120.19 ($NH\underline{C}_6H_4CH_3$), 126.32 [$\underline{C}HCC(CH_3)_2$], 126.36 [$\underline{C}HCC(CH_3)_2$], 127.22 ($\underline{C}CHP$), 129.32 ($NH\underline{C}_6H_4CH_3$), 129.63 ($NH\underline{C}_6H_4CH_3$), 137.16 ($NH\underline{C}_6H_4CH_3$), 137.26 ($NH\underline{C}_6H_4CH_3$), 139.60 [$\underline{C}C(CH_3)_3$], 140.99 ($CH_{py}$), 147.98 (d, $C_{py}$, $^3J_{PC}$ = 9.1), 150.02 ($C_{py}$), 152.16 (C=O), 152.54 (C=O), 153.40 (COH). $^{31}P$ NMR (DMSO-$d_6$, 242.94 MHz), $\delta$, ppm: 25.49. Anal. Calcd for $C_{42}H_{56}N_5O_6P$ (%): C, 66.56; H, 7.45; N, 9.24; P, 4.09. Found (%): C, 66.57; H, 7.47; N, 9.27; P, 4.11. HRMS (MALDI-TOF) m/z for $C_{42}H_{56}N_5O_6P$: calc. 757.4 $[M]^+$, found: 758.7 $[M+H]^+$; 780.6 $[M+Na]^+$, 802.6 $[M+K]^+$.

**Diphenyl((2,6-bis(3-(*p*-tolyl)ureido)pyridin-3-yl)(3,5-di-*tert*-butyl-4-hydroxyphenyl)methyl)phosphonate 9d.** White solid, yield 68%, M.p.: 208-209°C. IR (KBr), $\nu$, $cm^{-1}$:762; 1163; 1207; 1548; 1596, 1727; 2956; 3301; 3631. $^1H$ NMR (DMSO-$d_6$, 399.93 MHz), $\delta$, ppm: 1.32 [s, 18H, $C(CH_3)_3$], 2.26 (s, 3H, $CH_3$), 2.29 (s, 3H, $CH_3$), 5.44 (d, 1H, CHP, $^2J_{PH}$ = 28.2), 6.68 (d, 2H, $NHC_6\underline{H}_4OCH_3$, $^3J_{HH}$ = 8.0), 7.00 (m, 4H, $NHC_6\underline{H}_4OCH_3$, $CH_{py}$, OH), 7.05-7.15 (m, 6H, $OC_6H_5$), 7.22 (t, 2H, $OC_6H_5$, $^3J_{HH}$ = 7.8), 7.32 (t, 2H, $OC_6H_5$, $^3J_{HH}$ = 7.8), 7.42 (s, 2H, $C\underline{H}CC(CH_3)_3$), 7.48 (d, 2H, $NHC_6\underline{H}_4CH_3$, $^3J_{HH}$ = 7.4), 7.56 (d, 2H, $NHC_6\underline{H}_4CH_3$, $^3J_{HH}$ = 8.2), 8.16 (d, 1H, $CH_{py}$, $^3J_{HH}$ 8.0), 9.05 (s, 1H, NH), 9.49 (s, 1H, NH), 9.77 (s, 1H, NH), 10.39 (s, 1H, NH). $^{13}C\{^1H\}$ NMR (DMSO-$d_6$, 100.57 MHz), $\delta$, ppm: 21.32 ($CH_3$), 31.21 [$C(\underline{C}H_3)_3$]; 35.60 [$\underline{C}(CH_3)_3$], 43.29 (d, CHP, $^1J_{PC}$ = 140.8), 106.70 ($CH_{py}$); 113.90 ($C_{py}$), 120.34 ($NH\underline{C}_6H_4CH_3$), 120.57 ($NH\underline{C}_6H_4CH_3$), 121.10 (d, $OC_6H_5$, $^3J_{PC}$ = 4.0), 121.28 (d, $OC_6H_5$, $^3J_{PC}$ = 3.0), 126.01 [$\underline{C}HCC(CH_3)_2$], 126.01 ($\underline{C}CHP$), 126.23 [$\underline{C}HCC(CH_3)_2$], 129.88

($NH\underline{C}_6H_4CH_3$), 130.50 ($NH\underline{C}_6H_4CH_3$), 130.80 ($OC_6H_5$), 131.49 ($OC_6H_5$), 132.32 ($NH\underline{C}_6H_4CH_3$), 132.51 ($NH\underline{C}_6H_4CH_3$), 137.54 ($NH\underline{C}_6H_4CH_3$), 137.68 ($NH\underline{C}_6H_4CH_3$), 140.43 [$\underline{C}C(CH_3)_3$], 141.57 (d, $C_{py}$, $^3J_{PC}$ = 5.1), 148.71 (d, $C_{py}$, $^3J_{PC}$ = 9.1), 150.92 (d, $OC_6H_5$, $^2J_{PC}$ = 10.1), 150.97 ($C_{py}$), 151.20 (d, $OC_6H_5$, $^2J_{PC}$ = 9.1), 152.83 (C=O), 152.93 (C=O), 153.62 (COH). $^{31}P$ NMR (DMSO-$d_6$, 161.90 MHz), *δ*, ppm: 19.27. Anal. Calcd for $C_{48}H_{52}N_5O_6P$ (%): C, 69.80; H, 6.35; N, 8.48; P, 3.75. Found (%): C, 69.81; H, 6.36; N, 8.50; P, 3.77. HRMS (MALDI-TOF) m/z for $C_{48}H_{52}N_5O_6P$: calc. 825.4 $[M]^+$, found: 826.9 $[M+H]^+$, 848.9 $[M+Na]^+$, 864.9 $[M+K]^+$.

**Dimethyl((2,6-bis-(3-(4-methoxyphenyl)ureido)pyridin-3-yl)(3,5-di-*tert*-butyl-4-hydroxyphenyl)methyl)phosphonate 10a.** White solid, yield 51%, M.p.: 214-215°C. IR (KBr), *ν*, $cm^{-1}$:830; 1026; 1040; 1055; 1238; 1559, 1598; 1707; 2957; 3374; 3575. $^1H$ NMR (DMSO-$d_6$, 600.13 MHz), *δ*, ppm: 1.35 [s, 18H, $C(CH_3)_3$], 3.54 (d, 3H, $OCH_3$, $^3J_{PH}$ = 10.8), 3.58 (d, 3H, $OCH_3$, $^3J_{PH}$ = 10.8), 3.72 (s, 3H, $OCH_3$), 3.74 (s, 3H, $OCH_3$), 4.93 (d, 1H, CHP, $^2J_{PH}$ = 26.8), 6.82 (d, 2H, $NHC_6\underline{H}_4OCH_3$, $^3J_{HH}$ = 9.0), 6.92 (m, 4H, $NHC_6\underline{H}_4OCH_3$, OH, $CH_{py}$), 7.32 (s, 2H, $C\underline{H}CC(CH_3)_3$), 7.48 (d, 2H, $NHC_6\underline{H}_4OCH_3$, $^3J_{HH}$ = 9.0), 7.54 (d, 2H, $NHC_6\underline{H}_4OCH_3$, $^3J_{HH}$ = 9.1), 8.00 (d, 1H, $CH_{py}$, $^3J_{HH}$ = 8.5), 8.91 (s, 1H, NH), 9.40 (s, 1H, NH), 9.78 (s, 1H, NH), 10.31 (s, 1H, NH). $^{13}C\{^1H\}$ NMR (DMSO-$d_6$, 125.76 MHz), *δ*, ppm: 30.83 [$C(\underline{C}H_3)_3$], 35.09 [$\underline{C}(CH_3)_3$], 41.62 (d, CHP, $^1J_{PC}$ = 139.9), 53.41 (d, $POCH_3$, $^2J_{PC}$ = 6.3), 53.72 (d, $POCH_3$, $^2J_{PC}$ = 7.6), 55.62 ($OCH_3$), 55.69 ($OCH_3$), 105.80 ($CH_{py}$); 114.15 ($NH\underline{C}_6H_4OCH_3$), 114.25 (d, $C_{py}$, $^2J_{PC}$ = 2.5), 114.39 ($NH\underline{C}_6H_4OCH_3$), 121.58 ($NH\underline{C}_6H_4OCH_3$), 121.63 ($NH\underline{C}_6H_4OCH_3$), 125.99 [($\underline{C}HCC(CH_3)_2$], 126.04 [($\underline{C}HCC(CH_3)_2$], 126.88 (d, $\underline{C}CHP$, $^2J_{PC}$ = 3.8), 132.63 ($NH\underline{C}_6H_4OCH_3$), 132.79 ($NH\underline{C}_6H_4OCH_3$), 139.67 [$\underline{C}C(CH_3)_3$], 141.28 (d, $CH_{py}$, $^3J_{PC}$ = 5.0), 148.02 (d, $C_{py}$, $^3J_{PC}$ = 12.6), 150.16 ($C_{py}$), 152.47 (C=O), 152.55 (C=O), 153.46 (COH), 155.31 ($NH\underline{C}_6H_4OCH_3$), 155.48 ($NH\underline{C}_6H_4OCH_3$). $^{31}P$ NMR (DMSO-$d_6$, 242.94 MHz), *δ*, ppm: 28.63. Anal. Calcd for $C_{38}H_{48}N_5O_8P$ (%): C, 62.20; H, 6.59; N, 9.54; P, 4.22. Found (%): C, 62.21; H, 6.61; N, 9.55; P, 4.25. HRMS (MALDI-TOF) m/z for $C_{38}H_{48}N_5O_8P$: calc. 733.3 $[M]^+$, found: 756.7 $[M+Na]^+$, 772.7 $[M+K]^+$.

**Diethyl((2,6-bis-(3-(4-methoxyphenyl)ureido)pyridin-3-yl)(3,5-di-*tert*-butyl-4-hydroxyphenyl)methyl)phosphonate 10b.** White solid, yield 46%, M.p.: 214-215°C. IR (KBr), *ν*, $cm^{-1}$:829; 1021; 1050; 1228; 1603; 1667, 1721; 2958; 3306; 3598. $^1H$ NMR (DMSO-$d_6$, 600.13 MHz), *δ*, ppm: 1.04 (t, 3H, $OCH_2C\underline{H}_3$, $^3J_{HH}$ = 7.2), 1.10 (t, 3H, $OCH_2C\underline{H}_3$, $^3J_{HH}$ = 7.2), 1.35 [s, 18H, $CH(C\underline{H}_3)_2$], 3.72 (s, 3H, $OCH_3$), 3.74 (s, 3H, $OCH_3$), 3.83 (m, 1H, $OC\underline{H}_2CH_3$), 3.94 (m, 3H, $OC\underline{H}_2CH_3$), 4.85 (d, 1H, CHP, $^2J_{PH}$ = 26.9), 6.82 (d, 2H, $NHC_6\underline{H}_4OCH_3$, $^3J_{HH}$ = 9.3), 6.89 (d, 1H, $CH_{py}$, $^3J_{HH}$ = 6), 6.91 (m, 3H, $NHC_6\underline{H}_4OCH_3$, OH), 7.32 (s, 2H, $C\underline{H}CC(CH_3)_3$), 7.47 (d, 2H, $NHC_6\underline{H}_4OCH_3$, $^3J_{HH}$ = 9.1), 7.55 (d, 2H, $NHC_6\underline{H}_4OCH_3$, $^3J_{HH}$ = 9.3), 7.99 (d, 1H, $C\underline{H}_{py}$, $^3J_{HH}$ = 8.5), 8.90 (s, 1H, $N\underline{H}$), 9.40 (s, 1H, $N\underline{H}$), 9.74 (s, 1H, $N\underline{H}$), 10.36 (s, 1H, $N\underline{H}$). $^{13}C\{^1H\}$ NMR (DMSO-$d_6$, 125.76 MHz), *δ*, ppm: 16.43 (d, $OCH_2\underline{C}H_3$, $^3J_{PC}$ = 6.1), 16.57 (d, $OCH_2\underline{C}H_3$, $^3J_{PC}$ = 6.1), 30.81 [$C(\underline{C}H_3)_3$], 35.08 [$\underline{C}(CH_3)_3$], 42.34 (d, CHP, $^1J_{PC}$ = 139.4), 55.61 ($OCH_3$), 55.68 ($OCH_3$), 62.41 (d, $O\underline{C}H_2CH_3$, $^2J_{PC}$ = 6.1), 62.79 (d, $O\underline{C}H_2CH_3$, $^2J_{PC}$ = 7.1), 105.64 ($CH_{py}$); 114.12 ($NH\underline{C}_6H_4OCH_3$), 114.22 (d, $C_{py}$, $^2J_{PC}$ = 3.0), 114.39 ($NH\underline{C}_6H_4OCH_3$), 121.51 ($NH\underline{C}_6H_4OCH_3$), 121.66 ($NH\underline{C}_6H_4OCH_3$), 126.13 [$\underline{C}HCC(CH_3)_2$], 126.20 [$\underline{C}HCC(CH_3)_2$], 127.02 (d, $\underline{C}CHP$, $^2J_{PC}$ = 4.0), 132.65 ($NH\underline{C}_6H_4OCH_3$), 132.81 ($NH\underline{C}_6H_4OCH_3$), 139.58 [$\underline{C}C(CH_3)_3$], 141.23 (d, $CH_{py}$, $^3J_{PC}$ = 5.1), 148.10 (d, $C_{py}$, $^3J_{PC}$ = 10.1), 150.07 ($C_{py}$), 152.40 (C=O), 152.56 (C=O), 153.40 (COH), 155.30 ($NH\underline{C}_6H_4OCH_3$), 155.46 ($NH\underline{C}_6H_4OCH_3$). $^{31}P$ NMR (DMSO-$d_6$, 242.94 MHz), *δ*, ppm: 26.38. Anal. Calcd for $C_{40}H_{52}N_5O_8P$ (%): C, 63.06; H, 6.88; N, 9.19; P, 4.07. Found (%): C, 63.10; H, 6.92; N, 9.21; P, 4.10. HRMS (MALDI-TOF) m/z for $C_{40}H_{52}N_5O_8P$: calc. 761.4 $[M]^+$, found: 762.7 $[M+H]^+$, 784.7 $[M+Na]^+$, 800.8 $[M+K]^+$.

**Diisopropyl((2,6-bis-(3-(4-methoxyphenyl)ureido)pyridin-3-yl)(3,5-di-*tert*-butyl-4-hydroxyphenyl)methyl)phosphonate 10c.** White solid, yield 51%, M.p.: 201-202°C. IR (KBr), *ν*,

cm$^{-1}$:828; 1004; 1243; 1602; 1670, 1720; 2957; 3200; 3412. $^1$H NMR (DMSO-$d_6$, 600.13 MHz), $\delta$, ppm: 0.82 [d, 3H, $OCH(\underline{CH}_3)_2$,$^3J_{HH}$ = 6.2], 1.00 [d, 3H, $CH(\underline{CH}_3)_2$, $^3J_{HH}$ = 6.1], 1.19 [m, 6H, $OCH(\underline{CH}_3)_2$], 1.35 [s, 18H, $C(CH_3)_3$], 3.72 (s, 3H, $OCH_3$), 3.75 (s, 3H, $OCH_3$), 4.41 [m, 1H, $O\underline{CH}(CH_3)_2$], 4.51 [m, 1H, $O\underline{CH}(CH_3)_2$], 4.73 (d, 1H, CHP, $^2J_{PH}$ = 27.0), 6.82 (d, 2H, $NHC_6\underline{H}_4OCH_3$, $^3J_{HH}$ = 9.0), 6.86 (m, 1H, $CH_{py}$, $^3J_{HH}$ = 6), 6.91 (m, 3H, $NHC_6\underline{H}_4OCH_3$, OH), 7.36 (s, 2H, $C\underline{H}CC(CH_3)_3$), 7.47 (d, 2H, $NHC_6\underline{H}_4OCH_3$, $^3J_{HH}$ = 8.7), 7.57 (d, 2H, $NHC_6\underline{H}_4OCH_3$, $^3J_{HH}$ = 9.3), 8.03 (d, 1H, $CH_{py}$, $^3J_{HH}$ = 8.4), 8.94 (s, 1H, NH), 9.41 (s, 1H, NH), 9.68 (s, 1H, NH), 10.48 (s, 1H, NH). $^{13}C\{^1H\}$ NMR (DMSO-$d_6$, 150.92 MHz), $\delta$, ppm: 23.29 [d, $OCH(\underline{C}H_3)_2$, $^3J_{PC}$ = 4.5], 23.77 [d, $OCH(\underline{C}H_3)_2$, $^3J_{PC}$ = 4.5], 24.23 [$OCH(\underline{C}H_3)_2$], 24.49 [$OCH(\underline{C}H_3)_2$], 30.83 [$C(\underline{C}H_3)_3$]; 35.10 [$\underline{C}(CH_3)_3$], 42.02 (d, CHP, $^1J_{PC}$ = 138.9), 55.65 ($OCH_3$), 55.73 ($OCH_3$), 70.75 (d, $O\underline{C}H(CH_3)_2$, $^2J_{PC}$ = 6.1), 71.38 (d, $O\underline{C}H(CH_3)_2$, $^2J_{PC}$ = 6.1), 105.54 ($CH_{py}$); 114.13 ($NH\underline{C}_6H_4OCH_3$), 114.45 ($NH\underline{C}_6H_4OCH_3$), 114.57 (d, $C_{py}$, $^2J_{PC}$ = 3.0), 121.44 ($NH\underline{C}_6H_4OCH_3$), 121.75 ($NH\underline{C}_6H_4OCH_3$), 126.33 [$\underline{C}HCC(CH_3)_2$], 126.38 [$\underline{C}HCC(CH_3)_2$], 127.24 ($\underline{C}CHP$), 132.73 ($NH\underline{C}_6H_4OCH_3$), 132.87 ($NH\underline{C}_6H_4OCH_3$), 139.58 [$\underline{C}C(CH_3)_3$], 141.03 (d, $CH_{py}$, $^3J_{PC}$ = 5.1), 148.08 (d, $C_{py}$, $^3J_{PC}$ = 9.1), 150.04 ($C_{py}$), 152.35 (C=O), 152.63 (C=O), 153.39 (COH), 155.33 ($NH\underline{C}_6H_4OCH_3$), 155.47 ($NH\underline{C}_6H_4OCH_3$). $^{31}$P NMR (DMSO-$d_6$, 242.94 MHz), $\delta$, ppm: 25.53. Anal. Calcd for $C_{48}H_{52}N_5O_6P$ (%): C, 63.86; H, 7.15; N, 8.87; P, 3.92. Found (%): C, 63.90; H, 7.18; N, 8.90; P, 3.93. HRMS (MALDI-TOF) m/z for $C_{42}H_{56}N_5O_8P$: calc. 789.4 [M]$^+$, found: 790.6 [M+H]$^+$, 812.6 [M+Na]$^+$, 829.6 [M+K]$^+$.

**Diphenyl((2,6-bis-(3-(4-methoxyphenyl)ureido)pyridin-3-yl)(3,5-di-*tert*-butyl-4-hydroxyphenyl)methyl)phosphonate 10d.** White solid, yield 66%, M.p.: 201-202°C. IR (KBr), $\nu$, cm$^{-1}$:827; 1026; 1244; 1600; 1662, 1720; 2955; 3078; 3367. $^1$H NMR (DMSO-$d_6$, 600.13 MHz), $\delta$, ppm: 1.32 [s, 18H, $C(CH_3)_3$], 3.72 (s, 3H, $OCH_3$), 3.75 (s, 3H, $OCH_3$), 5.45 (d, 1H, CHP, $^2J_{PH}$ = 28.0), 6.68 (d, 2H, $NHC_6H_4OCH_3$, $^3J_{HH}$ = 8.0), 6.83 (d, 2H, $NHC_6\underline{H}_4OCH_3$, $^3J_{HH}$ = 8.0), 6.85 (d, 1H, $CH_{py}$, $^3J_{HH}$ = 8.8), 6.92 (d, 2H, $OC_6H_5$, $^3J_{HH}$ = 9.0), 6.98 (s, 1H, OH), 7.00 (d, 2H, $OC_6H_5$, $^3J_{HH}$ = 8.8), 7.11 (t, 1H, $OC_6H_5$, $^3J_{HH}$ = 7.4), 7.17 (t, 1H, $OC_6H_5$, $^3J_{HH}$ = 7.6), 7.22 (t, 2H, $OC_6H_5$, $^3J_{HH}$ =7.8), 7.32 (t, 2H, $OC_6H_5$, $^3J_{HH}$ = 7.8), 7.43 (s, 2H, $C\underline{H}CC(CH_3)_3$), 7.49 (d, 2H, $NHC_6\underline{H}_4OCH_3$, $^3J_{HH}$ = 8.9), 7.55 (d, 2H, $NHC_6\underline{H}_4OCH_3$, $^3J_{HH}$ = 9.0), 8.14 (d, 1H, $CH_{py}$, $^3J_{HH}$ = 8.5), 9.01 (s, 1H, NH), 9.44 (s, 1H, NH), 9.76 (s, 1H, NH), 10.30 (s, 1H, NH). $^{13}C\{^1H\}$ NMR (DMSO-$d_6$, 100.57 MHz), $\delta$, ppm: 30.69 [$C(\underline{C}H_3)_3$], 35.07 [$\underline{C}(CH_3)_3$], 42.72 (d, CHP, $^1J_{PC}$ = 140.4), 55.63 ($OCH_3$), 55.70 ($OCH_3$), 105.93 ($CH_{py}$), 113.02 (d, $C_{py}$, $^2J_{PC}$ = 3.3), 114.16, ($NHC_6H_4OCH_3$), 114.40 ($NHC_6H_4OCH_3$), 120.58 (d, $OC_6H_5$, $^3J_{PC}$ = 4.0), 120.75 (d, $OC_6H_5$, $^3J_{PC}$ = 4.0), 121.63 ($NHC_6H_4OCH_3$), 125.45 ($\underline{C}CHP$), 125.49 [$\underline{C}HCC(CH_3)_3$], 125.71 [$\underline{C}HCC(CH_3)_3$], 126.49 ($OC_6H_5$), 126.57 ($OC_6H_5$), 129.98 ($OC_6H_5$), 130.29 ($OC_6H_5$), 132.55 ($NH\underline{C}_6H_4OCH_3$), 132.74 ($NH\underline{C}_6H_4OCH_3$), 139.90 [$\underline{C}C(CH_3)_3$], 141.04 (d, $CH_{py}$, $^3J_{PC}$ = 5.1), 148.26 (d, $CH_{py}$, $^3J_{PC}$ = 12.1), 150.40 (d, $OC_6H_5$, $^2J_{PC}$ = 8.1), 150.44 ($C_{py}$), 150.67 (d, $OC_6H_5$, $^2J_{PC}$ = 10.1), 152.49 (C=O), 153.43 (COH), 155.32 ($NH\underline{C}_6H_4OCH_3$), 155.51 ($NH\underline{C}_6H_4OCH_3$). $^{31}$P NMR (DMSO-$d_6$, 242.94 MHz), $\delta$, ppm: 20.43. Anal. Calcd for $C_{48}H_{52}N_5O_8P$ (%): C, 67.20; H, 6.11; N, 8.16; P, 3.61. Found (%): C, 67.25; H, 6.14; N, 8.20; P, 3.64. HRMS (MALDI-TOF) m/z for $C_{48}H_{52}N_5O_8P$: calc. 857.4 [M]$^+$, found: 858.9 [M+H]$^+$; 880.9 [M+Na]$^+$, 896.9 [M+K]$^+$.

**Diethyl((2,6-bis(3-(*p*-chlorophenyl)ureido)pyridin-3-yl)(3,5-di-*tert*-butyl-4-hydroxyphenyl)methyl)phosphonate 11b.** White solid, yield 65%, m.p. 219-220°C. IR (KBr), $\nu$, cm$^{-1}$: 862; 1020; 1260; 1560; 1628, 1737; 3340; 3630. $^1$H NMR (DMSO-$d_6$, 400.13 MHz), $\delta$, ppm: 1.03 (t, 3H, $OCH_2CH_3$, $^3J_{HH}$ = 7.0), 1.10 (t, 3H, $OCH_2CH_3$, $^3J_{HH}$ = 7.0), 1.35 [s, 18H, $CH(CH_3)_2$], 3.83 (m, 1H, $OCH_2CH_3$), 3.94 (m, 1H, $OCH_2CH_3$), 3.94 (m, 2H, $OCH_2CH_3$), 4.86 (d, 1H, CHP, $^2J_{PH}$ = 26.9), 6.91 (d, 1H, CHpy, $^3J_{HH}$ = 8.9), 6.93 (s, 1H, OH), 7.30 (d, 2H, $NHC_6H_4Cl$, $^3J_{HH}$ = 8.9), 7.32 (s, 2H, $CHCC(CH_3)_3$), 7.40 (d, 2H, $NHC_6H_4Cl$, $^3J_{HH}$ = 8.9), 7.62 (d, 2H, $NHC_6H_4Cl$, $^3J_{HH}$ = 8.9), 7.69 (d, 2H,

$NHC_6H_4Cll$, $^3J_{HH}$ = 8.9), 8.02 (d, 1H, CHpy, $^3J_{HH}$ = 9.9), 9.07 (s, 1H, NH), 9.61 (s, 1H, NH), 9.95 (s, 1H, NH), 10.68 (s, 1H, NH). $^{13}C\{^1H\}$ NMR (DMSO-$d_6$, 100.57 MHz), $\delta$, ppm: 16.83 (d, $OCH_2CH_3$, $^3J_{PC}$ = 5.1), 16.98 (d, $OCH_2CH_3$, $^3J_{PC}$ = 5.1), 31.21 [$C(CH_3)_3$], 35.48 [$C(CH_3)_3$], 42.24 (d, CHP, $^1J_{PC}$ = 139.4), 62.81 (d, $OCH_2CH_3$, $^2J_{PC}$ = 7.1), 63.19 (d, $OCH_2CH_3$, $^2J_{PC}$ = 7.1), 106.58 (CHpy); 116.47 (CHpy), 114.61 (d, Cpy, $^2J_{PC}$ = 3.0), 121.90 ($NHC_6H_4Cl$), 122.05 ($NHC_6H_4Cll$), 126.52 [$CHCC(CH_3)_2$], 126.59 [$CHCC(CH_3)_2$], 126.80 ($NHC_6H_4Cl$), 126.87($NHC_6H_4Cl$), 127.42 (d, CCHP, $^2J_{PC}$ = 3.0), 130.47 (d, CHpy, $^3J_{PC}$ = 4.0), 129.90 ($NHC_6H_4Cll$), 130.17 ($NHC_6H_4Cl$), 139.27 ($NHC_6H_4Cl$), 139.29 ($NHC_6H_4Cl$), 139.97 [$CC(CH_3)_3$], 141.62 (d, CHpy, $^2J_{PC}$ = 3.0), 148.49 (d, Cpy, $^2J_{PC}$ = 10.1), 150.46 (Cpy), 152.79 (C=O), 152.95 (C=O), 153.79 (COH). $^{31}P$ NMR (DMSO-$d_6$, 161.90 MHz), $\delta$, ppm: 25.77. HRMS (MALDI-TOF/TOF) m/z for $C_{38}H_{46}Cl_2N_5O_6P$: calc. 769.3 [M]+, found: 770.4 [M+H]+, 792.4 [M+Na]+, 812.5 [M+K]+.

**Diisopropyl((2,6-bis(3-(*p*-chlorophenyl)ureido)pyridin-3-yl)(3,5-di-*tert*-butyl-4-hydroxyphenyl)methyl)phosphonate 11c.** White solid, yield 51%, M.p.: 222-223°C. IR (KBr), $\nu$, cm$^{-1}$:852; 1011; 1248; 1598; 1620, 1735; 3338; 3621. $^1H$ NMR (DMSO-$d_6$, 400.13 MHz), $\delta$, ppm: 0.81 (d, 3H, $CH(C\underline{H}_3)_2$, $^3J_{HH}$ = 6.1), 0.99 (d, 3H, $CH(C\underline{H}_3)_2$, $^3J_{HH}$ = 6.1), 1.19 [d, 6H, $OCH(\underline{CH}_3)_2$, $^3J_{HH}$ = 6.1], 1.35 [s, 18H, $C(CH_3)_3$], 4.40 (m, 1H, $C\underline{H}(CH_3)_2$), 4.52 (m, 1H, $C\underline{H}(CH_3)_2$), 4.73 (d, 1H, CHP, $^2J_{PH}$ = 26.9), 6.87 (d, 1H, $CH_{py}$, $^3J_{HH}$ = 8.6), 6.89 (s, 1H, OH), 7.30 (d, 2H, $NHC_6\underline{H}_4Cl$, $^3J_{HH}$ = 8.7), 7.36 (s, 2H, $C\underline{H}CC(CH_3)_3$), 7.40 (d, 1H, $NHC_6\underline{H}_4Cl$, $^3J_{HH}$ = 8.8), 7.61 (d, 1H, $NHC_6\underline{H}_4Cl$, $^3J_{HH}$ = 8.8), 7.70 (d, 1H, $NHC_6\underline{H}_4Cl$, $^3J_{HH}$ = 8.8), 8.06 (d, 1H, $CH_{py}$, $^3J_{HH}$ = 8.6), 9.10 (s, 1H, NH), 9.61 (s, 1H, NH), 9.89 (s, 1H, NH), 10.75 (s, 1H, NH). $^{13}C\{^1H\}$ NMR (DMSO-$d_6$, 100.57 MHz), $\delta$, ppm: 23.75 [d, $OCH(\underline{C}H_3)_2$, $^3J_{PC}$ = 5.1], 24.26 [d, $OCH(\underline{C}H_3)_2$, $^3J_{PC}$ = 4.5], 24.72 [$OCH(\underline{C}H_3)_2$], 24.99 [$OCH(\underline{C}H_3)_2$], 31.31 [$C(\underline{C}H_3)_3$]; 35.60 [$\underline{C}(CH_3)_3$], 43.57 (d, CHP, $^1J_{PC}$ = 138.4), 71.28 (d, $O\underline{C}H(CH_3)_2$, $^2J_{PC}$ = 8.1), 71.98 (d, $O\underline{C}H(CH_3)_2$, $^2J_{PC}$ = 8.1), 106.70 ($CH_{py}$); 115.79 ($C_{py}$), 121.58 ($NH\underline{C}_6H_4Cl$), 122.22 ($NH\underline{C}_6H_4Cl$), 126.80 ($NH\underline{C}_6H_4Cl$), 126.88 ($NH\underline{C}_6H_4Cl$), 127.17 [$\underline{C}HCC(CH_3)_2$], 127.21 [$\underline{C}HCC(CH_3)_2$], 127.59 ($\underline{C}CHP$), 129.30 ($NH\underline{C}_6H_4Cl$), 129.64 ($NH\underline{C}_6H_4Cl$), 139.21 ($NH\underline{C}_6H_4Cl$), 139.27 ($NH\underline{C}_6H_4Cl$), 140.14 [$\underline{C}C(CH_3)_3$], 141.71 ($CH_{py}$), 148.33 ($C_{py}$), 150.36 ($C_{py}$), 152.70 (C=O), 153.00 (C=O), 153.94 (COH). $^{31}P$ NMR (DMSO-$d_6$, 161.90 MHz), $\delta$, ppm: 23.34. Anal. Calcd for $C_{40}H_{50}Cl_2N_5O_6P$ (%): C, 60.15; H, 6.31; Cl, 8.88; N, 8.77; P, 3.88. Found (%): C, 60.20; H, 6.33; Cl, 8.90; N, 8.80; P, 3.90. HRMS (MALDI-TOF) m/z for $C_{40}H_{50}Cl_2N_5O_6P$: calc. 797.3 [M]$^+$, found: 798.4 [M+H]$^+$, 820.4 [M+Na]$^+$, 836.4 [M+K]$^+$.

**Diphenyl((2,6-bis(3-(*p*-chlorophenyl)ureido)pyridin-3-yl)(3,5-di-*tert*-butyl-4-hydroxyphenyl)methyl)phosphonate 11d.** White solid, yield 66%, M.p.: 292-293°C. IR (KBr), $\nu$, cm$^{-1}$:827; 1092; 1244; 1600; 1662, 1720; 2955; 3078, 3367. $^1H$ NMR (DMSO-$d_6$, 400.13 MHz), $\delta$, ppm: 1.31 [s, 18H, $C(CH_3)_3$], 5.46 (d, 1H, CHP, $^2J_{PH}$ = 28.3), 6.68 (d, 2H, $OC_6H_5$, $^3J_{HH}$ = 8.4), 6.97 (d, 1H, $CH_{py}$, $^3J_{HH}$ = 8.6), 7.00 (s, 1H, OH), 7.00 (d, 2H, $OC_6H_5$, $^3J_{HH}$ = 8.4), 7.11 (t, 1H, $OC_6H_5$, $^3J_{HH}$ = 7.4), 7.17 (t, 1H, $OC_6H_5$, $^3J_{HH}$ = 7.2), 7.22 (t, 2H, $OC_6H_5$, $^3J_{HH}$ = 7.8), 7.31 (d, 2H, $NHC_6\underline{H}_4Cl$, $^3J_{HH}$ = 8.9), 7.32 (t, 2H, $OC_6H_5$, $^3J_{HH}$ = 7.8), 7.41 (d, 2H, $NHC_6\underline{H}_4Cl$, $^3J_{HH}$ = 8.9), 7.42 (s, 2H, $C\underline{H}CC(CH_3)_3$), 7.62 (d, 2H, $NHC_6\underline{H}_4Cl$, $^3J_{HH}$ = 8.9), 7.69 (d, 2H, $NHC_6\underline{H}_4Cl$, $^3J_{HH}$ = 8.9), 8.17 (dd, 1H, $CH_{py}$, $^3J_{HH}$ = 8.5, 1.3), 9.22 (s, 1H, NH), 9.66 (s, 1H, NH), 10.01 (s, 1H, NH), 10.63 (s, 1H, NH). $^{13}C\{^1H\}$ NMR (DMSO-$d_6$, 125.76 MHz), $\delta$, ppm: 30.69 [$C(\underline{C}H_3)_3$], 35.08 [$\underline{C}(CH_3)_3$], 42.73 (d, CHP, $^1J_{PC}$ = 138.6), 106.52 ($CH_{py}$), 113.79 ($C_{py}$), 120.59 (d, $OC_6H_5$, $^3J_{PC}$ = 2.5), 120.75 (d, $OC_6H_5$, $^3J_{PC}$ = 2.5), 121.20, ($NHC_6H_5Cl$), 121.59 ($NHC_6H_5Cl$), 125.42 (d, $\underline{C}CHP$, $^2J_{PC}$ = 5.0), 125.51 [$\underline{C}HCC(CH_3)_3$], 125.72 [$\underline{C}HCC(CH_3)_3$], 126.51 ($NHC_6H_5Cl$), 126.58 ($NHC_6H_5Cl$), 126.66 ($OC_6H_5$), 126.71 ($OC_6H_5$), 128.84 ($NHC_6H_5Cl$), 129.10 ($NHC_6H_5Cl$), 129.99 ($OC_6H_5$), 130.29 ($OC_6H_5$), 138.60 ($NHC_6H_5Cl$), 138.67 ($NHC_6H_5Cl$), 139.92 [$\underline{C}C(CH_3)_3$], 141.25 (d, $CH_{py}$, $^3J_{PC}$ = 6.3), 147.92 (d, $CH_{py}$, $^3J_{PC}$ = 11.3), 150.24 ($C_{py}$), 150.38 (d, $OC_6H_5$, $^2J_{PC}$ = 10.1), 150.65 (d, $OC_6H_5$, $^2J_{PC}$ = 10.1), 152.36 (C=O), 152.39 (C=O), 153.93 (COH). $^{31}P$ NMR

(DMSO-$d_6$, 161.90 MHz), $\delta$, ppm: 19.19. Anal. Calcd for $C_{46}H_{46}Cl_2N_5O_6P$ (%): C, 63.74; H, 5.35; Cl, 8.18; N, 8.08; P, 3.57. Found (%): C, 63.77; H, 5.36; Cl, 8.20; N, 8.10; P, 3.60. HRMS (MALDI-TOF) m/z for $C_{46}H_{46}Cl_2N_5O_6P$: calc. 865.3 $[M]^+$, found: 866.6 $[M+H]^+$; 888.9 $[M+Na]^+$, 904.7 $[M+K]^+$.

**Diisopropyl((2,6-bis(3-(*p*-chlorophenyl)ureido)pyridin-3-yl)(3,5-di-*tert*-butyl-4-hydroxyphenyl)methyl)phosphonate 12c.** Yellow solid, yield 52%, M.p.: 273-274°C. IR (KBr), $\nu$, $cm^{-1}$:848; 1026; 1210; 1595; 1680, 1713; 2957; 3326, 3602. $^1H$ NMR (DMSO-$d_6$, 600.13 MHz), $\delta$, ppm: 0.81 (d, 3H, CH($C\underline{H}_3$)$_2$, $^3J_{HH}$ = 5.8), 0.99 (d, 3H, CH($C\underline{H}_3$)$_2$, $^3J_{HH}$ = 5.9), 1.18 [m, 6H, OCH($\underline{CH_3}$)$_2$], 1.33 [s, 18H, C($CH_3$)$_3$], 4.42 (m, 1H, C$\underline{H}$($CH_3$)$_2$), 4.52 (m, 1H, C$\underline{H}$($CH_3$)$_2$), 4.78 (d, 1H, CHP, $^2J_{PH}$ = 27.7), 6.90 (s, 1H, OH), 6.98 (d, 1H, $CH_{py}$,$^3J_{HH}$ = 8.7), 7.36 (s, 2H, C$\underline{H}$CC($CH_3$)$_3$), 7.85 (d, 2H, $NHC_6\underline{H}_4NO_2$, $^3J_{HH}$ = 8.9), 7.91 (d, 1H, $NHC_6\underline{H}_4NO_2$, $^3J_{HH}$ = 8.7), 8.10 (d, 1H, $NHC_6\underline{H}_4NO_2$, $^3J_{HH}$ = 9.1), 8.16 (d, 1H, $NHC_6\underline{H}_4NO_2$,$^3J_{HH}$ = 9.0), 8.27 (d, 1H, $CH_{py}$, $^3J_{HH}$ = 8.7), 9.36 (s, 1H, NH), 9.88 (s, 1H, NH), 10.38 (s, 1H, NH), 11.15 (s, 1H, NH). $^{13}C\{^1H\}$ NMR (DMSO-$d_6$, 100.57 MHz), $\delta$, ppm: 23.20 [d, OCH($\underline{C}H_3$)$_2$, $^3J_{PC}$ = 5.3], 23.73 [d, OCH($\underline{C}H_3$)$_2$, $^3J_{PC}$ = 5.3], 24.16 [d, OCH($\underline{C}H_3$)$_2$, $^3J_{PC}$ = 3.4], 24.46 [d, OCH($\underline{C}H_3$)$_2$, $^3J_{PC}$ = 3.4], 30.75 [C($\underline{C}H_3$)$_3$]; 35.06 [$\underline{C}$($CH_3$)$_3$], 43.17 (d, CHP, $^1J_{PC}$ = 141.4), 70.83 [d, O$\underline{C}$H($\underline{C}H_3$)$_2$, $^2J_{PC}$ = 7.1], 71.59 [d, O$\underline{C}$H($\underline{C}H_3$)$_2$, $^2J_{PC}$ = 7.1], 107.15 ($CH_{py}$), 116.53 [d, $C_{py}$, $^2J_{PC}$ = 4.1], 118.88 ($NHC_6H_4NO_2$), 119.55 ($NHC_6H_4NO_2$), 125.19 ($NHC_6H_4NO_2$), 125.54 ($NHC_6H_4NO_2$), 126.30 [$\underline{C}$HCC($CH_3$)$_3$], 126.37 [$\underline{C}$HCC($CH_3$)$_3$], 126.92 (d, $\underline{C}$CHP, $^2J_{PC}$ = 5.1), 139.66 [$\underline{C}$C($CH_3$)$_3$], 141.51 [d, $CH_{py}$, $^3J_{PC}$ = 5.1], 142.16 ($NHC_6H_4NO_2$), 142.22 ($NHC_6H_4NO_2$), 146.16 ($NHC_6H_4NO_2$), 146.22 ($NHC_6H_4NO_2$), 141.51 (d, $C_{py}$, $^3J_{PC}$ = 10.1), 149.56 ($C_{py}$), 151.98 (C=O), 152.21 (C=O), 153.46 (COH). $^{31}P$ NMR (DMSO-$d_6$, 242.94 MHz), $\delta$, ppm: 24.35. HRMS (MALDI-TOF) m/z for $C_{40}H_{50}N_7O_{10}P$: calc. 819.3 $[M]^+$, found: 819.7 $[M+H]^+$.

**Diphenyl((2,6-bis(3-(*p*-chlorophenyl)ureido)pyridin-3-yl)(3,5-di-*tert*-butyl-4-hydroxyphenyl)methyl)phosphonate 12d.** Yellow solid, yield 60%, M.p.: 292-293°C. IR (KBr), $\nu$, $cm^{-1}$:847; 1032; 1248; 1598; 1622, 1736; 2958; 3337, 3602. $^1H$ NMR (DMSO-$d_6$, 600.13 MHz), $\delta$, ppm: 1.31 [s, 18H, C($CH_3$)$_3$], 5.47 (d, 1H, CHP, $^2J_{PH}$ = 28.0), 6.68 (d, 2H, $OC_6H_5$, $^3J_{HH}$ = 8.0), 7.00 (d, 2H, $OC_6H_5$, $^3J_{HH}$ = 8.4), 7.01 (s, 1H, OH), 7.11 (m, 2H, $CH_{py}$, $OC_6H_5$), 7.17 (t, 1H, $OC_6H_5$, $^3J_{HH}$ = 7.3), 7.22 (t, 2H, $OC_6H_5$, $^3J_{HH}$ = 7.9), 7.32 (t, 2H, $OC_6H_5$, $^3J_{HH}$ = 7.9), 7.42 (s, 2H, C$\underline{H}$CC($CH_3$)$_3$), 7.85 (d, 2H, $NHC_6\underline{H}_4NO_2$, $^3J_{HH}$ = 9.0), 7.89 (d, 2H, $NHC_6\underline{H}_4NO_2$, $^3J_{HH}$ = 9.3), 8.17 (d, 2H, $NHC_6\underline{H}_4NO_2$, $^3J_{HH}$ = 9.2), 8.22 (d, 1H, $CH_{py}$, $^3J_{HH}$ = 8.6), 8.26 (d, 2H, $NHC_6\underline{H}_4NO_2$, $^3J_{HH}$ = 9.1), 9.41 (s, 1H, NH), 9.91 (s, 1H, NH), 10.39 (s, 1H, NH), 10.97 (s, 1H, NH). $^{13}C\{^1H\}$ NMR (DMSO-$d_6$, 100.57 MHz), $\delta$, ppm: 31.16 [C($\underline{C}H_3$)$_3$]; 35.51 [$\underline{C}$($CH_3$)$_3$], 44.44 (d, CHP, $^1J_{PC}$ = 140.4), 113.38 ($CH_{py}$), 116.84 ($CH_{py}$), 118.42 ($NHC_6H_4NO_2$), 118.53 ($NHC_6H_4NO_2$), 121.10 (d, $OC_6H_5$, $^2J_{PC}$ = 4.0), 121.41 (d, $OC_6H_5$, $^2J_{PC}$ = 4.0), 125.94 ($OC_6H_5$), 126.11 ($NHC_6H_4NO_2$), 126.21 ($OC_6H_5$), 126.99 $\underline{C}$HCC($CH_3$)$_3$, 127.07 $\underline{C}$HCC($CH_3$)$_3$, 127.34 ($\underline{C}$CHP), 130.46 ($OC_6H_5$), 130.77 ($OC_6H_5$), 139.45 ($C_{py}$), 140.27 [$\underline{C}$C($CH_3$)$_3$], 142.04 ($NHC_6H_4NO_2$), 142.09 ($NHC_6H_4NO_2$), 147.23 ($NHC_6H_4NO_2$), 147.44 ($NHC_6H_4NO_2$), 148.98 [d, $CH_{py}$, $^3J_{PC}$ = 4.0], 150.90 (d, $OC_6H_5$, $^2J_{PC}$ = 10.1), 151.24 (d, $OC_6H_5$, $^2J_{PC}$ = 10.1), 152.83 ($C_{py}$), 153.50 (COH), 154.26 (C=O), 154.27 (C=O). $^{31}P$ NMR (DMSO-$d_6$, 242.94 MHz), $\delta$, ppm: 19.12. Anal. Calcd for $C_{46}H_{46}N_7O_8P$ (%): C, 62.23; H, 5.22; N, 11.04; P, 3.49. Found (%): C, 62.27; H, 5.23; N, 11.07; P, 3.51. HRMS (MALDI-TOF) m/z for $C_{46}H_{46}N_7O_{10}P$: calc. 887.3 $[M]^+$, found: 888.5 $[M+H]^+$; 910.6 $[M+Na]^+$.

**Dimethyl((2,4-bis(3-(*m*-tolyl)ureido)phenyl)(3,5-di-*tert*-butyl-4-hydroxyphenyl)methyl)phosphonate 13a.** White solid, yield 88%, M.p.: 252-253°C. IR (KBr), $\nu$, $cm^{-1}$:830; 1039; 1225; 1559; 1611, 1639; 2954; 3325, 3620. $^1H$ NMR (DMSO-$d_6$, 600.13 MHz), $\delta$, ppm: 1.33 [s, 18H, C($CH_3$)$_3$], 2.28 (s, 6H, $CH_3$), 3.51 (t, 6H, $OCH_3$, $^3J_{PH}$ = 11.9), 4.74 (d, 3H, $OCH_3$, $^3J_{PH}$ = 27.1), 6.79 (s, 1H, $NHC_6\underline{H}_4CH_3$), 6.79 (s, 1H, OH), 6.86 (s, 1H, $NHC_6\underline{H}_4CH_3$), 7.12-7.31 (m, 7H, $CH_{m-}$

Ph, $NHC_6\underline{H}_4CH_3$), 7.31 (s, 2H, $C\underline{H}CC(CH_3)_3$), 7.64 (d, 1H, $CH_{m\text{-}Ph}$, $^3J_{HH}$ = 9.0), 7.79 (s, 1H, $CH_{m\text{-}Ph}$), 8.15 (s, 1H, NH), 8.49 (s, 1H, NH), 8.68 (s, 1H, NH), 8.77 (s, 1H, NH). $^{13}C\{^1H\}$ NMR (DMSO-$d_6$, 125.76 MHz), $\delta$, ppm: 21.68 ($CH_3$), 21.70 ($CH_3$), 30.81 [$C(\underline{C}H_3)_3$], 35.02 [$\underline{C}(CH_3)_3$], 42.02 (d, CHP, $^1J_{PC}$ = 139.9), 53.35 (d, $OCH_3$, $^2J_{PC}$ = 7.6), 53.43 (d, $OCH_3$, $^2J_{PC}$ = 7.6), 114.42 (CH), 114.86 ($CH_{m\text{-}Ph}$), 115.84 ($NH\underline{C}_6H_4CH_3$), 119.19 ($NH\underline{C}_6H_4CH_3$), 123.08 ($NH\underline{C}_6H_5CH_3$), 123.59 (d, $C_{m\text{-}Ph}$, $^2J_{PC}$ = 1.3), 126.00 [$\underline{C}HCC(CH_3)_3$], 126.06 [$\underline{C}HCC(CH_3)_3$], 127.78 (d, $\underline{C}CHP$, $^2J_{PC}$ = 3.8), 129.07 ($NH\underline{C}_6H_4CH_3$), 129.82 ($CH_{m\text{-}Ph}$), 137.18 (d, $C_{m\text{-}Ph}$, $^3J_{PC}$ = 3.8), 138.39 ($NH\underline{C}_6H_4CH_3$), 138.42 ($NH\underline{C}_6H_4CH_3$), 139.12 ($C_{m\text{-}Ph}$), 139.50 [$\underline{C}C(CH_3)_3$], 140.01 ($NH\underline{C}_6H_4CH_3$), 140.23 ($NH\underline{C}_6H_4CH_3$), 152.87 (C=O), 152.98 (C=O), 153.38 ( COH). $^{31}P$ NMR (DMSO-$d_6$, 242.94 MHz), $\delta$, ppm: 28.85. Anal. Calcd for $C_{39}H_{49}N_4O_6P$ (%): C, 66.84; H, 7.05; N, 7.99; P, 4.42. Found (%): C, 66.87; H, 7.08; N, 8.00; P, 4.46. HRMS (MALDI-TOF) m/z for $C_{39}H_{49}N_4O_6P$: calc. 700.3 $[M]^+$, found: 701.5 $[M+H]^+$; 723.5 $[M+Na]^+$, 739.5 $[M+K]^+$.

**Diethyl((2,4-bis(3-(*m*-tolyl)ureido)phenyl)(3,5-di-*tert*-butyl-4-hydroxyphenyl)methyl)phosphonate 13b.** White solid, yield 80%, M.p.: 235-236°C. IR (KBr), $\nu$, $cm^{-1}$: 775; 1019; 1050; 1201; 1560; 1669, 1732; 2957; 3306, 3645. $^1H$ NMR (DMSO-$d_6$, 400.13 MHz), $\delta$, ppm: 1.04 (t, 3H, $OCH_2C\underline{H}_3$, $^3J_{HH}$ = 7.0), 1.11 (t, 3H, $OCH_2C\underline{H}_3$, $^3J_{HH}$ = 7.0), 1.35 [s, 18H, $C(CH_3)_3$], 2.21 (s, 3H, $NHC_6H_4C\underline{H}_3$), 2.29 (s, 3H, $NHC_6H_4C\underline{H}_3$), 3.80 - 4.00 (m, 4H, $OC\underline{H}_2CH_3$), 4.84 (d, 1H, CHP, $^2J_{PH}$ = 26.9), 6.81 (d, 1H, $NHC_6\underline{H}_4CH_3$,$^3J_{HH}$ = 7.6), 6.85 (d, 1H, $NHC_6\underline{H}_4CH_3$, $^3J_{HH}$ = 7.6), 6.89 (d, 1H, $C_{m\text{-}Ph}$, $^3J_{HH}$ = 8.5), 6.92 (s, 1H, OH), 7.10-7.23 (m, 4H, $NHC_6\underline{H}_4CH_3$,), 7.33 (s, 2H, $C\underline{H}CC(CH_3)_3$), 7.40 (s, 1H, $NHC_6\underline{H}_4CH_3$), 7.40 (d, 1H, $NHC_6\underline{H}_4CH_3$, $^3J_{HH}$ = 6.6), 7.46 (s, 1H, $NHC_6\underline{H}_4CH_3$), 7.52 (d, 1H, $NHC_6\underline{H}_4CH_3$, $^3J_{HH}$ = 8.5), 8.02 (d, 1H, $CH_{m\text{-}Ph}$, $^3J_{HH}$ = 8.6), 8.53 (s, 1H, $CH_{m\text{-}Ph}$), 8.95 (s, 1H, NH), 9.50 (s, 1H, NH), 9.72 (s, 1H, NH), 10.57 (s, 1H, NH). $^{13}C\{^1H\}$ NMR (DMSO-$d_6$, 100.57 MHz), $\delta$, ppm: 16.42 (d, $OCH_2\underline{C}H_3$, $^3J_{PC}$ = 6.1), 16.57 (d, $OCH_2\underline{C}H_3$, $^3J_{PC}$ = 6.1), 21.67 ($CH_3$), 30.80 [$C(\underline{C}H_3)_3$], 35.07 [$\underline{C}(CH_3)_3$], 42.40 (d, CHP, $^1J_{PC}$ = 140.4), 62.45 (d, $O\underline{C}H_2CH_3$, $^2J_{PC}$ = 7.1), 62.84 (d, $O\underline{C}H_2CH_3$, $^2J_{PC}$ = 7.1), 114.05 ($CH_{m\text{-}Ph}$), 114.82 ($CH_{m\text{-}Ph}$), 115.83 ($NH\underline{C}_6H_4CH_3$), 119.17 ($NH\underline{C}_6H_4CH_3$), 123.01 ($NH\underline{C}_6H_4CH_3$), 123.99 ($C_{m\text{-}Ph}$), 126.09 [$\underline{C}HCC(CH_3)_3$], 126.17 [$\underline{C}HCC(CH_3)_3$], 126.97 (d, $\underline{C}CHP$, $^2J_{PC}$ = 4.0), 129.07 ($NH\underline{C}_6H_4CH_3$), 129.43 ($CH_{m\text{-}Ph}$), 136.76 (d, $C_{m\text{-}Ph}$, $^3J_{PC}$ = 4.0), 138.12 ($C_{m\text{-}Ph}$), 138.36 ($NH\underline{C}_6H_4CH_3$), 138.40 ($NH\underline{C}_6H_4CH_3$), 138.47 ($NH\underline{C}_6H_4CH_3$), 139.63 [$\underline{C}C(CH_3)_3$], 152.14 (C=O), 152.46 (C=O), 152.99 (COH). $^{31}P$ NMR (DMSO-$d_6$, 242.94 MHz), $\delta$, ppm: 25.80. Anal. Calcd for $C_{41}H_{53}N_4O_6P$ (%): C, 67.56; H, 7.33; N, 7.69; P, 4.25. Found (%): C, 67.57; H, 7.35; N, 7.72; P, 4.26. HRMS (MALDI-TOF) m/z for $C_{41}H_{53}N_4O_6P$: calc. 728.4 $[M]^+$, found: 729.4 $[M+H]^+$, 751.4 $[M+Na]^+$, 767.4 $[M+K]^+$.

**Diphenyl((2,4-bis(3-(*m*-tolyl)ureido)phenyl)(3,5-di-*tert*-butyl-4-hydroxyphenyl)methyl)phosphonate 13d.** White solid, yield 88%, M.p.: 231-232°C. IR (KBr), $\nu$, $cm^{-1}$: 817; 1028; 1259; 1576; 1600; 1638, 2966; 3335; 3469. $^1H$ NMR (DMSO-$d_6$, 400.13 MHz), $\delta$, ppm: 1.30 [s, 18H, $C(CH_3)_3$], 2.28 (s, 3H, $CH_3$), 2.29 (s, 3H, $CH_3$), 5.20 (d, 3H, $OCH_3$, $^3J_{PH}$ = 27.2), 6.65 (d, 2H, $OC_6H_5$, $^3J_{HH}$ = 8.0), 6.80 (t, 1H, $CH_{m\text{-}Ph}$, $^3J_{HH}$ = 8.0), 6.94 (s, 1H, OH), 6.95 (d, 2H, $OC_6H_5$, $^3J_{HH}$ = 8.0), 7.10 (t, 1H, $OC_6H_5$, $^3J_{HH}$ = 8.0), 7.15-7.25 (m, 7H, $OC_6H_5$, $NHC_6\underline{H}_4CH_3$), 7.25-7,28 (m, 5H, $NHC_6\underline{H}_4CH_3$, $CH_{m\text{-}Ph}$), 7.41 (s, 2H, $C\underline{H}CC(CH_3)_3$), 7.82 (dd, 1H, $CH_{m\text{-}Ph}$, $^3J_{HH}$ = 8.8, 1.6), 7.85 (s, 1H, $CH_{m\text{-}Ph}$), 8.26 (s, 1H, NH), 8.52 (s, 1H, NH), 8.74 (s, 1H, NH), 8.78 (s, 1H, NH). $^{13}C\{^1H\}$ NMR (DMSO-$d_6$, 100.57 MHz), $\delta$, ppm: 21.66 ($CH_3$), 21.70 ($CH_3$), 30.67 [$C(\underline{C}H_3)_3$], 34.99 [$\underline{C}(CH_3)_3$], 43.74 (d, CHP, $^1J_{PC}$ = 140.4), 114.68 ($CH_{m\text{-}Ph}$), 115.21 ($CH_{m\text{-}Ph}$), 115.79 ($NH\underline{C}_6H_4CH_3$), 115.91 ($NH\underline{C}_6H_4CH_3$), 119.19 ($NH\underline{C}_6H_4CH_3$), 119.25 ($NH\underline{C}_6H_4CH_3$), 120.59 (d, $OC_6H_5$, $^3J_{PC}$ = 4.0), 120.89 (d, $OC_6H_5$, $^3J_{PC}$ = 4.0), 122.95 (d, $C_{m\text{-}Ph}$, $^2J_{PC}$ = 2.0), 123.06 ($NH\underline{C}_6H_4CH_3$), 123.16 ($NH\underline{C}_6H_5CH_3$), 125.40 ($OC_6H_5$), 125.68 ($OC_6H_5$), 126.19 (d, $\underline{C}CHP$, $^2J_{PC}$ = 4.0), 126.52 [$\underline{C}HCC(CH_3)_3$], 126.60 [$\underline{C}HCC(CH_3)_3$], 129.10 ($NH\underline{C}_6H_4CH_3$), 129.68 (d, $CH_{m\text{-}Ph}$, $^3J_{PC}$ = 4.0), 129.94 ($OC_6H_5$), 130.24 ($OC_6H_5$), 137.44 (д, $C_{m\text{-}Ph}$, $^3J_{PC}$ =

12.1), 138.42 ($NH\underline{C}_6H_4CH_3$), 138.46 ($NH\underline{C}_6H_4CH_3$), 139.55 ($C_{m\text{-}Ph}$), 139.75 [$\underline{C}C(CH_3)_3$], 139.95 ($NH\underline{C}_6H_4CH_3$), 140.15 ($NH\underline{C}_6H_4CH_3$), 150.42 (d, $OC_6H_5$, $^2J_{PC}$ = 11.1), 150.64 (d, $OC_6H_5$, $^2J_{PC}$ = 10.1), 152.88 (C=O), 153.45 (C=O), 153.72 (COH). $^{31}$P NMR (DMSO-$d_6$, 161.90 MHz), $\delta$, ppm: 20.23. Anal. Calcd for $C_{49}H_{53}N_4O_6P$ (%): C, 71.34; H, 6.48; N, 6.79; P, 3.75. Found (%): C, 71.37; H, 6.51; N, 6.81; P, 3.78. HRMS (MALDI-TOF) m/z for $C_{49}H_{53}N_4O_6P$: calc. 824.4 $[M]^+$, found: 825.7 $[M+H]^+$, 863.6 $[M+K]^+$.

**Dimethyl((2,4-bis-(3-(*p*-tolyl)ureido)phenyl)(3,5-di-*tert*-butyl-4-hydroxyphenyl)methyl)phosphonate 14a.** White solid, yield 78%, M.p.: 248-249°C. IR (KBr), $\nu$, cm$^{-1}$: 816; 1039; 1237; 1550; 1600; 1641, 2954; 3309; 3625. $^1$H NMR (DMSO-$d_6$, 400.13 MHz), $\delta$, ppm: 1.32 [s, 18H, $C(CH_3)_3$], 2.24 (s, 3H, $CH_3$), 2.25 (s, 3H, $CH_3$), 3.50 (t, 6H, $OCH_3$, $^3J_{PH}$ = 10.2), 4.74 (d, 1H, CHP, $^2J_{PH}$ = 26.4), 6.87 (s, 1H, OH), 7.08 (dd, 4H, $NHC_6\underline{H}_4CH_3$, $^3J_{HH}$ = 8.1, 4.3), 7.24 (d, 1H, $CH_{m\text{-}Ph}$, $^3J_{HH}$ = 8.5, 2.4), 7.33 (m, 6H, $C\underline{H}CC(CH_3)_3$, $NHC_6\underline{H}_4CH_3$), 7.64 (dd, 1H, $CH_{m\text{-}Ph}$, $^3J_{HH}$ = 8.6, 1.5), 7.75 (s, 1H, $CH_{m\text{-}Ph}$), 8.12 (s, 1H, NH), 8.46 (s, 1H, NH), 8.64 (s, 1H, NH), 8.74 (s, 1H, NH). $^{13}C\{^1H\}$ NMR (DMSO-$d_6$, 100.57 MHz), $\delta$, ppm: 20.79 ($CH_3$), 30.82 [$C(\underline{C}H_3)_3$], 35.02 [$\underline{C}(CH_3)_3$], 42.54 (d, CHP, $^1J_{PC}$ = 139.4), 53.33 (d, $POCH_3$, $^2J_{PC}$ = 7.1), 53.40 (d, $POCH_3$, $^2J_{PC}$ = 7.1), 114.25 ($CH_{m\text{-}Ph}$); 114.66 ($CH_{m\text{-}Ph}$), 118.63 ($NH\underline{C}_6H_4CH_3$), 118.75 ($NH\underline{C}_6H_4CH_3$), 123.32 (d, $C_{m\text{-}Ph}$, $^2J_{PC}$ = 3.0), 125.99 [$\underline{C}HCC(CH_3)_2$], 126.07 [$\underline{C}HCC(CH_3)_2$], 127.81 (d, $\underline{C}CHP$, $^2J_{PC}$ = 3.0), 129.62 ($NH\underline{C}_6H_4CH_3$), 129.81 (d, $CH_{m\text{-}Ph}$, $^3J_{PC}$ = 5.1), 131.00 ($NH\underline{C}_6H_4CH_3$), 131.12 ($NH\underline{C}_6H_4CH_3$), 137.23 (d, $C_{m\text{-}Ph}$, $^3J_{PC}$ = 11.1), 137.55 ($NH\underline{C}_6H_4CH_3$), 137.77 ($NH\underline{C}_6H_4CH_3$), 139.20 ($C_{m\text{-}Ph}$), 139.48 [$\underline{C}C(CH_3)_3$], 152.93 (C=O), 153.24 (COH), 153.39 (C=O). $^{31}$P NMR (DMSO-$d_6$, 161.90 MHz), $\delta$, ppm: 28.77. Anal. Calcd for $C_{39}H_{49}N_4O_6P$ (%): C, 66.84; H, 7.05; N, 7.99; P, 4.42. Found (%): C, 66.85; H, 7.07; N, 8.01; P, 4.45. HRMS (MALDI-TOF) m/z for $C_{39}H_{49}N_4O_6P$: calc. 700.3 $[M]^+$, found: 723.5 $[M+Na]^+$, 739.5 $[M+K]^+$.

**Diethyl((2,4-bis-(3-(*p*-tolyl)ureido)phenyl)(3,5-di-*tert*-butyl-4-hydroxyphenyl)methyl)phosphonate 14b.** White solid, yield 68%, M.p.: 247-248°C. IR (KBr), $\nu$, cm$^{-1}$: 815; 1027; 1053; 1224; 1548; 1603; 1642, 2960; 3325; 3620. $^1$H NMR (DMSO-$d_6$, 600.13 MHz), $\delta$, ppm: 1.01 (t, 3H, $OCH_2C\underline{H}_3$, $^3J_{HH}$ = 7.0), 1.10 (t, 3H, $OCH_2C\underline{H}_3$, $^3J_{HH}$ = 7.0), 1.33 [s, 18H, $CH(C\underline{H}_3)_2$], 2.24 (s, 3H, $CH_3$), 2.25 (s, 3H, $CH_3$), 3.78 (m, 1H, $OC\underline{H}_2CH_3$), 3.84 (m, 1H, $OC\underline{H}_2CH_3$), 3.90 (m, 2H, $OC\underline{H}_2CH_3$), 4.68 (d, 1H, CHP, $^2J_{PH}$ = 26.5), 6.84 (s, 1H, OH), 7.08 (t, 4H, $NHC_6\underline{H}_4CH_3$, $^3J_{HH}$ = 8.0), 7.25 (d, 1H, $CH_{m\text{-}Ph}$, $^3J_{HH}$ = 7.0), 7.31 (s, 2H, $C\underline{H}CC(CH_3)_3$ ), 7.33 (t, 4H, $NHC_6\underline{H}_4CH_3$, $^3J_{HH}$ = 8.0), 8.00 (d, 1H, $CH_{m\text{-}Ph}$, $^3J_{HH}$ = 8.6), 7.78 (s, 1H, $CH_{m\text{-}Ph}$), 8.12 (s, 1H, N$\underline{H}$), 8.44 (s, 1H, N$\underline{H}$), 8.64 (s, 1H, N$\underline{H}$), 8.77 (s, 1H, N$\underline{H}$). $^{13}C\{^1H\}$ NMR (DMSO-$d_6$, 100.57 MHz), $\delta$, ppm: 16.97 (d, $OCH_2\underline{C}H_3$, $^3J_{PC}$ = 5.1), 17.12 (d, $OCH_2\underline{C}H_3$, $^3J_{PC}$ = 5.1), 21.32 ($CH_3$), 31.34 [$C(\underline{C}H_3)_3$], 35.54 [$\underline{C}(CH_3)_3$], 43.66 (d, CHP, $^1J_{PC}$ = 140.4), 62.80 (d, $O\underline{C}H_2CH_3$, $^2J_{PC}$ = 6.1), 63.05 (d, $O\underline{C}H_2CH_3$, $^2J_{PC}$ = 6.1), 114.48 ($CH_{m\text{-}Ph}$); 114.78 ($CH_{m\text{-}Ph}$), 119.13 ($NH\underline{C}_6H_4CH_3$), 119.27 ($NH\underline{C}_6H_4CH_3$), 123.74 ($C_{m\text{-}Ph}$), 126.63 [$\underline{C}HCC(CH_3)_2$], 126.70 [$\underline{C}HCC(CH_3)_2$], 128.47 ($\underline{C}CHP$), 130.14 ($NH\underline{C}_6H_4CH_3$), 130.21 (d, $CH_{m\text{-}Ph}$, $^3J_{PC}$ = 5.1), 131.50 ($NH\underline{C}_6H_4CH_3$), 131.62 ($NH\underline{C}_6H_4CH_3$), 137.85 (d, $C_{m\text{-}Ph}$, $^3J_{PC}$ = 10.1), 138.07 ($NH\underline{C}_6H_4CH_3$), 138.30 ($NH\underline{C}_6H_4CH_3$), 139.60 ($C_{m\text{-}Ph}$), 139.91 [$\underline{C}C(CH_3)_3$], 153.44 (C=O), 153.67 (COH), 153.83 (C=O).$^{31}$P NMR (DMSO-$d_6$, 242.94 MHz), $\delta$, ppm: 26.75. Anal. Calcd for $C_{41}H_{53}N_4O_6P$ (%): C, 67.56; H, 7.33; N, 7.69; P, 4.25. Found (%): C, 67.59; H, 7.35; N, 7.72; P, 4.27. HRMS (MALDI-TOF) m/z for $C_{41}H_{53}N_4O_6P$: calc. 728.4 $[M]^+$, found: 751.3 $[M+Na]^+$.

**Diphenyl((2,4-bis-(3-(*p*-tolyl)ureido)phenyl)(3,5-di-*tert*-butyl-4-hydroxyphenyl)methyl)phosphonate 14d.** White solid, yield 75%, M.p.: 231-232°C. IR (KBr), $\nu$, cm$^{-1}$: 815; 1026; 1208; 1550; 1603; 1642, 2956; 3323; 3627. $^1$H NMR (DMSO-$d_6$, 400.13 MHz), $\delta$, ppm: 1.29 [s, 18H, $C(CH_3)_3$], 2.25 (s, 3H, $CH_3$), 2.26 (s, 3H, $CH_3$), 5.20 (d, 1H, CHP, $^2J_{PH}$ = 27.3), 6.64 (d, 2H, $OC_6H_5$, $^3J_{HH}$ = 8.3), 6.95 (d, 1H, $OC_6H_5$, $^3J_{HH}$ = 7.2), 6.95 (s, 1H, OH), 7.09 (m, 5H, $OC_6H_5$,

NHC$_6\underline{H}_4$CH$_3$), 7.19 (m, 3H, OC$_6$H$_5$), 7.25-7.37 (m, 7H, OC$_6$H$_5$, NHC$_6\underline{H}_4$CH$_3$, CH$_{m\text{-Ph}}$), 7.40 (s, 2H, C$\underline{H}$CC(CH$_3$)$_3$), 7.80 (d, 1H, CH$_{m\text{-Ph}}$, $^3J_{HH}$ = 9.4), 7.83 (s, 1H, CH$_{m\text{-Ph}}$), 8.23 (s, 1H, NH), 8.49 (s, 1H, NH), 8.71 (s, 1H, NH), 8.75 (s, 1H, NH). $^{13}$C{$^1$H} NMR (DMSO-*d$_6$*, 100.57 MHz), *δ*, ppm: 21.33 (CH$_3$), 31.21 [C($\underline{C}$H$_3$)$_3$], 35.53 [$\underline{C}$(CH$_3$)$_3$], 44.22 (d, CHP, $^1J_{PC}$ = 140.8), 114.95 (CH$_{m\text{-Ph}}$); 115.38 (CH$_{m\text{-Ph}}$), 119.18 (NH$\underline{C}_6$H$_4$CH$_3$), 119.30 (NH$\underline{C}_6$H$_4$CH$_3$), 121.13 (d, OC$_6$H$_5$, $^3J_{PC}$ = 4.0), 121.44 (d, OC$_6$H$_5$, $^3J_{PC}$ = 4.0), 123.06 (C$_{m\text{-Ph}}$), 125.89 (OC$_6$H$_5$), 126.17 (OC$_6$H$_5$), 126.77 (d, $\underline{C}$CHP, $^3J_{PC}$ = 4.0), 127.01 [$\underline{C}$HCC(CH$_3$)$_2$], 127.10 [$\underline{C}$HCC(CH$_3$)$_2$], 130.16 (NH$\underline{C}_6$H$_4$CH$_3$), 130.21 (d, CH$_{m\text{-Ph}}$, $^3J_{PC}$ = 5.1), 130.44 (OC$_6$H$_5$), 130.75 (OC$_6$H$_5$), 131.59 (NH$\underline{C}_6$H$_4$CH$_3$), 131.68 (NH$\underline{C}_6$H$_4$CH$_3$), 138.03 (NH$\underline{C}_6$H$_4$CH$_3$), 138.05 (d, C$_{m\text{-Ph}}$, $^3J_{PC}$ = 12.1), 138.23 (NH$\underline{C}_6$H$_4$CH$_3$), 140.14 (C$_{m\text{-Ph}}$), 140.24 [$\underline{C}$C(CH$_3$)$_3$], 150.96 (d, OC$_6$H$_5$, $^3J_{PC}$ = 10.1), 151.29 (d, OC$_6$H$_5$, $^3J_{PC}$ = 10.1), 153.43 (C=O), 153.94 (C=O), 154.22 (COH). $^{31}$P NMR (DMSO-*d$_6$*, 161.90 MHz), *δ*, ppm: 20.26. Anal. Calcd for $C_{49}H_{53}N_4O_6P$ (%): C, 71.34; H, 6.48; N, 6.79; P, 3.75. Found (%): C, 71.37; H, 6.48; N, 6.81; P, 3.76. HRMS (MALDI-TOF) m/z for $C_{49}H_{53}N_4O_6P$: calc. 824.4 [M]$^+$, found: 825.7 [M+H]$^+$, 863.8 [M+K]$^+$.

**Dimethyl((2,4-bis-(3-(4-methoxyphenyl)ureido)phenyl)(3,5-di-*tert*-butyl-4-hydroxyphenyl)methyl)phosphonate 15a.** White solid, yield 88%, M.p.: 234-235°C. IR (KBr), *ν*, cm$^{-1}$: 830; 1034; 1123; 1241; 1555; 1605; 1640, 2954; 3324; 3620. $^1$H NMR (DMSO-*d$_6$*, 400.13 MHz), *δ*, ppm: 1.33 [s, 18H, C(CH$_3$)$_3$], 3.49 (d, 3H, OCH$_3$, $^3J_{PH}$ = 10.8), 3.51 (d, 3H, OCH$_3$, $^3J_{PH}$ = 10.8), 3.71 (s, 3H, OCH$_3$), 3.72 (s, 3H, OCH$_3$), 4.74 (d, 1H, CHP, $^2J_{PH}$ = 26.5), 6.86 (dd, 4H, NHC$_6\underline{H}_4$OCH$_3$,$^3J_{HH}$ = 8.6), 7.24 (dd, 1H, CH$_{m\text{-Ph}}$, $^3J_{HH}$ = 8.6, 2.0), 7.30 (s, 2H, C$\underline{H}$CC(CH$_3$)$_3$), 7.35 (dd, 4H, NHC$_6\underline{H}_4$OCH$_3$, $^3J_{HH}$ = 8.8, 6.5), 7.63 (d, 1H, CH$_{m\text{-Ph}}$, $^3J_{HH}$ = 9.1), 7.74 (s, 1H, CH$_{m\text{-Ph}}$), 8.08 (s, 1H, NH), 8.37 (s, 1H, NH), 8.61 (s, 1H, NH), 8.66 (s, 1H, NH). $^{13}$C{$^1$H} NMR (DMSO-*d$_6$*, 100.57 MHz), *δ*, ppm: 31.35 [C($\underline{C}$H$_3$)$_3$], 35.54 [$\underline{C}$(CH$_3$)$_3$], 43.13 (d, CHP, $^1J_{PC}$ = 139.4), 56.17 (OCH$_3$), 53.86 (d, POCH$_3$, $^2J_{PC}$ = 7.1), 53.93 (d, POCH$_3$, $^2J_{PC}$ = 7.1), 114.70 (CH$_{m\text{-Ph}}$), 114.99 (NH$\underline{C}_6$H$_4$OCH$_3$), 115.14 (CH$_{m\text{-Ph}}$), 120.93 (NH$\underline{C}_6$H$_4$OCH$_3$), 121.00 (NH$\underline{C}_6$H$_4$OCH$_3$), 123.71 (C$_{m\text{-Ph}}$), 126.49 [$\underline{C}$HCC(CH$_3$)$_3$], 126.57 [$\underline{C}$HCC(CH$_3$)$_3$], 128.35 (d, $\underline{C}$CHP, $^3J_{PC}$ = 3.0), 130.32 (CH$_{m\text{-Ph}}$), 133.97 (NH$\underline{C}_6$H$_4$OCH$_3$), 133.62 (NH$\underline{C}_6$H$_4$OCH$_3$), 133.80 (d, C$_{m\text{-Ph}}$, $^3J_{PC}$ = 9.1), 139.78 (C$_{m\text{-Ph}}$), 139.98 [$\underline{C}$C(CH$_3$)$_3$], 153.60 (C=O), 153.73 (C=O), 155.36 (NH$\underline{C}_6$H$_4$OCH$_3$), 155.39 (NH$\underline{C}_6$H$_4$OCH$_3$), 155.48 (s, COH). $^{31}$P NMR (DMSO-*d$_6$*, 161.90 MHz), *δ*, ppm: 28.81. HRMS (MALDI-TOF) m/z for $C_{39}H_{49}N_4O_8P$: calc. 723.3 [M]$^+$, found: 755.4 [M+Na]$^+$.

**Diethyl((2,4-bis-(3-(4-methoxyphenyl)ureido)phenyl)(3,5-di-*tert*-butyl-4-hydroxyphenyl)methyl)phosphonate 15b.** White solid, yield 90%, M.p.: 239-240°C. IR (KBr), *ν*, cm$^{-1}$: 829; 1030; 1179; 1553; 1606; 1640, 2957; 3321; 3627. $^1$H NMR (DMSO-*d$_6$*, 600.13 MHz), *δ*, ppm: 1.01 (t, 3H, OCH$_2$C$\underline{H}_3$, $^3J_{HH}$ = 6.8), 1.07 (t, 3H, OCH$_2$C$\underline{H}_3$, $^3J_{HH}$ = 7.1), 1.33 [s, 18H, CH(C$\underline{H}_3$)$_2$], 3.71 (s, 3H, OCH$_3$), 3.72 (s, 3H, OCH$_3$), 3.77 (m, 1H, OC$\underline{H}_2$CH$_3$), 3.83 (m, 1H, OC$\underline{H}_2$CH$_3$), 3.90 (m, 2H, OC$\underline{H}_2$CH$_3$), 4.68 (d, 1H, CHP, $^2J_{PH}$ = 27.4), 6.85 (s, 1H, OH), 6.86 (t, 4H, NHC$_6\underline{H}_4$OCH$_3$, $^3J_{HH}$ = 8.4), 7.24 (d, 1H, CH$_{m\text{-Ph}}$, $^3J_{HH}$ = 8.0), 7.30 (s, 2H, C$\underline{H}$CC(CH$_3$)$_3$), 7.35 (t, 4H, NHC$_6\underline{H}_4$OCH$_3$, $^3J_{HH}$ = 8.4), 7.64 (d, 1H, CH$_{m\text{-Ph}}$, $^3J_{HH}$ = 7.7), 7.76 (s, 1H, CH$_{m\text{-Ph}}$), 8.08 (s, 1H, N$\underline{H}$), 8.36 (s, 1H, N$\underline{H}$), 8.59 (s, 1H, N$\underline{H}$), 8.69 (s, 1H, N$\underline{H}$). ). $^{13}$C{$^1$H} NMR (DMSO-*d$_6$*, 100.57 MHz), *δ*, ppm: 16.44 (d, OCH$_2\underline{C}$H$_3$, $^3J_{PC}$ = 5.1), 16.59 (d, OCH$_2\underline{C}$H$_3$, $^3J_{PC}$ = 5.1), 30.81 [C($\underline{C}$H$_3$)$_3$], 35.01 [$\underline{C}$(CH$_3$)$_3$], 43.13 (d, CHP, $^1J_{PC}$ = 141.4), 55.64 (OCH$_3$), 62.27 (d, O$\underline{C}$H$_2$CH$_3$, $^2J_{PC}$ = 7.1), 62.51 (d, O$\underline{C}$H$_2$CH$_3$, $^2J_{PC}$ = 7.1), 113.88 (CH$_{m\text{-Ph}}$), 114.20 (CH$_{m\text{-Ph}}$), 114.47 (NH$\underline{C}_6$H$_4$OCH$_3$), 120.22 (NH$\underline{C}_6$H$_4$OCH$_3$), 120.47 (NH$\underline{C}_6$H$_4$OCH$_3$), 123.04 (C$_{m\text{-Ph}}$), 126.10 [$\underline{C}$HCC(CH$_3$)$_3$], 126.18 [$\underline{C}$HCC(CH$_3$)$_3$], 127.99 ($\underline{C}$CHP), 129.75 (CH$_{m\text{-Ph}}$), 137.41 (d, C$_{m\text{-Ph}}$, $^3J_{PC}$ = 11.1), 139.16 (C$_{m\text{-Ph}}$), 139.38 [$\underline{C}$C(CH$_3$)$_3$], 140.12 (NH$\underline{C}_6$H$_4$OCH$_3$), 140.35 (NH$\underline{C}_6$H$_4$OCH$_3$), 153.08 (C=O), 153.15 (C=O), 153.45 (s, COH), 154.86 (NH$\underline{C}_6$H$_4$OCH$_3$), 154.96 (NH$\underline{C}_6$H$_4$OCH$_3$). $^{31}$P NMR

(DMSO-$d_6$, 242.94 MHz), $\delta$, ppm: 26.78. HRMS (MALDI-TOF) m/z for $C_{41}H_{53}N_4O_8P$: calc. 760.4 $[M]^+$, found: 783.4 $[M+Na]^+$.

**Diphenyl((2,4-bis-(3-(4-methoxyphenyl)ureido)phenyl)(3,5-di-*tert*-butyl-4-hydroxyphenyl)methyl)phosphonate 15d.** White solid, yield 88%, M.p.: 195-196°C. IR (KBr), $\nu$, $cm^{-1}$: 829; 1183; 1221; 1242; 1554; 1607, 2955; 3323; 3620. $^1H$ NMR (DMSO-$d_6$, 400.13 MHz), $\delta$, ppm: 1.30 [s, 18H, $C(CH_3)_3$], 3.72 (s, 3H, $OCH_3$), 3.73 (s, 3H, $OCH_3$), 5.20 (d, 1H, CHP, $^2J_{PH}$ = 27.5), 6.64 (d, 2H, $OC_6H_5$, $^3J_{HH}$ = 8.1), 6.87 (dd, 4H, $OC_6H_5$, $^3J_{HH}$ = 8.8, 6.0), 6.95 (s, 1H, OH), 6.96 (d, 1H, $OC_6H_5$, $^3J_{HH}$ = 8.1), 7.25-7.45 (m, 7H, $OC_6H_5$, $NHC_6\underline{H}_4OCH_3$, $CH_{m\text{-}Ph}$), 7.40 (s, 2H, $C\underline{H}CC(CH_3)_3$), 7.80 (dd, 1H, $CH_{m\text{-}Ph}$, $^3J_{HH}$ = 8.0), 7.81 (s, 1H, $CH_{m\text{-}Ph}$), 8.19 (s, 1H, NH), 8.41 (s, 1H, NH), 8.67 (s, 2H, NH). $^{13}C\{^1H\}$ NMR (DMSO-$d_6$, 100.57 MHz), $\delta$, ppm: 31.22 [$C(\underline{C}H_3)_3$], 35.54 [$\underline{C}(CH_3)_3$], 44.23 (d, CHP, $^1J_{PC}$ = 141.1), 56.18 ($OCH_3$), 114.88 ($CH_{m\text{-}Ph}$); 115.02 ($NHC_6\underline{H}_4OCH_3$), 115.36 ($CH_{m\text{-}Ph}$), 120.84 ($NHC_6\underline{H}_4OCH_3$), 120.05 ($NHC_6\underline{H}_4OCH_3$), 121.15 (d, $OC_6H_5$, $^3J_{PC}$ = 4.0), 121.46 (d, $OC_6H_5$, $^3J_{PC}$ = 3.0), 122.94 (d, $C_{m\text{-}Ph}$, $^3J_{PC}$ = 1.0), 125.90 ($OC_6H_5$), 126.18 ($OC_6H_5$), 126.81 (d, $\underline{C}CHP$, $^3J_{PC}$ = 4.0), 127.03 [$\underline{C}HCC(CH_3)_2$], 127.12 [$\underline{C}HCC(CH_3)_2$], 130.19 (d, $CH_{m\text{-}Ph}$, $^3J_{PC}$ = 4.0), 130.46 ($OC_6H_5$), 130.76 ($OC_6H_5$), 133.66 ($NHC_6\underline{H}_4OCH_3$), 133.89 ($NHC_6\underline{H}_4OCH_3$), 138.15 (d, $C_{m\text{-}Ph}$, $^3J_{PC}$ = 12.1), 140.23 ($C_{m\text{-}Ph}$), 140.25 [$\underline{C}C(CH_3)_3$], 150.98 (d, $OC_6H_5$, $^3J_{PC}$ = 10.1), 151.33 (d, $OC_6H_5$, $^3J_{PC}$ = 10.1), 153.61 (C=O), 154.11 (C=O), 154.25 (COH). 155.46 ($NHC_6\underline{H}_4OCH_3$), 155.54 ($NHC_6\underline{H}_4OCH_3$). $^{31}P$ NMR (DMSO-$d_6$, 161.90 MHz), $\delta$, ppm: 20.29. HRMS (MALDI-TOF) m/z for $C_{49}H_{53}N_4O_8P$: calc. 856.4 $[M]^+$, found: 856.6 $[M+H]^+$; 879.6 $[M+Na]^+$.

**Dimethyl((2,4-bis-(3-(*p*-chlorophenyl)ureido)phenyl)(3,5-di-*tert*-butyl-4-hydroxyphenyl)methyl)phosphonate 16a.** White solid, yield 85%, M.p.: 249-250°C. IR (KBr), $\nu$, $cm^{-1}$: 829; 1039; 1226; 1550; 1697, 1639; 2955; 3323; 3631. $^1H$ NMR (DMSO-$d_6$, 600.13 MHz), $\delta$, ppm: 1.32 [s, 18H, $C(CH_3)_3$], 3.51 (t, 6H, $OCH_3$, $^3J_{PH}$ =10.1), 4.74 (d, 1H, CHP, $^2J_{PH}$ = 26.3), 6.87 (s, 1H, OH), 7.25 (dd, 1H, $CH_{m\text{-}Ph}$, $^3J_{HH}$ = 8.6, $^4J_{HH}$ = 2.3), 7.29 (s, 2H, $C\underline{H}CC(CH_3)_3$), 7.31 (d, 2H, $NHC_6\underline{H}_4Cl$, $^3J_{HH}$ = 3.5), 7.33 (d, 2H, $NHC_6\underline{H}_4Cl$, $^3J_{HH}$ = 3.4), 7.47 (d, 2H, $NHC_6\underline{H}_4Cl$, $^3J_{HH}$ = 3.9), 7.49 (d, 2H, $NHC_6\underline{H}_4Cl$, $^3J_{HH}$ = 3.7), 7.65 (dd, 1H, $CH_{m\text{-}Ph}$, $^3J_{HH}$ = 8.6), 7.74 (s, 1H, $CH_{m\text{-}Ph}$), 8.24 (s, 1H, NH), 8.73 (s, 1H, NH), 8.75 (s, 1H, NH), 8.99 (s, 1H, NH). $^{13}C\{^1H\}$ NMR (DMSO-$d_6$, 100.57 MHz), $\delta$, ppm: 31.33 [$C(\underline{C}H_3)_3$], 35.53 [$\underline{C}(CH_3)_3$], 43.21 (d, CHP, $^1J_{PC}$ = 139.4), 53.87 (d, $POCH_3$, $^2J_{PC}$ = 7.1), 53.97 (d, $POCH_3$, $^2J_{PC}$ = 7.1), 115.33 ($CH_{m\text{-}Ph}$), 115.83 ($CH_{m\text{-}Ph}$), 120.55 ($NH\underline{C}_6H_4Cl$), 120.71 ($NH\underline{C}_6H_4Cl$), 124.59 (d, $C_{m\text{-}Ph}$, $^3J_{PC}$ = 3.0), 126.24 ($NH\underline{C}_6H_4Cl$), 126.37 ($NH\underline{C}_6H_4Cl$), 126.48 [$\underline{C}HCC(CH_3)_3$], 126.56 [$\underline{C}HCC(CH_3)_3$], 128.24 (d, $\underline{C}CHP$, $^3J_{PC}$ = 4.0), 129.58 ($NH\underline{C}_6H_4Cl$), 130.39 (d, $CH_{m\text{-}Ph}$, $^3J_{PC}$ = 6.1), 137.46 (d, $C_{m\text{-}Ph}$, $^3J_{PC}$ = 11.1), 139.45 ($C_{m\text{-}Ph}$), 139.64 ($NH\underline{C}_6H_4Cl$), 139.88 ($NH\underline{C}_6H_4Cl$), 140.00 [$\underline{C}C(CH_3)_3$], 153.29 (C=O), 153.76 (s, COH), 153.82 (C=O). $^{31}P$ NMR (DMSO-$d_6$, 242.94 MHz), $\delta$, ppm: 28.73. Anal. Calcd for $C_{37}H_{43}Cl_2N_4O_6P$ (%): C, 59.92; H, 5.84; Cl, 9.56; N, 7.55; P, 4.18. Found (%): C, 59.94; H, 5.87; Cl, 9.58; N, 7.57; P, 4.20. HRMS (MALDI-TOF) m/z for $C_{37}H_{43}Cl_2N_4O_6P$: calc. 740.2 $[M]^+$, found: 780.4 $[M+K]^+$.

**Diethyl((2,4-bis-(3-(*p*-chlorophenyl)ureido)phenyl)(3,5-di-*tert*-butyl-4-hydroxyphenyl)methyl)phosphonate 16b.** White solid, yield 75%, M.p.: 240-241°C. IR (KBr), $\nu$, $cm^{-1}$: 828; 1026; 1055; 1221; 1548; 1698, 1641; 2962; 3317; 3631. $^1H$ NMR (DMSO-$d_6$, 400.13 MHz), $\delta$, ppm: 1.00 (t, 3H, $OCH_2C\underline{H}_3$, $^3J_{HH}$ = 7.0), 1.06 (t, 3H, $OCH_2C\underline{H}_3$, $^3J_{HH}$ = 7.0), 1.31 [s, 18H, $CH(C\underline{H}_3)_2$], 3.77 (m, 1H, $OC\underline{H}_2CH_3$), 3.83 (m, 1H, $OC\underline{H}_2CH_3$), 3.90 (m, 2H, $OC\underline{H}_2CH_3$), 4.68 (d, 1H, CHP, $^2J_{PH}$ = 26.5), 6.84 (s, 1H, OH), 7.24 (s, 1H, $CH_{m\text{-}Ph}$, $^3J_{HH}$ = 8.6, 2.1), 7.29 (s, 2H, $C\underline{H}CC(CH_3)_3$), 7.30 (d, 2H, $NHC_6\underline{H}_4Cl$, $^3J_{HH}$ = 3.6), 7.33 (d, 2H, $NHC_6\underline{H}_4Cl$, $^3J_{HH}$ = 3.6), 7.47 (d, 2H, $NHC_6\underline{H}_4Cl$, $^3J_{HH}$ = 3.3), 7.49 (d, 2H, $NHC_6\underline{H}_4Cl$, $^3J_{HH}$ = 3.3), 7.66 (d, 1H, $CH_{m\text{-}Ph}$, $^3J_{HH}$ = 8.6), 7.76 (s, 1H, $CH_{m\text{-}Ph}$), 8.23 (s, 1H, $N\underline{H}$), 8.72 (s, 1H, $N\underline{H}$), 8.74 (s, 1H, $N\underline{H}$), 9.01 (s, 1H, $N\underline{H}$). $^{13}C\{^1H\}$ NMR (DMSO-$d_6$, 100.57 MHz), $\delta$, ppm:

16.43 (d, $OCH_2\underline{C}H_3$, $^3J_{PC}$ = 6.1), 16.58 (d, $OCH_2\underline{C}H_3$, $^3J_{PC}$ = 6.1), 30.78 [$C(\underline{C}H_3)_3$], 35.00 [$\underline{C}(CH_3)_3$], 43.28 (d, CHP, $^1J_{PC}$ = 139.4), 62.29 (d, $O\underline{C}H_2CH_3$, $^2J_{PC}$ = 7.1), 62.56 (d, $O\underline{C}H_2CH_3$, $^2J_{PC}$ = 7.1), 114.51 ($CH_{m\text{-}Ph}$), 114.90 ($CH_{m\text{-}Ph}$), 119.99 ($NHC_6H_5Cl$), 120.18 ($NH\underline{C}_6H_4Cl$), 123.92 ($C_{m\text{-}Ph}$), 125.69 ($NH\underline{C}_6H_4Cl$), 125.82 ($NH\underline{C}_6H_4Cl$), 126.08 [$\underline{C}HCC(CH_3)_3$], 126.16 [$\underline{C}HCC(CH_3)_3$], 127.86 (d, $\underline{C}CHP$, $^2J_{PC}$ = 4.0), 129.06 ($NH\underline{C}_6H_4Cl$), 129.87 (d, $CH_{m\text{-}Ph}$, $^2J_{PC}$ = 6.1), 137.04 (d, $C_{m\text{-}Ph}$, $^3J_{PC}$ = 11.1), 138.81 ($C_{m\text{-}Ph}$), 139.13 ($NH\underline{C}_6H_4Cl$), 139.39 ($NH\underline{C}_6H_4Cl$), 139.39 [$\underline{C}C(CH_3)_3$], 152.77 (C=O), 153.16 (C=O), 153.21 (COH). $^{31}$P NMR (DMSO-$d_6$, 242.94 MHz), $\delta$, ppm: 26.60. Anal. Calcd for $C_{39}H_{47}Cl_2N_4O_6P$ (%): C, 60.86; H, 6.15; Cl, 9.21; N, 7.28; P, 4.02. Found (%): C, 60.88; H, 6.17; Cl, 9.24; N, 7.30; P, 4.05. HRMS (MALDI-TOF) m/z for $C_{39}H_{47}Cl_2N_4O_6P$: calc. 768.3 $[M]^+$, found: 769.4 $[M+H]^+$, 791.2 $[M+Na]^+$.

**Diphenyl((2,4-bis-(3-(*p*-chlorophenyl)ureido)phenyl)(3,5-di-*tert*-butyl-4-hydroxyphenyl)methyl)phosphonate 16d.** White solid, yield 83%, M.p.: 220-221°C. IR (KBr), $\nu$, cm$^{-1}$: 826; 1011; 1026; 1207; 1547; 1696, 1641; 2959; 3310; 3629. $^1$H NMR (DMSO-$d_6$, 400.13 MHz), $\delta$, ppm: 1.28 [s, 18H, $C(CH_3)_3$], 5.20 (d, 1H, CHP, $^2J_{PH}$ = 27.4), 6.63 (d, 2H, $OC_6H_5$, $^3J_{HH}$ = 8.0), 6.94 (s, 1H, OH), 6.95 (d, 1H, $CH_{m\text{-}Ph}$, $^3J_{HH}$ = 8.4), 7.09 (t, 1H, $OC_6H_5$, $^3J_{HH}$ = 7.6), 7.18 (m, 3H, $OC_6H_5$), 7.31 (m, 9H, $OC_6H_5$, $C\underline{H}CC(CH_3)_3$, $NHC_6\underline{H}_4Cl$, $CH_{m\text{-}Ph}$), 7.48 (m, 4H, $NHC_6\underline{H}_4Cl$), 7.81 (s, 1H, $CH_{m\text{-}Ph}$), 7.82 (d, 1H, $CH_{m\text{-}Ph}$, $^3J_{HH}$ = 9.4), 8.34 (s, 1H, NH), 8.77 (s, 1H, NH), 8.81 (s, 1H, NH), 9.00 (s, 1H, NH). $^{13}C\{^1H\}$ NMR (DMSO-$d_6$, 100.57 MHz), $\delta$, ppm: 31.19 [$C(\underline{C}H_3)_3$], 35.52 [$\underline{C}(CH_3)_3$], 44.33 (d, CHP, $^1J_{PC}$ = 142.4), 115.53 ($CH_{m\text{-}Ph}$), 116.08 ($CH_{m\text{-}Ph}$), 120.58 ($NH\underline{C}_6H_4Cl$), 120.75 ($NH\underline{C}_6H_4Cl$), 120.05 ($NH\underline{C}_6H_4Cl$), 121.12 (d, $OC_6H_5$, $^3J_{PC}$ = 4.0), 121.43 (d, $OC_6H_5$, $^3J_{PC}$ = 3.0), 123.86 ($C_{m\text{-}Ph}$), 125.91 ($OC_6H_5$), 126.18 ($OC_6H_5$), 126.30 ($NH\underline{C}_6H_4Cl$), 126.42 ($NH\underline{C}_6H_4Cl$), 126.69 (d, $\underline{C}CHP$, $^3J_{PC}$ = 4.0), 127.00 [$\underline{C}HCC(CH_3)_2$], 127.08 [$\underline{C}HCC(CH_3)_2$], 130.23 (d, $CH_{m\text{-}Ph}$, $^3J_{PC}$ = 4.0), 129.59 ($NH\underline{C}_6H_4Cl$), 130.45 ($OC_6H_5$), 130.75 ($OC_6H_5$), 137.75 (d, $C_{m\text{-}Ph}$, $^3J_{PC}$ = 12.1), 139.61 ($NH\underline{C}_6H_4Cl$), 139.83 ($NH\underline{C}_6H_4Cl$), 139.89 ($C_{m\text{-}Ph}$), 140.24 [$\underline{C}C(CH_3)_3$], 150.94 (d, $OC_6H_5$, $^3J_{PC}$ = 11.1), 150.27 (d, $OC_6H_5$, $^3J_{PC}$ = 9.1), 153.29 (C=O), 153.87 (C=O), 154.24 (COH). $^{31}$P NMR (DMSO-$d_6$, 161.90 MHz), $\delta$, ppm: 20.17. Anal. Calcd for $C_{47}H_{47}Cl_2N_4O_6P$ (%): C, 65.20; H, 5.47; Cl, 8.19; N, 6.47; P, 3.58. Found (%): C, 65.23; H, 5.49; Cl, 8.23; N, 6.50; P, 3.60. HRMS (MALDI-TOF) m/z for $C_{47}H_{47}Cl_2N_4O_6P$: calc. 864.3 $[M]^+$, found: 865.6 $[M+H]^+$; 903.6 $[M+K]^+$.

**Dimethyl((2,6-bis-(3-(*p*-nitrophenyl)ureido)pyridin-3-yl)(3,5-di-*tert*-butyl-4-hydroxyphenyl)methyl)phosphonate 17a.** Yellow solid, yield 83%, M.p.: 266-267°C. IR (KBr), $\nu$, cm$^{-1}$: 848; 1037; 1213; 1541; 1594, 1718; 2958; 3335; 3601. $^1$H NMR (DMSO-$d_6$, 400.13 MHz), $\delta$, ppm: 1.31 [s, 18H, $C(CH_3)_3$], 3.52 (t, 6H, $OCH_3$, $^3J_{PH}$ = 10.2), 4.77 (d, 1H, CHP, $^2J_{PH}$ = 26.3), 6.88 (s, 1H, OH), 7.29 (s, 2H, $C\underline{H}CC(CH_3)_3$), 7.31 (d, 1H, $CH_{m\text{-}Ph}$, $^4J_{HH}$ = 2.2), 7.31 (d, 2H, $NHC_6\underline{H}_4NO_2$, $^3J_{HH}$ = 3.5), 7.70 (m, 5H, $NHC_6\underline{H}_4NO_2$, $CH_{m\text{-}Ph}$), 7.80 (s, 1H, $CH_{m\text{-}Ph}$), 8.19 (d, 2H, $NHC_6\underline{H}_4NO_2$, $^3J_{HH}$ = 5.2), 8.21 (d, 2H, $NHC_6\underline{H}_4NO_2$, $^3J_{HH}$ = 5.2), 8.49 (s, 1H, NH), 9.00 (s, 1H, NH), 9.37 (s, 1H, NH), 9.61 (s, 1H, NH). $^{13}C\{^1H\}$ NMR (DMSO-$d_6$, 100.57 MHz), $\delta$, ppm: 31.29 [$C(\underline{C}H_3)_3$], 35.52 [$\underline{C}(CH_3)_3$], 43.39 (d, CHP, $^1J_{PC}$ = 139.4), 53.91 (d, $POCH_3$, $^2J_{PC}$ = 7.1), 54.06 (d, $POCH_3$, $^2J_{PC}$ = 7.1), 116.15 ($CH_{m\text{-}Ph}$); 116.60 ($CH_{m\text{-}Ph}$), 118.41 ($NH\underline{C}_6H_4NO_2$), 118.51 ($NH\underline{C}_6H_4NO_2$), 125.64 (d, $C_{m\text{-}Ph}$, $^2J_{PC}$ = 4.0), 126.11 ($NH\underline{C}_6H_4NO_2$), 126.47 [$\underline{C}HCC(CH_3)_2$], 126.55 [$\underline{C}HCC(CH_3)_2$], 128.10 (d, CCHP, $^2J_{PC}$ = 4.0), 130.58 (d, $CH_{m\text{-}Ph}$, $^3J_{PC}$ = 4.0), 137.03 (d, $C_{m\text{-}Ph}$, $^3J_{PC}$ = 10.1), 139.03 ($C_{m\text{-}Ph}$), 140.04 [$\underline{C}C(CH_3)_3$], 142.00 ($NH\underline{C}_6H_4NO_2$), 142.07 ($NH\underline{C}_6H_4NO_2$), 147.27 ($NH\underline{C}_6H_4NO_2$), 147.52 ($NH\underline{C}_6H_4NO_2$), 152.84 (C=O), 153.45 (C=O), 153.80 (COH). $^{31}$P NMR (DMSO-$d_6$, 161.90 MHz), $\delta$, ppm: 26.83. Anal. Calcd for $C_{37}H_{43}N_6O_{10}P$ (%): C, 58.26; H, 5.68; N, 11.02; P, 4.06. Found (%): C, 58.30; H, 5.70; N, 11.04; P, 4.10. HRMS (MALDI-TOF) m/z for $C_{37}H_{43}N_6O_{10}P$: calc. 762.3 $[M]^+$, found: 785.5 $[M+Na]^+$.

**Diethyl((2,4-bis-(3-(*p*-nitrophenyl)ureido)phenyl)(3,5-di-*tert*-butyl-4-hydroxyphenyl)methyl)phosphonate 17b.** Yellow solid, yield 91%, M.p.: 264-265°C. IR (KBr), $\nu$,

$cm^{-1}$: 849; 1025; 1052; 1208; 1541; 1594, 1723; 2958; 3327; 3630. $^1$H NMR (DMSO-$d_6$, 400.13 MHz), $\delta$, ppm: 1.01 (t, 3H, $OCH_2C\underline{H}_3$, $^3J_{HH}$ = 7.0), 1.07 (t, 3H, $OCH_2C\underline{H}_3$, $^3J_{HH}$ = 7.0), 1.31 [s, 18H, $CH(C\underline{H}_3)_2$], 3.77 (m, 1H, $OC\underline{H}_2CH_3$), 3.83 (m, 1H, $OC\underline{H}_2CH_3$), 3.90 (m, 2H, $OC\underline{H}_2CH_3$), 4.70 (d, 1H, CHP, $^2J_{PH}$ = 26.5), 6.85 (s, 1H, OH), 7.27 (d, 1H, $CH_{m\text{-}Ph,}$ $^3J_{HH}$ = 8.6, 2.1), 7.29 (s, 2H, $C\underline{H}CC(CH_3)_3$, 7.70 (m, 5H, $NHC_6\underline{H}_4NO_2$, $CH_{m\text{-}Ph}$), 7.82 (s, 1H, $CH_{m\text{-}Ph}$), 8.19 (dd, 4H, $NHC_6\underline{H}_4NO_2$, $^3J_{HH}$ = 9.0, 5.2), 8.49 (s, 1H, NH), 8.99 (s, 1H, NH), 9.35 (s, 1H, NH), 9.62 (s, 1H, NH). $^{13}C\{^1H\}$ NMR (DMSO-$d_6$, 100.57 MHz), $\delta$, ppm: 16.30 (d, $OCH_2\underline{C}H_3$, $^3J_{PC}$ = 6.1), 16.50 (d, $OCH_2\underline{C}H_3$, $^3J_{PC}$ = 6.1), 31.17 [$C(\underline{C}H_3)_3$], 35.40 [$\underline{C}(CH_3)_3$], 43.27 (d, CHP, $^1J_{PC}$ = 139.4), 62.39 (d, $O\underline{C}H_2CH_3$, $^2J_{PC}$ = 7.1), 62.72 (d, $O\underline{C}H_2CH_3$, $^2J_{PC}$ = 7.1), 116.03 ($CH_{m\text{-}Ph}$); 116.47 ($CH_{m\text{-}Ph}$), 118.28 ($NH\underline{C}_6H_4NO_2$), 118.38 ($NH\underline{C}_6H_4NO_2$), 125.49 (d, $C_{m\text{-}Ph}$, $^2J_{PC}$ = 4.0), 125.98 ($NH\underline{C}_6H_4NO_2$), 126.34 [$\underline{C}HCC(CH_3)_2$], 126.42 [$\underline{C}HCC(CH_3)_2$], 127.99 (d, CCHP, $^2J_{PC}$ = 4.0), 130.47 (d, $CH_{m\text{-}Ph}$, $^3J_{PC}$ = 4.0), 136.90 (d, $C_{m\text{-}Ph}$, $^3J_{PC}$ = 10.1), 138.90 ($C_{m\text{-}Ph}$), 139.91 [$\underline{C}C(CH_3)_3$], 141.87 ($NH\underline{C}_6H_4NO_2$), 141.97 ($NH\underline{C}_6H_4NO_2$), 147.14 ($NH\underline{C}_6H_4NO_2$), 147.39 ($NH\underline{C}_6H_4NO_2$), 152.71 (C=O), 153.32 (C=O), 153.67 (COH). $^{31}$P NMR (DMSO-$d_6$, 161.90 MHz), $\delta$, ppm: 26.50. Anal. Calcd for $C_{39}H_{47}N_6O_{10}P$ (%): C, 59.23; H, 5.99; N, 10.63; P, 3.92. Found (%): C, 59.26; H, 6.00; N, 10.67; P, 3.95. HRMS (MALDI-TOF) m/z for $C_{39}H_{47}N_6O_{10}P$: calc. 790.3 $[M]^+$, found: 813.5 $[M+Na]^+$.

**Diphenyl((2,4-bis-(3-(*p*-nitrophenyl)ureido)phenyl)(3,5-di-*tert*-butyl-4-hydroxyphenyl)methyl)phosphonate 17d.** Yellow solid, yield 80%, M.p.: 221-222°C. IR (KBr), $\nu$, $cm^{-1}$: 851; 1025; 1210; 1543; 1679, 1719; 2962; 3317; 3631. $^1$H NMR (DMSO-$d_6$, 400.13 MHz), $\delta$, ppm: 1.28 [s, 18H, $C(CH_3)_3$], 5.22 (d, 1H, CHP, $^2J_{PH}$ = 27.2), 6.64 (d, 2H, $OC_6H_5$, $^3J_{HH}$ = 8.0), 6.95 (s, 1H, OH), 6.96 (d, 1H, $CH_{m\text{-}Ph}$, $^3J_{HH}$ = 8.4), 7.10 (t, 1H, $OC_6H_5$, $^3J_{HH}$ = 7.2), 7.19 (m, 3H, $OC_6H_5$), 7.32 (m, 3H, $OC_6H_5$, $CH_{m\text{-}Ph}$), 7.38 (s, 2H, $C\underline{H}CC(CH_3)_3$, 7.71 (dd, 4H, $NHC_6\underline{H}_4NO_2$, $^3J_{HH}$ = 9.4, 2.2), 7.86 (s, 1H, $CH_{m\text{-}Ph}$), 8.20 (dd, 4H, $NHC_6\underline{H}_4NO_2$, $^3J_{HH}$ = 9.1, 5.8), 8.57 (s, 1H, NH), 9.06 (s, 1H, NH), 9.40 (s, 1H, NH), 9.59 (s, 1H, NH). $^{13}C\{^1H\}$ NMR (DMSO-$d_6$, 100.57 MHz), $\delta$, ppm: 31.16 [$C(\underline{C}H_3)_3$], 35.51 [$\underline{C}(CH_3)_3$], 44.44 (d, CHP, $^1J_{PC}$ = 140.4), 53.91 (d, $POCH_3$, $^2J_{PC}$ = 7.1), 54.06 (d, $POCH_3$, $^2J_{PC}$ = 7.1), 116.36 ($CH_{m\text{-}Ph}$); 116.84 ($CH_{m\text{-}Ph}$), 118.42 ($NH\underline{C}_6H_4NO_2$), 118.53 ($NH\underline{C}_6H_4NO_2$), 121.10 (d, $OC_6H_5$, $^3J_{PC}$ = 4.0), 121.41 (d, $OC_6H_5$, $^3J_{PC}$ = 4.0), 124.91 (d, $C_{m\text{-}Ph}$, $^2J_{PC}$ = 4.0), 125.94 ($OC_6H_5$), 126.11 ($NH\underline{C}_6H_4NO_2$), 126.21 ($OC_6H_5$), 126.56 (d, CCHP, $^2J_{PC}$ =5.1), 126.99 [$\underline{C}HCC(CH_3)_2$], 126.07 [$\underline{C}HCC(CH_3)_2$], 130.37 ($CH_{m\text{-}Ph}$), 130.46 ($OC_6H_5$), 130.77 ($OC_6H_5$), 137.30 (d, $C_{m\text{-}Ph}$, $^3J_{PC}$ = 10.1), 139.45 ($C_{m\text{-}Ph}$), 140.27 [$\underline{C}C(CH_3)_3$], 142.04 ($NH\underline{C}_6H_4NO_2$), 142.09 ($NH\underline{C}_6H_4NO_2$), 147.23 ($NH\underline{C}_6H_4NO_2$), 147.44 ($NH\underline{C}_6H_4NO_2$), 151.00 (d, $OC_6H_5$, $^3J_{PC}$ = 10.1), 151.24 (d, $OC_6H_5$, $^3J_{PC}$ = 10.1), 152.83 (C=O), 153.50 (C=O), 154.27 (COH). $^{31}$P NMR (DMSO-$d_6$, 161.90 MHz), $\delta$, ppm: 19.99. Anal. Calcd for $C_{47}H_{47}N_6O_{10}P$ (%): C, 63.65; H, 5.34; N, 9.48; P, 3.49. Found (%): C, 63.67; H, 5.37; N, 9.51; P, 3.51. HRMS (MALDI-TOF) m/z for $C_{47}H_{47}N_6O_{10}P$: calc. 886.3 $[M]^+$, found: 909.4 $[M+K]^+$.

**Dimethyl[2-amino-6-(3-phenylthioureido)pyridin-3-yl)(3,5-di-*tert*-butyl-4-hydroxyphenyl)methyl]phosphonate 18a**. White-gray solid, yield 0.45 g (80%), M.p.: 108–109°C. IR (KBr), $\nu$, $cm^{-1}$: 768, 1032, 1058, 1235, 1357 (C=S), 1599, 3326 (NH), 3433 ($NH_2$), 3628 (OH). $^1$H NMR (500.13 MHz, DMCO-$d_6$), $\delta$, ppm: 1.37, 1.38, 1.39 [all s, 18H, $C(CH_3)_3$], 3.50, 3.56, 3.57, 3.62 (all d, 6H, $^3J_{HH}$ = 10.6 Hz, $OCH_3$), 4.67, 4.92, 4.98 (all d, 1H, $^2J_{PH}$ = 26.7 Hz, CHP), 6.24, 6.44 (all d, 1H, $^3J_{HH}$ = 8.5 Hz, $CH_{py}$), 6.50 (br.s, 2H, $NH_2$), 6.88, 6.91, 6.93 (all s, 1H, OH), 7.06-7.22 (m, 1H, $NHC_6\underline{H}_5$), 7.30, 7.31, 7.32 [all s, 2H, $C\underline{H}CC(CH_3)_3$], 7.34-7.38 (m, 2H, $NHC_6\underline{H}_5$), 7.50, 7.68 (all d, 2H, $^3J_{HH}$ = 9.3 Hz, $NHC_6\underline{H}_5$), 7.87, 8.05 (dd, 1H, $^3J_{HH}$ = 8.2 Hz, $^4J_{PH}$ = 1.4 Hz, $CH_{py}$), 9.43, 9.77, 9.88, 10.32 (all s, 1H, NH), 10.45, 10.83 (all s, 1H, NH). $^{13}C\{^1H\}$ NMR (125.76 MHz, DMCO-$d_6$), $\delta$, ppm: 30.80, 30.87 [$C(\underline{C}H_3)_3$], 35.05, 35.07, 35.12 [$\underline{C}(CH_3)_3$], 42.02, 43.33, 43.46 (all d, $^1J_{PC}$ = 134.6 Hz, CHP), 53.20, 53.45, 53.67, 53.82 (all d, $^2J_{PC}$ = 7.0 Hz, $OCH_3$), 100.05, 103.14 ($CH_{py}$), 110.64 ($C_{py}$), 124.10 ($NHC_6H_5$), 124.40 ($NHC_6H_5$), 124.75 ($NHC_6H_5$), 124.87 ($NHC_6H_5$), 126.38 ($NHC_6H_5$), 126.16, 126.41 (all d, $^3J_{PC}$

= 7.4 Hz, $\underline{C}HCC(CH_3)_3$], 127.26, 128.60 (all d, $^2J_{PC}$ = 4.6 Hz, $\underline{C}CHP$), 128.58, 128.63, 128.77, 128.88 ($NHC_6H_5$), 139.37, 139.46 [$\underline{C}C(CH_3)_3$], 139.59 ($NHC_6H_5$), 139.93 ($NHC_6H_5$), 140.27, 140.75 (all d, $^3J_{PC}$ = 4.8 Hz, $CH_{py}$), 146.75, 155.15 (all d, $^3J_{PC}$ = 11.4 Hz, $C_{py}$), 151.02, 151.39 ($C_{py}$), 153.23 ($NHC_6H_5$), 153.39 (OH), 151.33, 155.20 ($C_{py}$), 180.12, 180.30 (C=S). $^{31}$P NMR (202.46 MHz, DMCO-*d*$_6$), *δ*, ppm: 27.92, 28.47, 28.50. Anal. Calcd for $C_{29}H_{39}N_4O_4PS$ (%): C, 61.03; H, 6.89; N, 9.82; P, 5.43; S, 5.62. Found (%): C, 61.05; H, 6.90; N, 9.84; P, 5.46; S, 5.64. HRMS (MALDI-TOF) *m*/*z* for $C_{29}H_{39}N_4O_4PS$: calc. 570.24 $[M]^+$, found 571.46 $[M+H]^+$, 593.44 $[M+Na]^+$.

**Diethyl[(2-amino-6-(3-phenylthioureido)pyridin-3-yl)(3,5-di-*tert*-butyl-4-hydroxyphenyl)methyl]phosphonate 18b**. White-gray solid, yield 0.45 g (75%), M.p.: 111–112°C. IR (KBr), *ν*, cm$^{-1}$: 772, 1034, 1059, 1237, 1345, 1597, 3322, 3436, 3630. $^1$H NMR (500.13 MHz, DMCO-*d*$_6$), *δ*, ppm: 1.54, 1.57 (all t, 3H, $^3J_{HH}$ = 7.1 Hz, $OCH_2C\underline{H}_3$), 1.62, 1.68 (all t, 3H, $^3J_{HH}$ = 7.1 Hz, $OCH_2C\underline{H}_3$), 1.89, 1.91 [all s, 18H, $C(CH_3)_3$], 4.31 (m, 1H, $OC\underline{H}_2CH_3$), 4.47(m, 1H, $OC\underline{H}_2CH_3$), 4.54 (m, 2H, $OC\underline{H}_2CH_3$), 4.98, 5.00, 5.34 (all d, 1H, $^2J_{PH}$ = 27.0 Hz, CHP), 6.42 (br.s, 2H, $NH_2$), 6.51, 6.55 (all s, 1H, OH), 6.81, 7.01 (all d, 1H, $^3J_{HH}$ = 8.5 Hz, $CH_{py}$), 7.63 (m, 1H, $NHC_6\underline{H}_5$), 7.80 (m, 2H, $NHC_6\underline{H}_5$), 7.89, 7.90 [all d, 2H, $^4J_{PH}$ = 1.5 Hz, $C\underline{H}CC(CH_3)_3$], 8.23 (m, 2H, $NHC_6\underline{H}_5$), 8.43, 8.47 (all d, 1H, $^3J_{HH}$ = 8.1 Hz, $CH_{py}$), 9.43 (s, 1H, NH), 9.92 (s, 1H, NH). $^{13}$C{$^1$H} NMR (125.76 MHz, DMCO-*d*$_6$), *δ*, ppm: 16.19, 16.24 ($OCH_2\underline{C}H_3$), 30.26, 30.30 [$C(\underline{C}H_3)_3$], 34.84, 34.93 [$\underline{C}(CH_3)_3$], 44.20, 45.32 (all d, $^1J_{PC}$ = 138.6 Hz, CHP), 62.39, 62.42 (all d, $^2J_{PC}$ = 6.9 Hz, $O\underline{C}H_2CH_3$), 62.87, 63.11 (all d, $^2J_{PC}$ = 6.9 Hz, $O\underline{C}H_2CH_3$), 100.90, 102.67 ($CH_{py}$), 107.85, 110.79 ($C_{py}$), 124.62 ($NHC_6H_5$), 124.79 ($NHC_6H_5$), 1245.01 ($NHC_6H_5$), 125.14 ($NHC_6H_5$), 125.51 ($NHC_6H_5$), 126.91, 126.94 (all d, $^3J_{PC}$ = 7.6 Hz, $\underline{C}HCC(CH_3)_3$], 126.57, 127.06 (all d, $^2J_{PC}$ = 4.8 Hz, $\underline{C}CHP$), 128.54 ($NHC_6H_5$), 128.58 ($NHC_6H_5$), 137.75, 137.87, 138.09, 138.21 [$\underline{C}C(CH_3)_3$], 139.91, 140.06, 140.15 ($NHC_6H_5$), 141.30, 141.98 (all d, $^3J_{PC}$ = 6.0 Hz, $CH_{py}$), 149.81, 151.58, 151.66 ($C_{py}$), 153.70, 153.80 (COH), 155.60, 156.78 ($C_{py}$), 178.92, 179.12, 179.25, 179.35 (C=S). $^{31}$P NMR (202.46 MHz, DMCO-*d*$_6$), *δ*, ppm: 25.65, 26.07, 26.24. Anal. Calcd for $C_{31}H_{43}N_4O_4PS$ (%): C, 62.19; H, 7.24; N, 9.36; P, 5.17; S, 5.35. Found (%): C, 62.20; H, 7.26; N, 9.38; P, 5.20; S, 5.39. HRMS (MALDI-TOF) *m*/*z* for $C_{31}H_{43}N_4O_4PS$: calc. 598.27 $[M]^+$, found 599.32 $[M+H]^+$, 621.33 $[M+Na]^+$.

**Diisopropyl[(2-amino-6-(3-phenylthioureido)pyridin-3-yl)(3,5-di-*tert*-butyl-4-hydroxyphenyl)methyl]phosphonate 18c**. White-gray solid, yield 0.49 g (78%), M.p.: 125–126°C. IR (KBr), *ν*, cm$^{-1}$: 748, 992, 1236, 1357, 1599, 3302, 3433, 3629. $^1$H NMR (500.13 MHz, DMCO-*d*$_6$), *δ*, ppm: 0.83, 0.87 [all d, 3H, $^3J_{HH}$ = 6.2 Hz, $OCH(C\underline{H}_3)_2$], 1.04, 1.06 [all d, 3H, $^3J_{HH}$ = 6.2 Hz, $OCH(C\underline{H}_3)_2$], 1.16, 1.18 [all d, 6H, $^3J_{HH}$ = 6.1 Hz, $OCH(C\underline{H}_3)_2$], 1.36, 1.37, 1.38 [all s, 18H, $C(CH_3)_3$], 4.42 [all m, 1H, $OC\underline{H}(CH_3)_2$], 4.47 [all m, 1H, $OC\underline{H}(CH_3)_2$], 4.49, 4.67 (all d, 1H, $^2J_{PH}$ = 27.0 Hz, CHP), 6.12, 6.22, 6.33 (all d, 1H, $^3J_{PH}$ = 7.6 Hz, $CH_{py}$), 6.46 (br.s, 2H, $NH_2$), 6.82, 6.87, 6.90 (all s, 1H, OH), 7.06-7.19 (m, 1H, $NHC_6\underline{H}_5$), 7.32-7.37 [m, 4H, $C\underline{H}CC(CH_3)_3$ and $NHC_6\underline{H}_5$], 7.50, 7.65 (all d, 2H, $^3J_{HH}$ = 8.4 Hz, $NHC_6\underline{H}_5$), 7.93, 8.04 (all d, 1H, $^3J_{HH}$ = 8.3 Hz, $CH_{py}$), 9.47, 9.76, 10.00 (all s, 1H, NH), 10.42, 10.78, 12.88 (all s, 1H, NH). $^{13}$C{$^1$H} NMR (125.76 MHz, DMCO-*d*$_6$), *δ*, ppm: 23.38, 23.80, 24.34, 24.45 [$OCH(\underline{C}H_3)_2$], 30.72, 30.83, 30.85 [$C(\underline{C}H_3)_3$], 35.04, 35.09, 35.13 [$\underline{C}(CH_3)_3$], 42.61, 45.52, 45.62 (all d, $^1J_{PC}$ = 140.2 Hz, CHP), 70.45, 70.71, 70.82, 71.23 [all d, $^2J_{PC}$ = 7.3 Hz, $O\underline{C}H(CH_3)_2$], 99.87, 103.02, 103.44 ($CH_{py}$), 107.14, 110.53, 110.62 ($C_{py}$), 124.12, 124.52, 124.88, 124.49 ($NHC_6H_5$), 126.33, 126.62 [all d, $^3J_{PC}$ = 7.6 Hz, $\underline{C}HCC(CH_3)_3$], 126.62, 127.02 ($\underline{C}CHP$), 128.65, 128.90 ($NHC_6H_5$), 139.17, 139.49, [$\underline{C}C(CH_3)_3$], 139.65, 139.98 ($NHC_6H_5$), 139.50, 142.02 ($CH_{py}$), 153.09 (COH), 155.32, 156.55 ($C_{py}$), 178.52, 180.13 (C=S). $^{31}$P NMR (202.46 MHz, DMCO-*d*$_6$), *δ*, ppm: 24.50, 24.88, 24.97. Anal. Calcd for $C_{31}H_{43}N_4O_4PS$ (%): C, 63.24; H, 7.56; N, 8.94; P, 4.94; S, 5.11. Found (%): C, 63.26; H, 7.57; N,

8.98; P, 4.99; S, 5.16. HRMS (MALDI-TOF) *m/z* for $C_{33}H_{47}N_4O_4PS$: calc. 626.31 $[M]^+$, found 625.42 $[M-H]^+$.

**Diphenyl[(2-amino-6-(3-phenylthioureido)pyridin-3-yl)(3,5-di-*tert*-butyl-4-hydroxyphenyl)methyl]phosphonate 18d**. White-gray solid, yield 0.55 g (79%), M.p.: 129–130°C. IR (KBr), *ν*, $cm^{-1}$: 766, 1008, 1239, 1360, 1593, 3321, 3398, 3625. $^1H$ NMR (400.05 MHz, DMCO-$d_6$), *δ*, ppm: 1.32, 1.33 [all s, 18H, $C(CH_3)_3$], 5.14, 5.53 (all d, 1H, $^2J_{PH}$ = 28.2 Hz, CHP), 6.27, 6.45 (all d, 1H, $^3J_{HH}$ = 8.6 Hz, $CH_{py}$), 6.51 (br.s, 2H, $NH_2$), 6.62 (d, 2H, $^3J_{HH}$ = 7.6 Hz, $OC_6H_5$), 6.96-6.99 (m, 2H, $NHC_6\underline{H}_5$), 7.06-7.11 (m, 2H, $OC_6H_5$ and $NHC_6\underline{H}_5$), 7.16-7.22 [m, 3H, $C\underline{H}CC(CH_3)_3$ and $NHC_6\underline{H}_5$], 7.32-7.41 (m, 6H, $OC_6H_5$ and $NHC_6\underline{H}_5$), 7.64 (d, 2H, $^3J_{HH}$ = 7.6 Hz, $OC_6H_5$), 7.67 (d, 2H, $^3J_{HH}$ = 7.5 Hz, $OC_6H_5$), 7.92, 7.98 (all d, 1H, $^3J_{HH}$ = 8.3 Hz, $CH_{py}$), 9.41 (s, 1H, NH), 9.76, 10.48 (all s, 1H, NH). $^{13}C\{^1H\}$ NMR (100.60 MHz, DMCO-$d_6$), *δ*, ppm: 30.72 [$C(\underline{C}H_3)_3$], 35.04, 35.10 [$\underline{C}(CH_3)_3$], 42.02, 43.44 (all d, $^1J_{PC}$ = 139.6 Hz, CHP), 100.14, 103.29 ($CH_{py}$), 106.10, 108.46 ($C_{py}$), 120.56 ($OC_6H_5$), 120.57 ($CNH_6H_5$), 124.10 ($NHC_6H_5$), 125.32, 125.64 ($OC_6H_5$), 125.34 ($NHC_6H_5$), 125.71 ($NHC_6H_5$), 126.55, 126.78 (all d, $^3J_{PC}$ = 7.8 Hz, $\underline{C}HCC(CH_3)_3$], 128.59 ($OC_6H_5$), 128.63 ($OC_6H_5$), 129.31, 129.90 ($\underline{C}CHP$), 129.90 ($NHC_6H_5$), 130.24 ($NHC_6H_5$), 139.58, 139.64 [$\underline{C}C(CH_3)_3$], 139.69 ($NHC_6H_5$), 139.84 ($NHC_6H_5$), 140.13, 141.29 ($CH_{py}$), 150.41 (d, $^2J_{PC}$ = 6.4 Hz, $OC_6H_5$), 150.60 (d, $^2J_{PC}$ = 6.4 Hz, $OC_6H_5$), 150.76, 151.58 ($C_{py}$), 153.72, 153.84 (COH), 156.86 ($C_{py}$), 178.66 (all s, C=S). $^{31}P$ NMR (161.94 MHz, DMCO-$d_6$), *δ*, ppm: 19.99, 20.15. Anal. Calcd for $C_{39}H_{43}N_4O_4PS$ (%): C, 67.42; H, 6.24; N, 8.06; P, 4.46; S, 4.61. Found (%): C, 67.44; H, 6.26; N, 8.09; P, 4.51; S, 4.67. HRMS (MALDI-TOF) *m/z* for $C_{39}H_{43}N_4O_4PS$: calc. 694.27 $[M]^+$, found 695.42 $[M+H]^+$, 711.32 $[M+Na]^+$, 733.42 $[M+K]^+$.

**Dimethyl[(2,4-bis(3-phenylthioureido)phenyl)(3,5-di-*tert*-butyl-4-hydroxyphenyl)methyl]phosphonate 19a**. White-gray solid, yield 0.53 g (75%), M.p.: 140–141°C. IR (KBr), *ν*, $cm^{-1}$: 759, 1032, 1054, 1237, 1315, 1596, 3422 (NH), 3624 (OH). $^1H$ NMR (400.05 MHz, DMCO-$d_6$), *δ*, ppm: 1.32, 1.33, 1.35 [all s, 18H, $C(CH_3)_3$], 3.43, 3.47 (all d, 3H, $^3J_{PH}$ =10.6 Hz, $OCH_3$), 3.52, 3.57 (all d, 3H, $^3J_{PH}$ =10.7 Hz, $OCH_3$), 4.62, 4.78 ( all d, 1H, $^2J_{PH}$ = 24.9 Hz, CHP), 6.81, 6.85 (all s, 1H, OH), 7.09-7.14 (m, 2H, $NHC_6H_5$), 7.24-7.33 (m, 7H, $CH_{arom}$, $C\underline{H}CC(CH_3)_3$ and $NHC_6\underline{H}_5$), 7.41-7.46 (m, 4H, $NHC_6\underline{H}_5$), 7.51 (s, 1H, $CH_{arom}$), 7.65 (d, 1H, $^3J_{HH}$ = 10.0 Hz, $CH_{arom}$), 9.38 (s, 2H, NH), 9.76 (s, 1H, NH), 9.86 (s, 1H, NH). $^{13}C\{^1H\}$ NMR (DMCO-$d_6$, 100.60 MHz), *δ*, ppm: 30.80, 30.91 [$C(\underline{C}H_3)_3$], 35.02 [$\underline{C}(CH_3)_3$], 43.55 (d, $^1J_{PC}$ = 137.7 Hz, CHP), 53.20 (d, $^2J_{PC}$ = 6.8 Hz, $OCH_3$), 53.68 (d, $^2J_{PC}$ = 6.8 Hz, $OCH_3$), 122.88 (s, $CH_{arom}$), 124.11 ($NHC_6H_5$), 124.30 ($NHC_6H_5$), 124.38 ($C_{arom}$), 124.56 ($NHC_6H_5$), 124.88 ($NHC_6H_5$), 126.54 [d, $^3J_{PC}$ = 7.9 Hz, $\underline{C}HCC(CH_3)_3$], 127.22 (d, $^3J_{PC}$ = 5.0 Hz, $C_{arom}$), 128.28 ($NHC_6H_5$), 128.89 ($NHC_6H_5$), 129.91 ($CH_{arom}$), 137.21 (d, $^3J_{PC}$ = 10.4 Hz, $C_{arom}$), 138.93 ($C_{arom}$), 139.29 [s, $\underline{C}C(CH_3)_3$], 139.72 ($NHC_6H_5$), 139.93 ($NHC_6H_5$), 153.38 (COH), 179.77 (C=S), 180.98 (C=S). $^{31}P$ NMR (161.94 MHz, DMCO-$d_6$), *δ*, ppm: 28.36, 29.19. Anal. Calcd for $C_{37}H_{45}N_4O_4PS_2$ (%): C, 63.05; H, 6.44; N, 7.95; P, 4.39; S, 9.10. Found (%): C, 63.07; H, 6.46; N, 7.99; P, 4.41; S, 9.14. HRMS (MALDI-TOF) *m/z* for $C_{37}H_{45}N_4O_4PS_2$: calc. 704.26 $[M]^+$, found 705.42 $[M+H]^+$, 727.42 $[M+Na]^+$, 743.02 $[M+K]^+$.

**Diethyl[(2,4-bis(3-phenylthioureido)phenyl)(3,5-di-*tert*-butyl-4-hydroxyphenyl)methyl]phosphonate 19b**. Drak-gray solid, yield 0.57 g (78%), M.p.: 127-128°C. IR (KBr), *ν*, $cm^{-1}$: 772, 1031, 1056, 1230, 1315, 1597, 3326, 3626. $^1H$ NMR (400.05 MHz, DMCO-$d_6$), *δ*, ppm: 1.03 (t, 3H, $^3J_{HH}$ = 7.0 Hz, $OCH_2C\underline{H}_3$), 1.08 (t, 3H, $^3J_{HH}$ = 7.0 Hz, $OCH_2C\underline{H}_3$), 1.34 [s, 18H, $C(CH_3)_3$], 3.77 (m, 1H, $OC\underline{H}_2CH_3$), 3.88 (m, 3H, $OC\underline{H}_2CH_3$), 4.56 (d, 1H, $^2J_{PH}$ = 25.3 Hz, CHP), 6.84 (s, 1H, OH), 7.09-7.15 (m, 2H, $NHC_6\underline{H}_5$), 7.25-7.34 [m, 7H, $C\underline{H}CC(CH_3)_3$, $CH_{arom}$ and $NHC_6\underline{H}_5$], 7.42-7.51 (m, 4H, $NHC_6\underline{H}_5$), 7.54 (s, 1H, $CH_{arom}$), 7.68 (d, 1H, $^3J_{HH}$ = 8.4 Hz, $CH_{arom}$), 9.36 (s, 2H, NH), 9.76 (s, 1H, NH), 9.87 (s, 1H, NH). $^{13}C\{^1H\}$ NMR (100.60 MHz, DMCO-$d_6$), *δ*, ppm: 16.47 (d, $^3J_{PC}$ =

5.3 Hz, $OCH_2\underline{C}H_3$), 16.61 (d, $^3J_{PC}$ = 4.4 Hz, $OCH_2\underline{C}H_3$), 30.78 [$C(\underline{C}H_3)_3$], 35.02 [$\underline{C}(CH_3)_3$], 44.34 (d, $^1J_{PC}$ = 137.5 Hz, CHP), 62.33 (d, $^2J_{PC}$ = 7.0 Hz, $O\underline{C}H_2CH_3$), 62.67 (d, $^2J_{PC}$ = 7.0 Hz, $O\underline{C}H_2CH_3$), 122.18 ($CH_{m\text{-}Ph}$), 124.12 ($NHC_6H_5$), 124.29 ($NHC_6H_5$), 122.34 ($CH_{arom}$), 124.59 ($NHC_6H_5$), 124.89 ($NHC_6H_5$), 126.65 [d, $^3J_{PC}$ = 7.3 Hz, $\underline{C}HCC(CH_3)_3$], 127.36 ($\underline{C}CHP$), 128.69 ($NHC_6H_5$), 128.89 ($NHC_6H_5$), 131.18 ($CH_{arom}$), 137.26 (d, $^3J_{PC}$ = 10.1 Hz, $C_{arom}$), 138.86 ($C_{arom}$), 139.10 [$\underline{C}C(CH_3)_3$], 139.70 ($NHC_6H_5$), 139.93 ($NHC_6H_5$), 153.29 (COH), 179.76 (C=S), 180.96 (C=S). $^{31}P$ NMR (DMCO-$d_6$, 161.94 MHz), $\delta$, ppm: 26.36. Anal. Calcd for $C_{39}H_{49}N_4O_4PS_2$ (%): C, 63.91; H, 6.74; N, 7.64; P, 4.23; S, 8.75. Found (%): C, 63.95; H, 6.76; N, 7.69; P, 4.25; S, 8.77. HRMS (MALDI-TOF) *m*/*z* for $C_{39}H_{49}N_4O_4PS_2$: calc. 732.29 $[M]^+$, found 755.34 $[M+Na]^+$, 771.28 $[M+K]^+$.

**Diphenyl[(2,4-bis(3-phenylthioureido)phenyl)(3,5-di-*tert*-butyl-4-hydroxyphenyl)methyl]phosphonate 19d**. Violet-white solid, yield 0.66 g (80%), M.p.: 129–130°C. IR (KBr), $\nu$, $cm^{-1}$: 761, 939, 1236, 1313, 1593, 3373, 3627. $^1H$ NMR (600.13 MHz, DMCO-$d_6$,), $\delta$, ppm: 1.31 [s, 18H, $C(CH_3)_3$], 5.25 (d, 1H, $^2J_{PH}$ = 26.1 Hz, CHP), 6.64 (d, 2H, $^3J_{HH}$ = 8.1 Hz, $OC_6H_5$), 6.92 (s, 1H, OH), 6.94 (d, 2H, $^3J_{HH}$ = 8.2 Hz, $OC_6H_5$), 6.92 (s, 1H, OH), 6.94 (d, 2H, $^3J_{HH}$ = 8.2 Hz, $OC_6H_5$), 7.09-7.13 (m, 4H, $OC_6H_5$), 7.17-7.21 (m, 4H, $NHC_6\underline{H}_5$), 7.25 (t, 2H, $^3J_{HH}$ = 7.7 Hz, $OC_6H_5$), 7.28-7.36 (m, 6H, $OC_6H_5$ and $NHC_6\underline{H}_5$), 7.39 [s, 2H, $C\underline{H}CC(CH_3)_3$], 7.44-7.52 (m, 4H, $NHC_6\underline{H}_5$), 7.57 (s, 1H, $CH_{arom}$), 7.87 (d, 1H, $^2J_{HH}$ = 8.5 Hz, $CH_{arom}$), 9.41 (s, 1H, NH), 9.49 (s, 1H, NH), 9.80 (s, 1H, NH), 9.90 (s, 1H, NH). $^{13}C\{^1H\}$ NMR (150.19 MHz, DMCO-$d_6$,), $\delta$, ppm: 30.70 [$C(\underline{C}H_3)_3$], 35.03 [$\underline{C}(CH_3)_3$], 44.77 (d, $^1J_{PC}$ = 139.7 Hz, CHP), 120.61 ($OC_6H_5$), 121.08 ($OC_6H_5$), 122.72 ($CH_{arom}$), 124.13 ($C_{arom}$), 124.31 ($NHC_6H_5$), 124.61 ($NHC_6H_5$), 124.83 ($CH_{arom}$), 125.14 ($NHC_6H_5$), 125.40 ($NHC_6H_5$), 125.73 ($C_{arom}$), 127.08 [d, $^3J_{PC}$ = 7.7 Hz, $\underline{C}HCC(CH_3)_3$], 128.67 ($NHC_6H_5$), 128.92 ($NHC_6H_5$), 129.77 ($\underline{C}CHP$), 129.96 ($OC_6H_5$), 130.21 ($OC_6H_5$), 130.41 ($CH_{arom}$), 137.60 (d, $^3J_{PC}$ = 10.1 Hz, $C_{arom}$), 139.57 ($C_{arom}$), 139.59 [$\underline{C}C(CH_3)_3$], 139.80 ($NHC_6H_5$), 150.49 (d, $^2J_{PC}$ = 9.6 Hz, $OC_6H_5$), 150.80 (d, $^2J_{PC}$ = 9.6 Hz, $OC_6H_5$), 153.86 (COH), 180.07 (C=S), 181.20 (C=S). $^{31}P$ NMR (242.94 MHz, DMCO-$d_6$), $\delta$, ppm: 19.89. Found (%): C, 68.06; H, 5.99; N, 6.78; P, 3.76; S, 7.71. Calc. for $C_{47}H_{49}N_4O_4PS_2$ (%): C, 68.09; H, 6.01; N, 6.80; P, 3.79; S, 7.73. HRMS (MALDI-TOF) *m*/*z* for $C_{47}H_{49}N_4O_4PS_2$: calc. 828.29 $[M]^+$, found 851.31 $[M+Na]^+$, 867.32 $[M+K]^+$.

## The X-ray diffraction data

The X-ray diffraction data for the crystals of compound **7a** was collected on a Rigaku XtaLab Synergy S instrument with a HyPix detector and a PhotonJet microfocus X-ray tube using Cu K$\alpha$ (1.54184 Å) radiation at low temperature. Images were indexed and integrated using the CrysAlisPro data reduction package. Data were corrected for systematic errors and absorption using the ABSPACK module: numerical absorption correction based on Gaussian integration over a multifaceted crystal model and empirical absorption correction based on spherical harmonics according to the point group symmetry using equivalent reflections. The GRAL module was used for analysis of systematic absences and space group determination.

The X-ray diffraction data for the crystals compound **7b** were collected on a Smart Apex II automatic diffractometer using graphite monochromated radiation. The structures **7a**,**b** was solved by direct methods using SHELXT [3] and refined by the full-matrix least-squares on $F^2$ using SHELXL. Non-hydrogen atoms were refined anisotropically. The hydrogen atoms were inserted at the calculated positions and refined as riding atoms. All the non-hydrogen atoms

were refined with anisotropic atomic displacement parameters. All figures were made using the program OLEX2 [4]. Crystallographic data for the structures **7a**,**b** reported in this paper have been deposited with the Cambridge Crystallographic Data Center (deposit number is 2266198 - **7a**; 1957500 - **7b**).

Compounds **7a** and b crystallize in the centrosymmetric space group *P*-1. The bond lengths, valence and torsion angles in the molecules of compounds **7a**,**b** in the crystal are in the ranges of characteristic values for the corresponding types of bonds. The compound **7a** crystallizes with two molecules in an independent part of the unit cell. The geometry of independent molecules differs very slightly in the configuration of the substituent, in which the phenyl fragment rotated differently relative to the plane of the urea fragment (the angles between the planes are 19.3(3)° and 9.8(2)° in different molecules).Meanwhile only the benzene ring of the substituent comes out of the plane. In molecules **7b** the curvature of the urea substituent is almost the same (the angle between the planes is 23.86(7)°), however the curvature does not occur along the nitrogen atom, as in **7a**, but along the carbonyl carbon atom. The N-H…O and C-H…O bonds stabilizing the conformations of these substituents are almost the same, the N…N distances are 2.719(7)Å, distances C…O - 2.85 (1)Å. The main difference in the geometry of the molecules of compounds **7a** and 17 in crystals is the reversal of the diaminopyridine substituent relative to the rest of the molecule – the angles between the plane of the substituent and P-C bond are 121.1(2)° and 121.9(2)° in **7a** and 101.1(1)° in **7b**. Meanwhile no additional intramolecular interactions were detected in **7b**. The presence of a large number of hydrogen binding centers leads to the formation of the crystal packing of both compounds due to hydrogen bonds. The independent molecules in **7a** form infinite chains in which the molecules are bonded by two classical NH…O hydrogen bonds: between one of the H-atoms of the amino group and carbonyl oxygen and the hydrogen atom of the NH group and the carbonyl oxygen atom. The second hydrogen atom of the amino group and the hydrogen of the OH group are bound to DMSO molecules which are localized between the chains of molecules (SI, table S1).

An infinite ribbon of centrosymmetric hydrogen-bonded dimers is formed in crystal of compound **7b**. Their formation occurs due to the bonding of hydrogen atoms of the NH groups and carboxylic oxygens. Dimers crosslinking is carried out due to the O-H…O interactions between hydroxyl hydrogen and phosphoryl oxygen. One of the hydrogen atoms of the amino groups is bound to the solvate molecules of DMSO, thereby embedded in the ribbons (SI, table S2). The other H-atom is directed towards the aromatic fragment of di-*tret*-butylphenol with a distance to the nearest carbon atom of 2.500(3)Å, and to the centroid of the cycle of 3.1708(2)Å, which further stabilizes the conformation of the molecule.

Thus, the crystal structure of both compounds is formed due to hydrogen bonds and consists of columns or ribbons directed parallel to the 0a axis and connected in a three-dimensional grid.

Crystal Data for **7a**: $C_{33}H_{51}N_4O_7PS_2$ ($M$ =710.86 g/mol): triclinic, space group P-1 (no. 2), $a$ = 9.1479(3) Å, $b$ = 15.6620(3) Å, $c$ = 26.1825(3) Å, $\alpha$ = 90.5049(12)°, $\beta$ = 90.946(2)°, $\gamma$ = 92.230(2)°, $V$ = 3747.73(15) Å$^3$, $Z$ = 4, $T$ = 100.00(10) K, μ(CuK$\alpha$) = 2.096 mm$^{-1}$, $Dcalc$ = 1.260 g/cm$^3$, 108799 reflections measured (5.648° ≤ 2Θ ≤ 153.844°), 14950 unique ($R_{int}$ = 0.1633, $R_{sigma}$ = 0.0588) which were used in all calculations. The final $R_1$ was 0.1187 (I > 2σ(I)) and $wR_2$ was 0.3234 (all data).

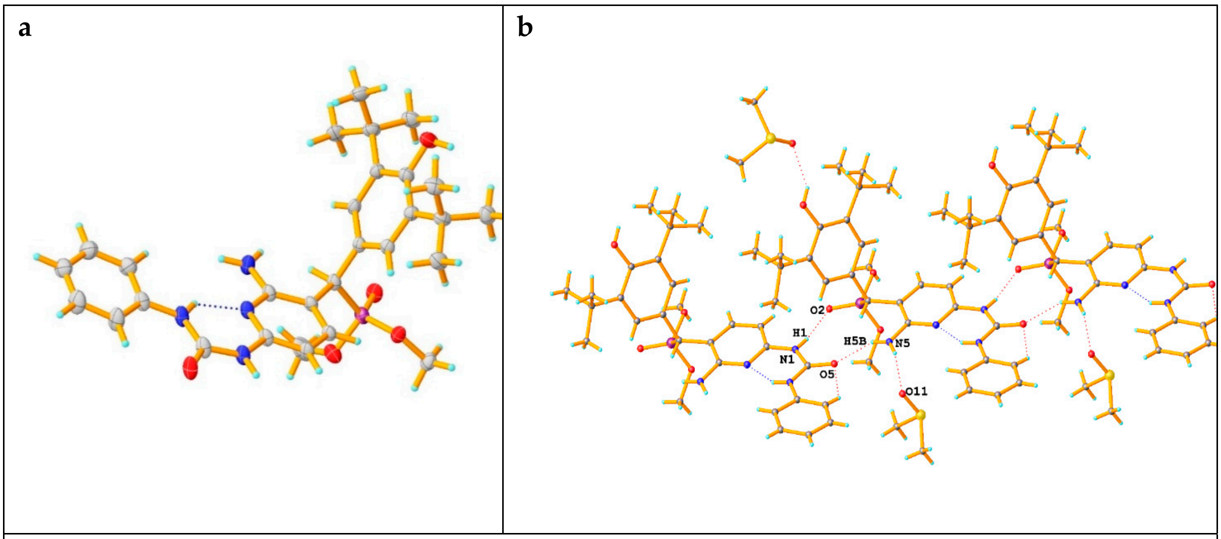


**Figure S1**. **a**) Molecular structure of compound **7a** in crystal. Ellipsoids are shown with 50% probability. **b**) System of H-bonds in crystal of compound **7a**.

**Table S1.** H-bonds in crystal of compound **7a**

| | | D - H | H...A | D...A | D - H...A |
|---|---|---|---|---|---|
| O7-H7... S1' | | 0.840 | 3.008 | 3.389(5) | 110.09 |
| O7-H7... O13' | | 0.840 | 2.029 | 2.698(8) | 136.02 |
| N1-H1... O2 | x-1, y, z | 0.880 | 2.003 | 2.872(7) | 169.32 |
| N2-H2... N7 | | 0.880 | 2.009 | 2.719(7) | 136.90 |
| N5-H5A... O11 | | 0.880 | 2.051 | 2.894(8) | 159.93 |
| N5-H5A... S2' | | 0.880 | 2.875 | 3.578(9) | 138.08 |
| N5-H5B... O5 | x+1, y, z | 0.880 | 2.180 | 2.978(8) | 150.66 |
| C12-H12... O5 | x+1, y, z | 1.000 | 2.257 | 3.243(8) | 168.41 |
| C12-H12... N5 | | 1.000 | 2.388 | 2.906(8) | 111.36 |
| C20-H20... O2 | x-1, y, z | 0.950 | 2.515 | 3.271(7) | 136.58 |
| C38-H38... O11 | | 0.950 | 2.581 | 3.393(7) | 143.55 |
| C50-H50... O5 | | 0.950 | 2.245 | 2.855(8) | 121.22 |

| | | | | | |
|---|---|---|---|---|---|
| O4-H4... S7' | | 0.840 | 3.028 | 3.717(8) | 140.80 |
| O4-H4... O15' | | 0.840 | 1.845 | 2.649(9) | 159.89 |
| O4-H4... O14'' | | 0.840 | 2.203 | 2.701(8) | 118.01 |
| N3-H3A... O12 | | 0.880 | 2.004 | 2.861(6) | 164.40 |
| N3-H3A... S4'' | | 0.880 | 2.997 | 3.709(9) | 139.33 |
| N3-H3B... O10 | x+1, y, z | 0.880 | 2.239 | 3.018(8) | 147.42 |
| N4-H4A... O6 | x-1, y, z | 0.880 | 2.012 | 2.871(8) | 165.08 |
| N6-H6... N8 | | 0.880 | 2.013 | 2.719(7) | 136.36 |
| C25-H25... O10 | x+1, y, z | 1.000 | 2.229 | 3.215(8) | 168.47 |
| C25-H25... N3 | | 1.000 | 2.409 | 2.909(7) | 110.08 |
| C28-H28... O6 | x-1, y, z | 0.950 | 2.542 | 3.294(8) | 136.26 |
| C56-H56... O10 | | 0.950 | 2.257 | 2.85(1) | 119.71 |
| C67'-H67C'... S3' | -x, -y, -z | 0.980 | 3.017 | 3.690(8) | 126.92 |
| C65-H65A'... N7 | -x+1, -y+1, -z+1 | 0.980 | 2.544 | 3.491(8) | 162.43 |
| C65-H65D''... N1 | -x+1, -y+1, -z+1 | 0.980 | 2.560 | 3.537(8) | 175.11 |
| C63-H63A'... N6 | | 0.980 | 2.698 | 3.550(6) | 145.53 |
| C63-H63E''... N3 | -x, -y, -z | 0.980 | 2.504 | 3.461(9) | 165.38 |
| C63-H63F''... N6 | | 0.980 | 2.608 | 3.550(7) | 161.26 |

Crystal data for **7b**: $C_{31}H_{43}N_4O_5P$*$(CH_3)_2SO$, M = 660.79, colorless crystal, triclinic, space group P-1, Z = 2, a=10.0771(8), b=13.8844(10), c=13.8959(11)Å, $\alpha$=68.025(2), β=82.413(2), $\gamma$=77.857(2)°, V = 1759.6(2) Å$^3$, *calc = 1.247 g/cm$^3$, * = 0.185 mm$^{-1}$, 14008 reflections collected (±*h*, ±*k*, ±*l*), 6875 independent ($R_{int}$ =0.0426) and 4452 observed reflections [$I \geq 2\ \sigma(I)$], 434 refined parameters, *R* = 0.0609, $wR^2$ = 0.1686, residual electron density 0.58 (-0.44)eÅ$^{-3}$.

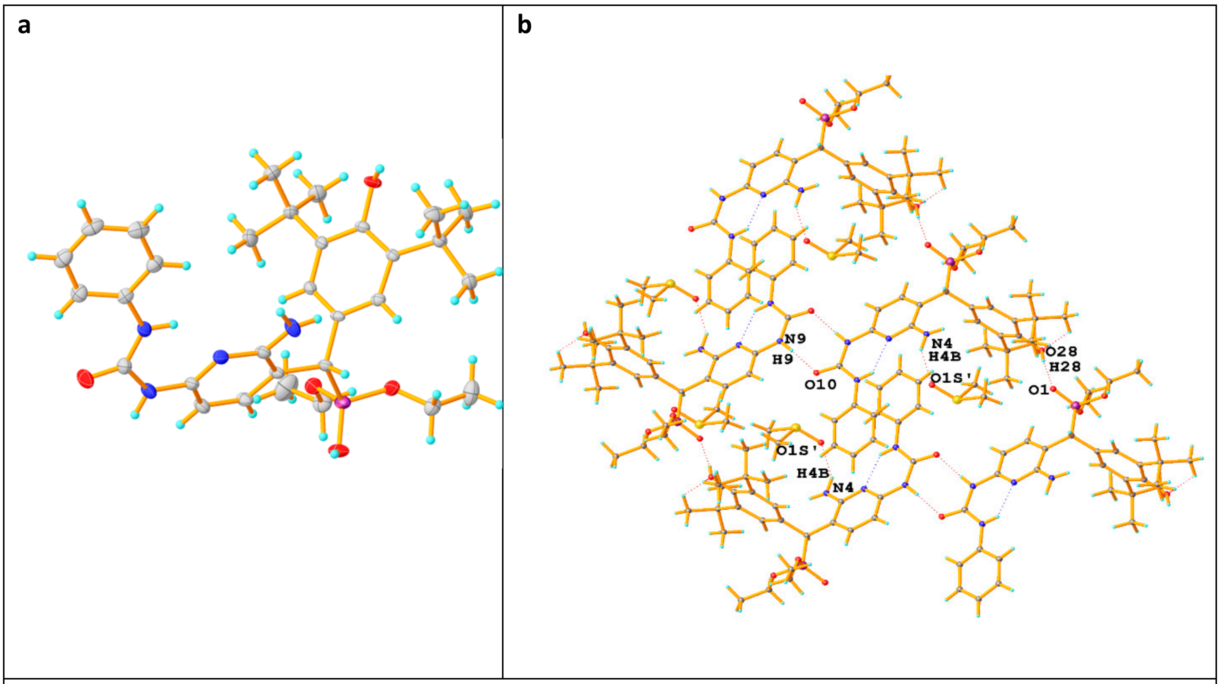


**Figure S2**. **a**) Molecular structure of compound **7b** in crystal. Ellipsoids are shown with 50% probability. **b**) System of H-bonds in crystal of compound **7b**.

**Table S2.** H-bonds in crystal of compound **7b**

| H-bond | Symmetry equivalent | D - H | H...A | D...A | D - H...A |
|---|---|---|---|---|---|
| N4-H4B...S1S | 1-x,1-y,1-z | 0.97 | 2.84 | 3.469(7) | 124 |
| N4-H4B...O1S' | 1-x,1-y,1-z | 0.97 | 2.11 | 2.98(4) | 149 |
| N4-H4B...O1S | 1-x,1-y,1-z | 0.97 | 2.01 | 2.86(6) | 146 |
| N4-H4B...O1S" | 1-x,1-y,1-z | 0.97 | 2.08 | 2.95(5) | 150 |
| N9-H9...O10 | 2-x,1-y,-z | 0.89 | 1.98 | 2.865(4) | 175 |
| N11-H11...N5 | | 0.89 | 2.04 | 2.753(4) | 136 |
| O28-H28...O1 | -1+x,y,z | 0.98 | 1.79 | 2.680(3) | 150 |
| C2-H2...O1S | [1555.03] | 1.00 | 2.51 | 3.48(5) | 166 |
| C2-H2...O1S" | [1555.04] | 1.00 | 2.52 | 3.49(5) | 163 |
| C8-H8...O33 | | 0.95 | 2.51 | 3.147(5) | 124 |
| C13-H13...O10 | | 0.95 | 2.28 | 2.878(4) | 120 |

| | | | | | |
|---|---|---|---|---|---|
| C25-H25A...O28 | | 0.98 | 2.26 | 2.920(4) | 124 |
| C26-H26A...O28 | | 0.98 | 2.36 | 3.003(4) | 122 |
| C30-H30C...O1 | -1+x,y,z | 0.98 | 2.37 | 3.341(4) | 172 |
| C31-H31B...O28 | | 0.98 | 2.25 | 2.902(3) | 123 |
| C34-H34A...O28 | 1+x,y,z | 0.99 | 2.51 | 3.445(5) | 157 |
| H-bond | Symmetry equivalent | D - H | H...A | D...A | D - H...A |

Electrochemical measurements

On cyclic voltammetry compound **8b** (Figure S5) also, after the addition of increasing amounts of trifluoroacetic acid shows that the OH group, despite the close location of the oxidation peaks with the pyridine fragment is oxidized slightly more positively. As shown above, although compound **8b** exhibits biological activity, it is inferior in selectivity to the leader compound **17b** - this is directly related to the fact that the oxidation process into the quinone form is slowed down by parallel processes in the molecule.

The CV of the oxidation of compound **17b** shows that oxEp1 corresponds to the OH group because the first oxidation peak increases with increasing amounts of acid (Figure S6).

Lipid peroxidation inhibition, oxygen radical scavenging capacity and DPPH anti-radical activity of test compounds and DPPH values expressed in TE showed differences between compounds **14b** and **17b.** We compared these compounds on iSDLSV curves (Figure S7).

It is important to note that **17b** does not change the shape of the re-reductive wave on the semi-differential CV both in the presence and in the absence of acid. The reverse semi-differential wave (or iSDLSV) of the compound under study should not change shape in the presence of acid, since the phenol-quinone equilibrium could be disrupted.

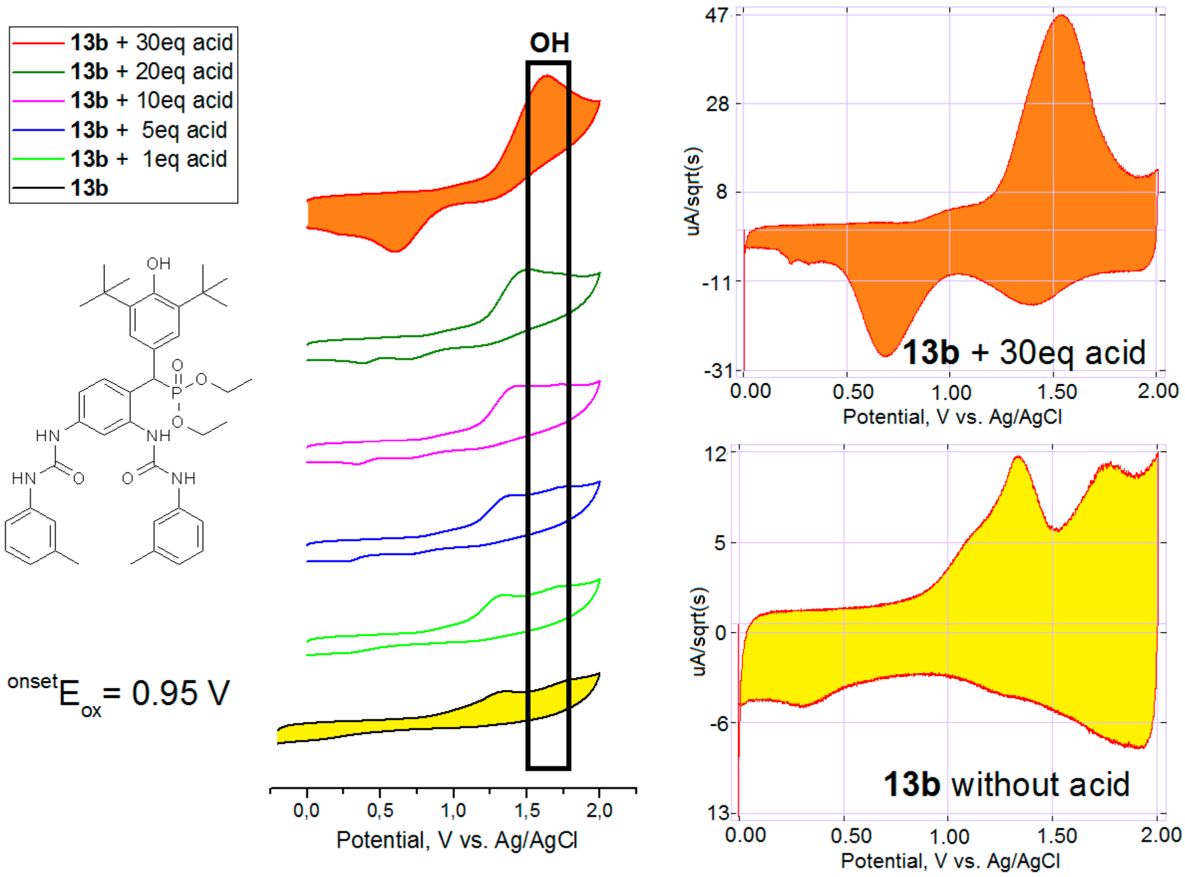


**Figure S3.** Cyclic voltammogram for oxidation of compound **13b** (left). Semi-derivative of CV for oxidation of **13b**. Conditions: 0.1 mM $CH_3CN$ (0.1 M $Bu_4NBF_4$); Potentials vs. Ag/AgCl; Work electrode: GC; Scan rate: 0.1 V/s

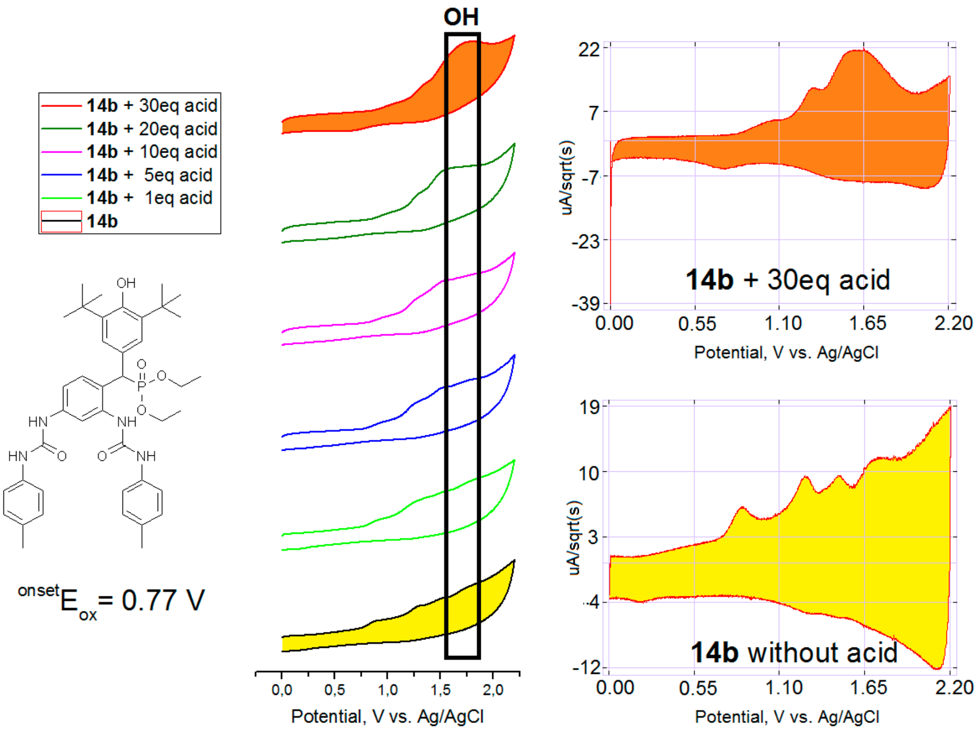


**Figure S4** Cyclic voltammogram for oxidation of compound **14b** (left). Semi-derivative of CV for oxidation of **14b**. Conditions: 0.1 mM $CH_3CN$ (0.1 M $Bu_4NBF_4$); Potentials vs. Ag/AgCl; Work electrode: GC; Scan rate: 0.1 V/s

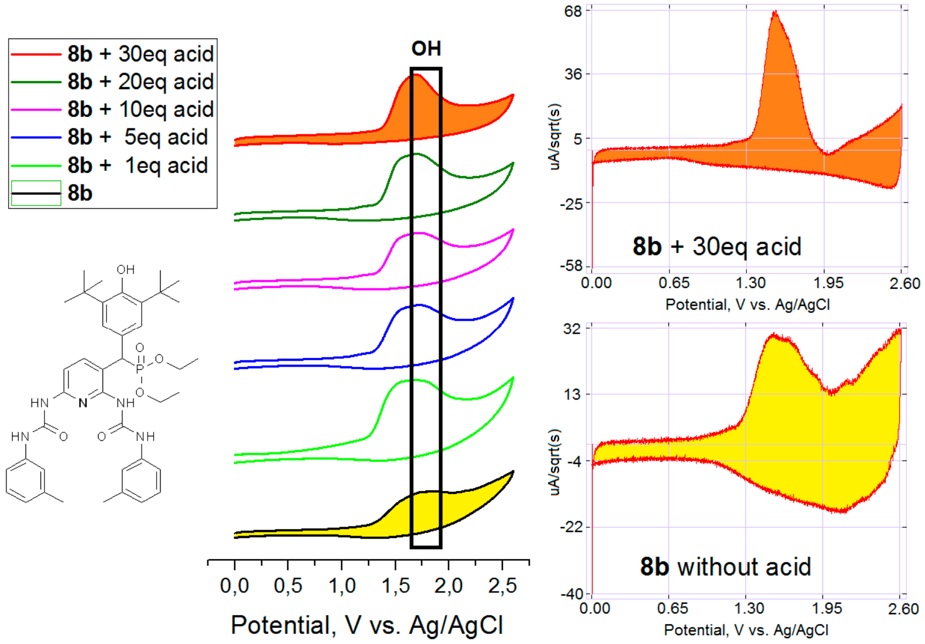


**Figure S5**. Cyclic voltammogram for oxidation of compound **8b** (left). Semi-derivative of CV for oxidation of **8b**. Conditions: 0.1 mM $CH_3CN$ (0.1 M $Bu_4NBF_4$); Potentials vs. Ag/AgCl; Work electrode: GC; Scan rate: 0.1 V/s

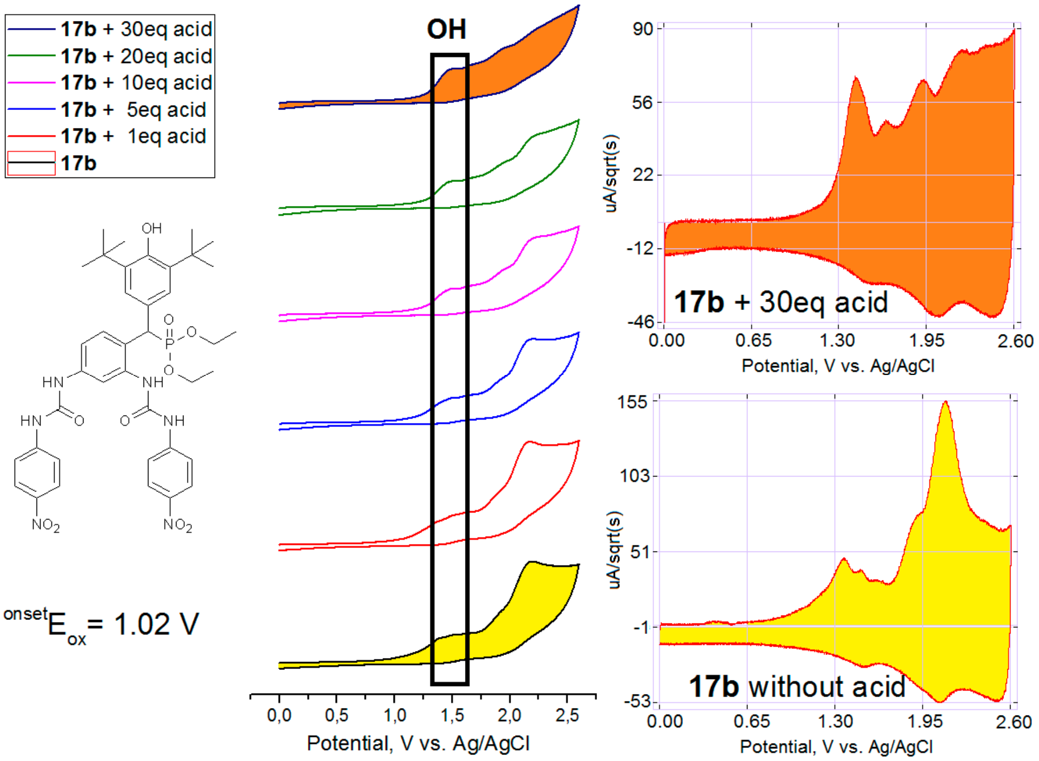


**Figure S6.** Cyclic voltammogram for oxidation of compound **17b** (left). Semi-derivative of CV for oxidation of **17b**. Conditions: 0.1 mM $CH_3CN$ (0.1 M $Bu_4NBF_4$); Potentials vs. Ag/AgCl; Work electrode: GC ; Scan rate: 0.1 V/s

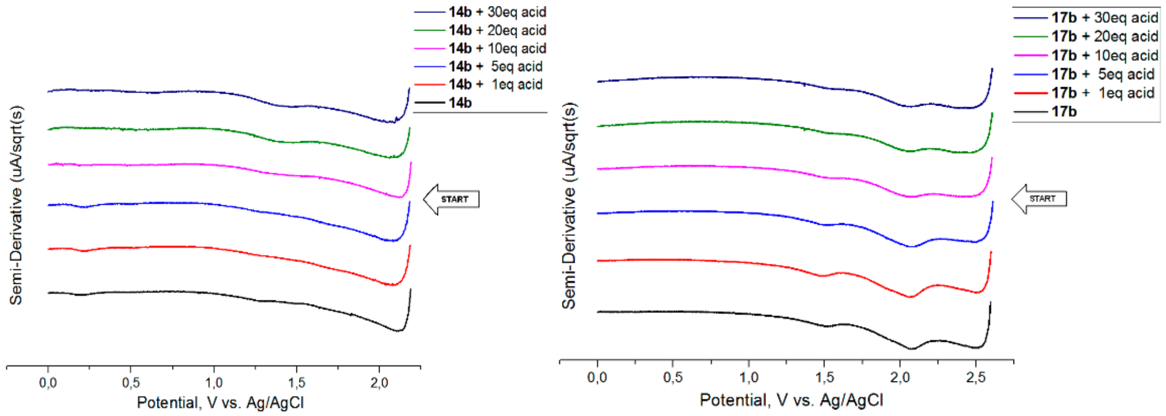


**Figure S7**. iSDLSV for reduction of the oxidized compounds **14b** and **17b.** Conditions: 0.1 mM $CH_3CN$ (0.1 M $Bu_4NBF_4$); Potentials vs. Ag/AgCl; Work electrode: GC

Cartesian Coordinates

**Computational Details**. All quantum chemical calculations were performed by the Gaussian16 program package[1] using (U)M06-2X/6-311++G(d, p) level of theory [5–7] and atom-pairwise

dispersion correction with the Becke-Johnson damping scheme (D3) [8,9]. All calculations were performed using SMD solvation correction method of choice for water. TightSCF convergence criteria was applied throughout. Numerical harmonic frequency calculations were used to obtain thermodynamic quantities and to verify that all stationary points found were local minima.

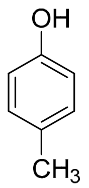


**p - Cresol ($C_7H_8O$)**
# Of imaginary frequencies = 0
E = -346.732914616 Eh
Symmetry C1
Charge 0 Multiplicity 1

| | | | |
|---|---|---|---|
| C | 0.641764000 | -1.198701000 | -0.004301000 |
| C | 1.375717000 | -0.013254000 | -0.004687000 |
| C | 0.667703000 | 1.193388000 | -0.004273000 |
| C | -0.720658000 | 1.220226000 | -0.000679000 |
| C | -1.430480000 | 0.021460000 | 0.001577000 |
| C | -0.752052000 | -1.191647000 | -0.000678000 |
| H | 1.164285000 | -2.149708000 | -0.007732000 |
| H | 1.214831000 | 2.130952000 | -0.007667000 |
| H | -1.263066000 | 2.158750000 | -0.001435000 |
| H | -1.313234000 | -2.120455000 | -0.001447000 |
| C | 2.882147000 | -0.019869000 | 0.005424000 |
| H | 3.268381000 | 0.355920000 | 0.956791000 |
| H | 3.269083000 | -1.029304000 | -0.140514000 |
| H | 3.282660000 | 0.619546000 | -0.784576000 |
| O | -2.803976000 | 0.095036000 | 0.003677000 |
| H | -3.175984000 | -0.795597000 | 0.002869000 |

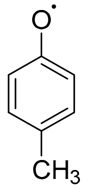


**p - Cresol radical ($C_7H_7O$)**
# Of imaginary frequencies = 0
E = -346.093876275 Eh
Symmetry C1
Charge 0 Multiplicity 2

| | | | |
|---|---|---|---|
| C | 0.587920000 | -1.220772000 | -0.012962000 |
| C | 1.305959000 | 0.000126000 | -0.014843000 |
| C | 0.587835000 | 1.220910000 | -0.012958000 |
| C | -0.778792000 | 1.236199000 | -0.002512000 |
| C | -1.532104000 | -0.000034000 | 0.003898000 |
| C | -0.778667000 | -1.236211000 | -0.002509000 |
| H | 1.145009000 | -2.151338000 | -0.020443000 |
| H | 1.144811000 | 2.151538000 | -0.020430000 |
| H | -1.337854000 | 2.164991000 | -0.001616000 |
| H | -1.337641000 | -2.165055000 | -0.001627000 |
| C | 2.797950000 | -0.000005000 | 0.012971000 |
| H | 3.143611000 | -0.003681000 | 1.053315000 |
| H | 3.202156000 | -0.890383000 | -0.470068000 |
| H | 3.202387000 | 0.893398000 | -0.464176000 |
| O | -2.787886000 | -0.000093000 | 0.012317000 |

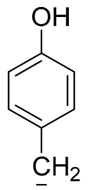


**p - Cresol anion ($C_7H_7O$)**
# Of imaginary frequencies = 0
E = -346.180516318 Eh
Symmetry C1
Charge -1 Multiplicity 1

| | | | |
|---|---|---|---|
| C | 0.725337000 | 1.201363000 | -0.002328000 |
| C | 1.495354000 | -0.010315000 | 0.001782000 |
| C | 0.698943000 | -1.203486000 | -0.002601000 |
| C | -0.690252000 | -1.181758000 | -0.001516000 |
| C | -1.386733000 | 0.023763000 | 0.000256000 |
| C | -0.662070000 | 1.212995000 | -0.001555000 |
| H | 1.252857000 | 2.151135000 | -0.002795000 |
| H | 1.203198000 | -2.165731000 | -0.003328000 |
| H | -1.245753000 | -2.116632000 | -0.003171000 |
| H | -1.197278000 | 2.158232000 | -0.003105000 |
| C | 2.902270000 | -0.024224000 | 0.018686000 |
| H | 3.465170000 | 0.900772000 | -0.041215000 |
| H | 3.447572000 | -0.959751000 | -0.040144000 |
| O | -2.785771000 | 0.091057000 | 0.001783000 |
| H | -3.136685000 | -0.806509000 | 0.003154000 |

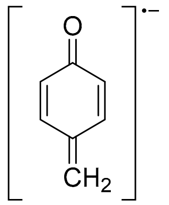


**p - Quinone methide ($C_7H_6O$)**
# Of imaginary frequencies = 0
E = Eh
Symmetry C1
Charge 0 Multiplicity 1

| | | | |
|---|---|---|---|
| C | 0.637915000 | -1.248095000 | 0.000000000 |
| C | 1.392471000 | 0.000000000 | 0.000000000 |
| C | 0.637915000 | 1.248095000 | 0.000000000 |
| C | -0.705377000 | 1.254280000 | 0.000000000 |
| C | -1.466475000 | 0.000000000 | 0.000000000 |
| C | -0.705377000 | -1.254280000 | 0.000000000 |
| H | 1.201390000 | -2.175592000 | 0.000000000 |
| H | 1.201390000 | 2.175592000 | 0.000000000 |
| H | -1.276144000 | 2.175800000 | 0.000000000 |
| H | -1.276144000 | -2.175800000 | 0.000000000 |
| C | 2.736961000 | 0.000000000 | 0.000000000 |

| | | | |
|---|---|---|---|
| H | 3.295492000 | -0.929895000 | 0.000000000 |
| H | 3.295492000 | 0.929895000 | 0.000000000 |
| O | -2.701209000 | 0.000000000 | 0.000000000 |

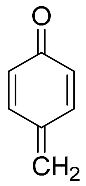

***p* - Quinone methide radical anion ($C_7H_6O$)**
# Of imaginary frequencies = 0
E = -345.594284851 Eh
Symmetry CS
Charge -1 Multiplicity 2

| | | | |
|---|---|---|---|
| C | -0.000156000 | 0.653790000 | 1.210867000 |
| C | 0.000538000 | 1.413647000 | 0.000000000 |
| C | -0.000156000 | 0.653790000 | -1.210867000 |
| C | -0.000156000 | -0.721195000 | -1.211276000 |
| C | 0.000017000 | -1.483380000 | 0.000000000 |
| C | -0.000156000 | -0.721195000 | 1.211276000 |
| H | -0.000423000 | 1.190715000 | 2.155430000 |
| H | -0.000423000 | 1.190715000 | -2.155430000 |
| H | -0.000263000 | -1.266217000 | -2.150764000 |
| H | -0.000263000 | -1.266217000 | 2.150764000 |
| C | 0.000484000 | 2.811732000 | 0.000000000 |
| H | 0.000329000 | 3.367779000 | 0.929922000 |
| H | 0.000329000 | 3.367779000 | -0.929922000 |
| O | -0.000222000 | -2.778460000 | 0.000000000 |

**Oxygen triplet ( $^3O_2$)**
# Of imaginary frequencies = 0
E =  -150.308322466 Eh
Symmetry C1
Charge 0 Multiplicity 3

| | | | |
|---|---|---|---|
| O | 0.000000000 | 0.000000000 | 0.593916000 |
| O | 0.000000000 | 0.000000000 | -0.593916000 |

**Oxygen radical anion ($O_2$)**
# Of imaginary frequencies = 0
E = -150.420661509 Eh
Symmetry C1
Charge -1 Multiplicity 2

| | | | |
|---|---|---|---|
| O | 0.000000000 | 0.000000000 | 0.658816000 |
| O | 0.000000000 | 0.000000000 | -0.658816000 |

# NMR spectra of compounds

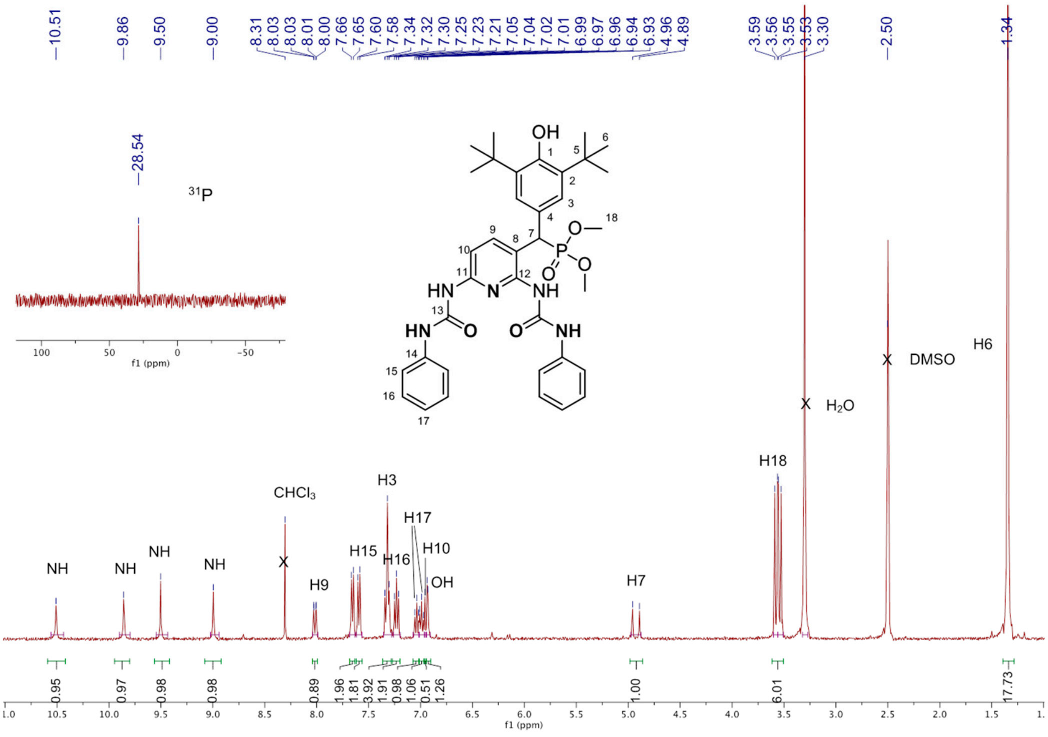


**Figure S8**. $^{1}$H-, $^{31}$P- NMR of co8mpound **5a**.

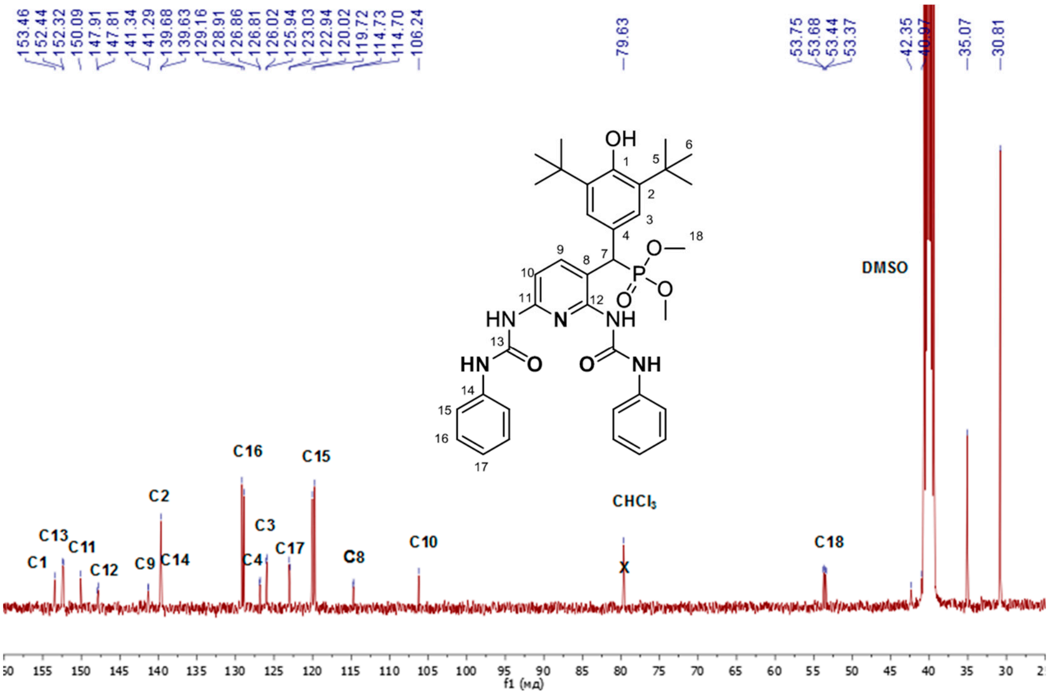


**Figure S9**. $^{13}$C- NMR of compound **5a**.

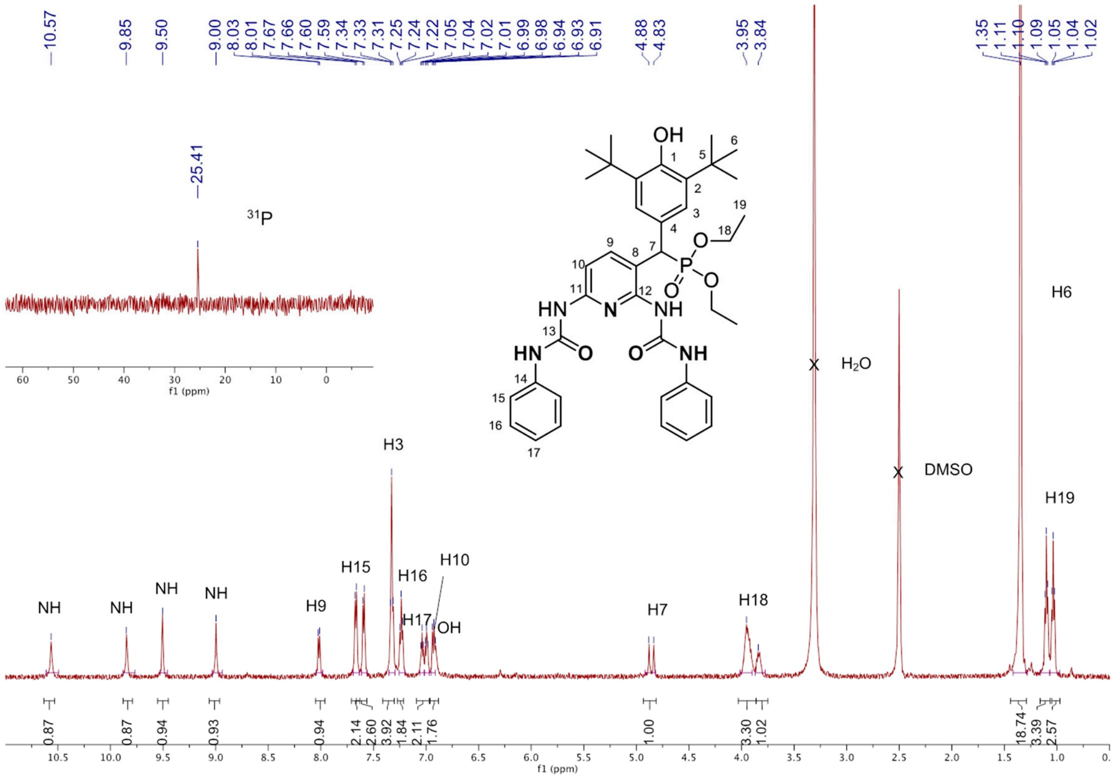


**Figure S10**. $^{1}H$-, $^{31}P$-NMR of compound **5b**.

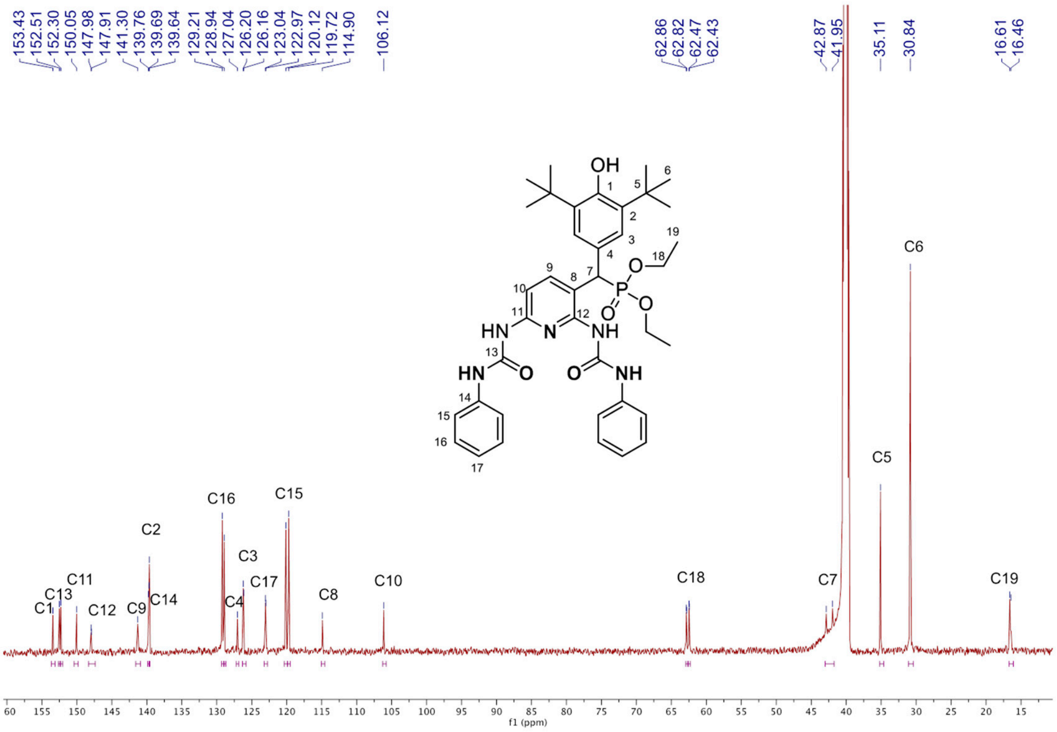


**Figure S11**. $^{13}C$- NMR of compound **5b**.

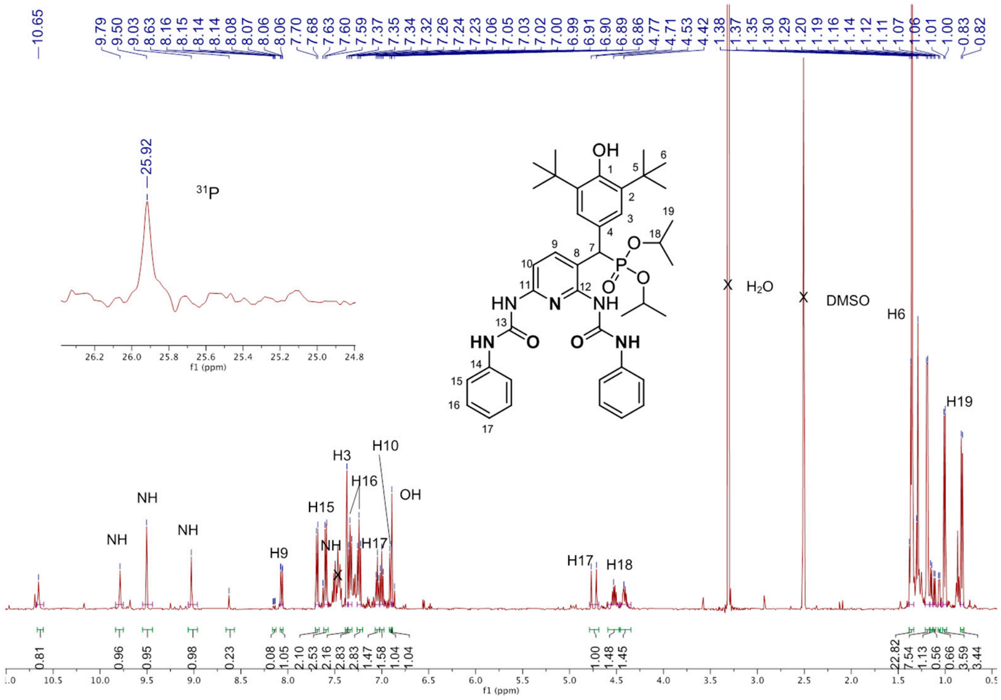


**Figure S12**. $^{1}H$-, $^{31}P$- NMR of compound **5c**.

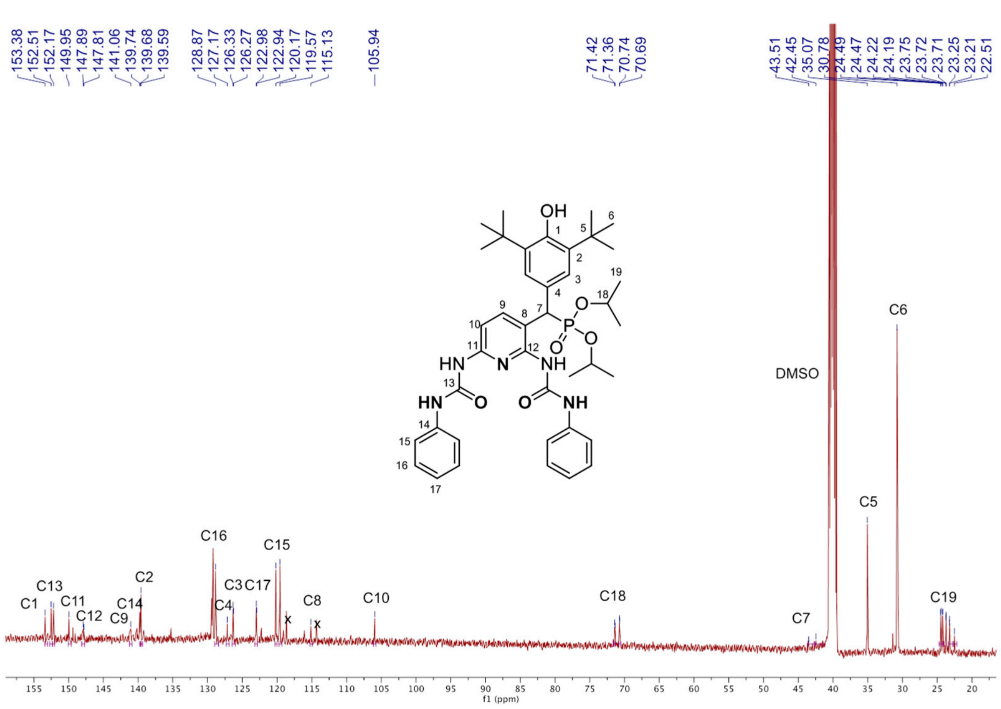


**Figure S13**. $^{13}C$- NMR of compound **5c**.

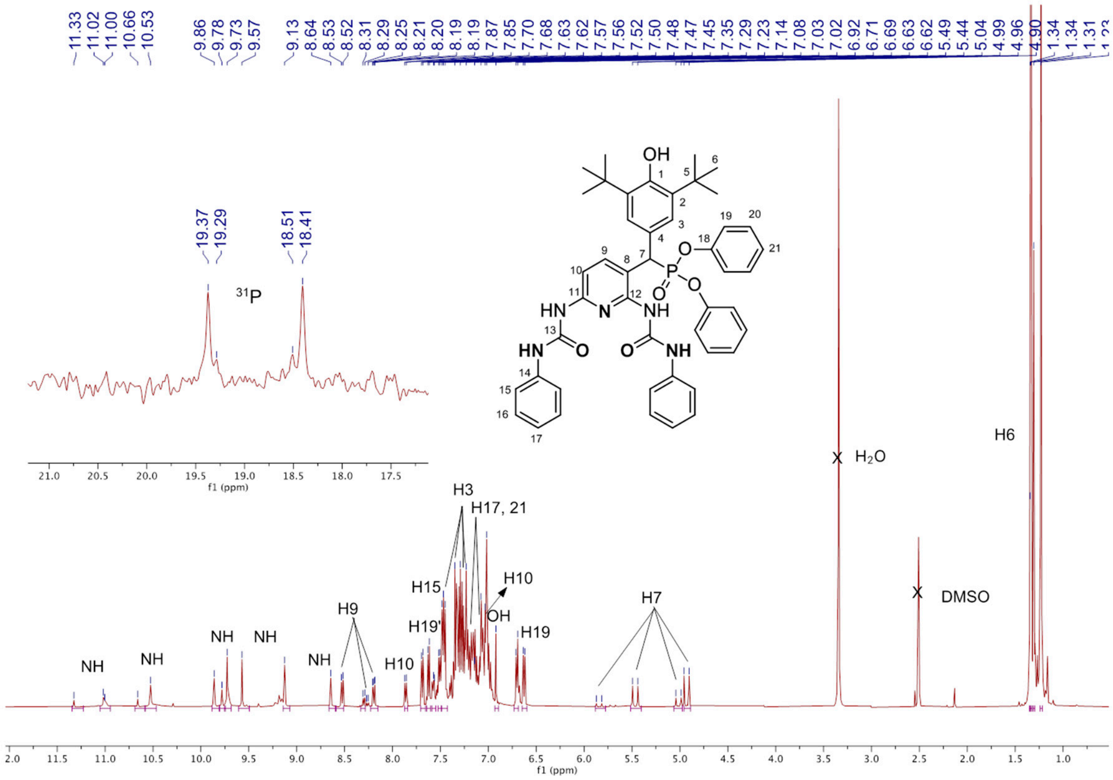


**Figure S14**. $^{1}$H-,$^{31}$P-NMR of compound **5d**.

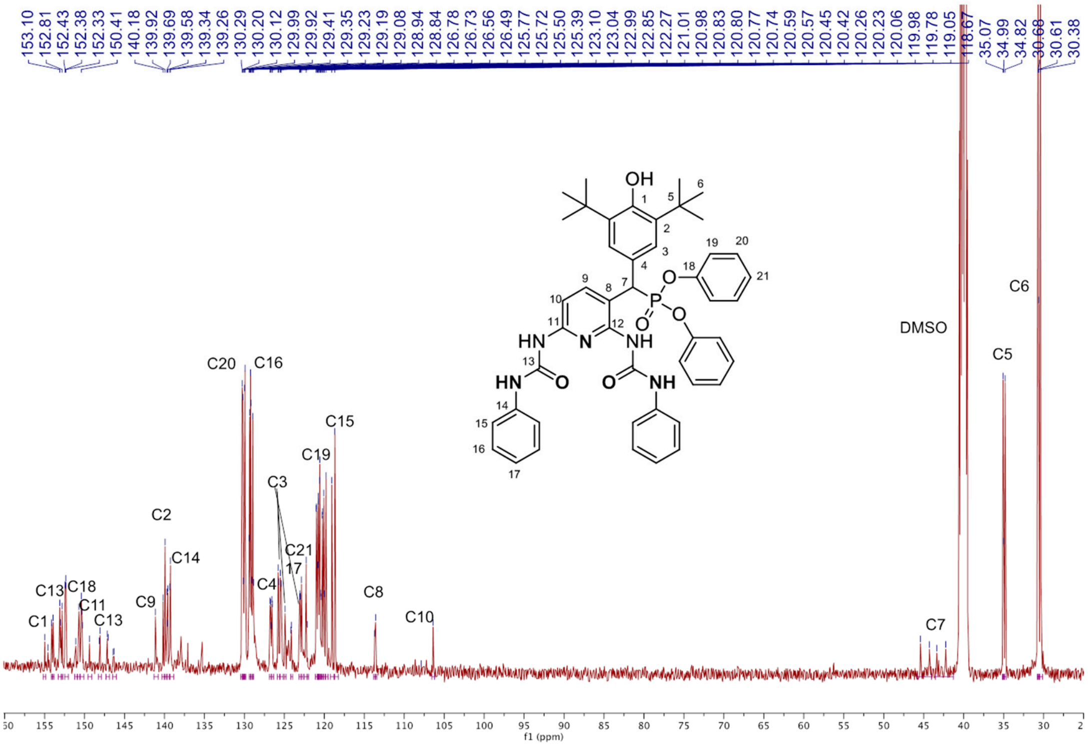


**Figure S15**. $^{13}$C- NMR of compound **5d**.

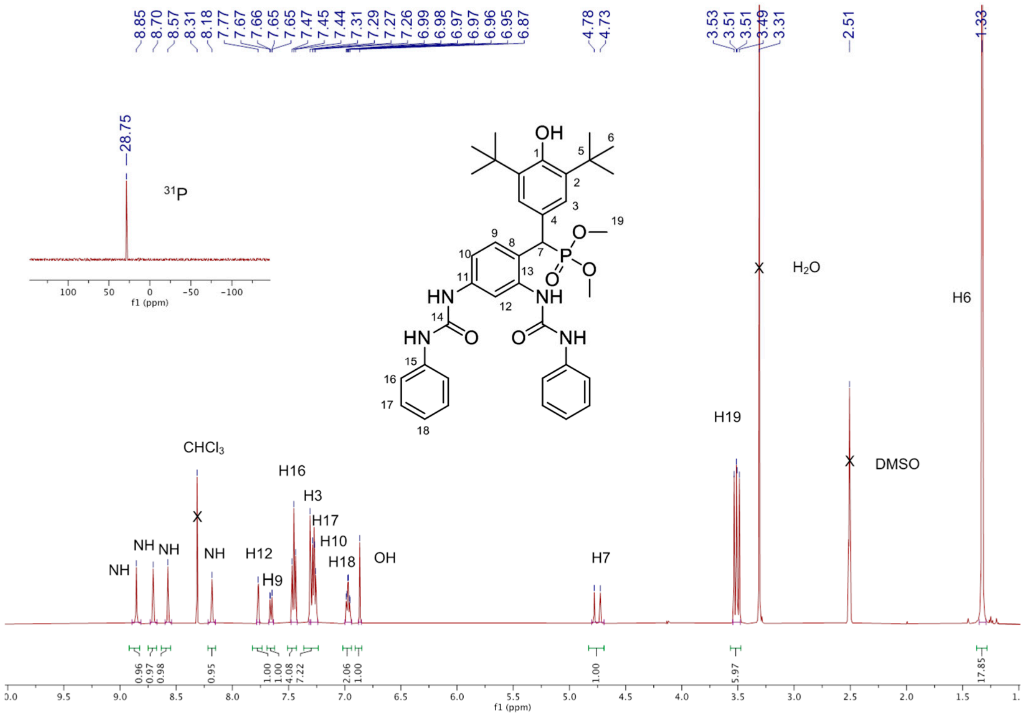

**Figure S16**. $^{1}$H-, $^{31}$P- NMR of compound **6a**.

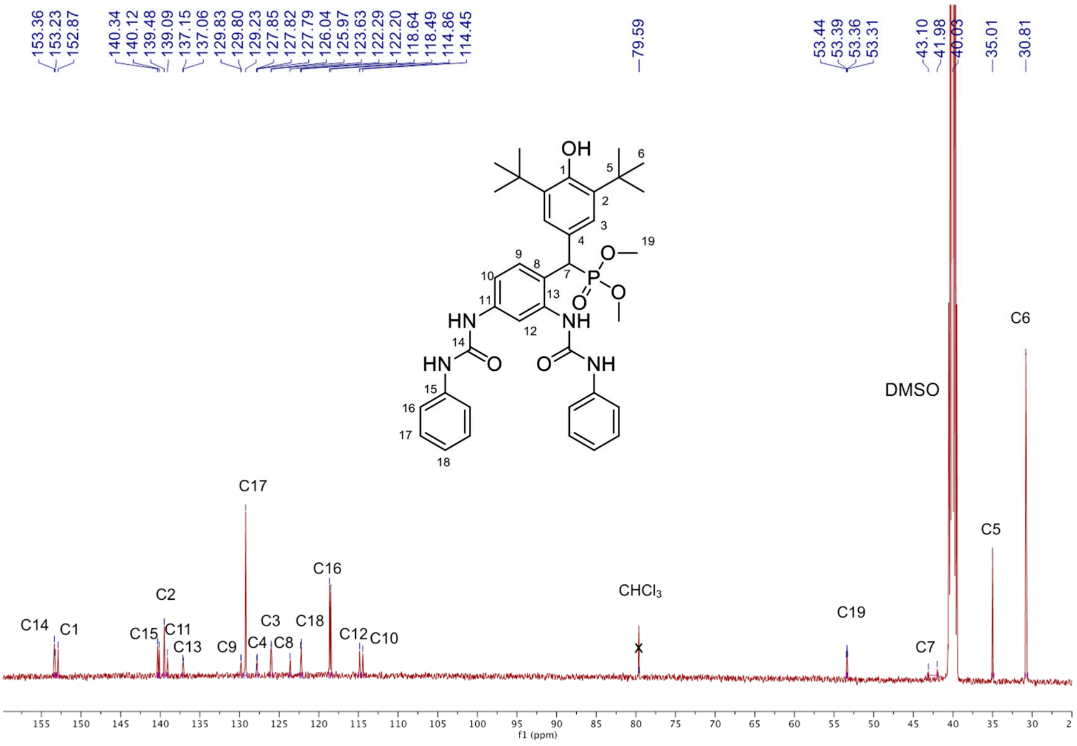

**Figure S17**. $^{13}$C- NMR of compound **6a**.

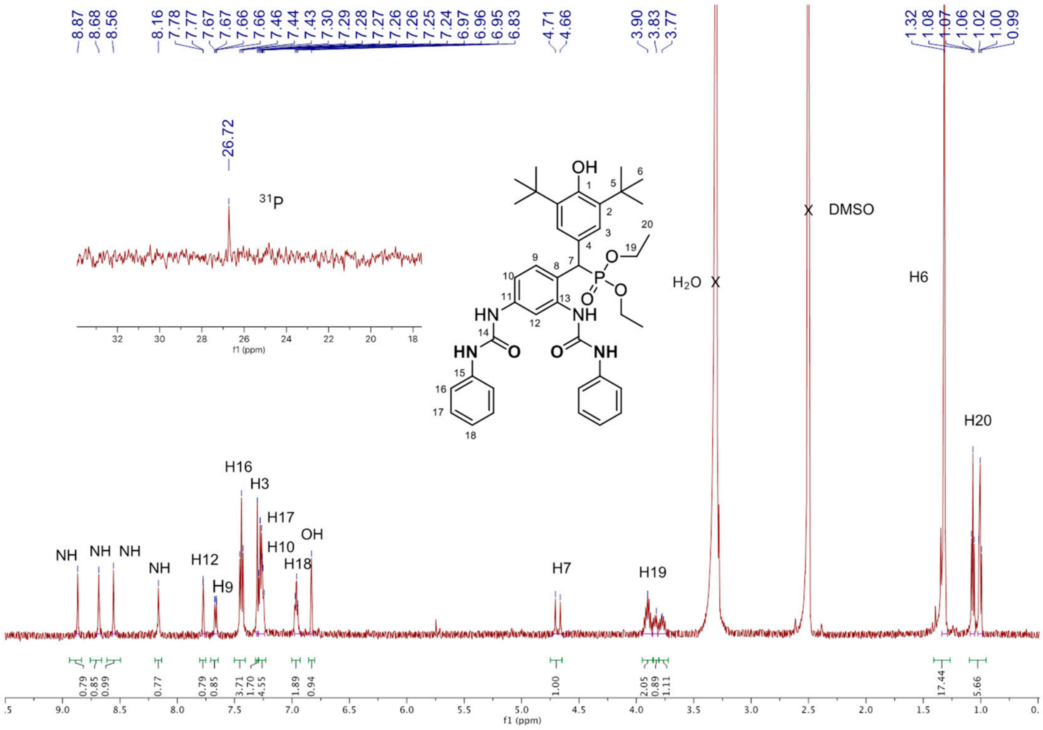


**Figure S18.** $^{1}H$-, $^{31}P$-NMR of compound **6b**.

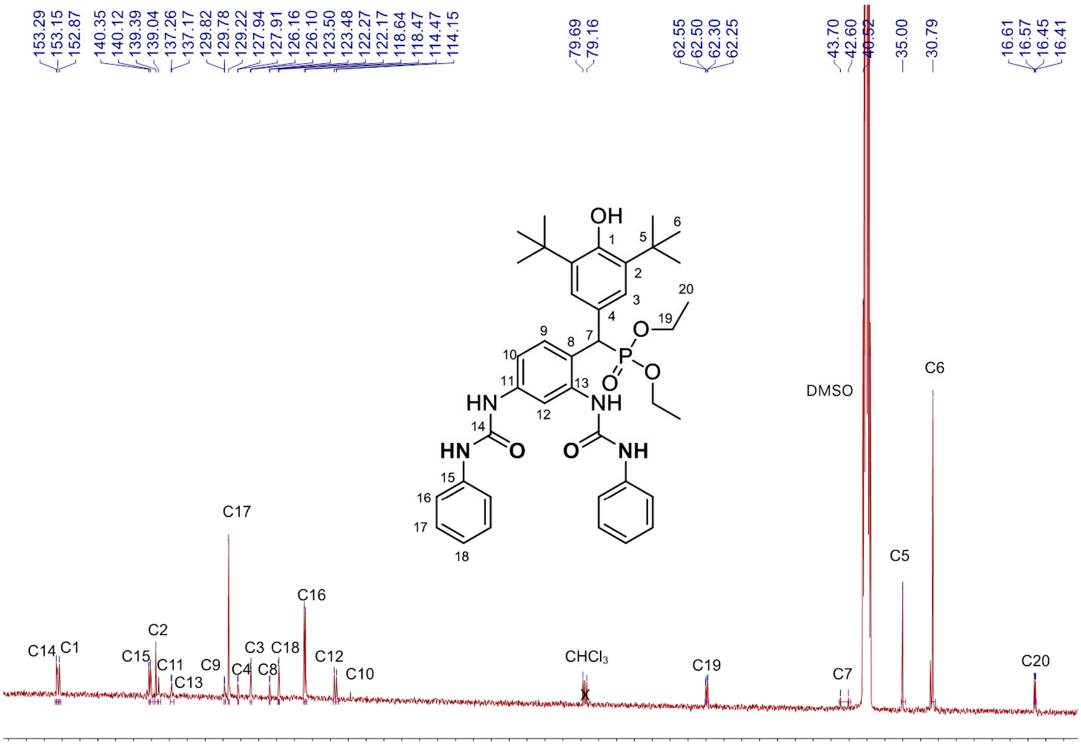


**Figure S19**. $^{13}C$-NMR of compound **6b**.

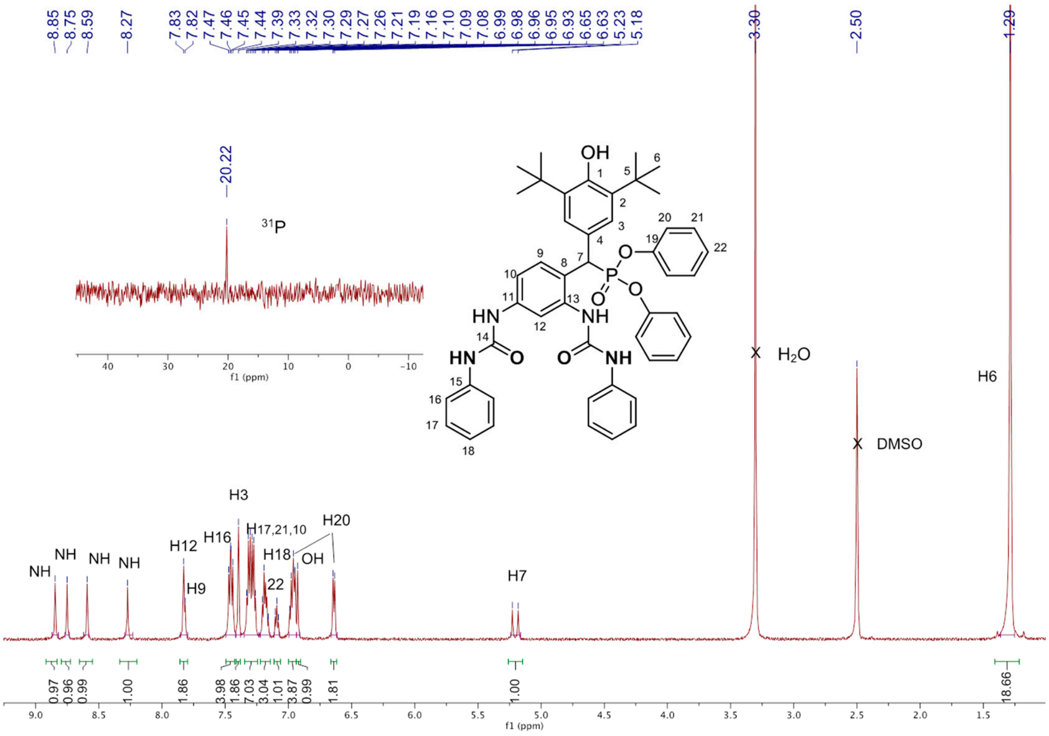

**Figure S20**. $^{1}$H-, $^{31}$P- NMR of compound **6d**.

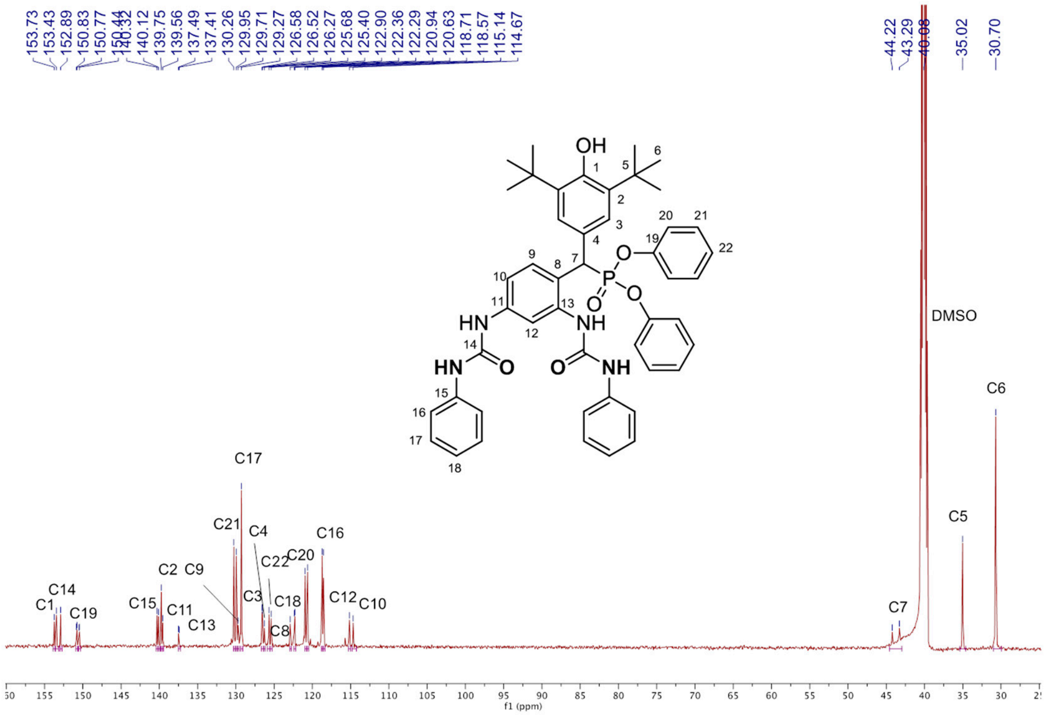

**Figure S21**. $^{13}$C-NMR of compound **6d**.

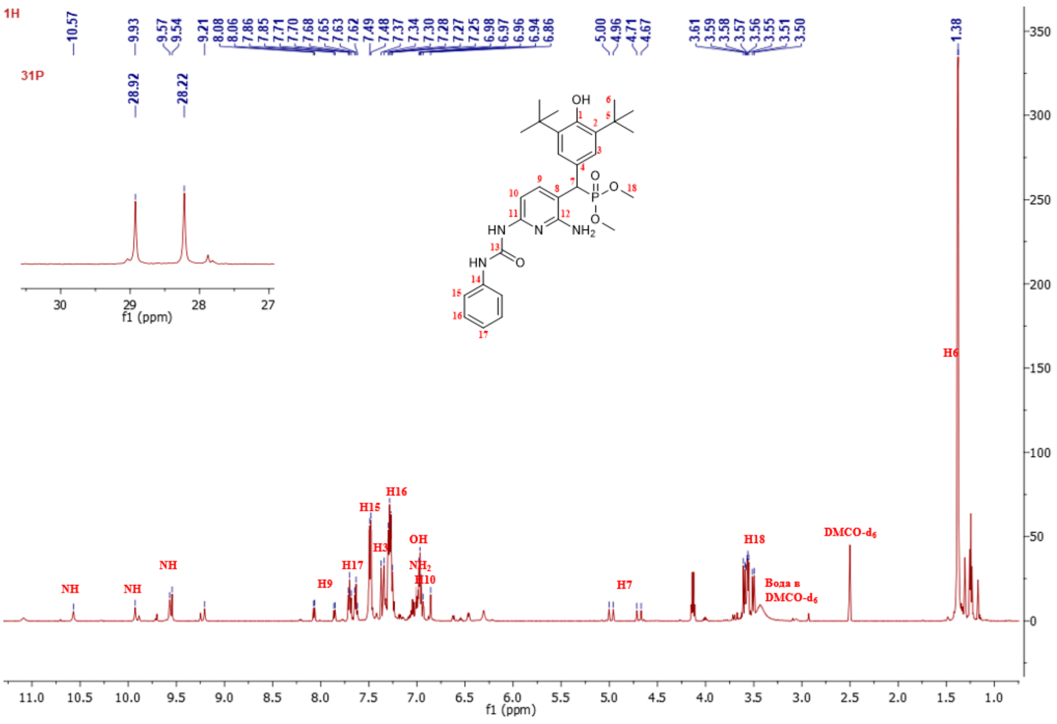

**Figure S22**. $^{1}$H-, $^{31}$P- NMR of compound **7a**.

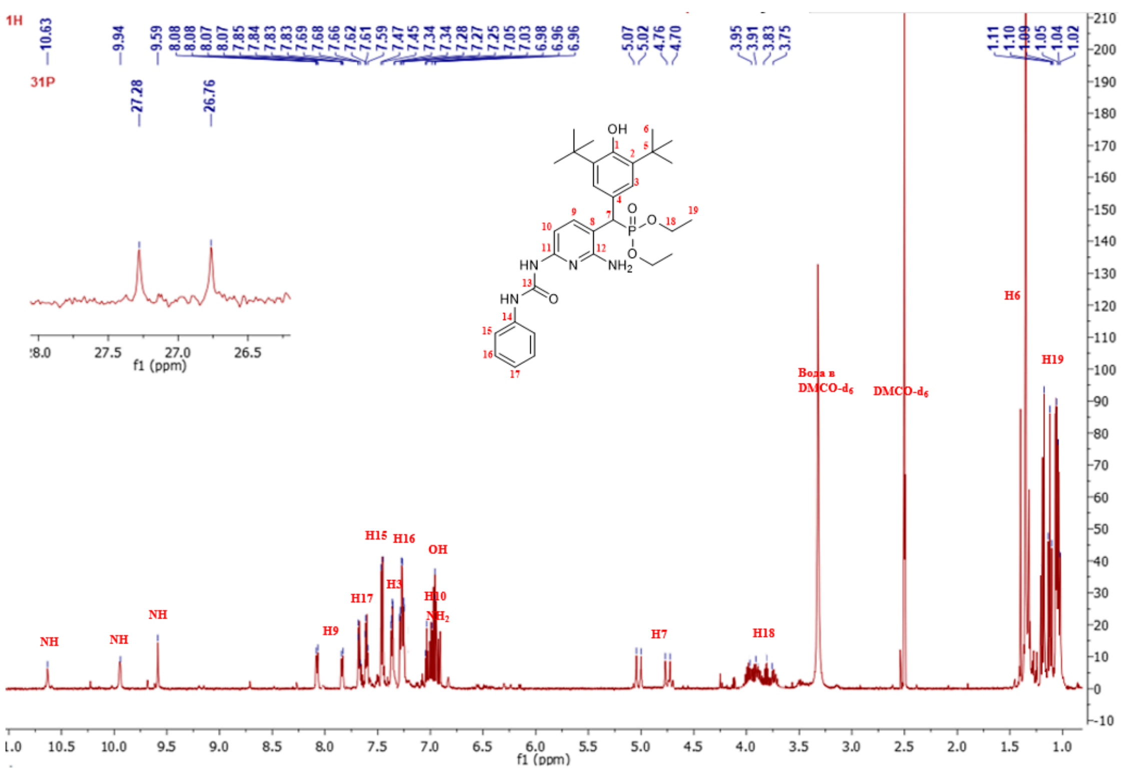

**Figure S23**. $^{1}$H-, $^{31}$P- NMR of compound **7b**

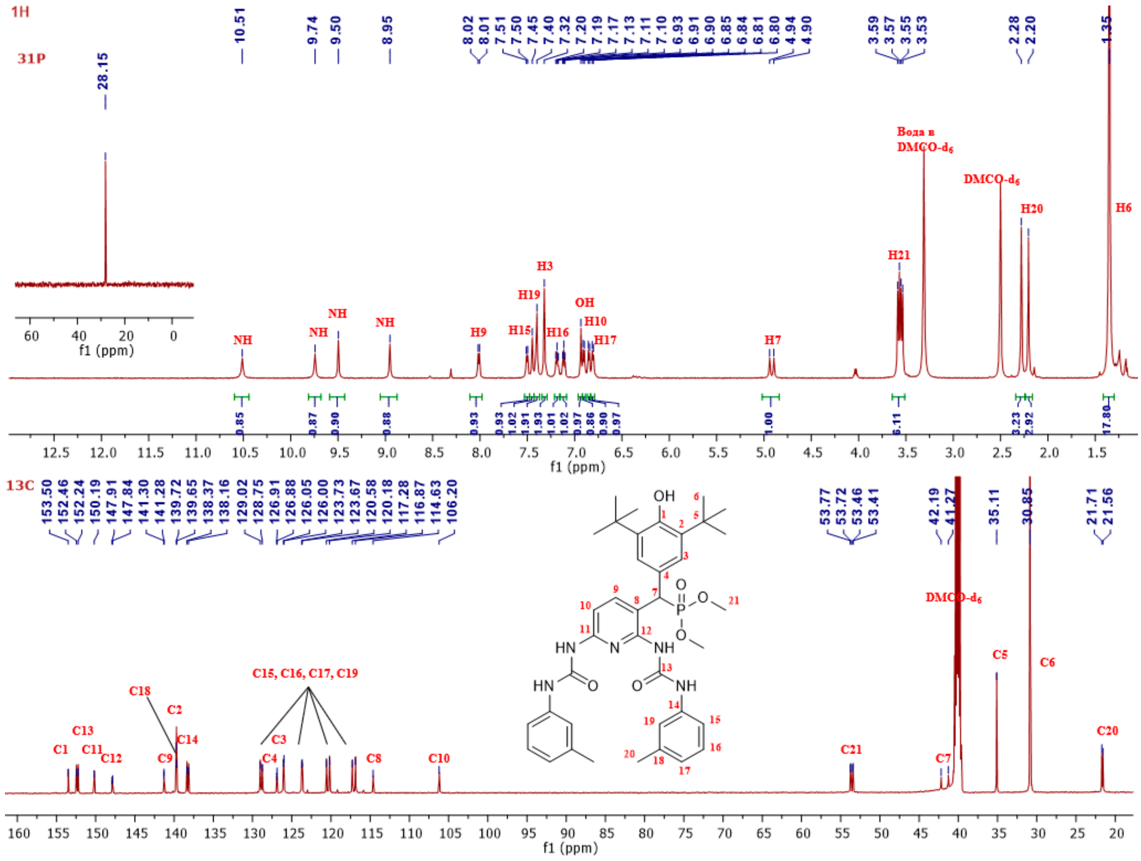

**Figure S24**. $^{1}$H-$^{31}$P-$^{13}$C- NMR of compound **8a**.

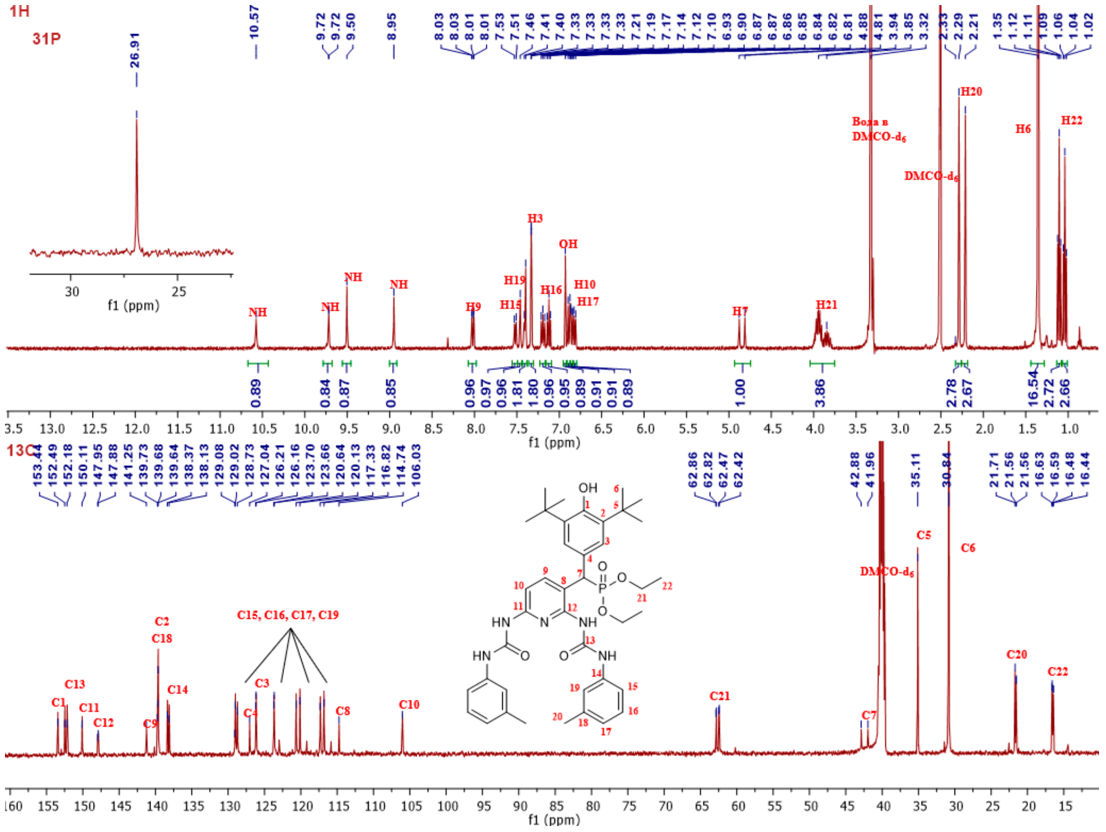

**Figure S25**. $^{1}$H-$^{31}$P-$^{13}$C- NMR of compound **8b**.

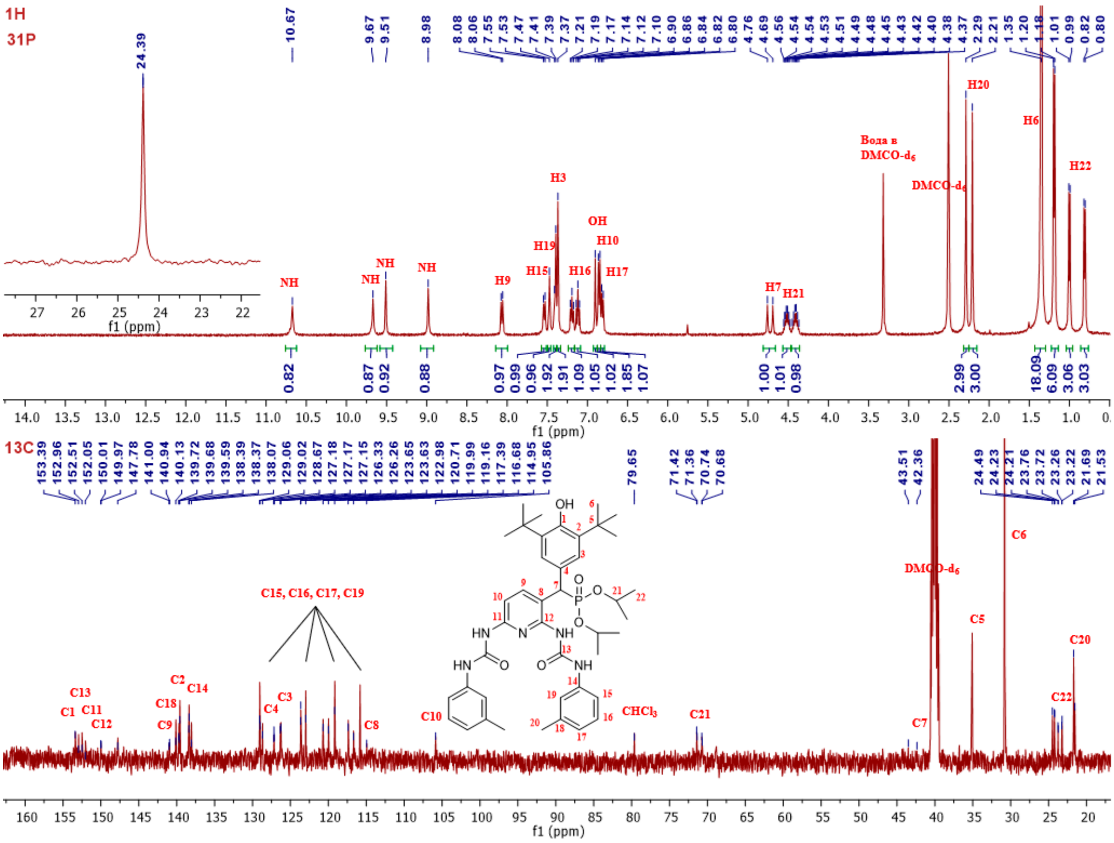

**Figure S26**. $^{1}H$-$^{31}P$-$^{13}C$- NMR of compound **8c**.

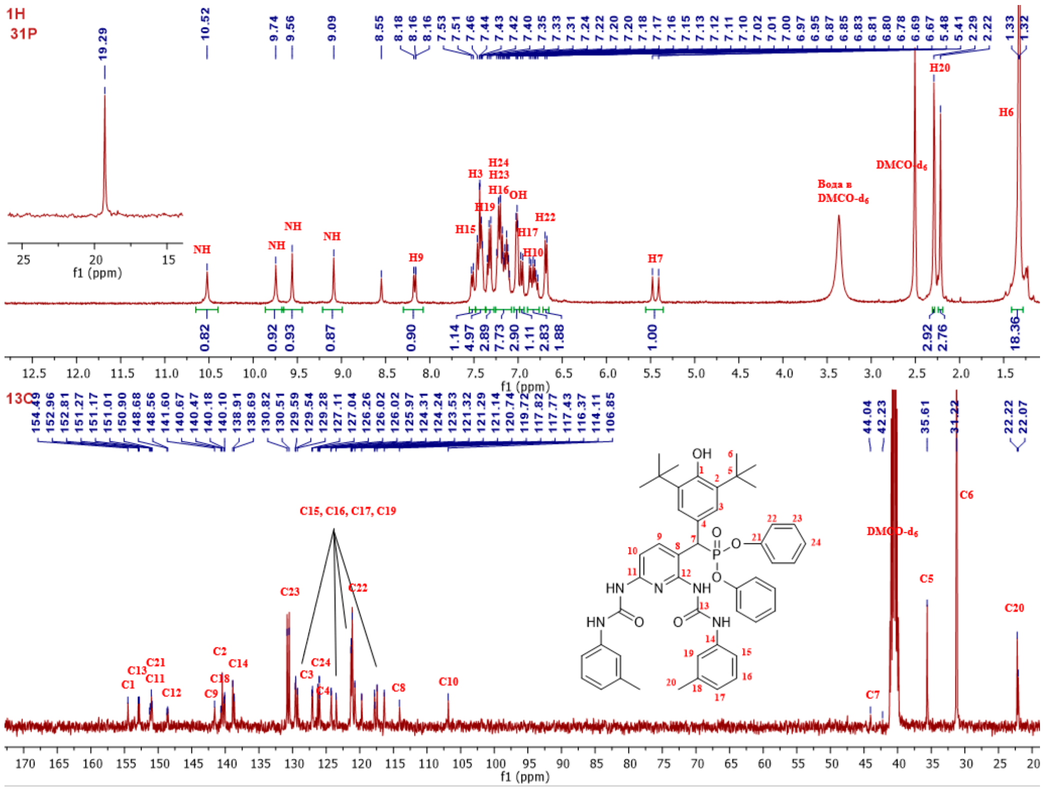

**Figure S27**. $^{1}H$-$^{31}P$-$^{13}C$- NMR of compound **8d**.

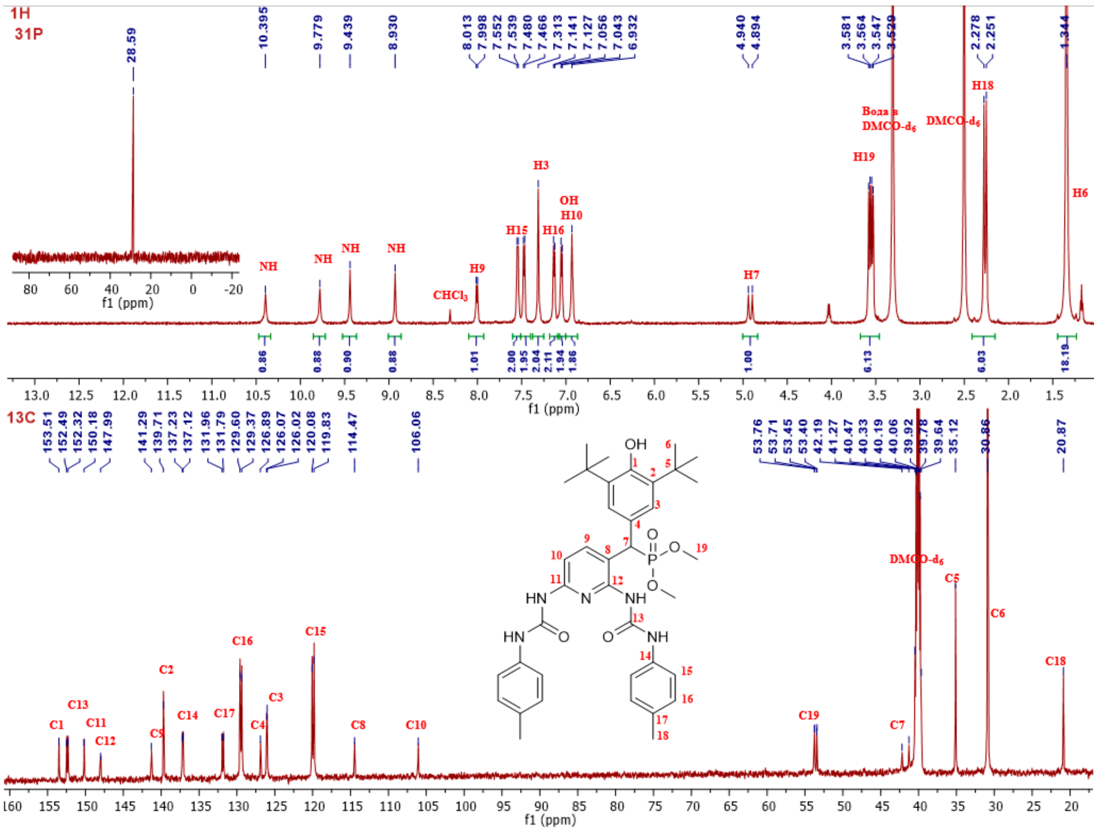

**Figure S28**. $^{1}H$-$^{31}P$-$^{13}C$- NMR of compound **9a**.

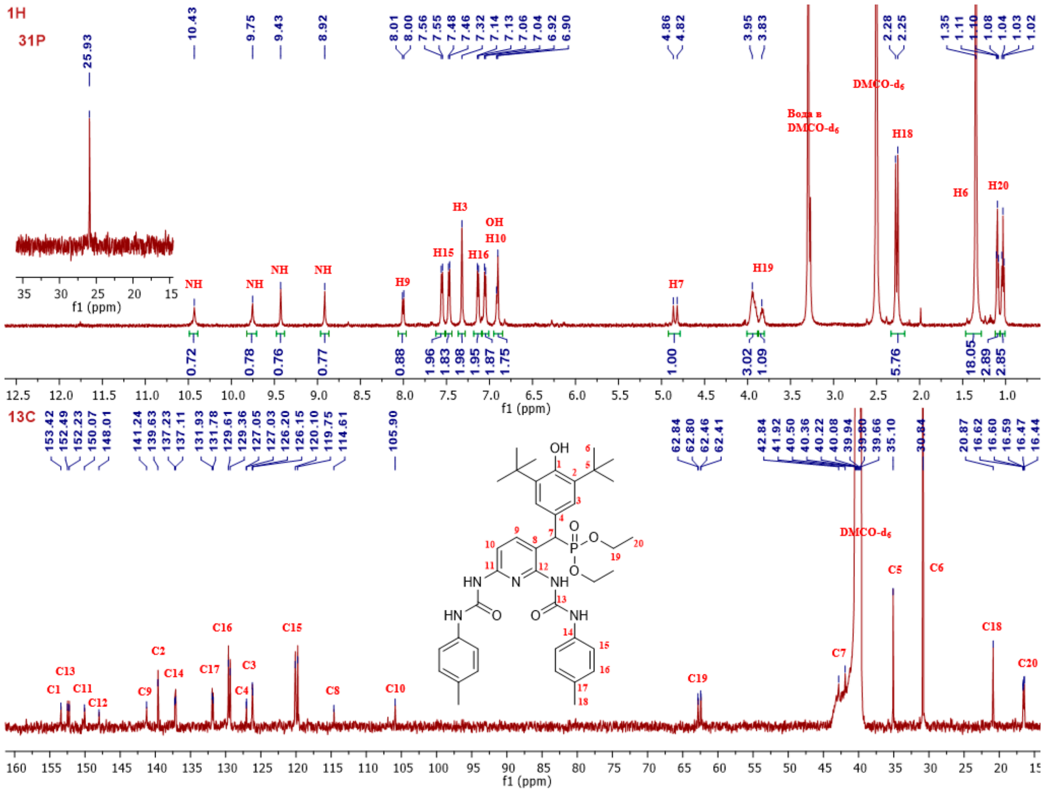

**Figure S29**. $^{1}H$-$^{31}P$-$^{13}C$- NMR of compound **9b**.

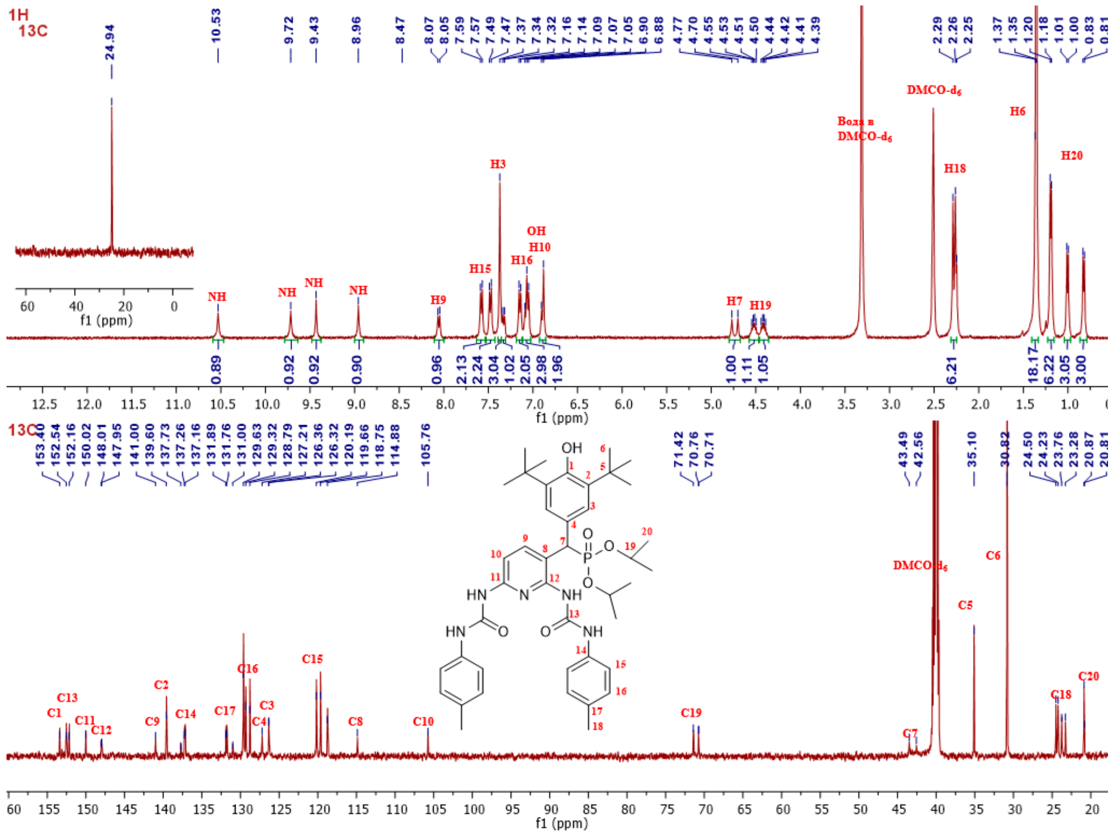

**Figure S30**. $^{1}H$-$^{31}P$-$^{13}C$- NMR of compound **9c**.

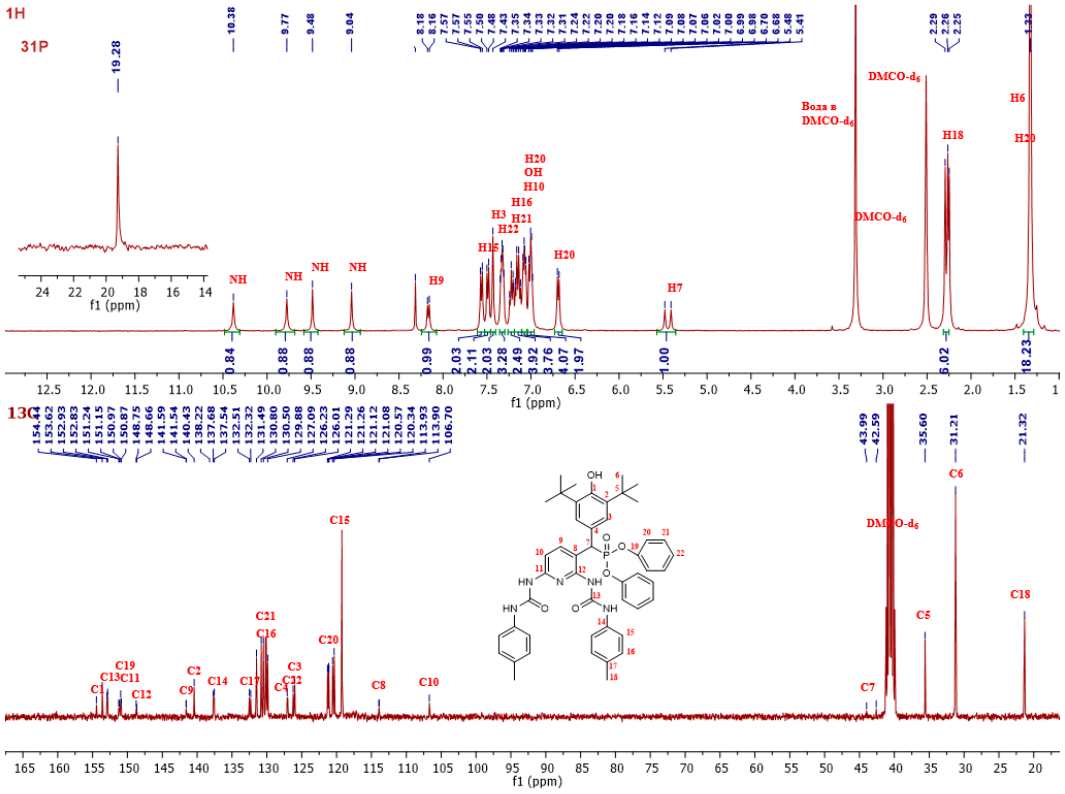

**Figure S31**. $^{1}H$-$^{31}P$-$^{13}C$- NMR of compound **9d**.

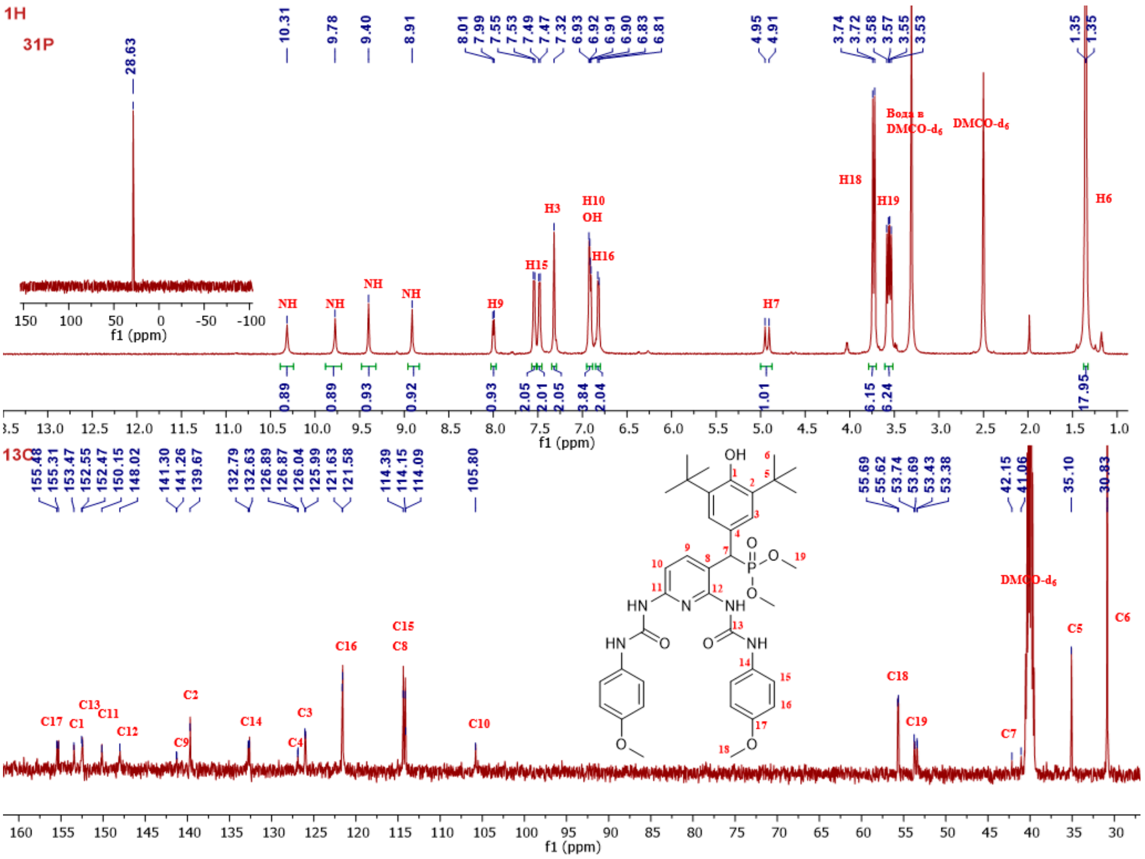

**Figure S32**. $^{1}$H-,$^{31}$P-,$^{13}$C- NMR of compound **10a**.

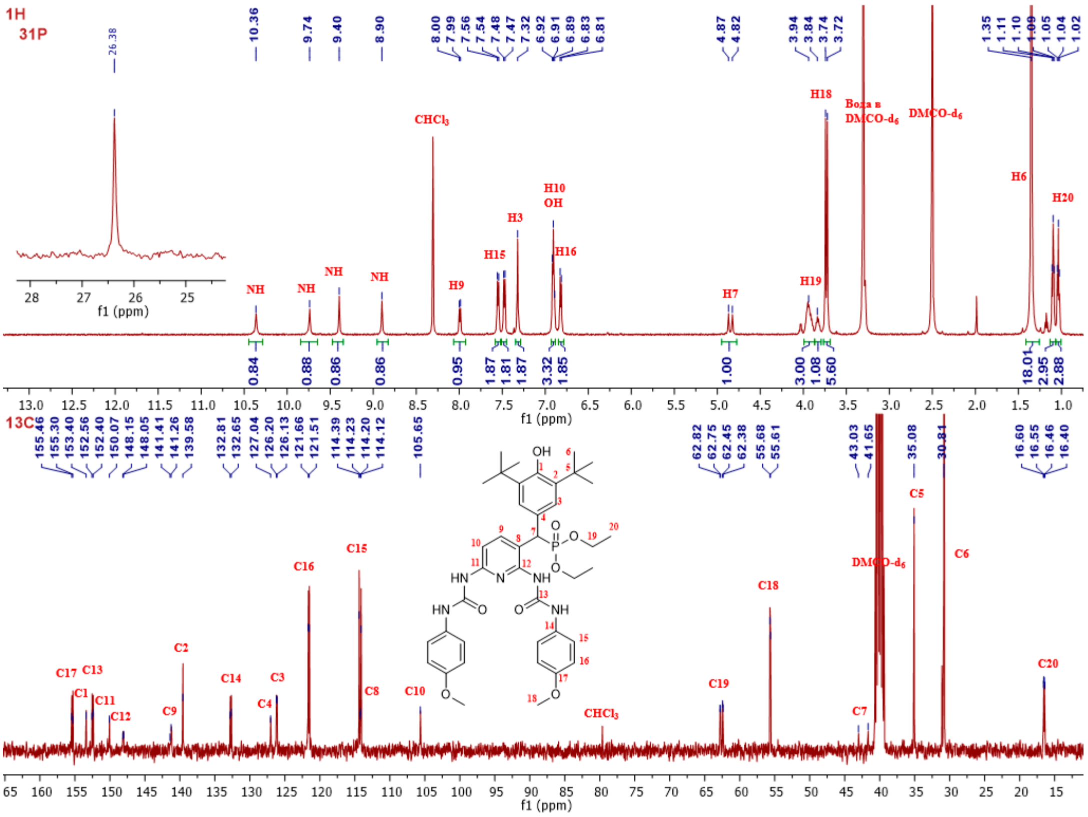

**Figure S33**. $^{1}$H-,$^{31}$P-,$^{13}$C- NMR of compound **10b**.

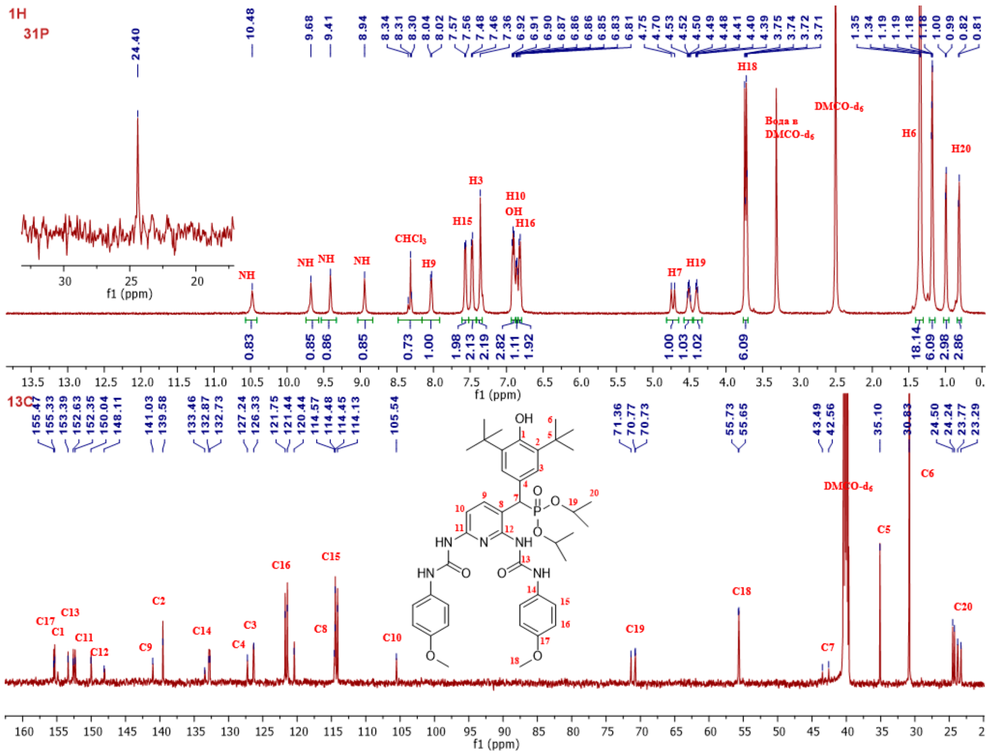


**Figure S34**. $^{1}H$-,$^{31}P$-,$^{13}C$- NMR of compound **10c**.

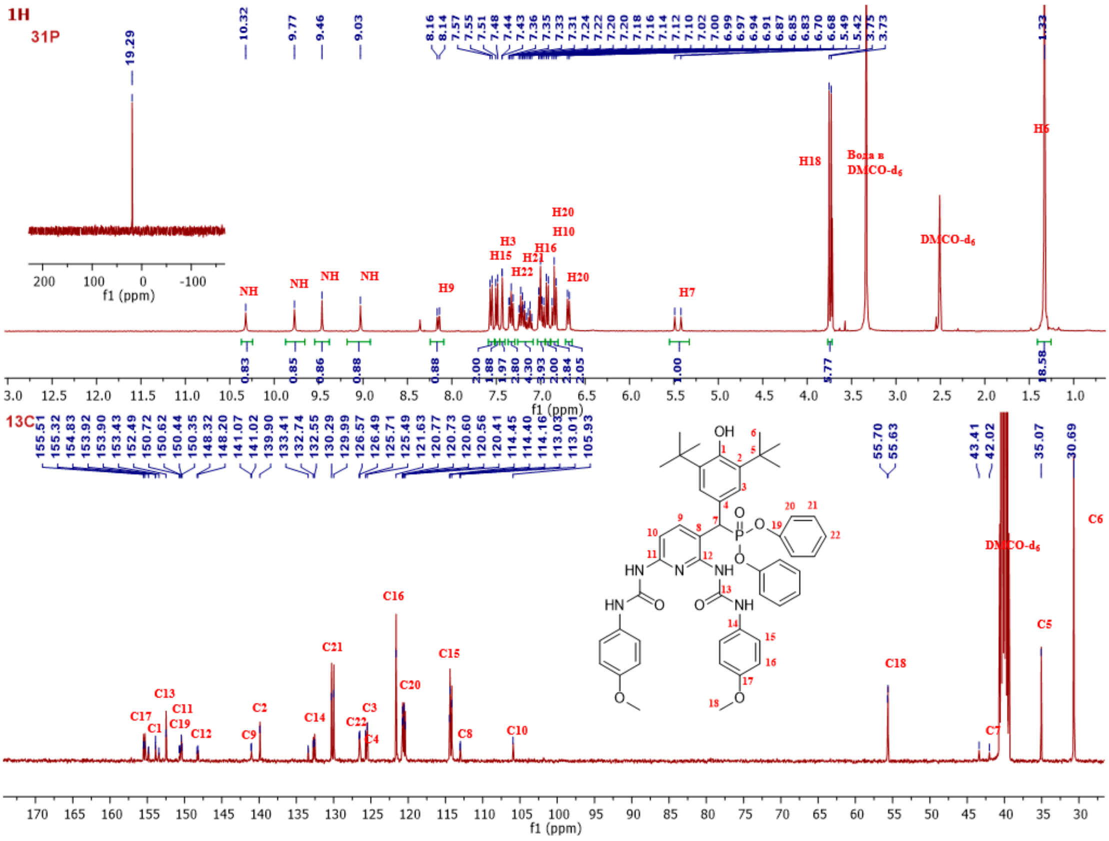


**Figure S35**. $^{1}H$-,$^{31}P$-,$^{13}C$- NMR of compound **10d**.

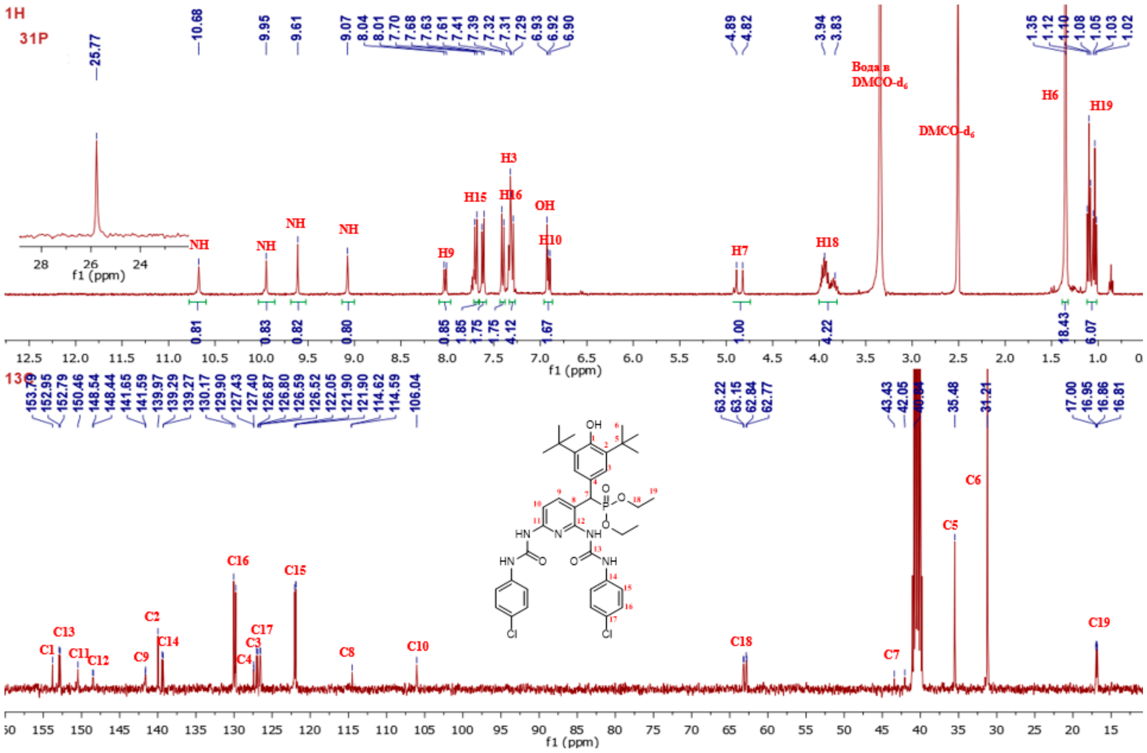


**Figure S36**. ¹H-,³¹P-,¹³C- NMR of compound **11b**.

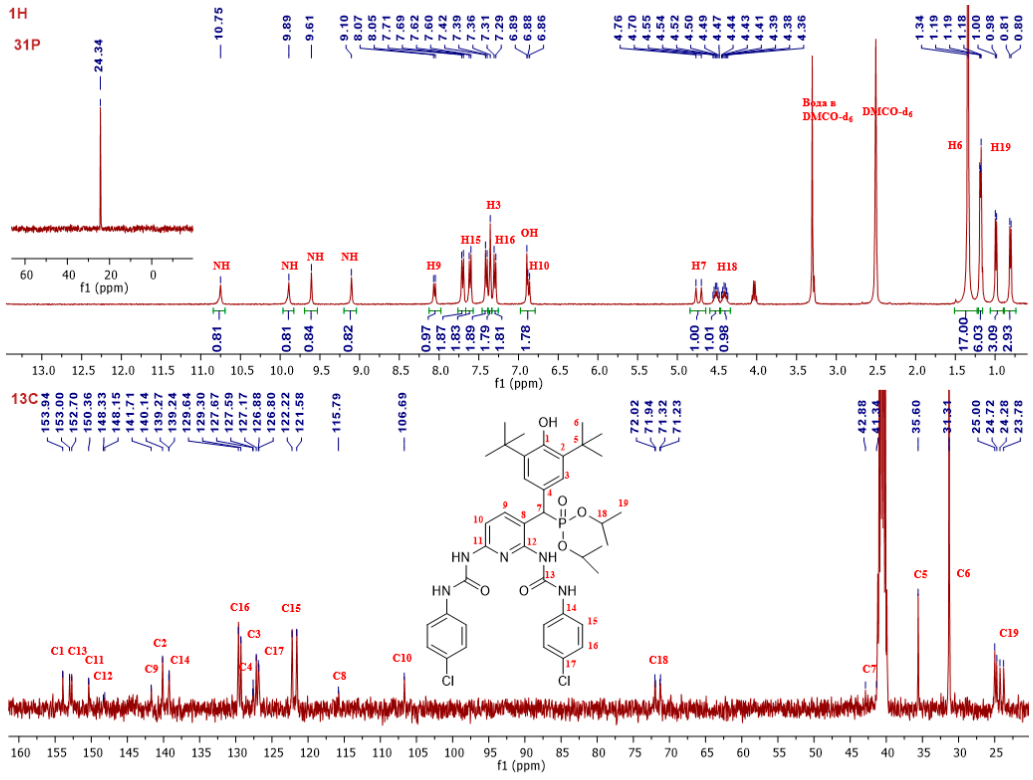


**Figure S37**. ¹H-,³¹P-,¹³C- NMR of compound **11c**.

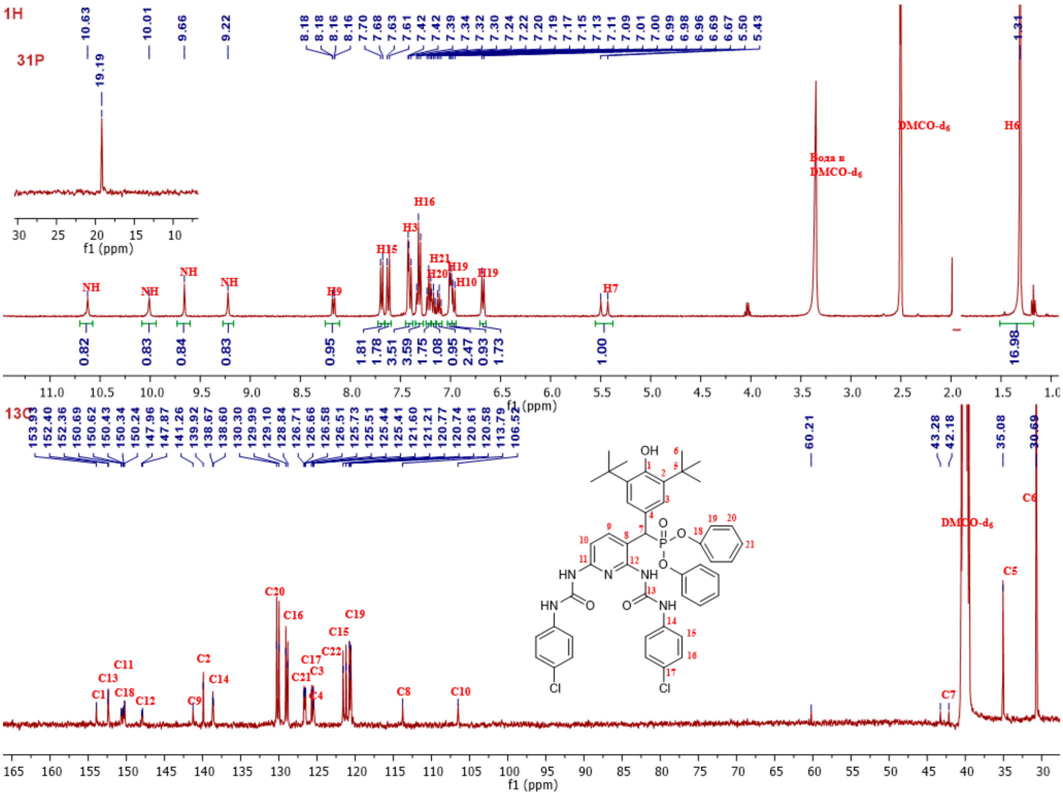


**Figure S38**. $^{1}$H-,$^{31}$P-,$^{13}$C- NMR of compound **11d**.

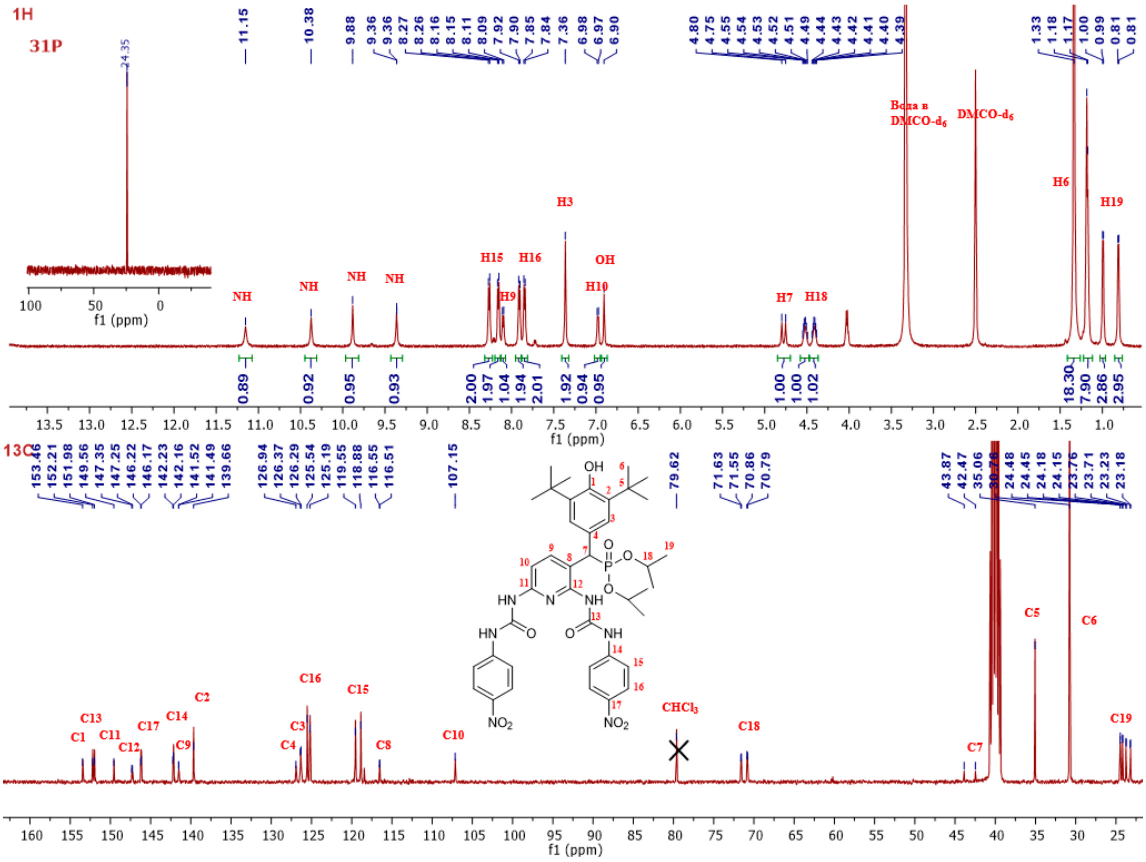


**Figure S39**. $^{1}$H-,$^{31}$P-,$^{13}$C- NMR of compound **12c**.

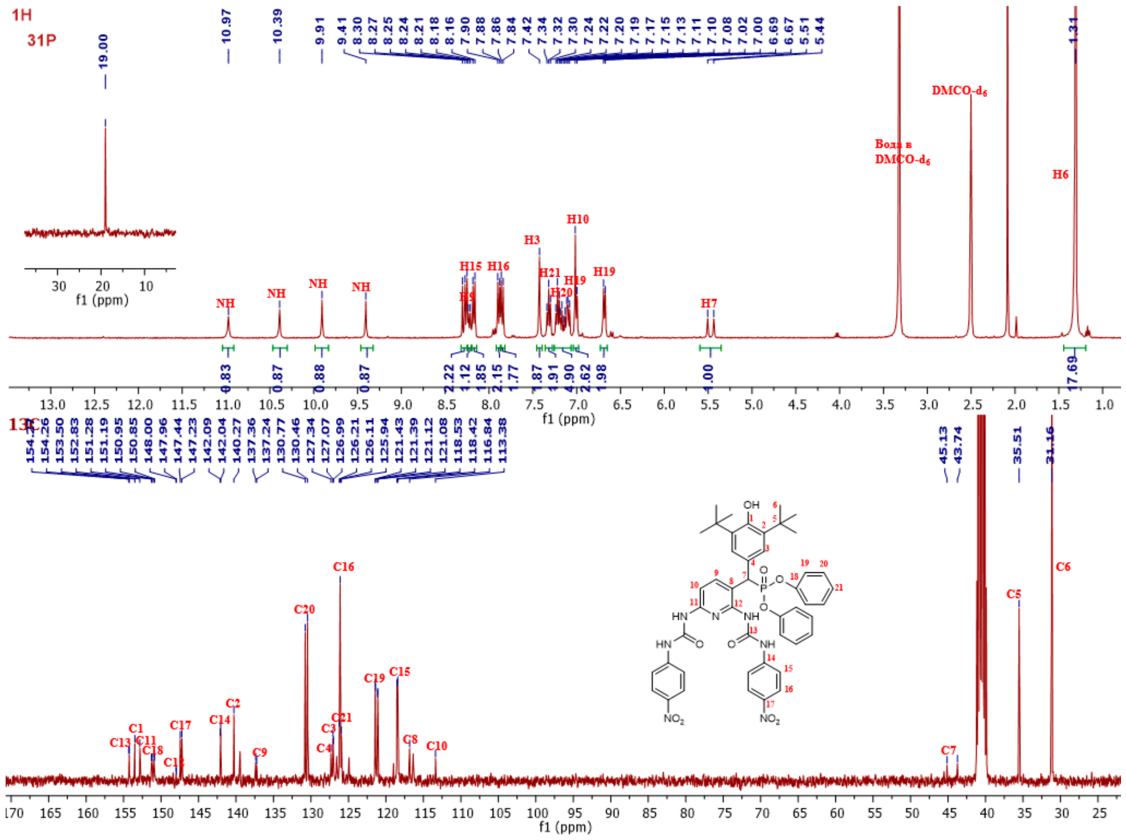

**Figure S40**. $^{1}$H-,$^{31}$P-,$^{13}$C- NMR of compound **12d**.

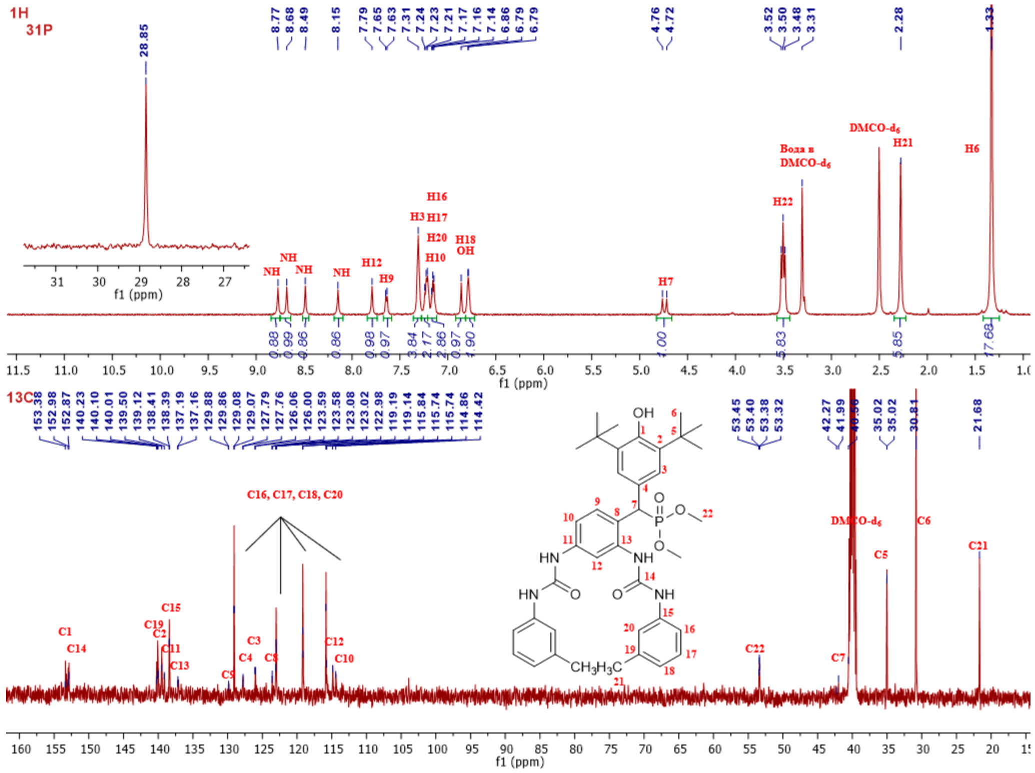

**Figure S41**. $^{1}$H-,$^{31}$P-,$^{13}$C- NMR of compound **13a**.

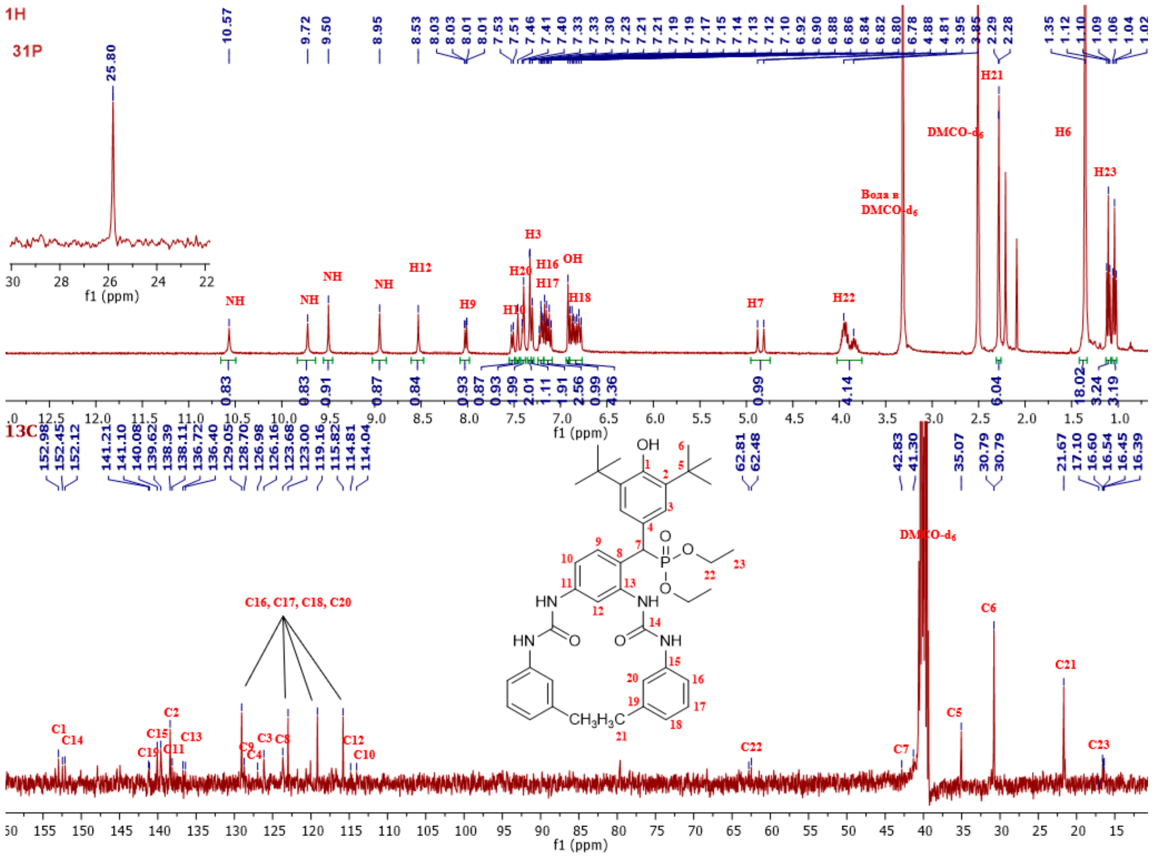

**Figure S42**. ¹H-,³¹P-,¹³C- NMR of compound **13b**.

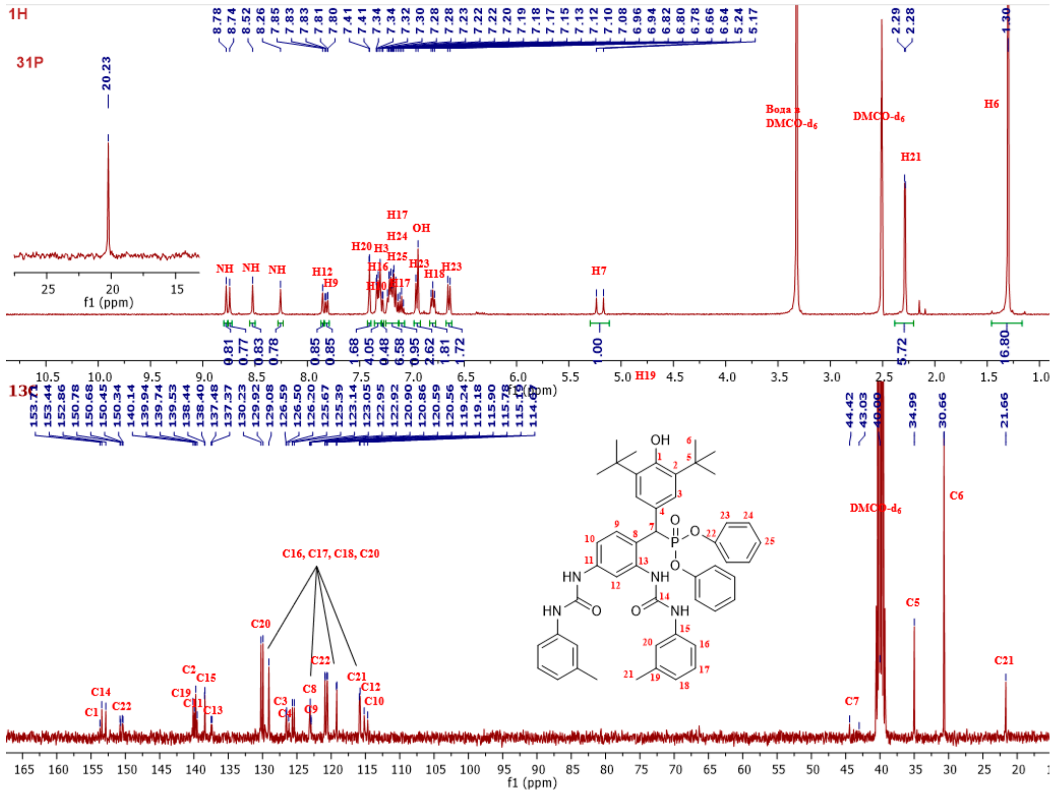

**Figure S43**. ¹H-,³¹P-,¹³C- NMR of compound **13d**.

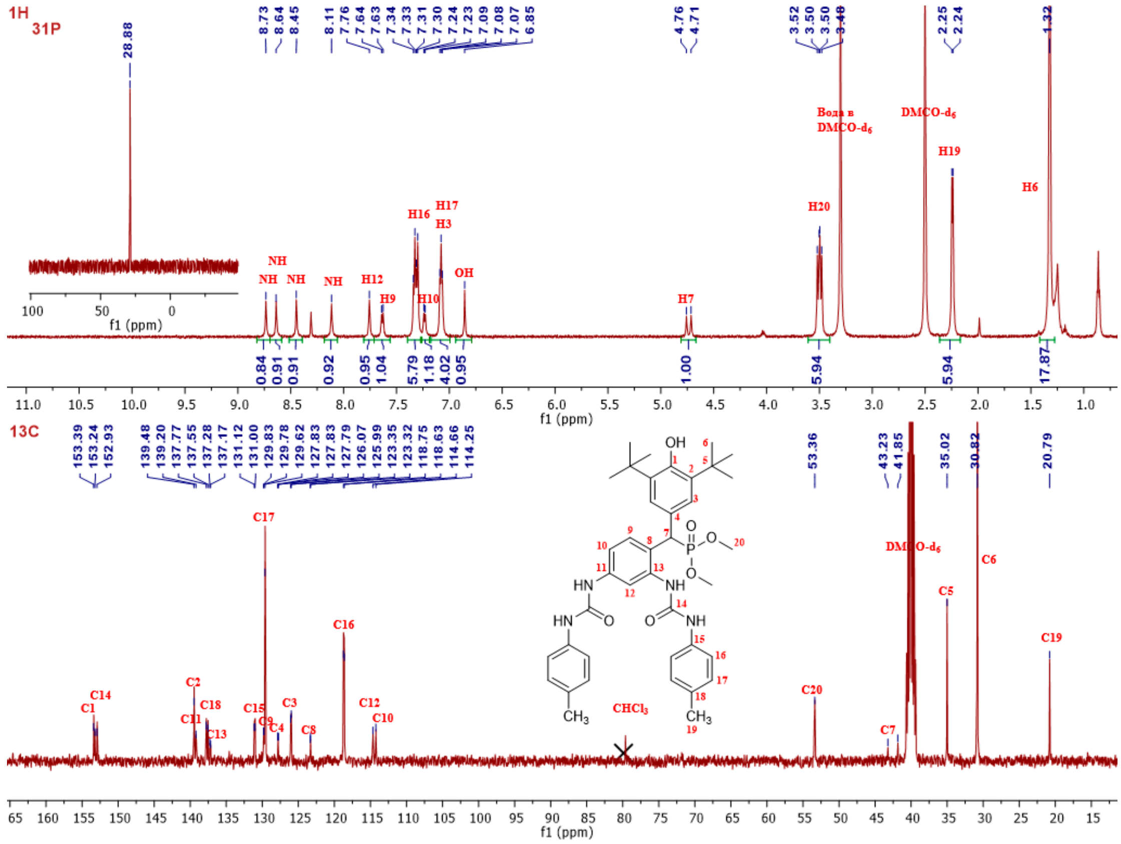

**Figure S44**. $^{1}$H-,$^{31}$P-,$^{13}$C- NMR of compound **14a**.

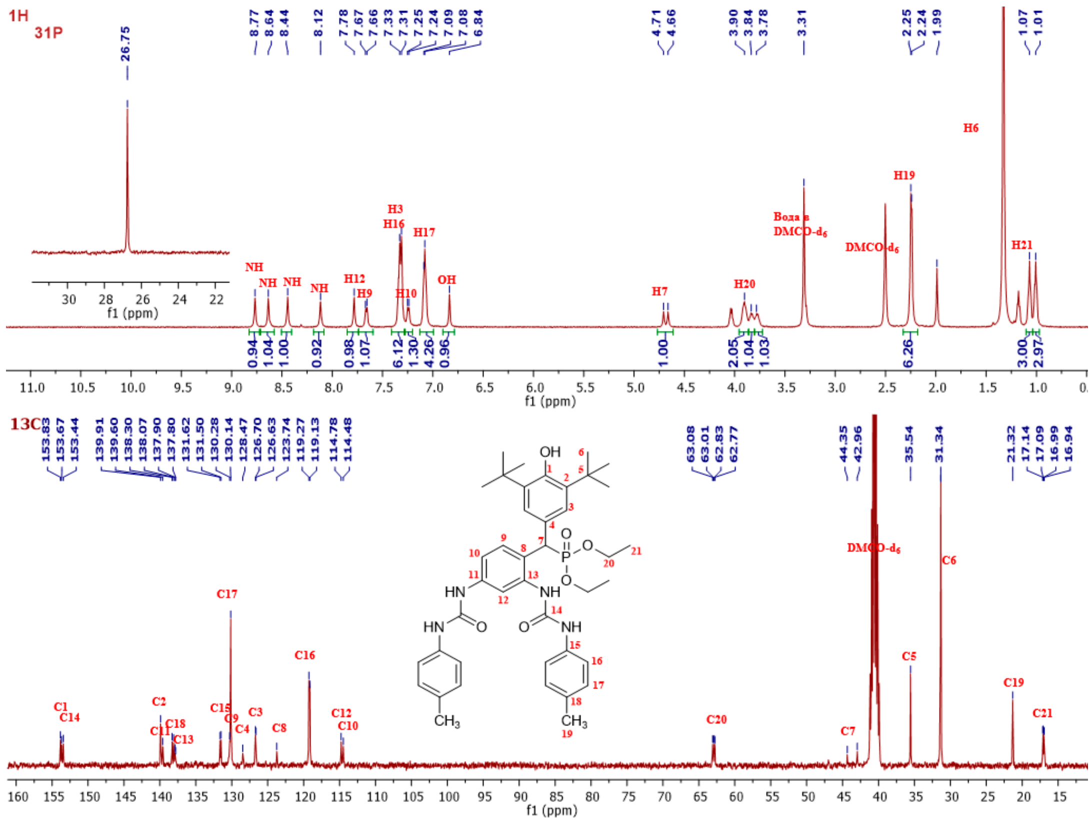

**Figure S45**. $^{1}$H-,$^{31}$P-,$^{13}$C- NMR of compound **14b**.

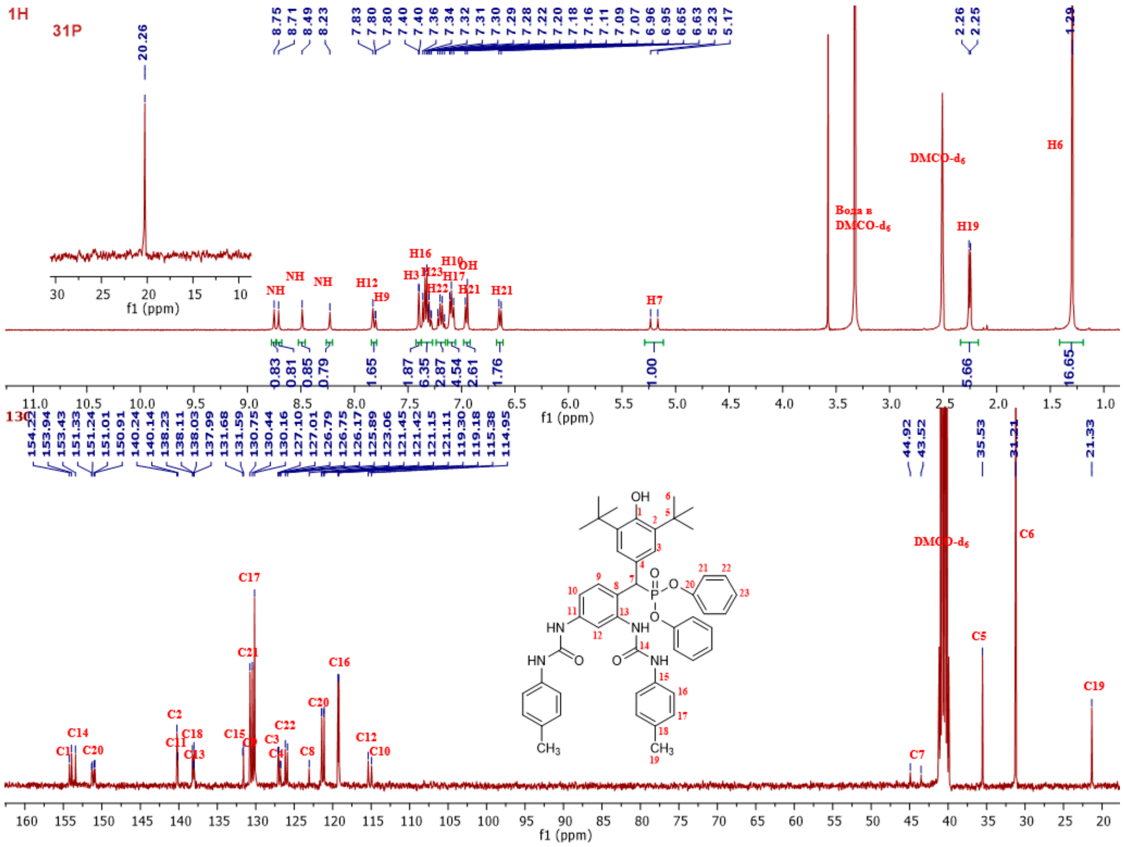

**Figure S46**. $^{1}H$-,$^{31}P$-,$^{13}C$- NMR of compound **14d**.

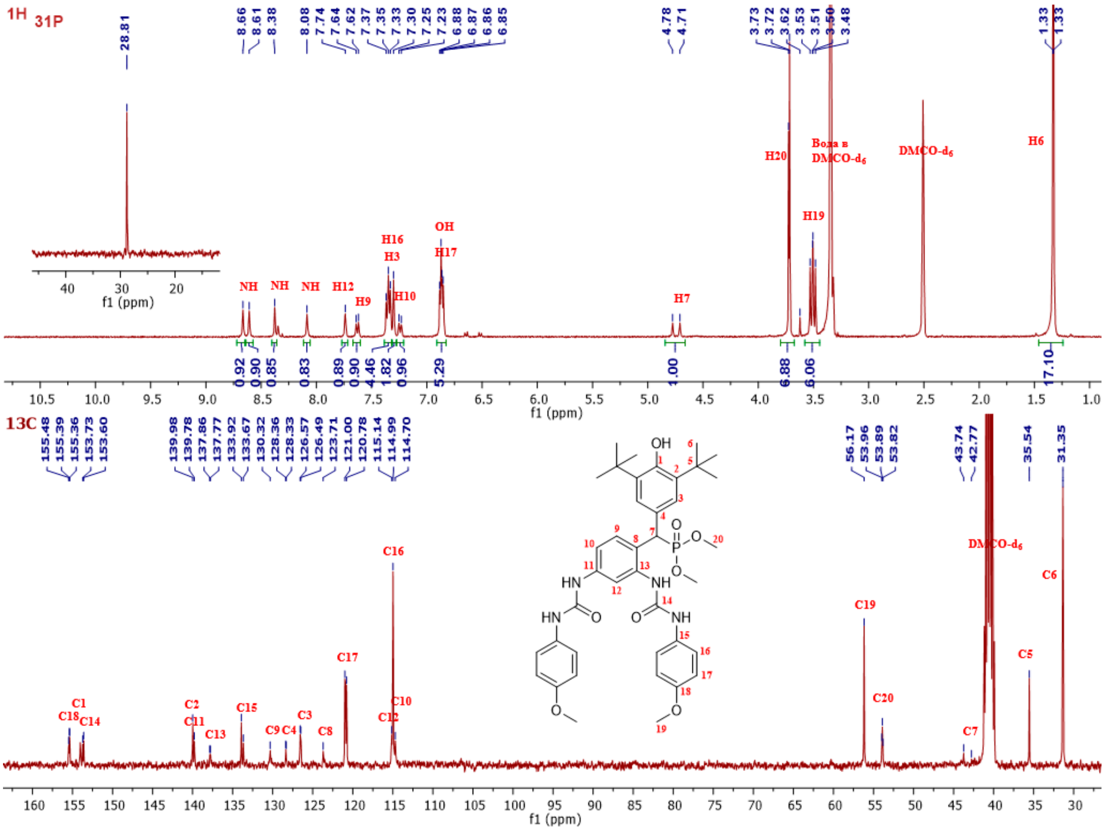

**Figure S47**. $^{1}H$-,$^{31}P$-,$^{13}C$- NMR of compound **15a**.

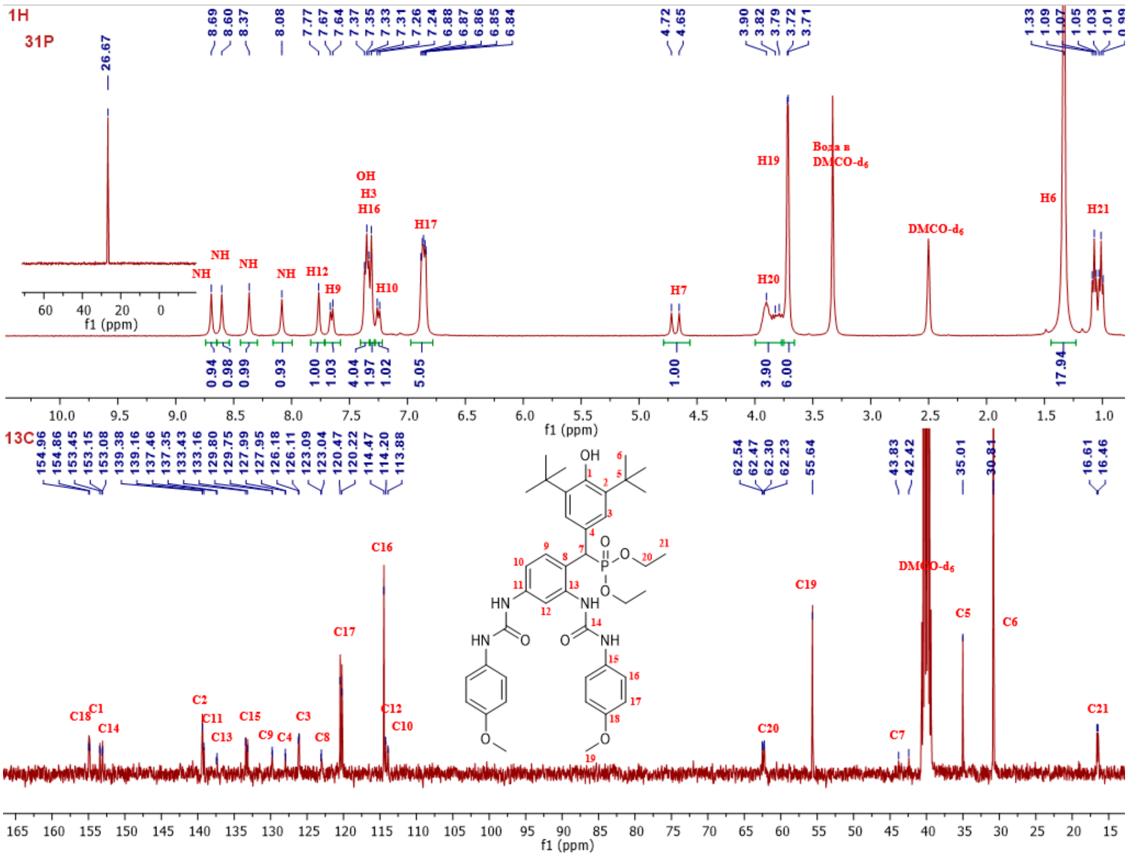

**Figure S48**. $^{1}H$-,$^{31}P$-,$^{13}C$- NMR of compound **15b**.

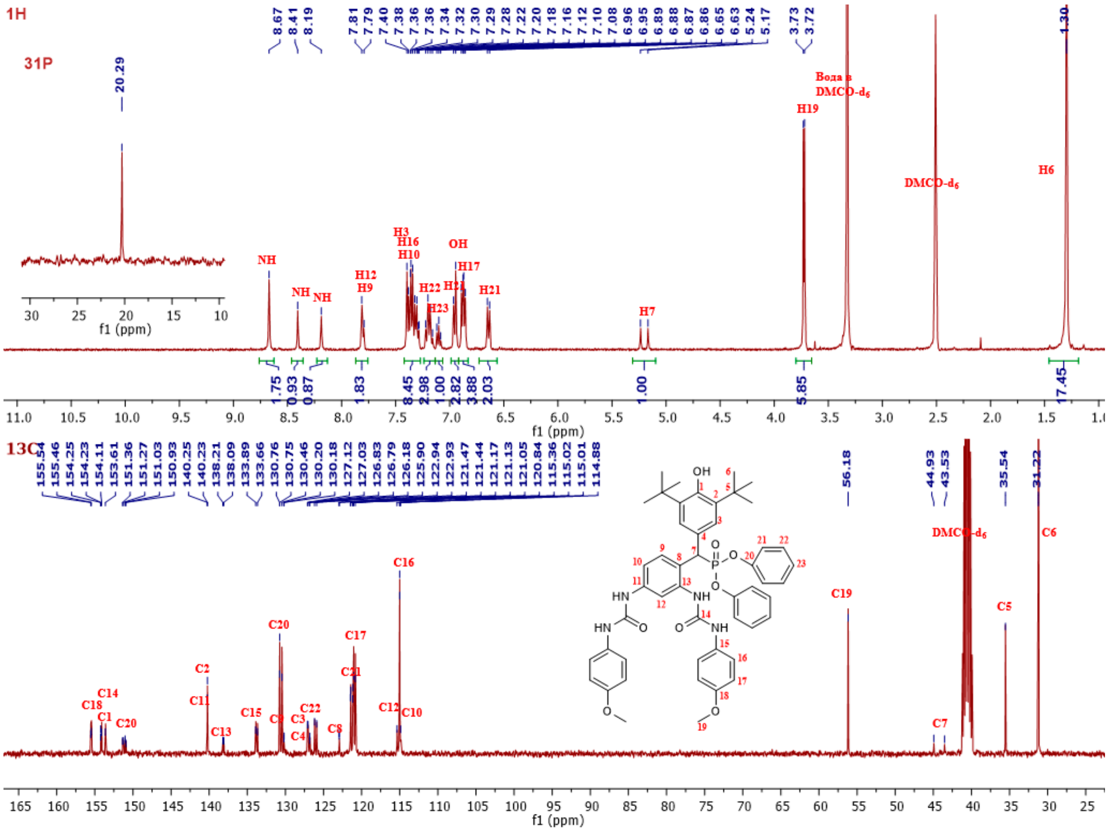

**Figure S49**. $^{1}H$-,$^{31}P$-,$^{13}C$- NMR of compound **15d**.

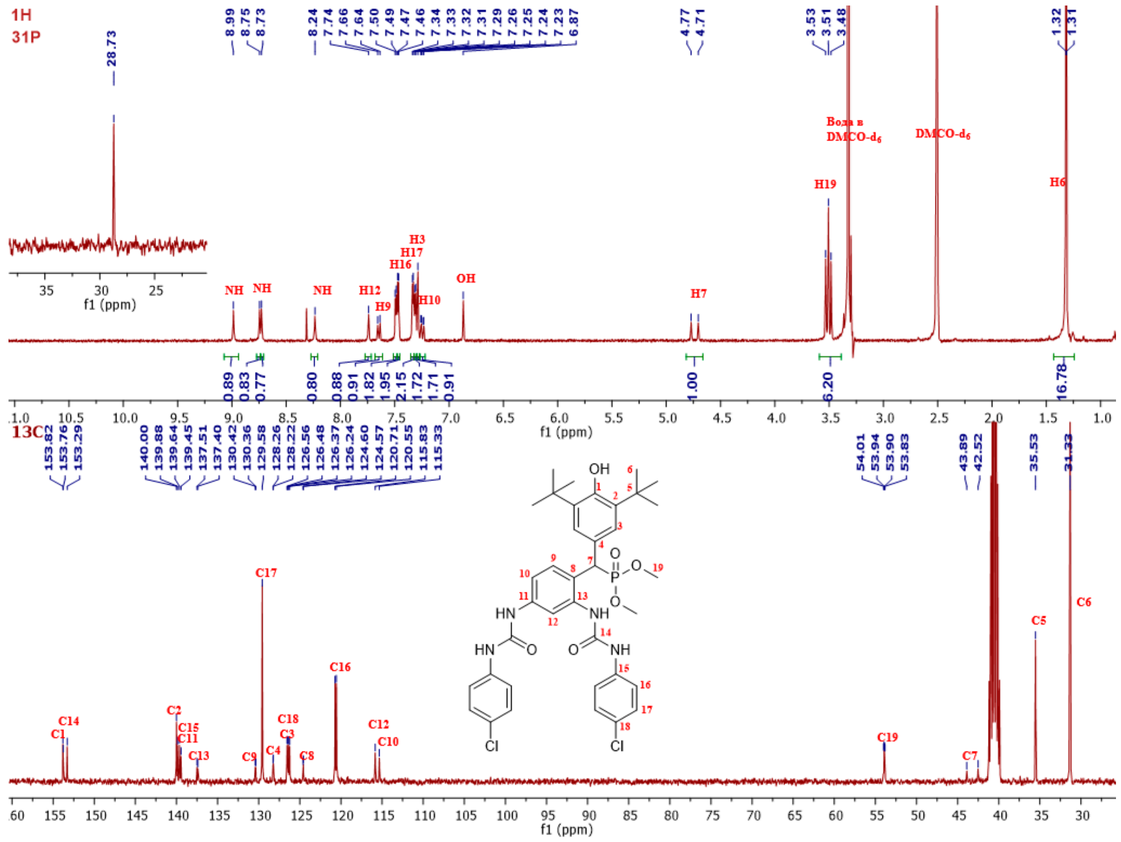

**Figure S50**. $^{1}$H-,$^{31}$P-,$^{13}$C- NMR of compound **16a**

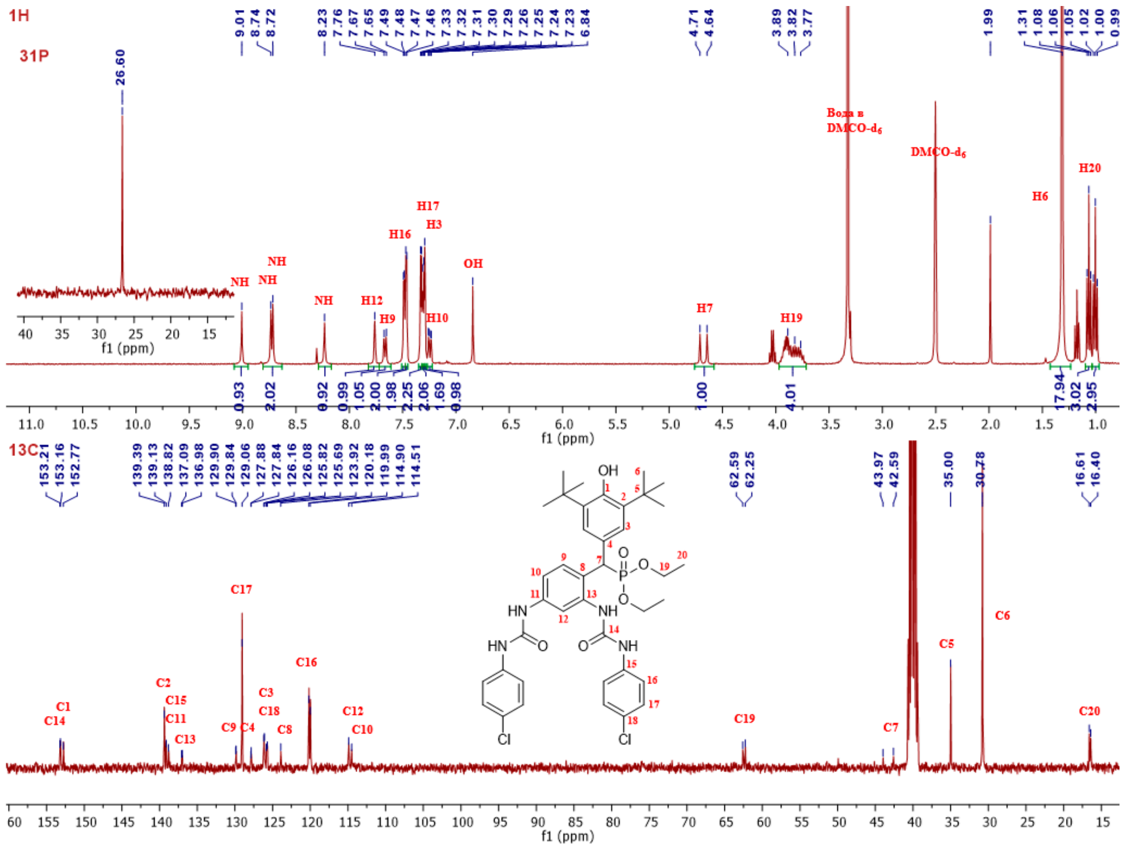

**Figure S51**. $^{1}$H-,$^{31}$P-,$^{13}$C- NMR of compound **16b**

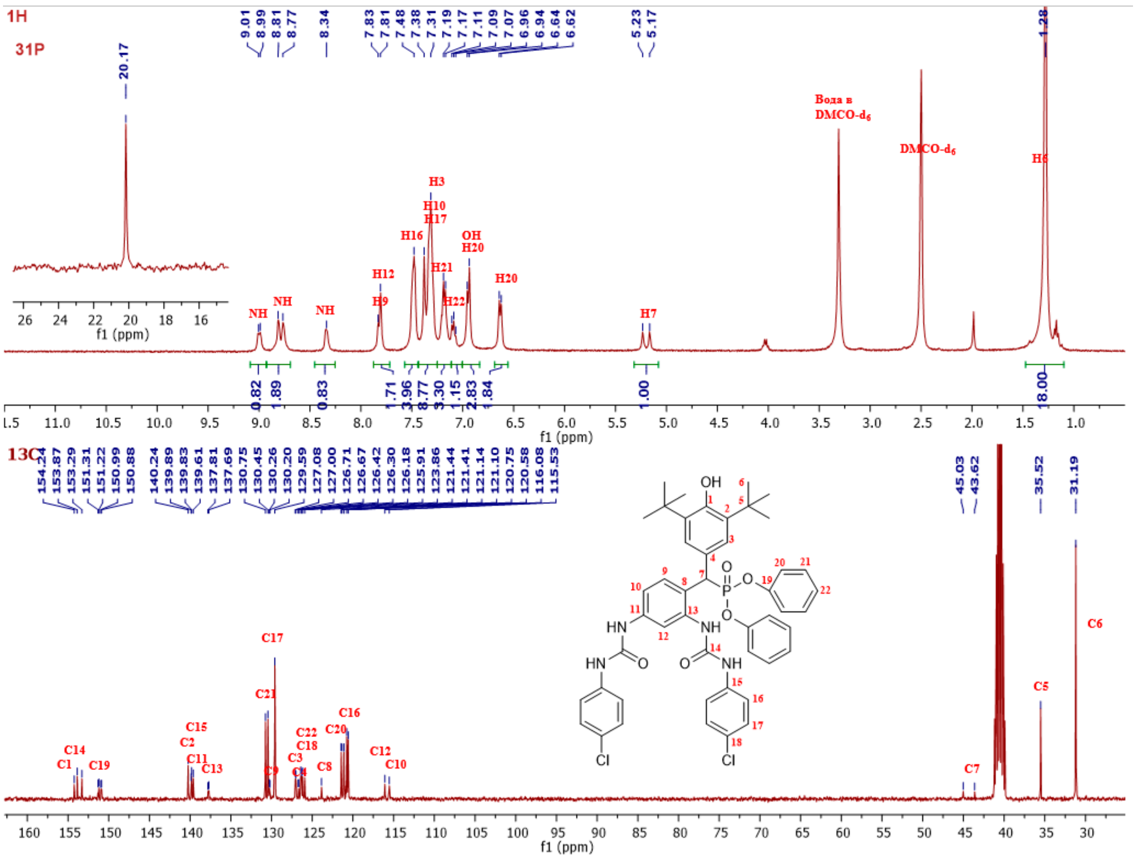

**Figure S52**. $^{1}H$-,$^{31}P$-,$^{13}C$- NMR of compound **16d**

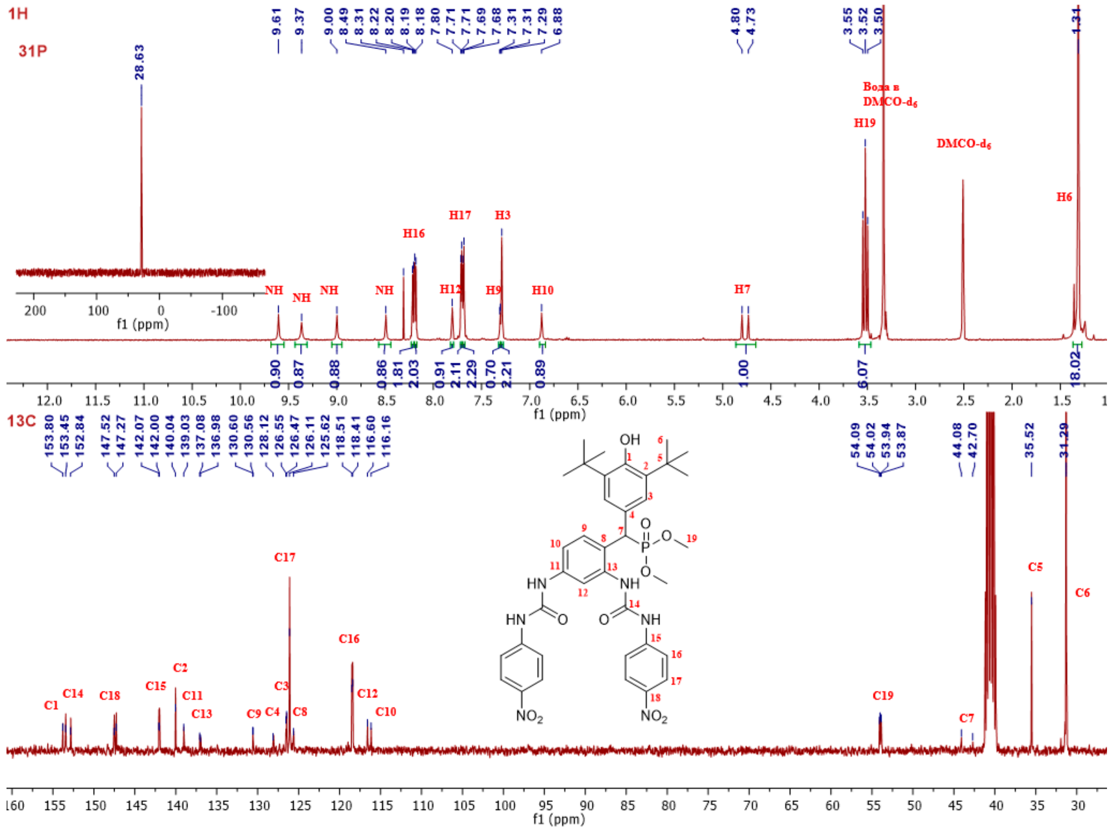

**Figure S53**. $^{1}H$-,$^{31}P$-,$^{13}C$- NMR of compound **17a**.

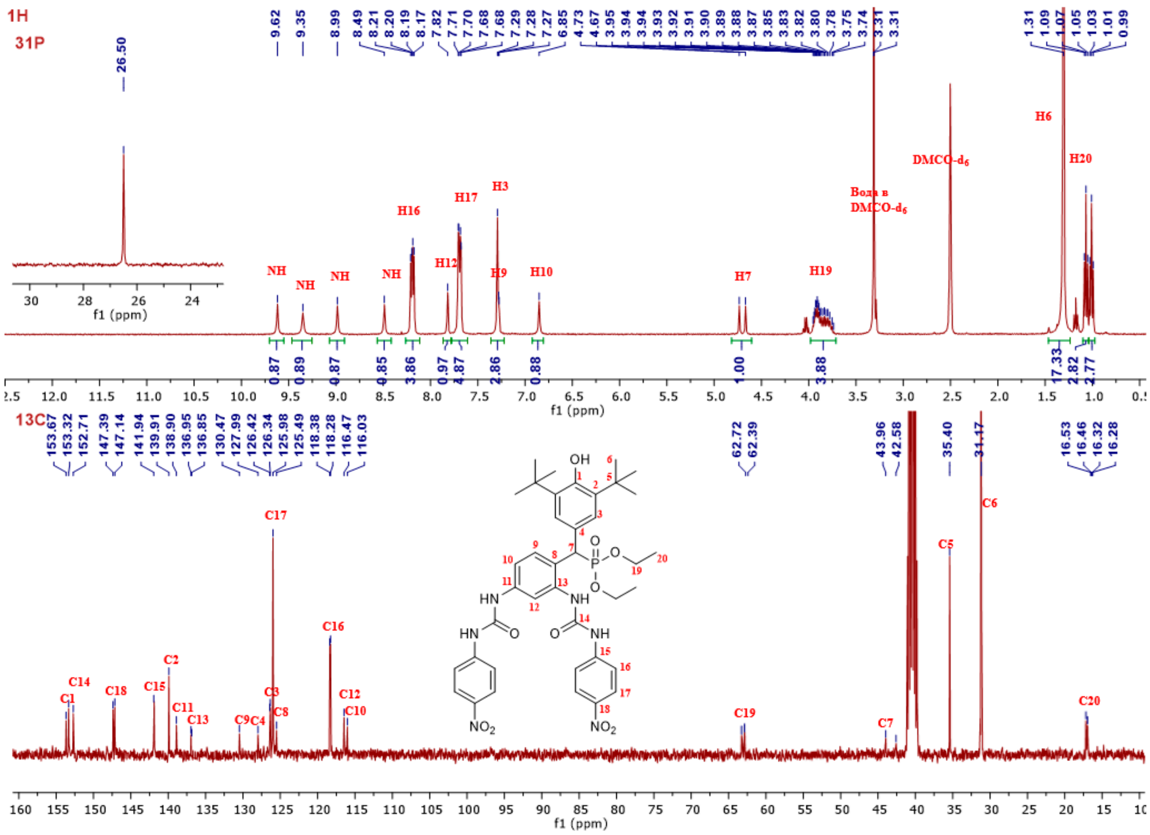

**Figure S54**. $^{1}$H-,$^{31}$P- NMR of compound **17b**.

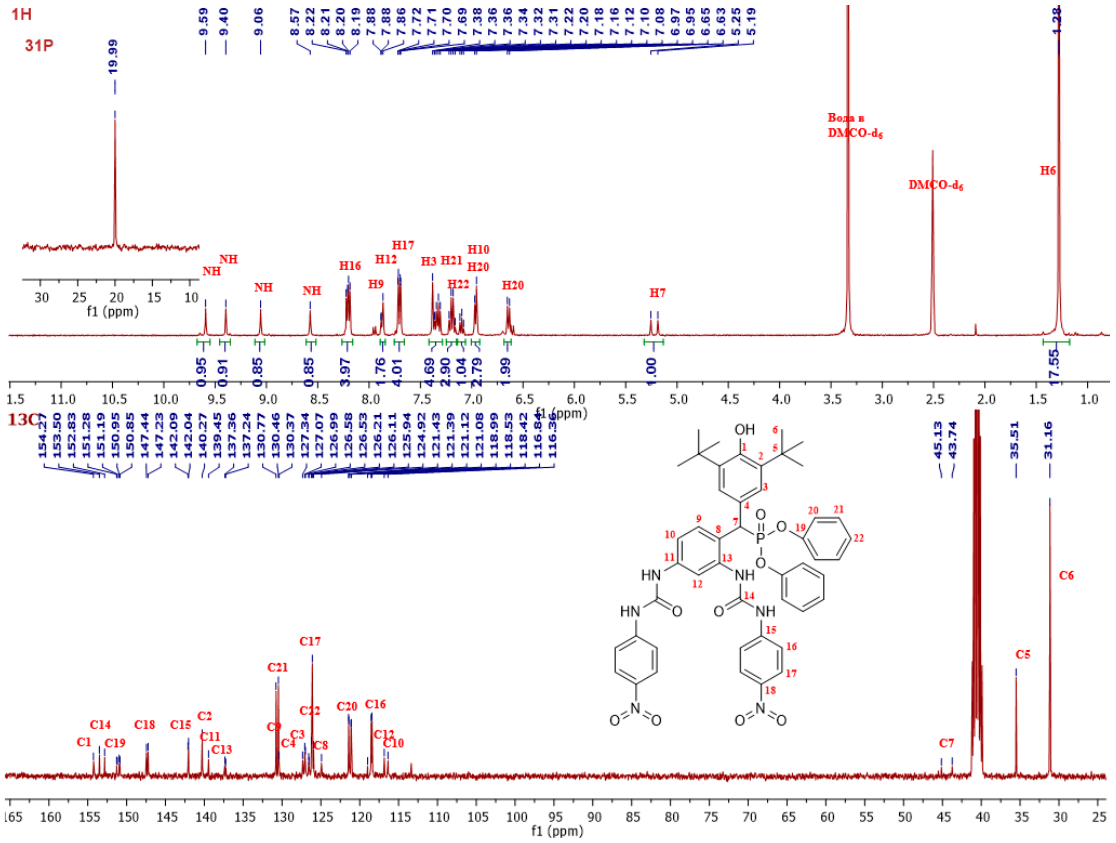

**Figure S55**. $^{1}$H-,$^{31}$P-,$^{13}$C- NMR of compound **17d**.

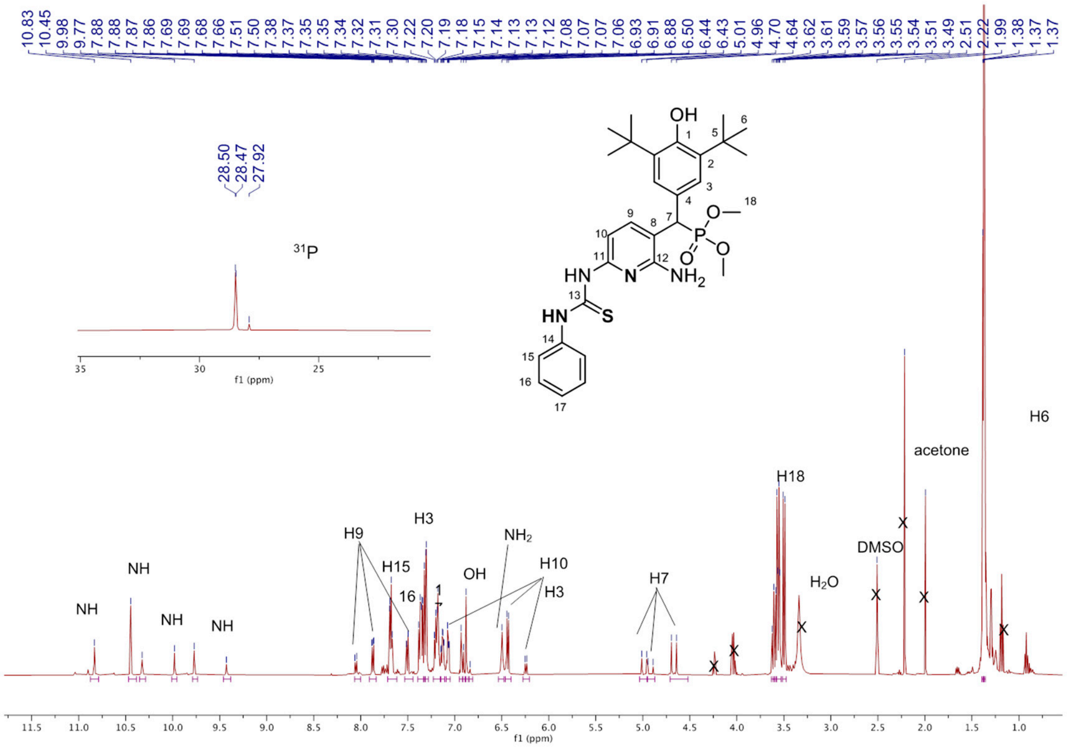


**Figure S56**. $^{1}$H-, $^{31}$P- NMR of compound **18a**.

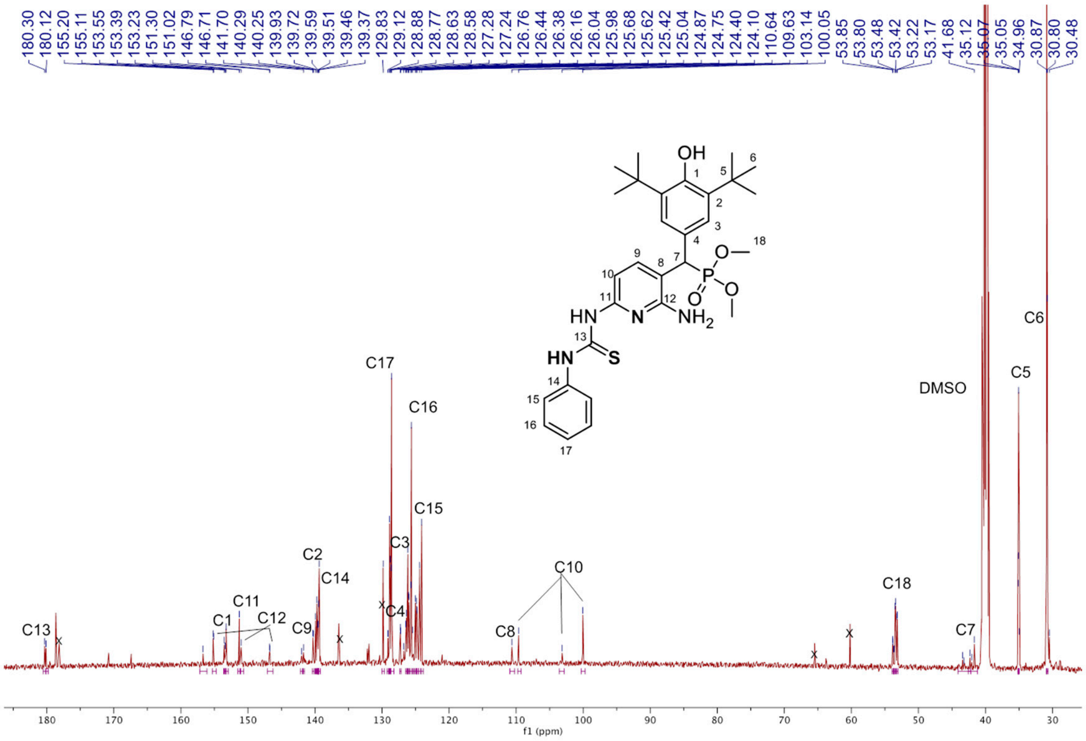


**Figure S57**. $^{13}$C- NMR of compound **18a**.

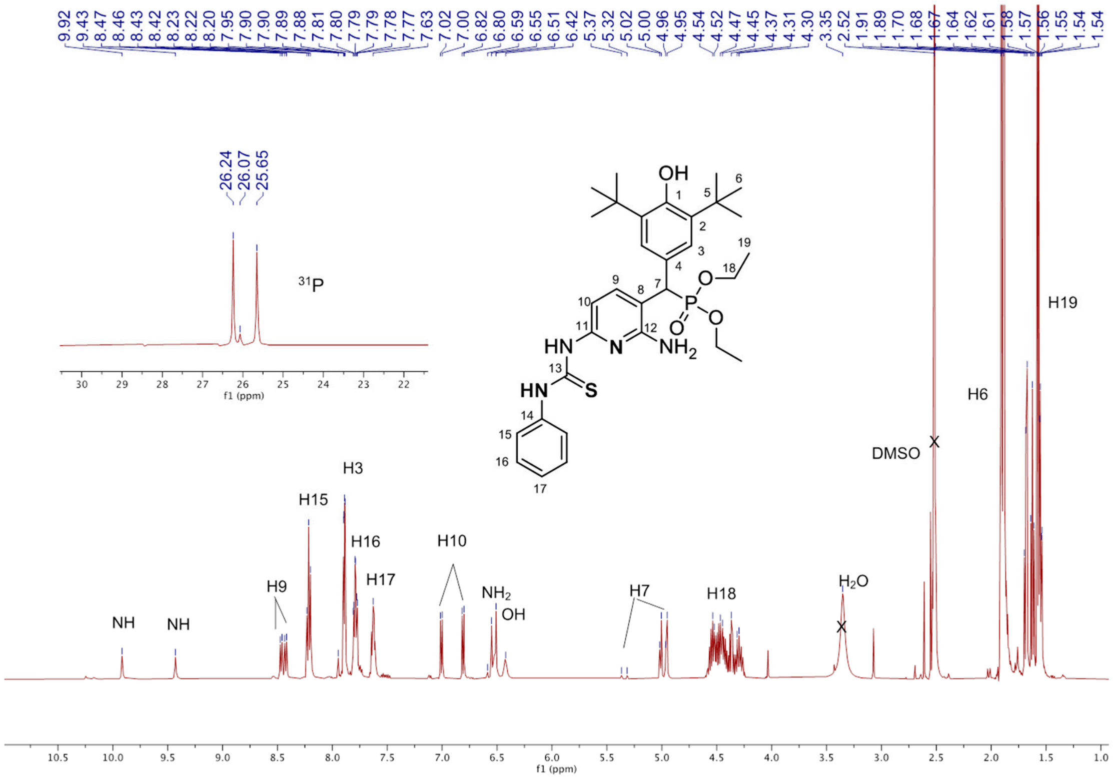


**Figure S58**. $^{1}H$-,$^{31}P$- NMR of compound **18b**.

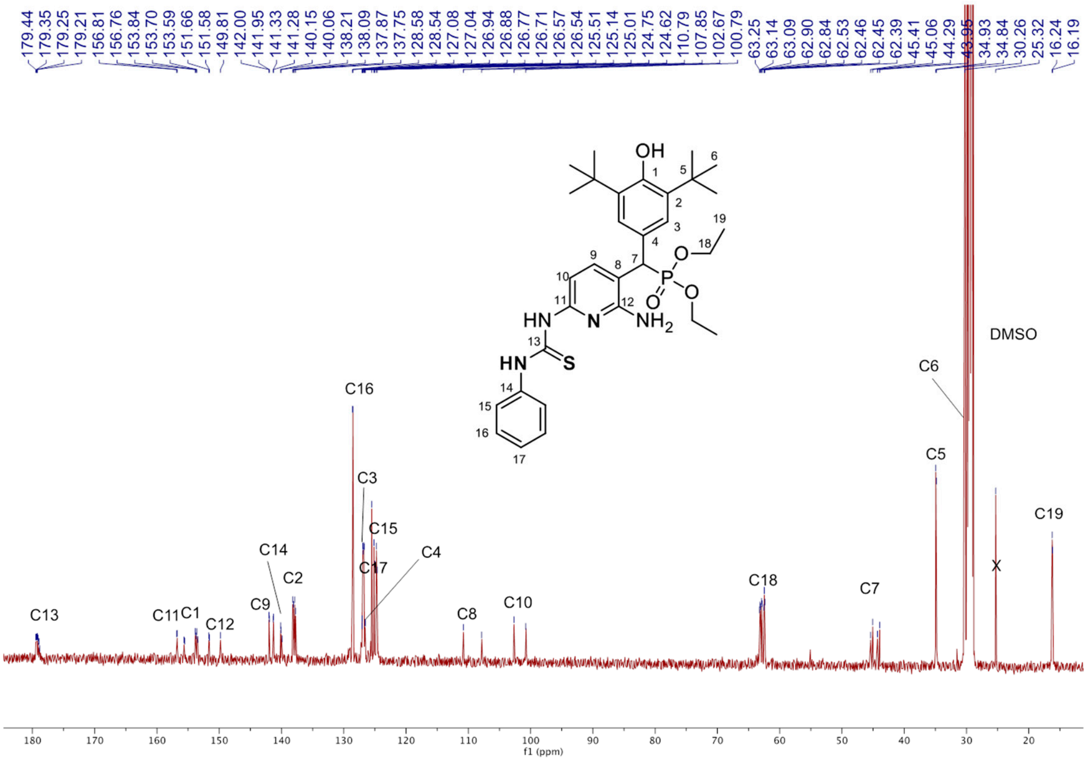


**Figure S59**. $^{13}C$- NMR of compound **18b**.

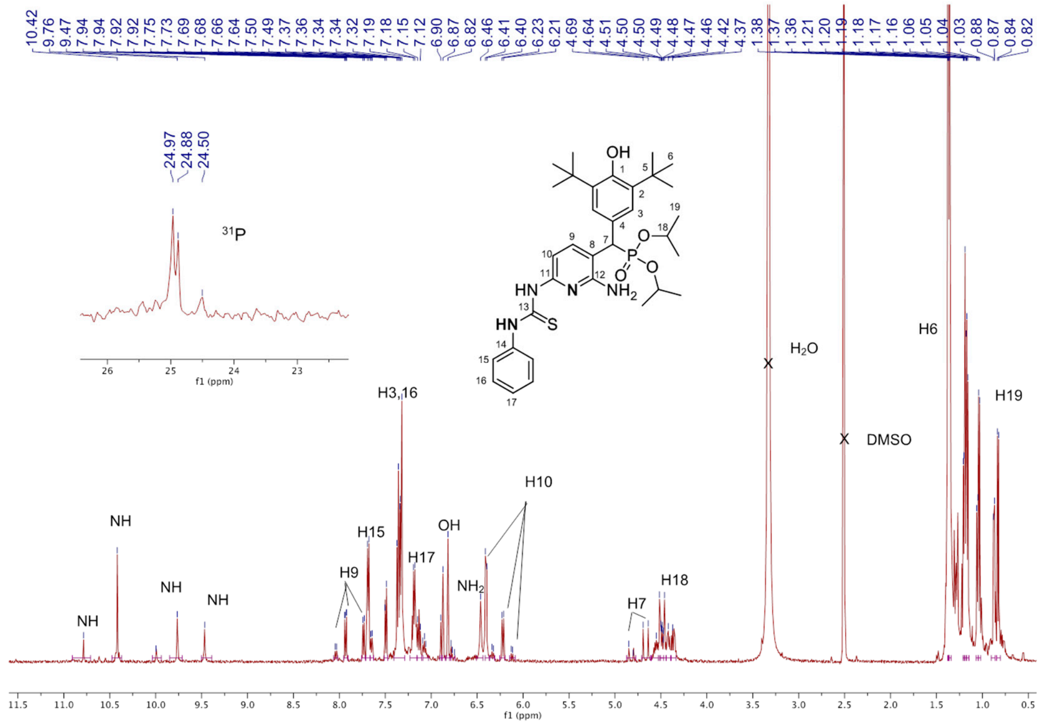


**Figure S60**. $^{1}$H-,$^{31}$P- NMR of compound **18c**.

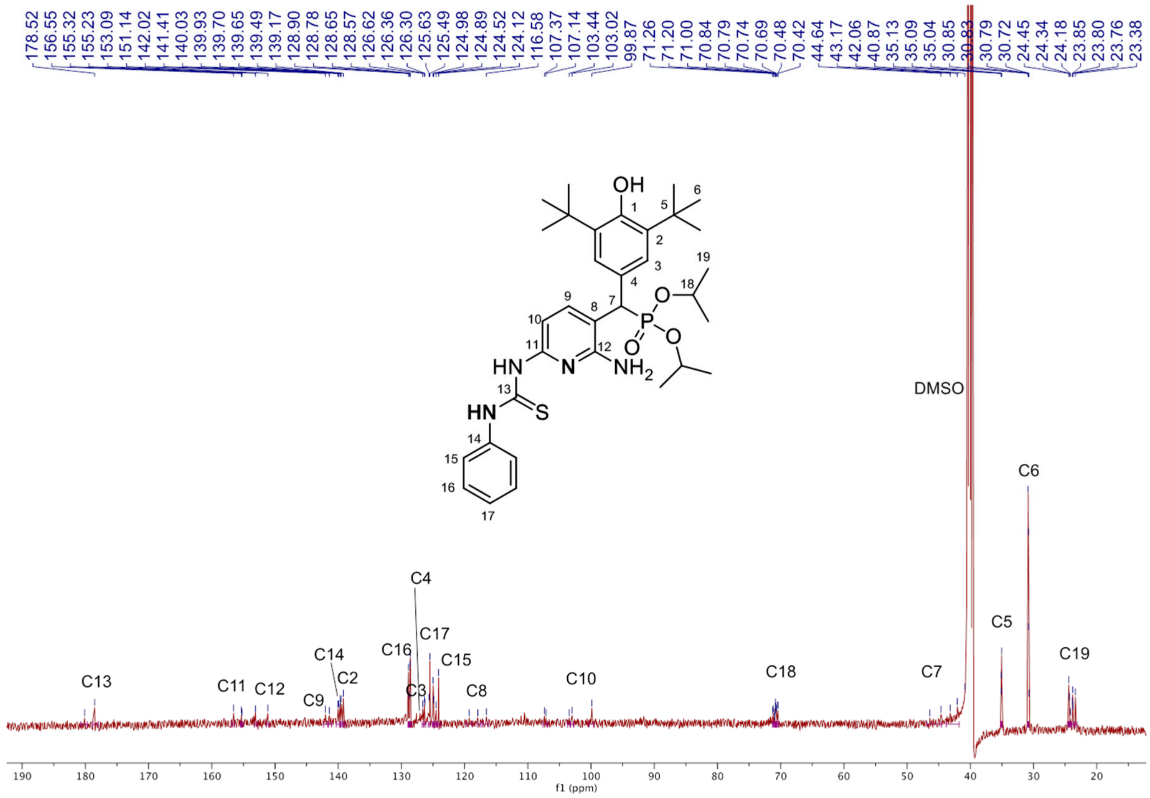


**Figure S61**. $^{13}$C- NMR of compound **18c**.

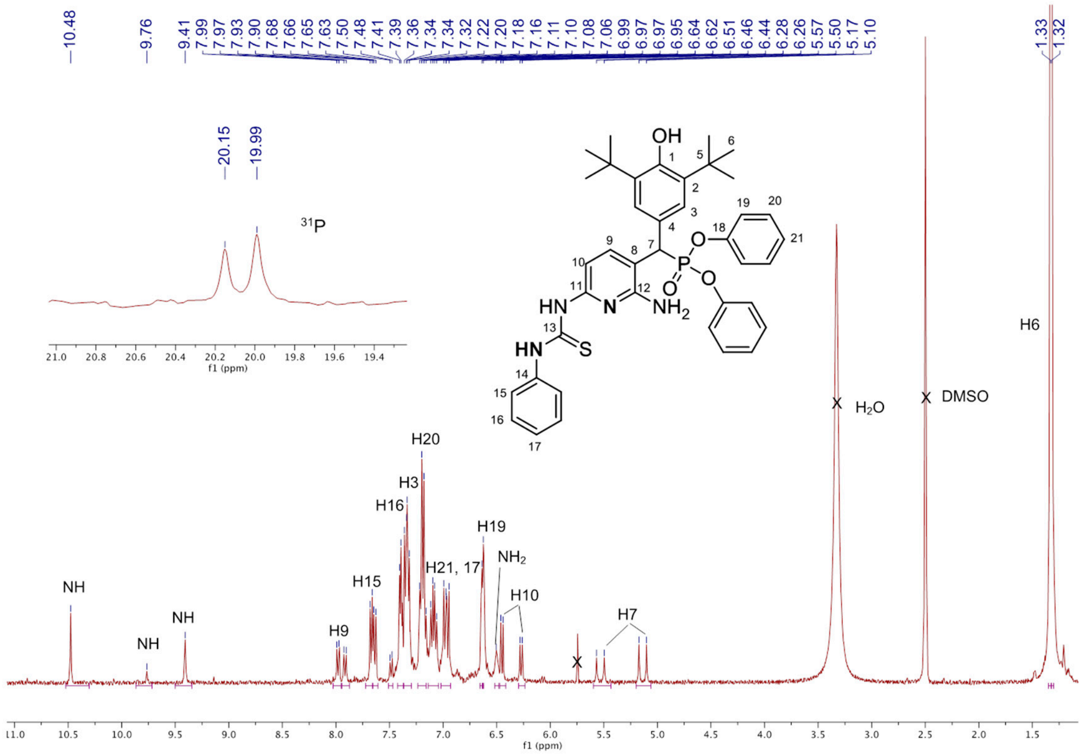


**Figure S62**. $^{1}H$-,$^{31}P$- NMR of compound **18d**.

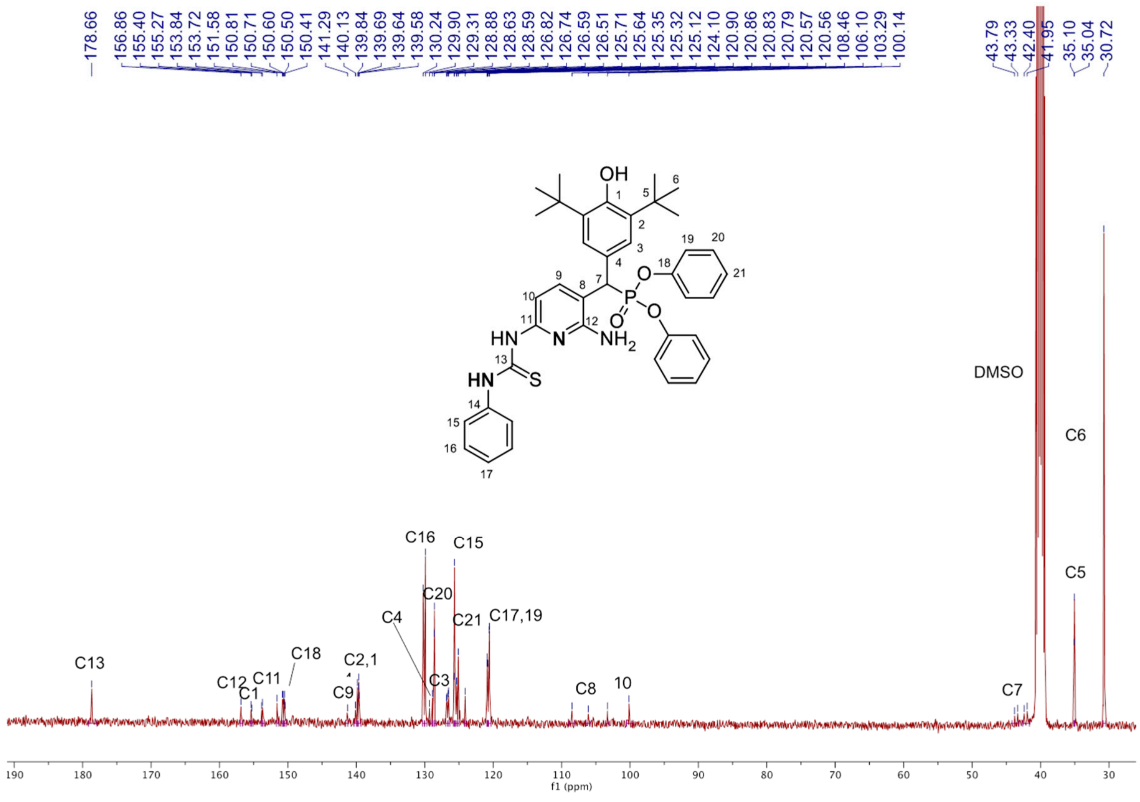

**Figure S63**. $^{13}$C- NMR of compound **18d**.

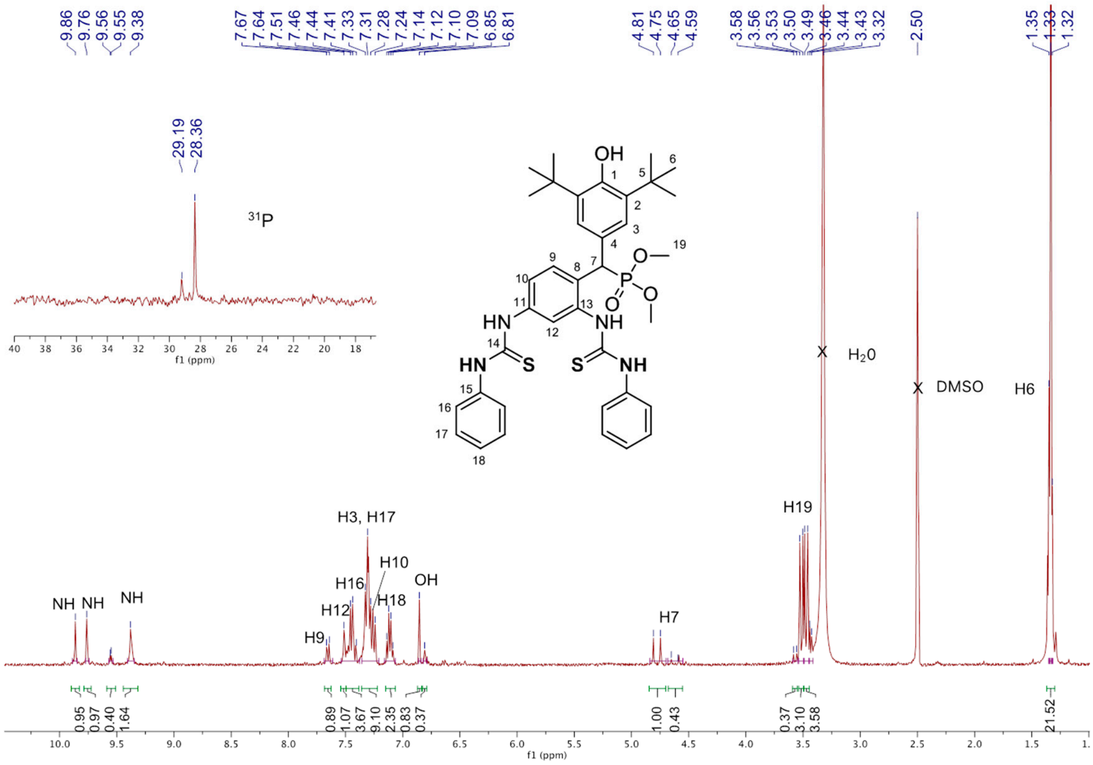


**Figure S64**. $^{1}$H-, $^{31}$P-NMR of compound **19a**.

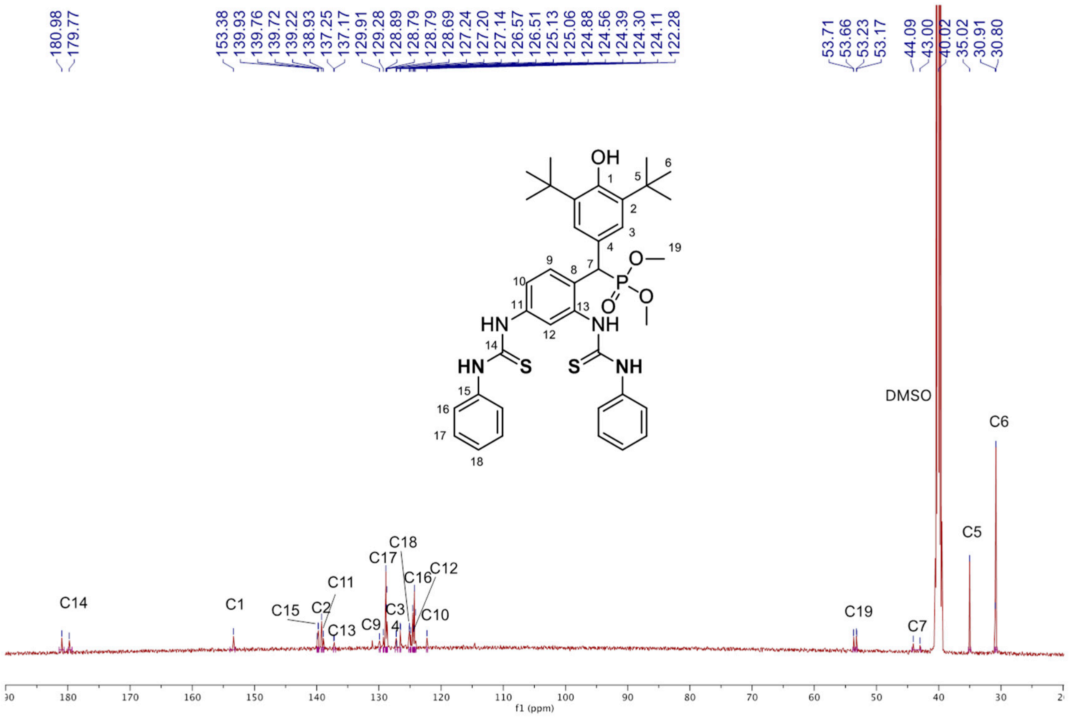

**Figure S65**. $^{13}$C- NMR of compound **19a**.

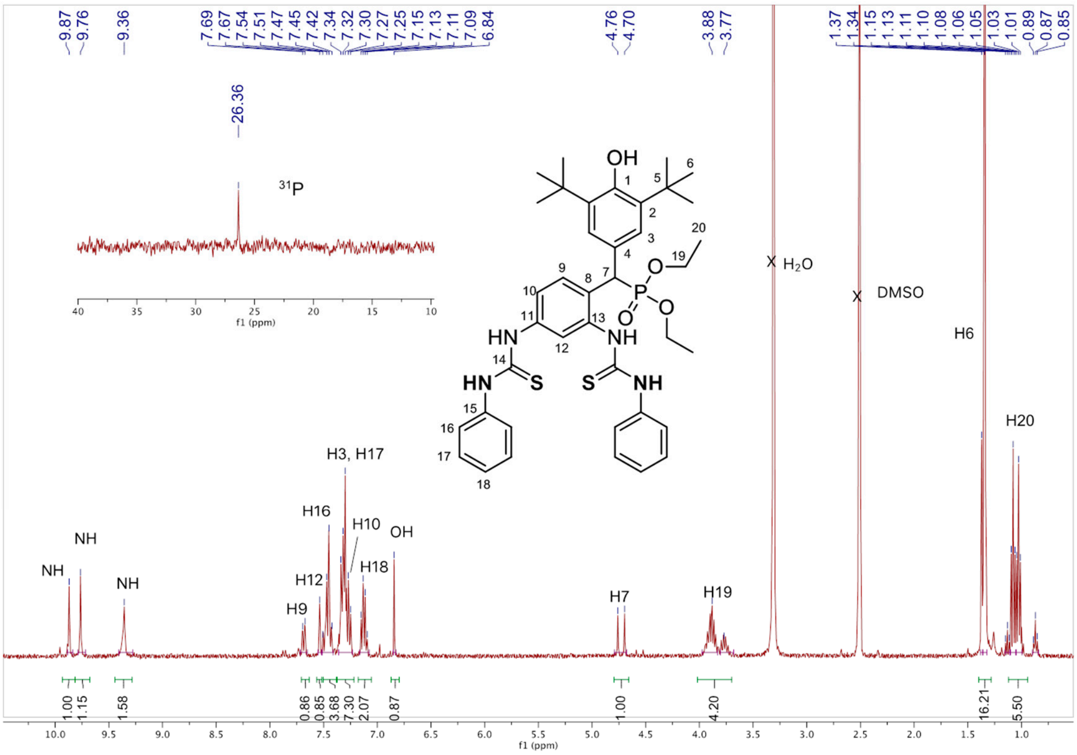


**Figure S66**. $^{1}$H-$^{31}$P- NMR of compound **19b**.

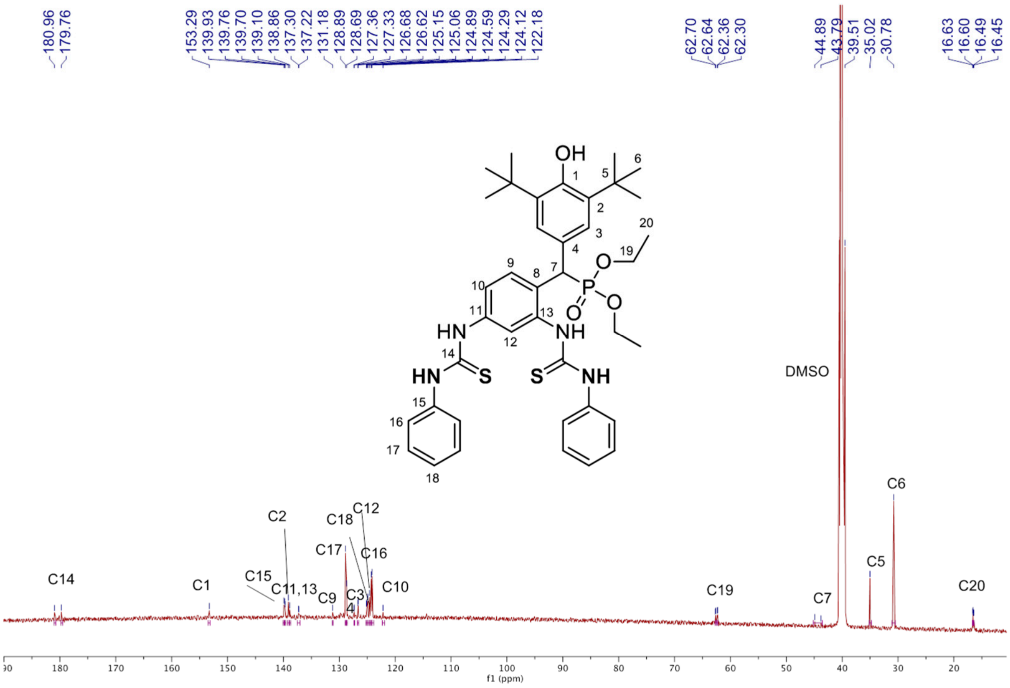


**Figure S67**. $^{13}$C- NMR of compound **19b**.

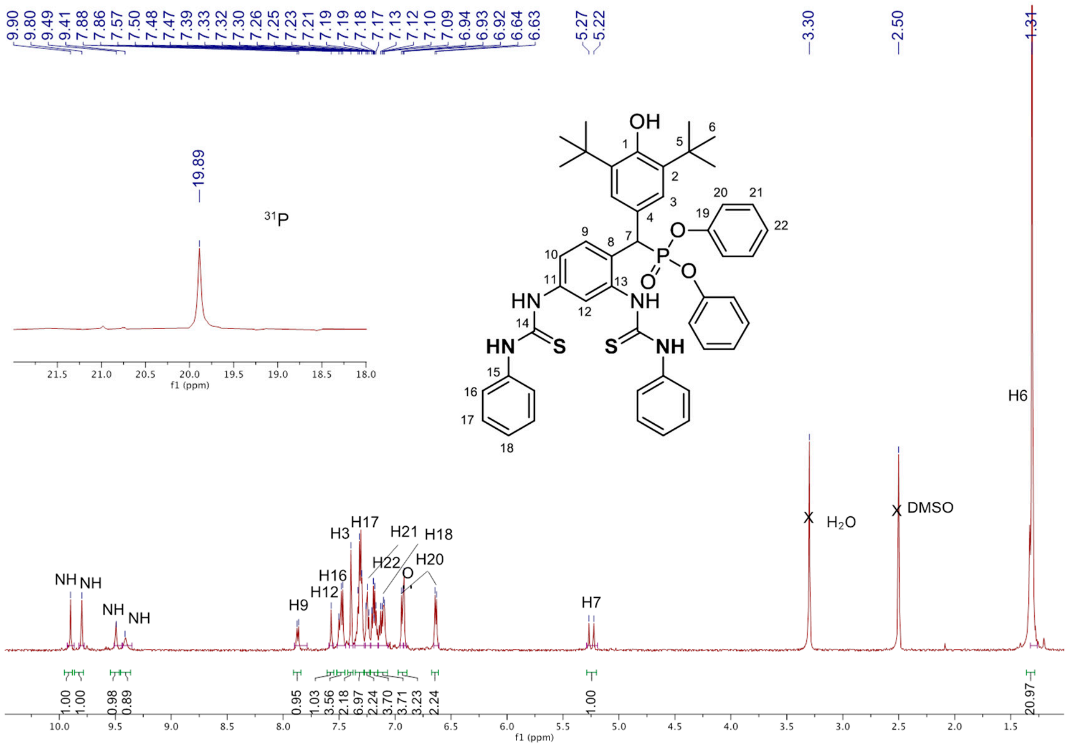

**Figure S68**. $^{1}H$-,$^{31}P$- NMR of compound **19d**.

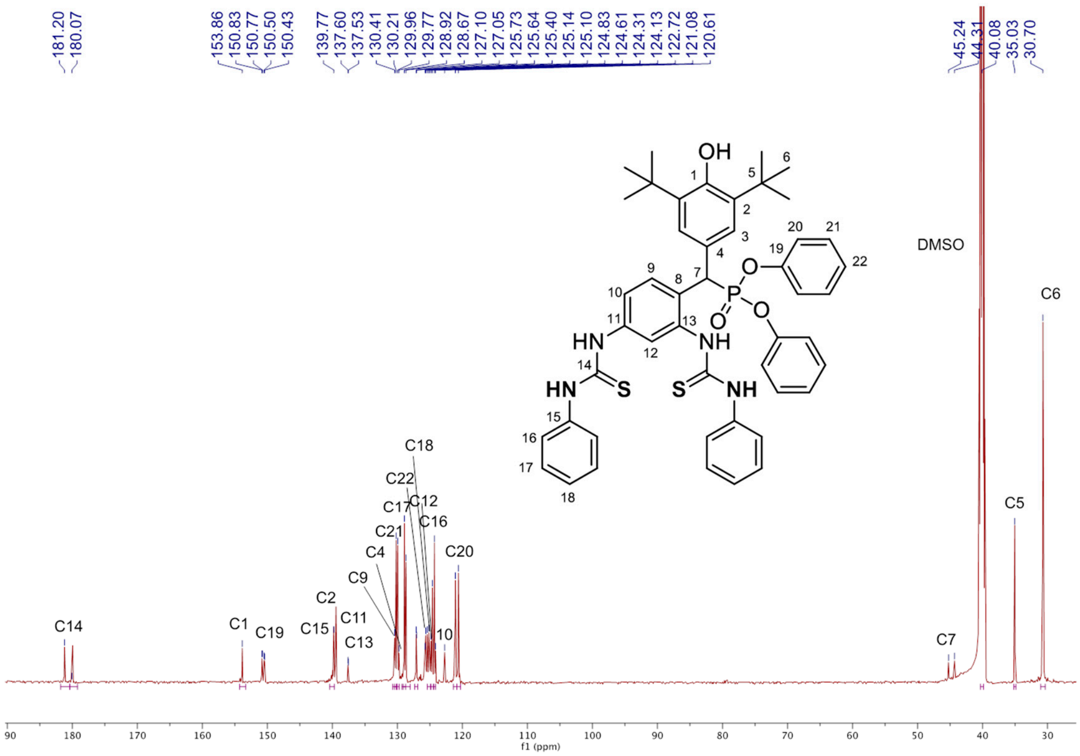

**Figure S69**. $^{13}C$- NMR of compound **19d**.

## 2D correlation NMR experiments

## $^{1}H$-$^{1}H$ COSY, $^{1}H$-$^{13}C$ HSQC, $^{1}H$-$^{13}C$ HMBC) of compounds 5a-d; 6a,b,d; 18a-d and 19a,b,d.

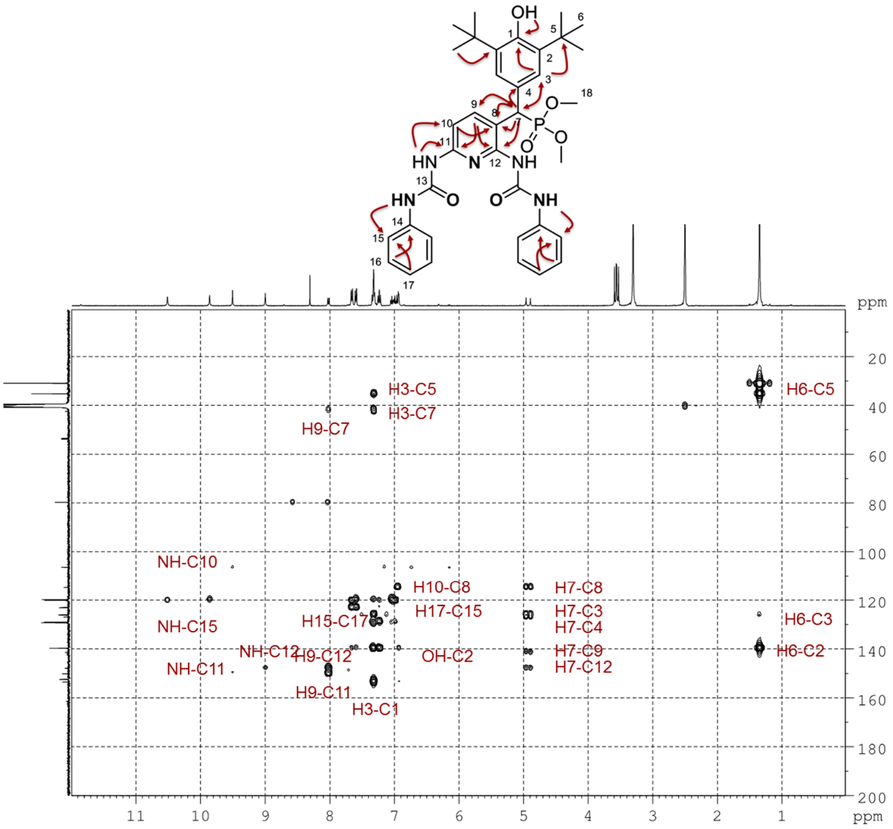


**Figure S70**. $^{1}H$- $^{13}C$ HMBC correlations of compound **5a**.

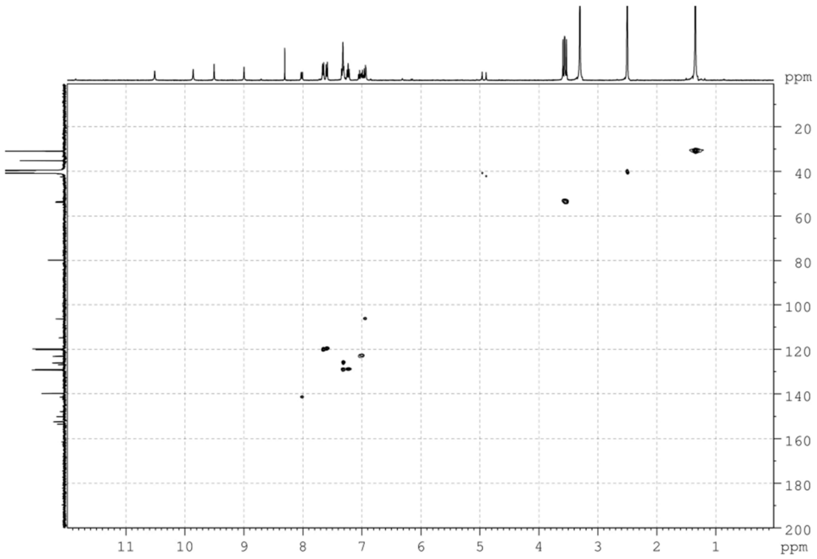


**Figure S71**. $^{1}$H- $^{13}$C HSQC correlations of compound **5a**.

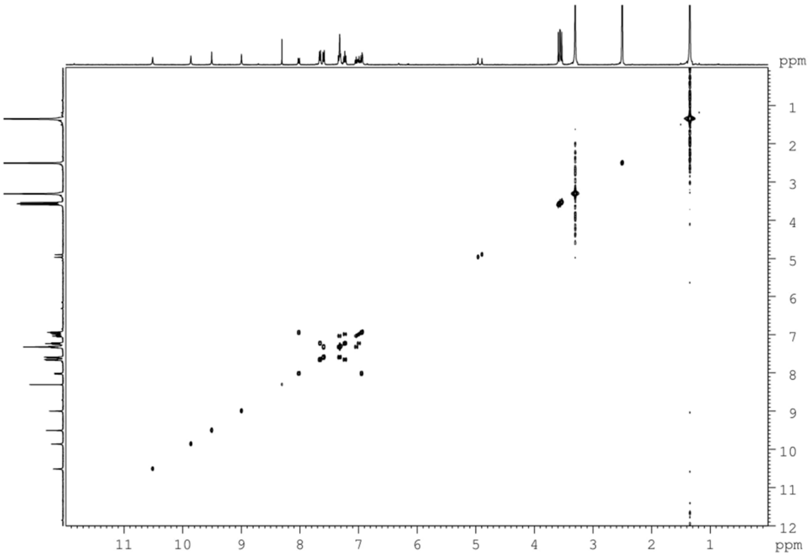


**Figure S72**. $^{1}$H- $^{1}$H COSY correlations of compound **5a**.

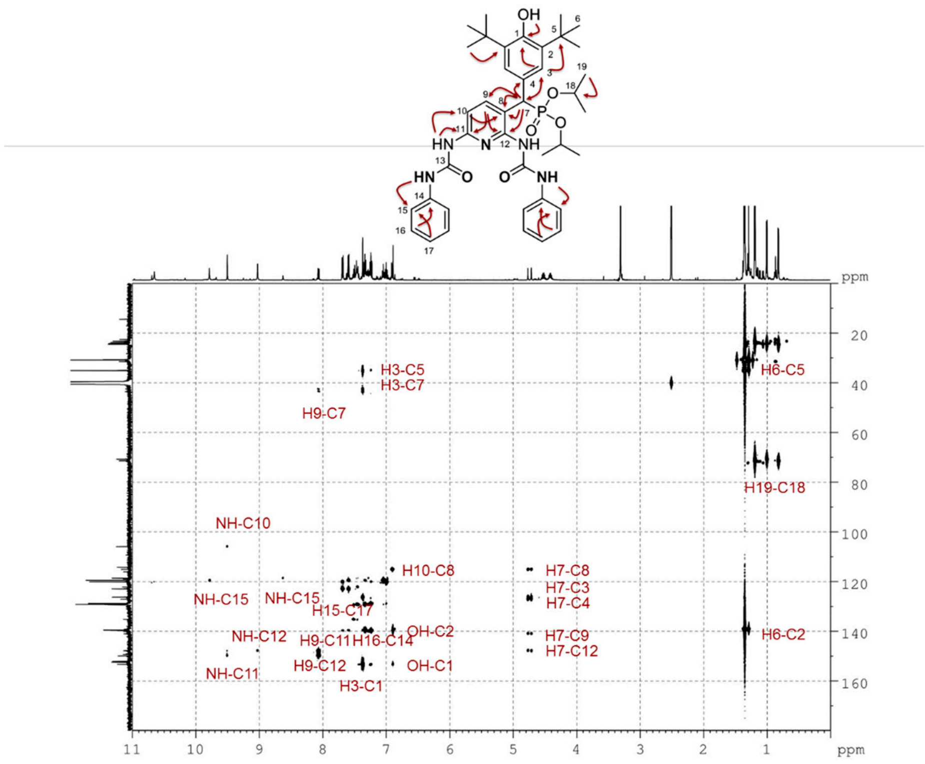


**Figure S73**. $^{1}H$- $^{13}C$ HMBC correlations of compound **5c**.

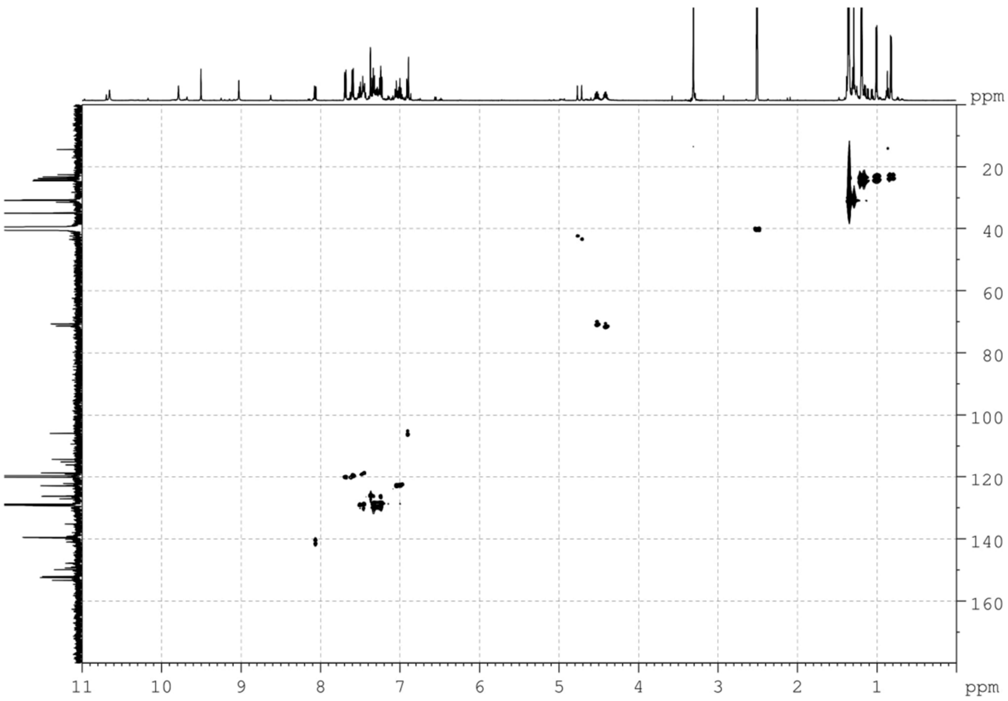

**Figure S74**. $^{1}$H- $^{13}$C HSQC correlations of compound **5c**.

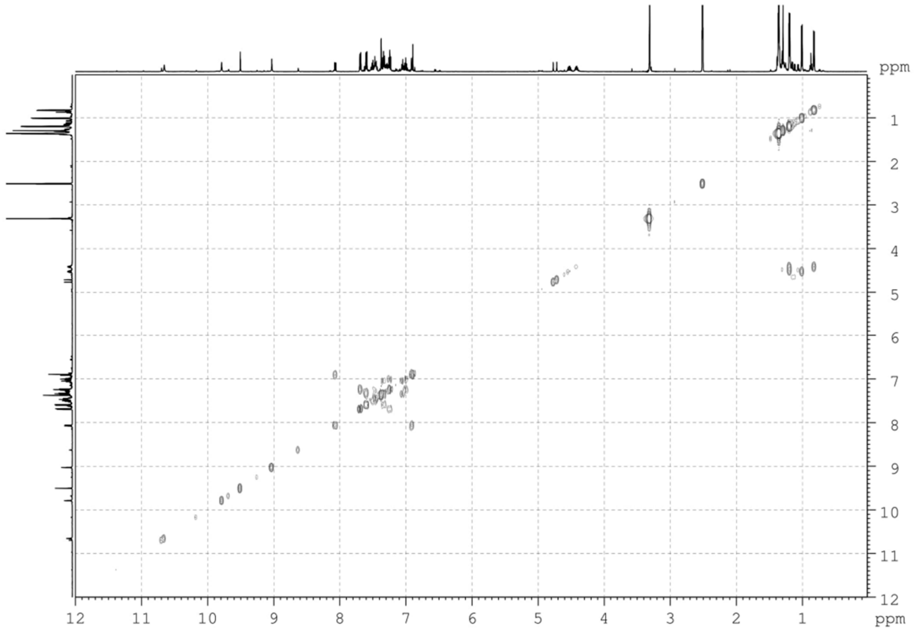

**Figure S75**. $^{1}$H- $^{1}$H COSY correlations of compound **5c**.

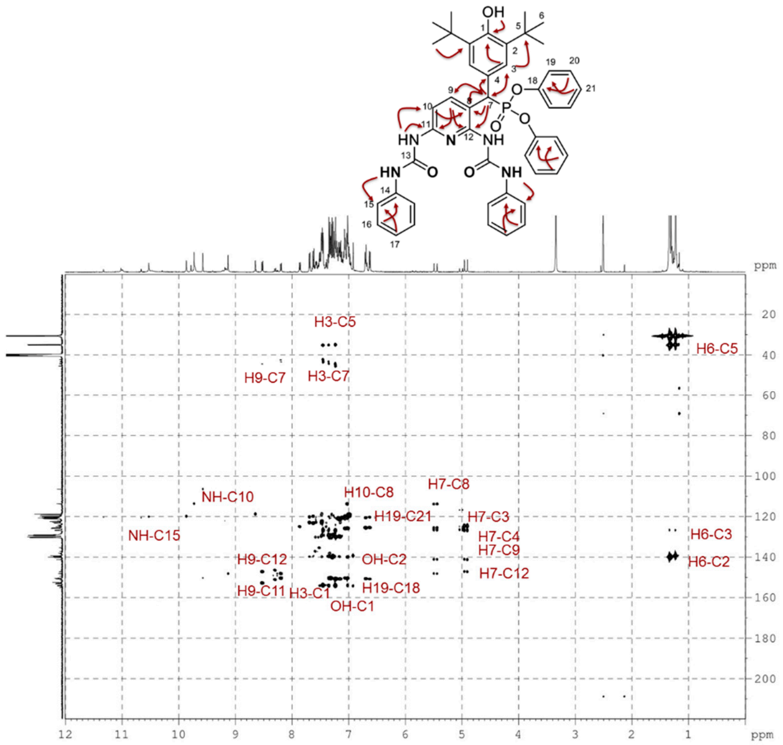


**Figure S76**. $^{1}$H- $^{13}$C HMBC correlations of compound **5d**.

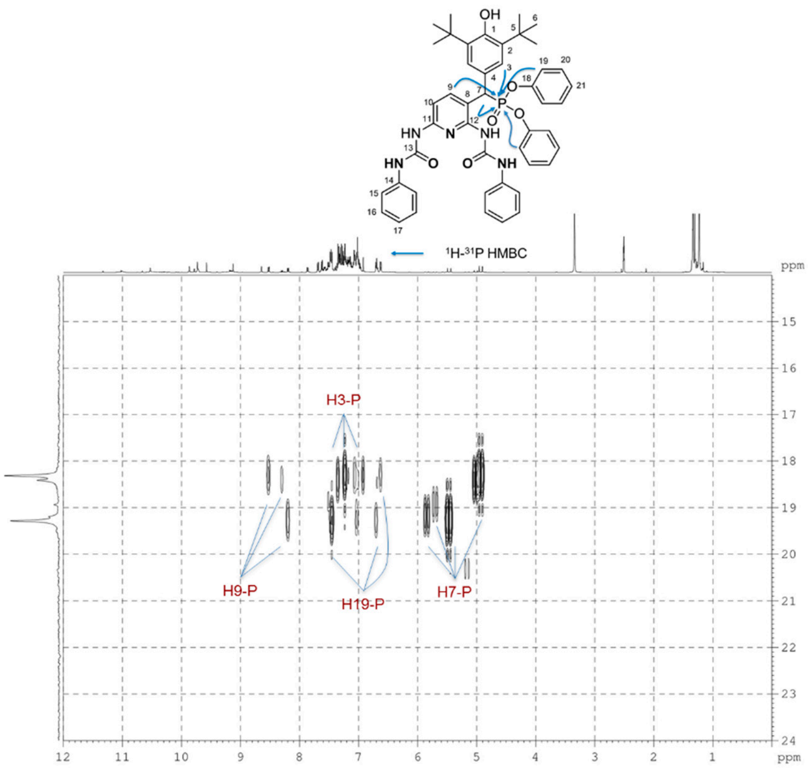


**Figure S77**. $^{1}$H- $^{31}$P HMBC correlations of compound **5d**.

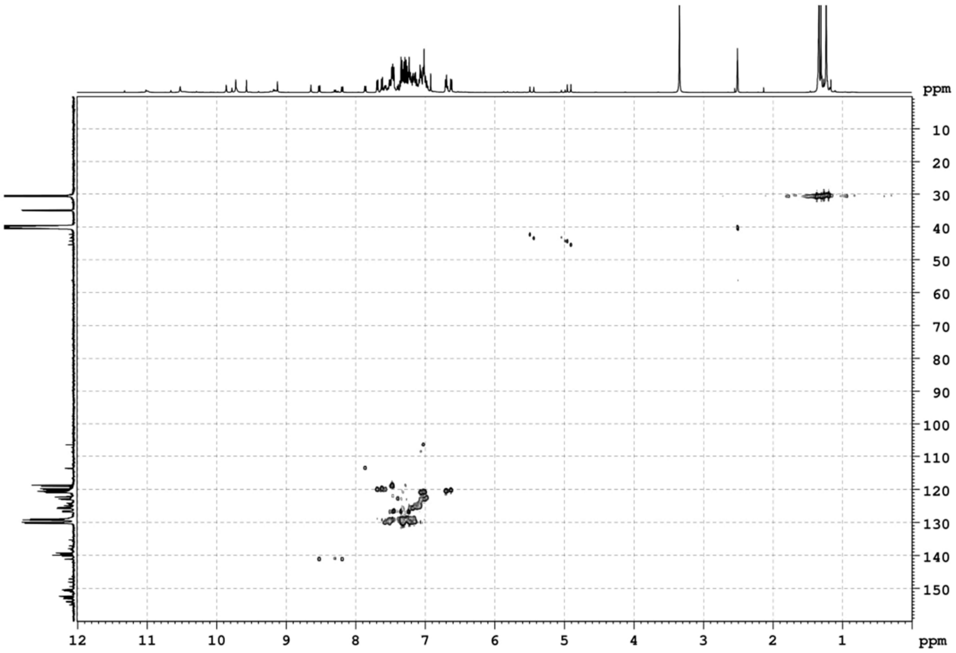


**Figure S78**. $^{1}$H- $^{13}$C HSQC correlations of compound **5d**.

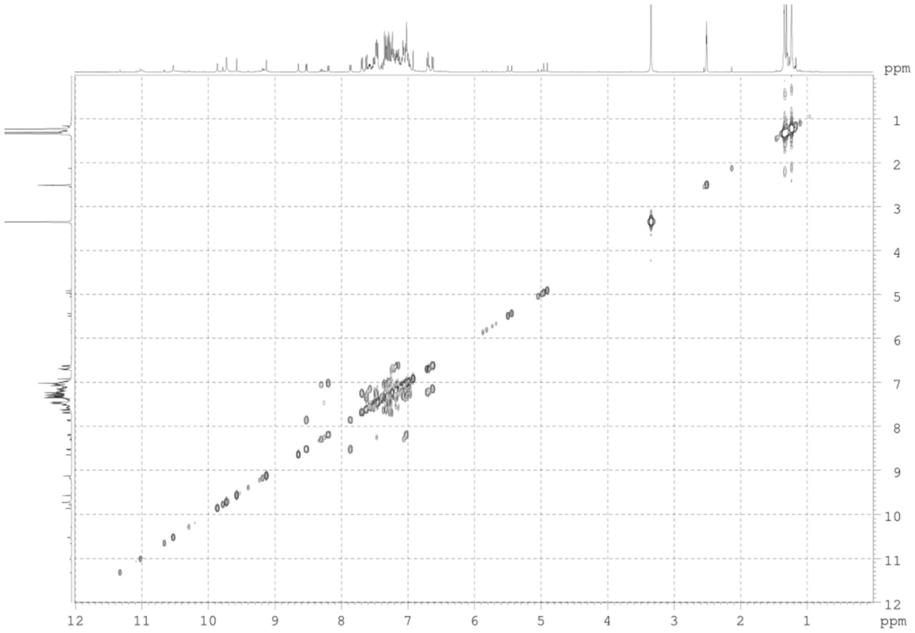

**Figure S79**. $^{1}$H- $^{1}$H COSY correlations of compound **5d**.

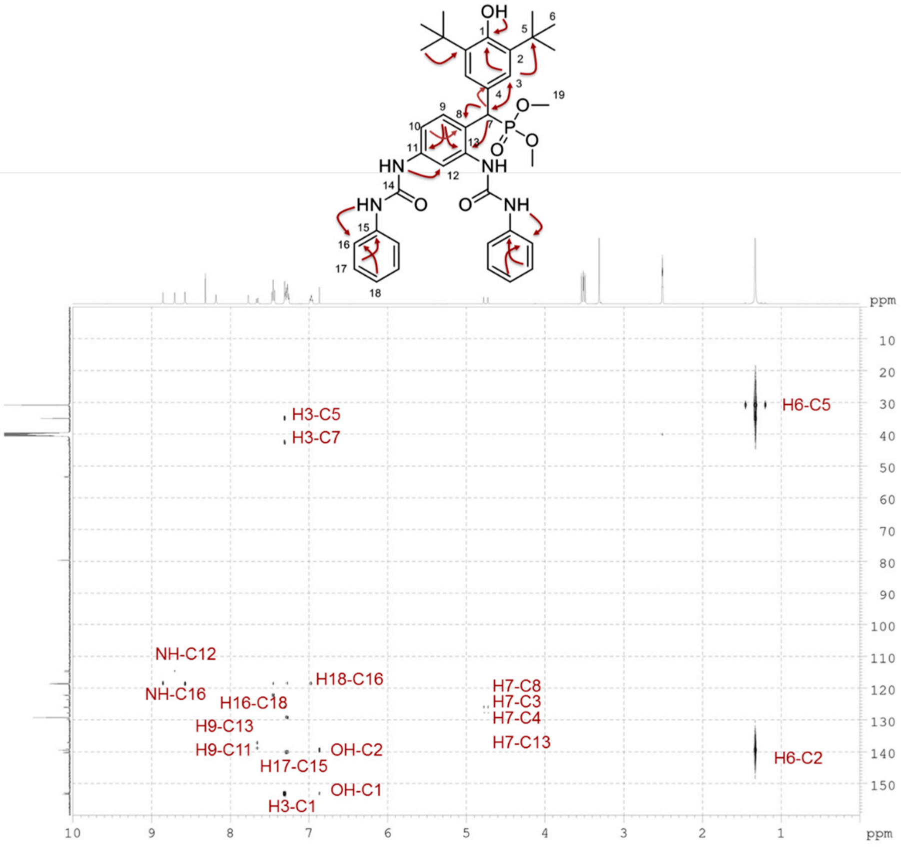

**Figure S80**. $^{1}$H- $^{13}$C HMBC correlations of compound **6a**.

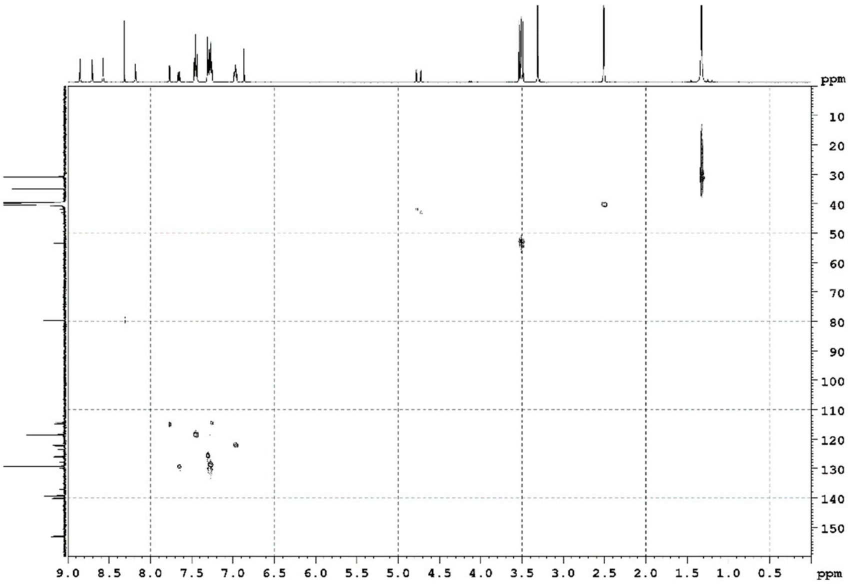

**Figure S81**. $^{1}$H-$^{13}$C HSQC correlations of compound **6a**.

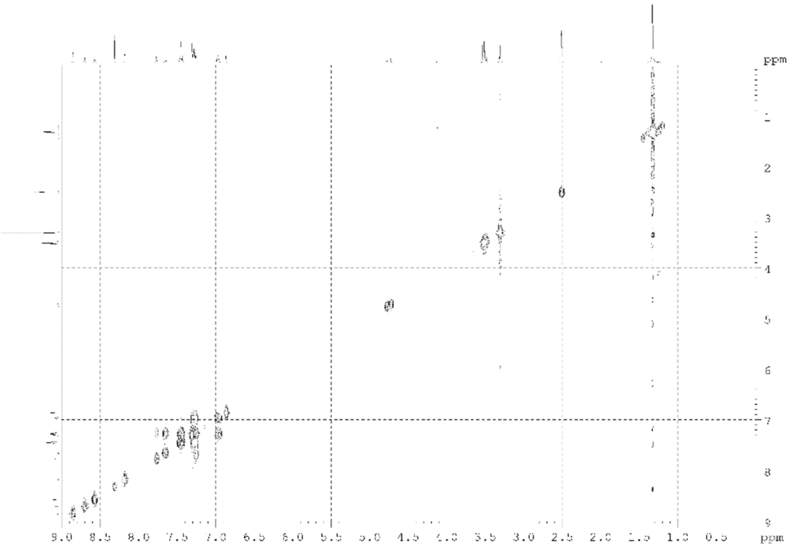

**Figure S82**. $^{1}$H- $^{1}$H COSY correlations of compound **6a**.

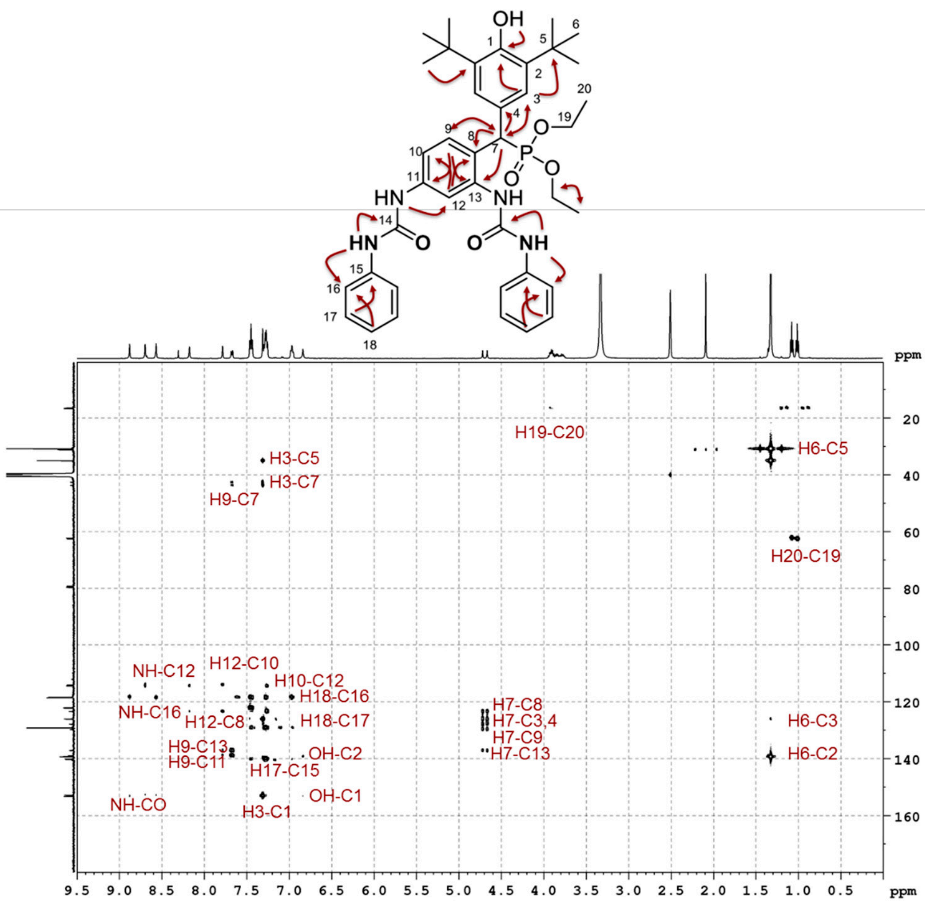


**Figure S83**. $^{1}H$- $^{13}C$ HMBC correlations of compound **6b**.

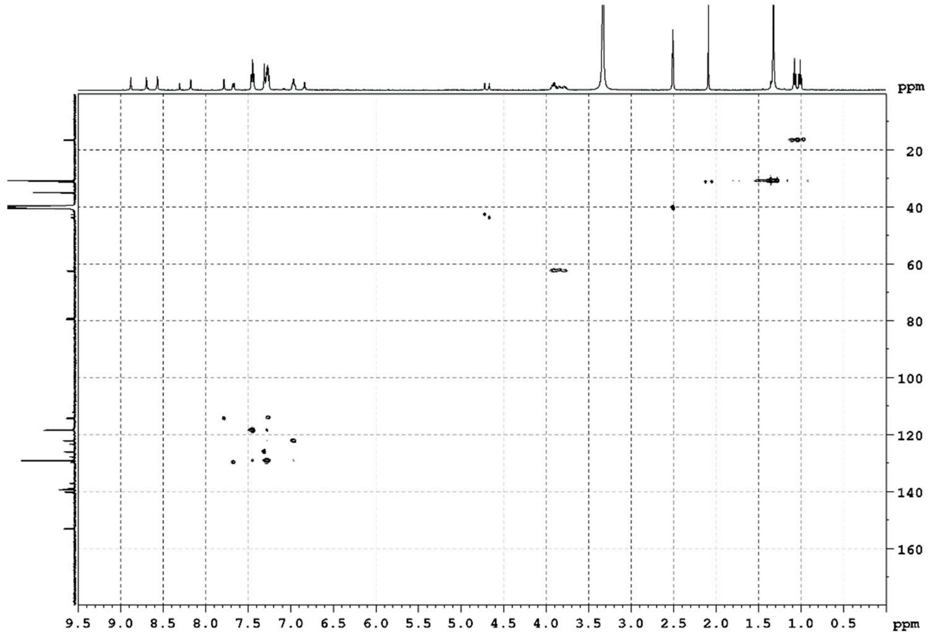


**Figure S84**. ¹H- ¹³C HSQC correlations of compound **6b**.

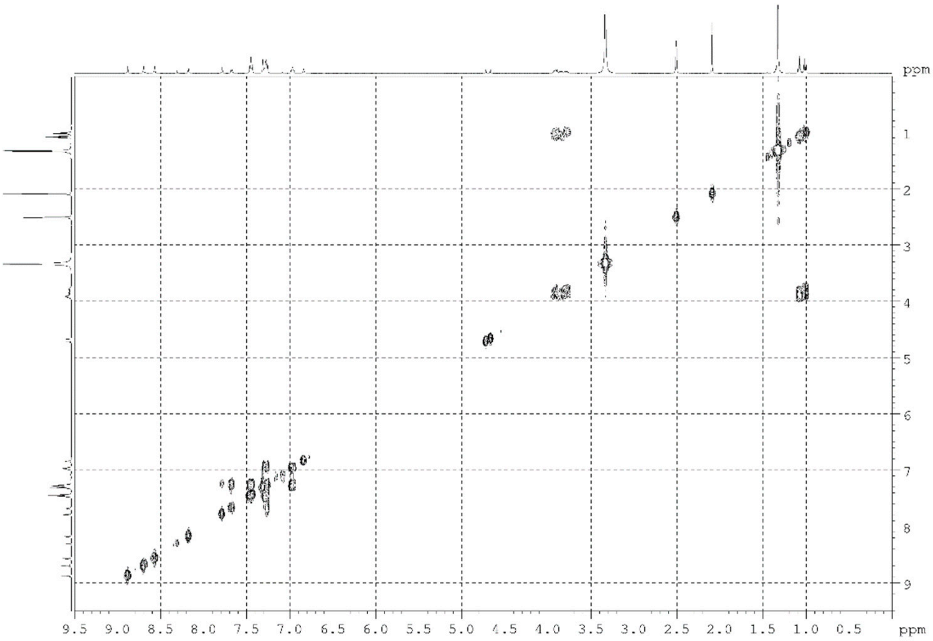


**Figure S85**. ¹H- ¹H COSY correlations of compound **6b**.

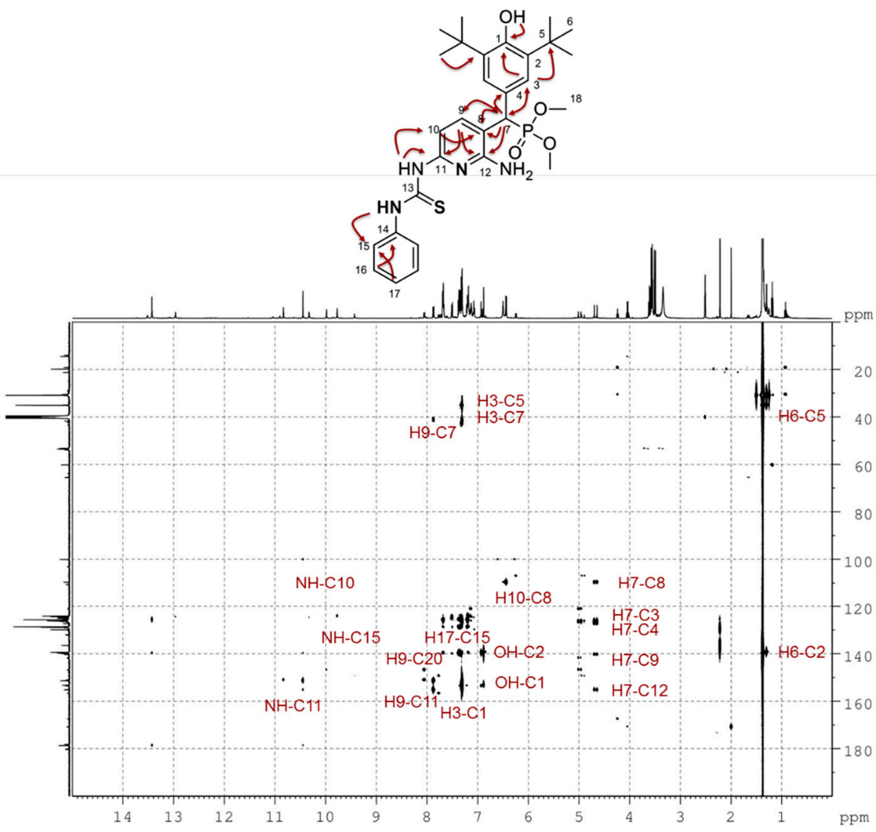


**Figure S86**. ¹H- ¹³C HMBC correlations of compound **18a**.

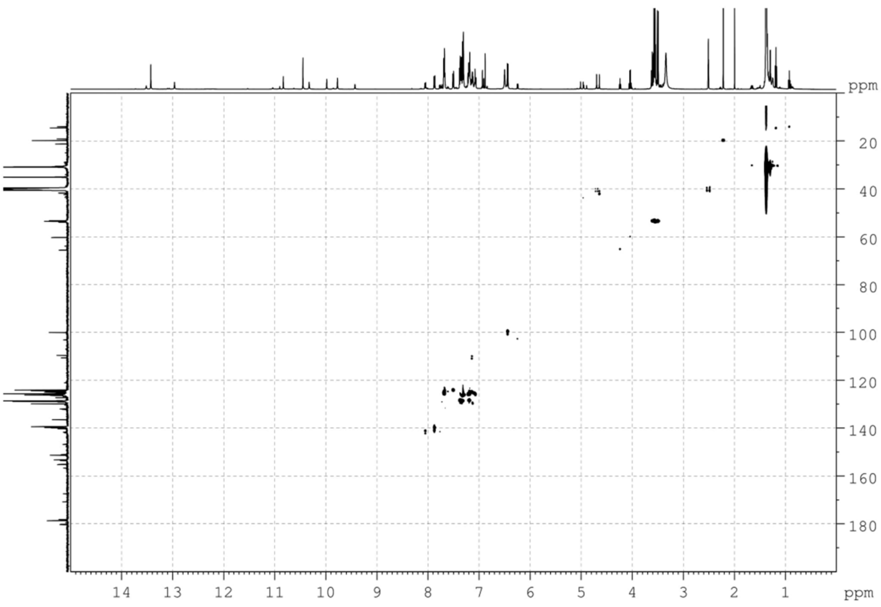


**Figure S87**. ¹H- ¹³C HSQC correlations of compound **18a**.

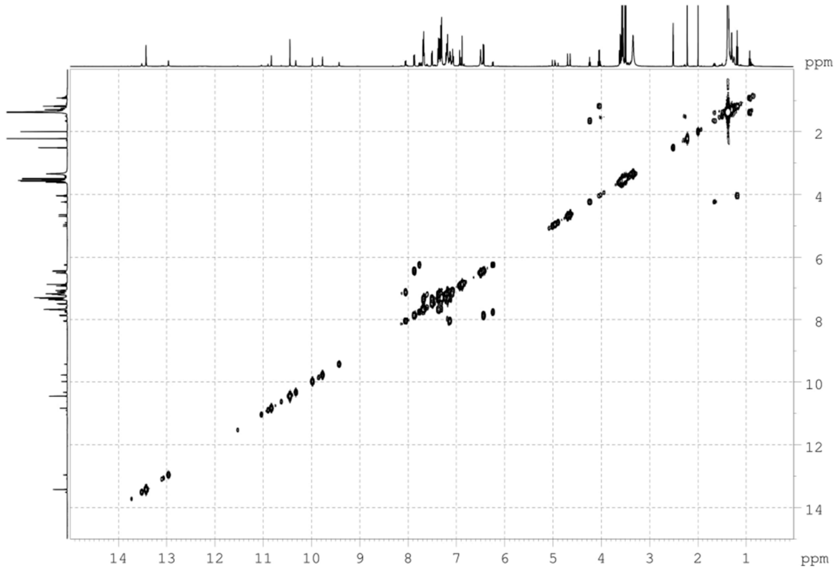


**Figure S88**. $^{1}H$- $^{1}H$ COSY correlations of compound **18a**.

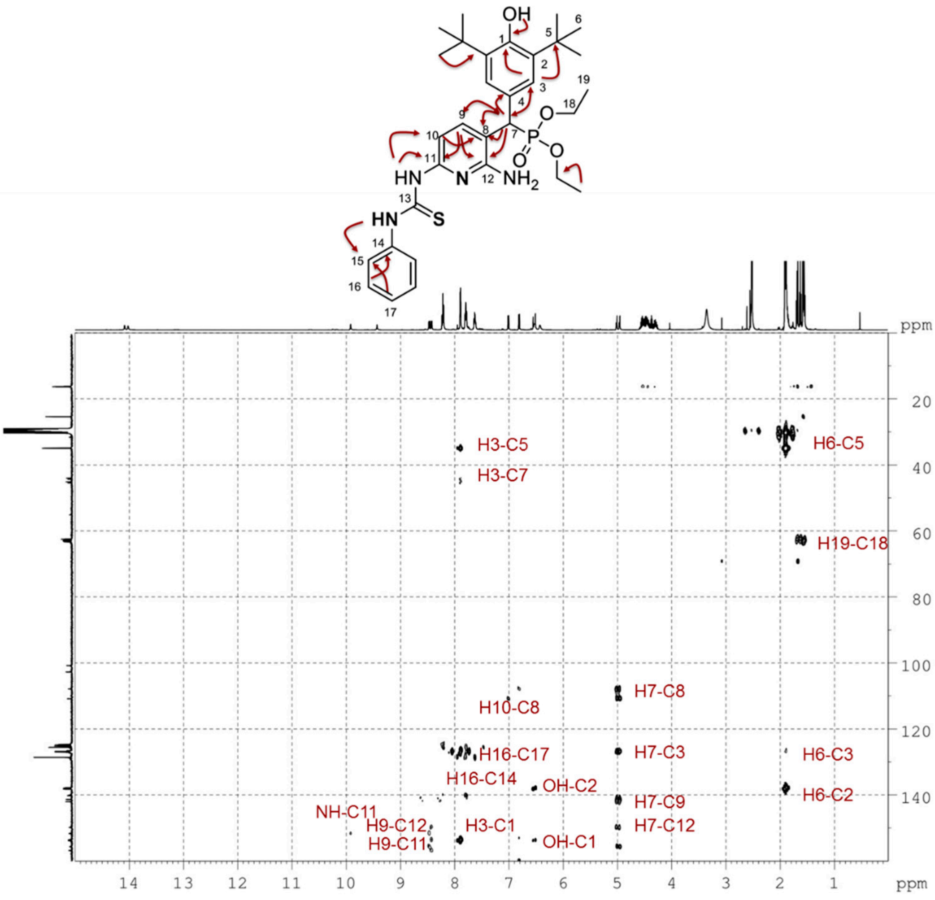

**Figure S89**. $^{1}H$- $^{13}C$ HMBC correlations of compound **18b**.

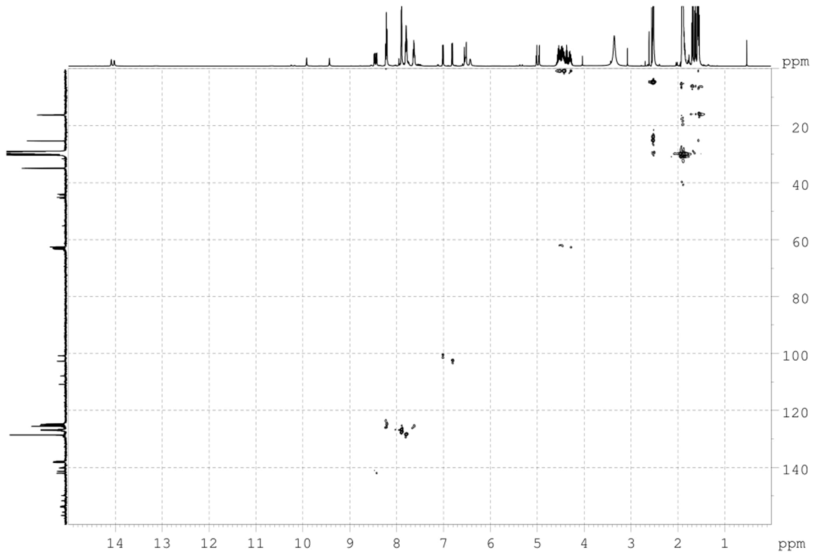

**FigureS90**. $^{1}H$- $^{13}C$ HSQC correlations of compound **18b**.

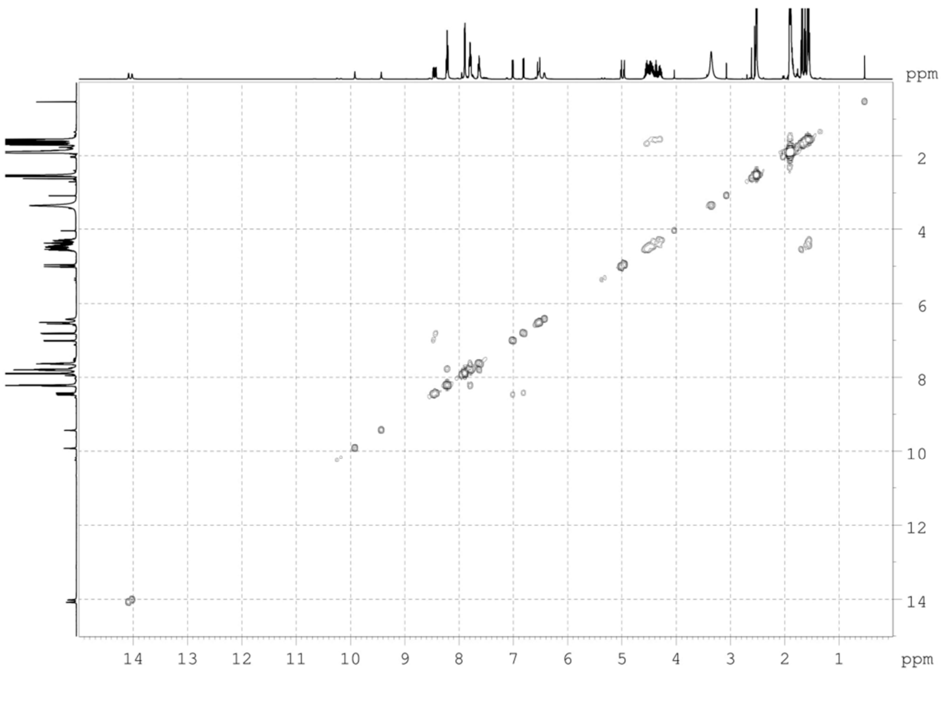

**Figure S91**. $^{1}H$- $^{1}H$ COSY correlations of compound **18b**.

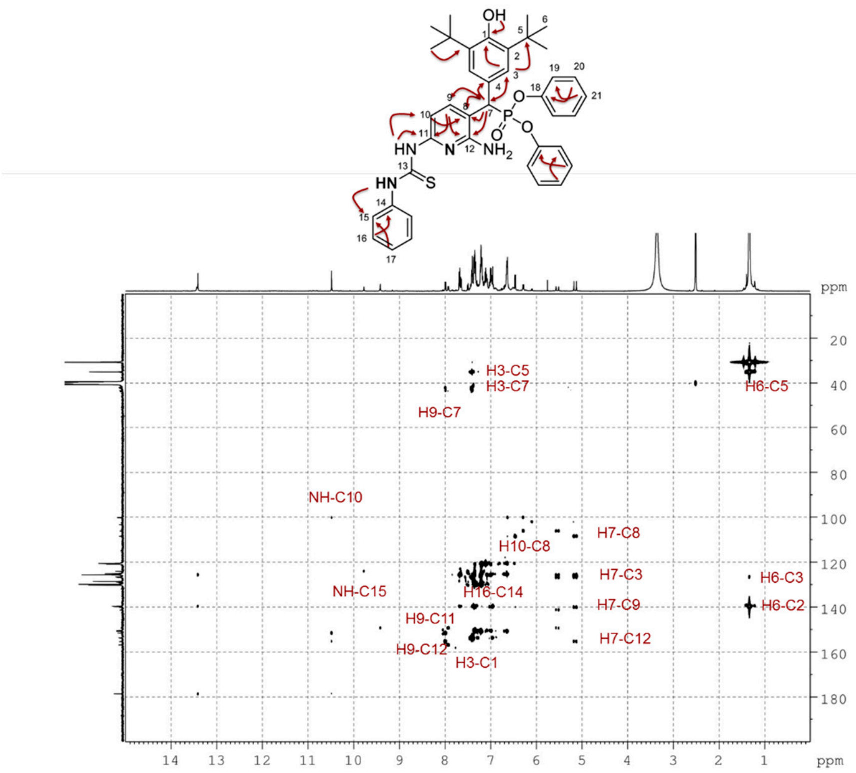


**Figure S92**. $^{1}H$- $^{13}C$ HMBC correlations of compound **18d**.

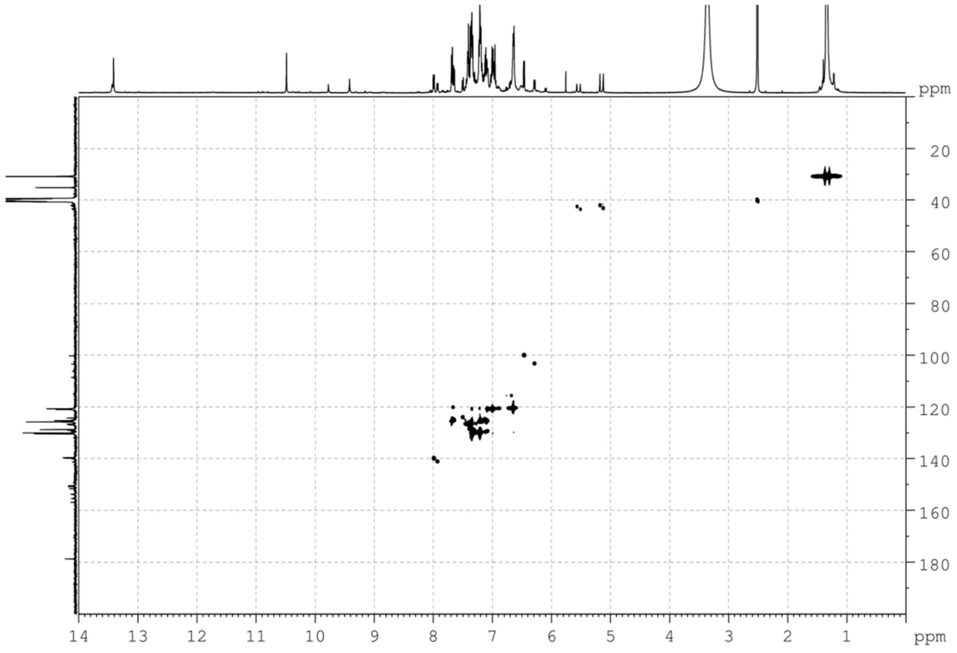


**Figure S93**. $^{1}H$- $^{13}C$ HSQC correlations of compound **18d**.

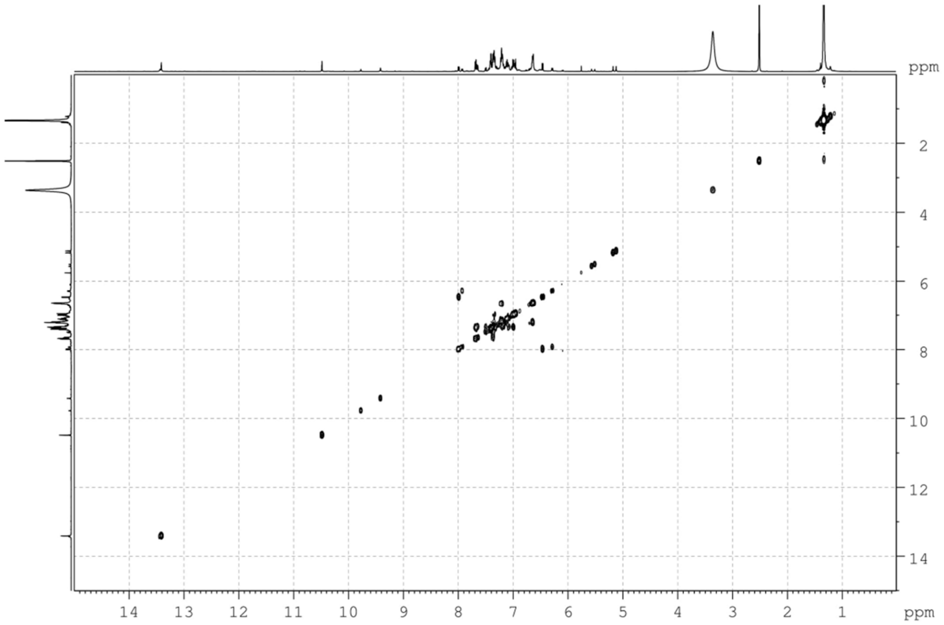

**Figure S94**. $^{1}$H- $^{1}$H COSY correlations of compound **18d**.

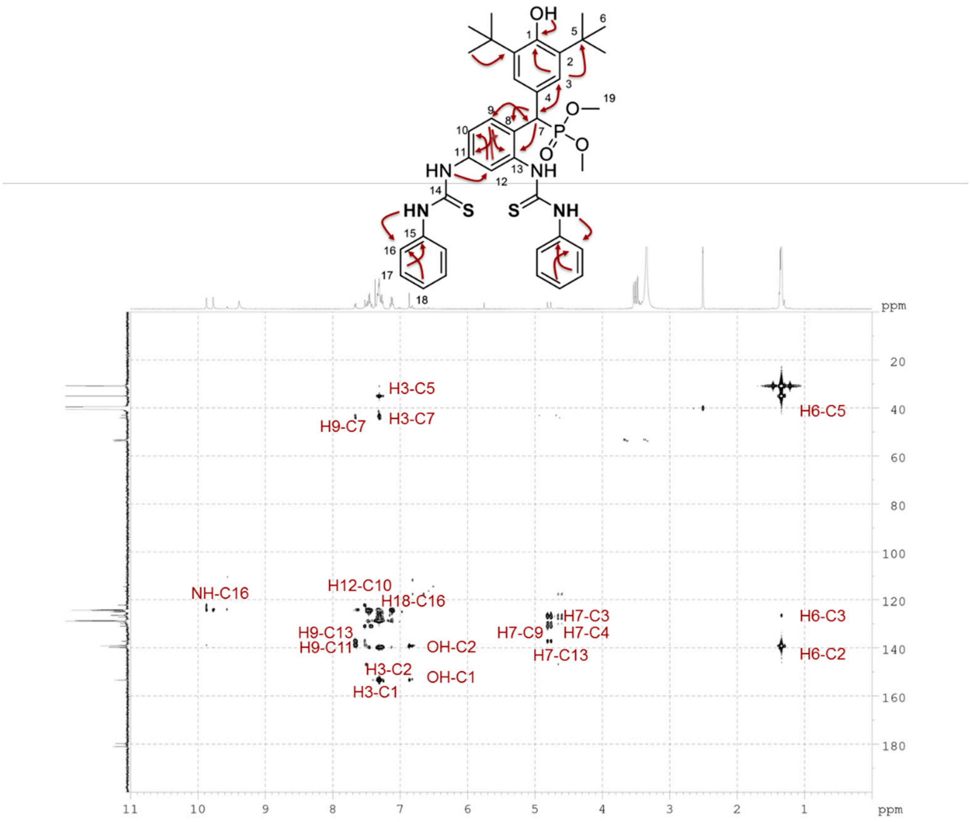


**Figure S95**. $^{1}$H- $^{13}$C HMBC correlations of compound **19a**.

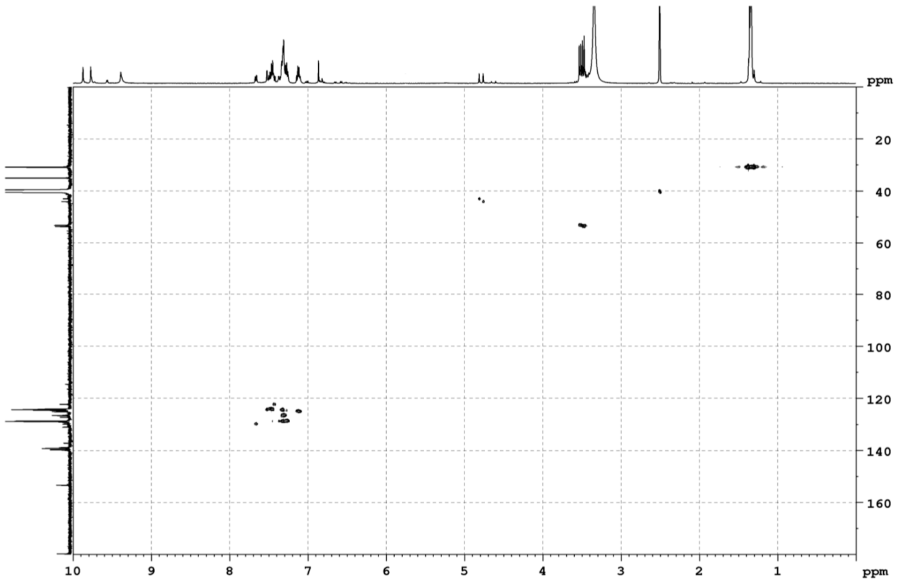


**Figure S96**.$^{1}H$- $^{13}C$ HSQC correlations of compound **19a**.

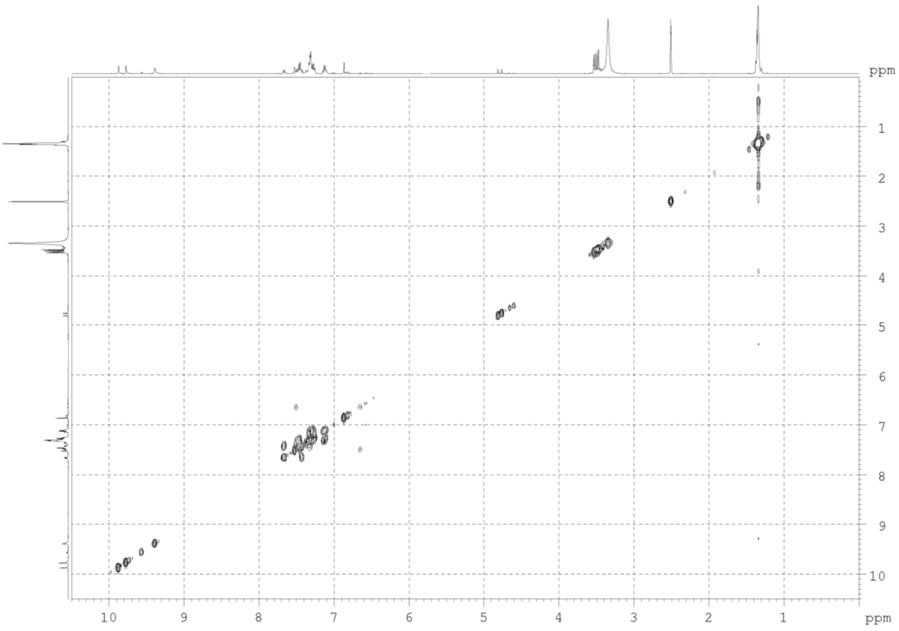


**Figure S97**. $^{1}H$- $^{1}H$ COSY correlations of compound **19a**.

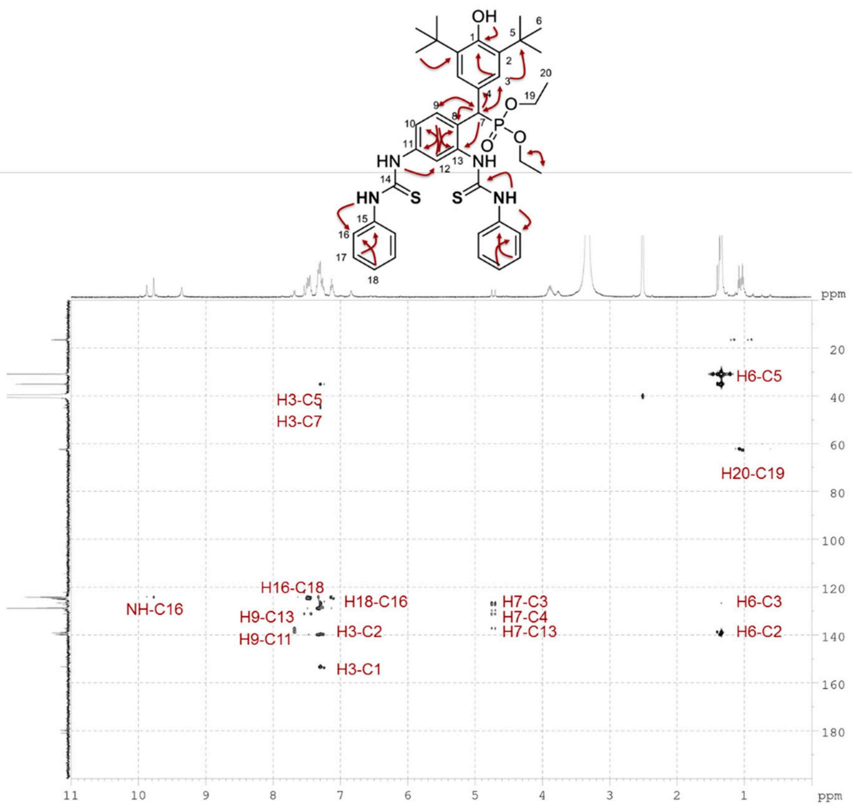


**Figure S98**. ¹H- ¹³C HMBC correlations of compound **19b**.

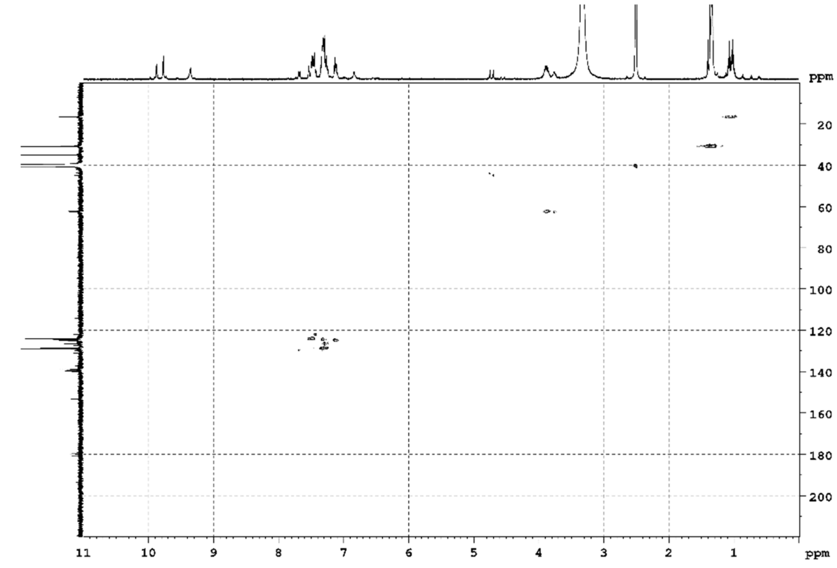

**Figure S99**. ¹H- ¹³C HSQC correlations of compound **19b**.

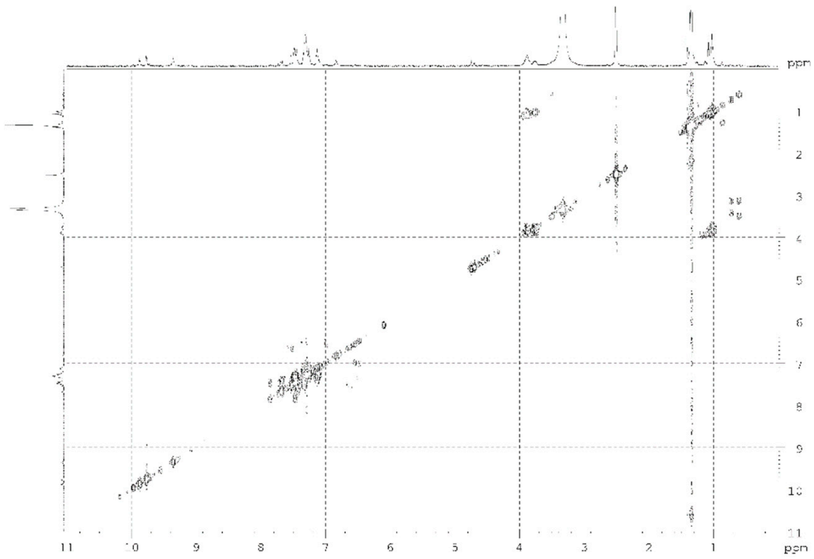

**Figure S100**. $^{1}$H- $^{1}$H COSY correlations of compound **19b**.

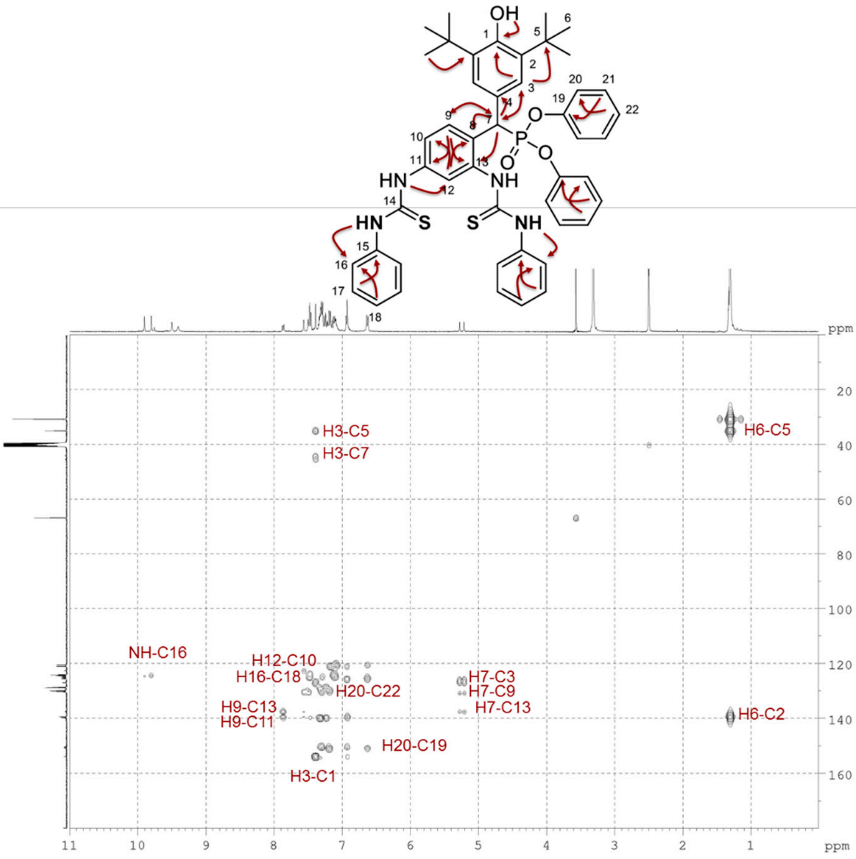


**Figure S101**. $^{1}$H- $^{13}$C HMBC correlations of compound **19d**.

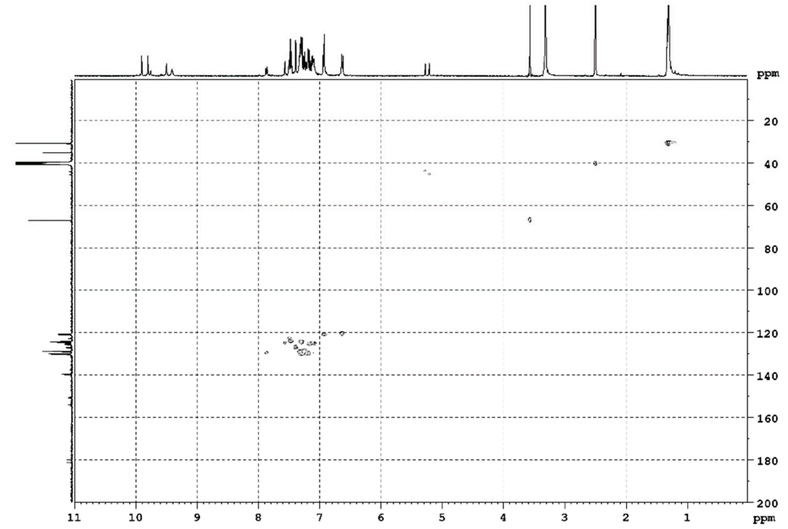


**Figure S102**. $^{1}H$- $^{13}C$ HSQC correlations of compound **19d**.

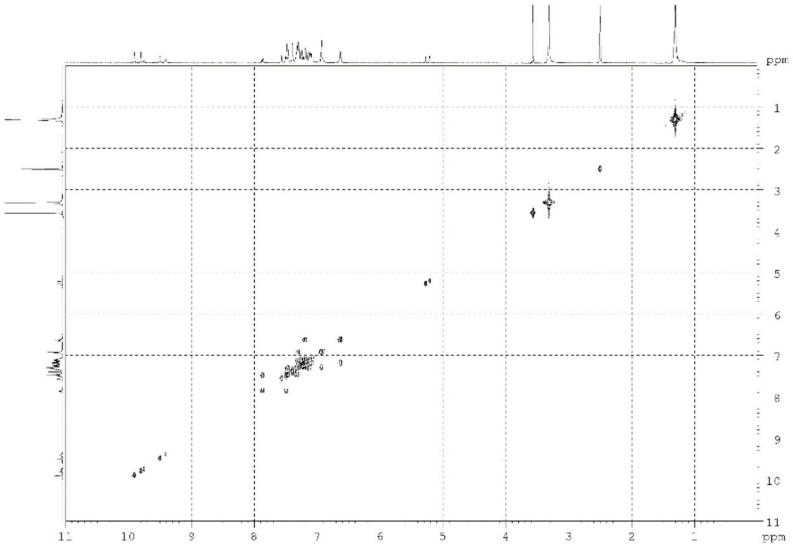


**Figure S103**. $^{1}H$- $^{1}H$ COSY correlations of compound **19d**.

References


1. Gibadullina, E.; Nguyen, T.T.; Strelnik, A.; Sapunova, A.; Voloshina, A.; Sudakov, I.; Vyshtakalyuk, A.; Voronina, J.; Pudovik, M.; Burilov, A. New 2,6-Diaminopyridines Containing a Sterically Hindered Benzylphosphonate Moiety in the Aromatic Core as Potential Antioxidant and Anti-Cancer Drugs. *Eur J Med Chem* **2019**, *184*, doi:10.1016/j.ejmech.2019.111735.

2. Gibadullina, E.M.; Shaekhov, T.R.; Badrtdinov, A.K.; Burilov, A.R. α-Phosphorylated 2,6-Di-Tert-Butyl-4-Methylidene-2,5-Cyclohexadienones in the Reactions with Meta-Phenylenediamine. *Russian Chemical Bulletin* **2014**, *63*, doi:10.1007/s11172-014-0619-2.

3. Sheldrick, G.M. SHELXTL v.6.12, Structure Determination Software Suite, Bruker AXS, Madison, WI, USA, 2000. *Bruker AXS, Madison, WI, USA, 2000]* **2000**.

4. Dolomanov, O.V.; B.L.J.; G.R.J.; H.J.A.K.; P.H.J. OLEX2: A Complete Structure Solution, Refinement and Analysis Program. *J Appl Crystallogr* **2009**, *42*, 339–341.

5. Frisch, M.J.; Trucks, G.W.; Schlegel, H.B.; Scuseria, G.E.; Robb, M.A.; Cheeseman, J.R.; Scalmani, G.; Barone, V.; Petersson, G.A.; Nakatsuji, H.; et al. Gaussian 16, Revision C.01: Gaussian Inc., Wallingford, CT. *Gaussian 16* 2016.

6. Zhao, Y.; Truhlar, D.G. Exploring the Limit of Accuracy of the Global Hybrid Meta Density Functional for Main-Group Thermochemistry, Kinetics, and Noncovalent Interactions. *J Chem Theory Comput* **2008**, *4*, doi:10.1021/ct800246v.

7. Zhao, Y.; Truhlar, D.G. Construction of a Generalized Gradient Approximation by Restoring the Density-Gradient Expansion and Enforcing a Tight Lieb-Oxford Bound. *Journal of Chemical Physics* **2008**, *128*, doi:10.1063/1.2912068.

8. Grimme, S.; Ehrlich, S.; Goerigk, L. Effect of the Damping Function in Dispersion Corrected Density Functional Theory. *J Comput Chem* **2011**, *32*, doi:10.1002/jcc.21759.

9. Schwabe, T.; Grimme, S. Double-Hybrid Density Functionals with Long-Range Dispersion Corrections: Higher Accuracy and Extended Applicability. *Physical Chemistry Chemical Physics* **2007**, *9*, doi:10.1039/b704725h.
